# Genome of *Drosophila suzukii*, the Spotted Wing Drosophila

**DOI:** 10.1534/g3.113.008185

**Published:** 2013-10-18

**Authors:** Joanna C. Chiu, Xuanting Jiang, Li Zhao, Christopher A. Hamm, Julie M. Cridland, Perot Saelao, Kelly A. Hamby, Ernest K. Lee, Rosanna S. Kwok, Guojie Zhang, Frank G. Zalom, Vaughn M. Walton, David J. Begun

**Affiliations:** * Department of Entomology and Nematology, College of Agricultural and Environmental Sciences, University of California, Davis, California 95616; † China National Genebank, BGI-Shenzhen, 518083, China; ‡ Department of Evolution and Ecology, College of Biological Sciences, University of California, Davis, California 95616; § Sackler Institute for Comparative Genomics, American Museum of Natural History, New York, New York 10024; ** Department of Horticulture, Oregon State University, Corvallis, Oregon 97331

**Keywords:** *Drosophila suzukii*, genome evolution, SpottedWingFlyBase, ortholog

## Abstract

*Drosophila suzukii* Matsumura (spotted wing drosophila) has recently become a serious pest of a wide variety of fruit crops in the United States as well as in Europe, leading to substantial yearly crop losses. To enable basic and applied research of this important pest, we sequenced the *D. suzukii* genome to obtain a high-quality reference sequence. Here, we discuss the basic properties of the genome and transcriptome and describe patterns of genome evolution in *D. suzukii* and its close relatives. Our analyses and genome annotations are presented in a web portal, SpottedWingFlyBase, to facilitate public access.

The fly genus *Drosophila* (Diptera: Drosophilidae) has served as the foundational model system in animal genetics for more than a century (Morgan 1910; Sturtevant 1913) and has been the source of crucial insights into many biological processes. In addition to the vast *Drosophila* literature that has accumulated, the model species *D. melanogaster* is associated with an extremely high-quality annotated reference sequence (Adams *et al.* 2000; Drosophila 12 Genomes Consortium 2007) and large array of experimental tools. Nevertheless, most of the knowledge derived from *Drosophila* studies has not been transferred to applied entomological problems because *Drosophila* has rarely been considered an economically important pest species.

*Drosophila suzukii* Matsumura (spotted wing drosophila) is an exception. Native to Southeast Asia, the species first invaded and became pervasive in the Hawaiian ecosystems in the early 1980s (Kaneshiro 1983; Leblanc *et al.* 2009). Since its first detection in mainland United States in 2008, *D. suzukii* has become a globally expanding invasive pest (Hauser 2011; Lee *et al.* 2011a; Calabria *et al.* 2012; Cini *et al.* 2012; Kiss *et al.* 2013). In Europe, *D. suzukii* was first detected in the Mediterranean countries (Spain, France, and Italy) in 2009, and by 2012 it had been reported in 11 other countries, including Russia (Cini *et al.* 2012; Rota-Stabelli *et al.* 2013). *D. suzukii* females exhibit an ovipositional preference for intact ripe and marketable fruit, unlike females of most other *Drosophila* species (Lee *et al.* 2011b; Hauser 2011; Burrack *et al.* 2013). This behavior is facilitated by the presence of a serrated ovipositor. Small (*e.g.*, caneberry) and soft-skin stone fruit (*e.g.*, cherry) producers have already reported significant crop losses throughout the United States, Canada, and Europe (Lee *et al.* 2011a; Calabria *et al.* 2012; Cini *et al.* 2012). Efforts to estimate the potential for economic damage have been difficult; however, in Pacific production regions, an estimated $500 million could be lost annually at 20% damage (Bolda *et al.* 2010; Walsh *et al.* 2011). As an example, revenues could be decreased by 37% for California commercial raspberries if *D. suzukii* is not managed during the production season (Goodhue *et al.* 2011). To our knowledge, this is the first time that such an invasive agricultural pest has been closely related to a model organism.

To facilitate the evolutionary genetic and applied analysis of this economically important pest, we have created a high-quality *D. suzukii* reference sequence. We describe the basic properties of the genome after having performed a comparative genomic analysis of *D. suzukii* with 14 of its close relatives in the *Sophophora* and *Drosophila* subgenera. In addition, to encourage the use of the reference sequence by a broad spectrum of basic and applied biologists, we have created a web portal, SpottedWingFlyBase, to disseminate our analysis and annotation of the *D. suzukii* genome.

## Materials and Methods

### Genome sequencing and assembly

We applied whole-genome shotgun sequencing using the Illumina HiSequation 2000. DNA was extracted from adult female *D. suzukii* from a strain that was established as an isofemale line from a female collected in Watsonville, California, in September 2009, and then inbred by sib-mating for 10 generations. The resulting strain, designated WT3, has been deposited in the Drosophila Species Stock Center. To reduce the risk of nonrandomness of clone coverage, we constructed seven paired-end libraries, with insert sizes of approximately 250 base pairs (bp), 300 bp, 500 bp, 2 kb, 9 kb, 10 kb, and 20 kb (Supporting Information, Table S1). In total, we generated approximately 78.28 G of data, and 38.91 G (176× coverage) of data were retained for assembly after filtering out low-quality and duplicated reads. The genome size *G* can be calculated from the formula *G* = *K_num_/K_depth_*, where *K_num_* is the total number of k-mers and *K_depth_* denotes the frequency that occurs most frequently (Li *el al*. 2010). A k-mer of length *K* refers to a *K*-nucleotide subsequence of a sequencing read. A raw sequence read with *L* bp contains (*L − K* + 1) k-mers if the length of each k-mer is *K* bp. The frequency of each k-mer can be calculated from the genome sequence reads. Typically, k-mer frequencies, when plotted against the sequence depth gradient, follow a Poisson distribution in a randomly sequenced dataset, although sequencing errors may lead to overrepresentation of low-frequency k-mers. In this work, *K* was 17, *K_num_* was 5,515,021,508, and *K_depth_* was 25; the *D. suzukii* genome size was therefore estimated to be 220 million bp (Figure S1 and Table S2).

The *D. suzukii* genome was *de novo* assembled by SOAPdenovo2 (Li *et al.* 2010), a short-read assembly method that uses the *de Bruijn* graph algorithm to simplify the task of assembly and to reduce computational complexity. First, reads with low quality were removed. We filtered out the following types of reads: reads from short insert-size libraries having an "N" more than 10% of its length and 20% for large insert-size libraries; reads from short insert-size libraries having more than 40% bases with quality (Q) ≤7 and reads from large insert-size libraries that contained more than 30% bases with (Q) ≤7; reads with more than 10 bp from the adapter sequence (allowing no more than 2 bp mismatches); short insert-size paired-end reads that overlapped ≥10 bp between the two ends (with the exception of 250 bp insert-size with PE 150 bp reads); and read 1 and read 2 of two paired-end reads that were completely identical (and thus considered to be the products of PCR duplication). After these quality-control and filtering steps, a total of 38.91 Gb (or 176×) data were retained for assembly. SOAPdenovo first constructs the *de Bruijn* graph by splitting the reads from short insert-size libraries (250–500 bp) into 41-mers and then merging the 41-mers; contigs that exhibit unambiguous connections in *de Bruijn* graphs are then collected. All reads were aligned onto the contigs for scaffold building using the paired-end information. This paired-end information was subsequently used to link contigs into scaffolds, iteratively, from short insert sizes to long insert sizes. Approximately 20.49 G (or 93×) of data were used to build contigs, whereas all high-quality data were used to build scaffolds. Some intra-scaffold gaps were filled using local assembly from the reads in a read-pair, where one end uniquely aligned to a contig while the other end was located within the gap. The final total contig size and N50 were 204.9 million bp and 23.2 kb, respectively. The final total scaffold size and N50 were 235.5 million bp and 385.2 kb, respectively (Table S3). To assess assembly quality, high-quality reads from short insert libraries (250–500 bp) were aligned onto the assembly using the Burrows-Wheeler Alignment Tool version 0.6.2 (Li and Durbin 2009) with default parameters. A total of 93.47% reads could be unambiguously mapped, and they covered 99.59% of the assembly, excluding gaps.

### Transcriptome sequencing, assembly, and gene expression differences between sexes

Total RNA was extracted separately from 2-d-old virgin female and male *D. suzukii* adults. After polyA RNA enrichment, paired-end libraries with an approximate average insert length of 170 bp were created. Libraries were sequenced using 100 bp paired-end Illumina HiSeq sequencing. Male and female RNA sequencing reads were filtered based on quality score. We required minimum base Q >20 and average Q for reads >35. Identical duplicate reads were removed. The *de novo* transcriptome assemblies were created using the *de Bruijn* graph-based assembler (Trinity release 2013-02-25) (Grabherr *et al.* 2011). Assembly was performed using high-quality, cleaned, and filtered paired-end sequences with a fixed k-mer size of 25; minimum k-mer coverage was 3 and minimum isoform ratio was 0.05. Assembled contigs with at least 200 bp were kept. We used Tophat version 2.0.6 (Trapnell *et al.* 2009) to map reads to the *D. suzukii* genome assembly. Parameters for Tophat were as follows: segment length = 40; initial read mismatch = 2; splice mismatch = 0; segment mismatch = 2; maximum insertion length = 1; and maximum deletion length = 1. This was followed by differential expression analysis using Cuffdiff version 2.0.0 (Trapnell *et al.* 2010), with upper-quartile normalization and a false discovery rate of 0.05. An inference of sexually dimorphic expression required at least two-fold expression difference between sexes with at least one sex showing expression FPKM >2.

### Annotation of genome and transcriptome

Genome annotation was performed using the MAKER2 pipeline (Holt and Yandell 2011). Augustus version 2.5.5 (Stanke *et al.* 2008), SNAP (release 2013-02-16) (Korf 2004), and GeneMark-ES version 2.3e (Lomsadze *et al.* 2005) were used as *ab initio* gene predictors. Our *D. suzukii* transcriptome and *D. melanogaster* protein sequences from FlyBase (release FB2013_01) were used as transcript and homology-based evidence, respectively. To evaluate our annotation for completeness, a set of 458 core eukaryotic genes (Parra *et al.* 2007) were searched against our annotated protein set using BLASTP. Syntenic relationships between *D. suzukii* scaffolds and *D. melanogaster* chromosomes were examined using SyMAP version 4.0 (Soderlund *et al.* 2011) with a minimum size of 500 kb.

### Comparative genomics analysis and functional annotation using OrthologID

Gene orthology was evaluated using prereleased version 2.0 of the OrthologID pipeline. Similar to the original version (Chiu *et al.* 2006), the latest version of OrthologID takes complete gene sets from all ingroup and outgroup taxa as input and assigns them into gene clusters. In this analysis, *Anopheles gambiae* was used as the outgroup, and the ingroup taxa included 14 species spanning multiple groups in the subgenus *Sophophora* (*D. melanogaster*, *D. simulans*, *D. sechellia*, *D. yakuba*, *D. erecta*, *D. ananassae*, *D. pseudoobscura*, *D. persimilis*, *D. willistoni*, *D. takahashii*, *D. biarmipes*) and subgenus *Drosophila* (*D. virillis*, *D. mojavensis*, *D. grimshawi*). The complete gene set for *A. gambiae* and those for *D. simulans*, *D. sechellia*, *D. yakuba*, *D. erecta*, *D. ananassae*, *D. pseudoobscura*, *D. persimilis*, *D. willistoni*, *D. mojavensis*, *D. virillis*, *D. grimshawi*, and *D. melanogaster* were retrieved from VectorBase (Megy *et al.* 2012) and FlyBase (Marygold *et al.* 2013), respectively. To generate gene sets for *D. takahashii* and *D. biarmipes*, we downloaded genome assemblies from GenBank and transcriptomes from the *Drosophila* modENCODE Project (modENCODE consortium *et al.* 2010), and then annotated using MAKER2 (Holt *et al.* 2011) with the same *ab initio* gene predictors and protein homology–based evidence as our *D. suzukii* annotation. OrthologID then performed sequence alignment using MAFFT version 7.017b (Katoh and Standley 2013) and parsimony phylogenetic inference using PAUP* (Swofford 2002) for each gene cluster and extracted one or more sets of orthologous genes from each cluster based on the gene tree topology. In addition to improved execution pipeline on Sun Grid Engine clusters, this version of OrthologID used the MCL algorithm (Enright *et al.* 2002; Van Dongen 2008) for improved clustering and included automated extraction of orthologs from gene trees into a partitioned matrix in a single package. Edge weights of the MCL graph were functions of BLAST E-values with a cutoff of 1E−10. Using gene sets from 16 species as input, OrthologID recovered 13,941 sets of orthologs with at least 4 taxa represented from 13,264 gene clusters. Among the identified ortholog sets, 5322 of them had all ingroup taxa represented. Functional annotations of *D. suzukii* genes, including Gene Ontology (GO) terms, were generated from the FlyBase (FB2013_02) annotations of *D. melanogaster* orthologs identified by OrthologID as described.

### Construction of SpottedWingFlyBase

The SpottedWingFlyBase web site was developed on the WordPress publishing platform (wordpress.org) and includes a custom gene search engine written in PHP with a MySQL (mysql.com) database backend, BLAST service using the Ruby-based SequenceServer (A. Priyam, B. J. Woodcroft, Y. Wurm, unpublished data) in conjunction with NCBI BLAST+ (Altschul *et al.* 1990; Camacho *et al.* 2009), graphical gene tree rendering using the jsPhyloSVG javascript library (Smits and Ouverney 2010), Jalview 2 (Troshin *et al.* 2011) applet as the alignment viewer, and GBrowse 2 (Stein *et al.* 2002) as the genome annotation viewer.

### Phylogenetic analysis

For inference of the species phylogeny, we performed maximum likelihood (ML) analysis on a matrix of 5322 fully represented gene partitions with 5,199,249 sites assembled by OrthologID. The best protein substitution model for each gene partition was selected individually using the “ProteinModelSelection.pl” script (Stamatakis 2012) over 36 different models. Partitioned analyses with Γ-distributed rate heterogeneity over sites were performed using RAxML version 7.4.2 (Stamatakis 2006, 2012). Rapid bootstrap with 250 bootstrap replicates was performed using the MPI-AVX version of RAxML, and the PTHREADS-AVX version was used to search for the best scoring trees. A highly supported topology with bootstrap value of 100 at every node was recovered.

To estimate branch lengths and to explore rate of evolution among *D. suzukii*, *D. biarmipes*, and *D. takahashii*, we conducted a separate partitioned ML analysis in RAxML using 4919 codon-aligned one-to-one orthologous gene sets predicted by OrthologID, with *D. melanogaster* as the outgroup. Each gene was treated as a partition, which allowed parameters of the general time-reversible (GTR) substitution model (with Γ distribution of rate variation among sites) to be estimated independently for each locus. We partitioned the orthologs into three data sets: X-linked; autosomal; and a combined data set. Of the 4919 protein-coding genes, 802 were identified as likely X-linked based on conservation of Muller elements (Bhutkar *et al.* 2008) and location of the orthologous gene on the *D. melanogaster* X chromosome.

### Codon analysis

To examine the variation in synonymous and nonsynonymous substitutions, we calculated *d*N and *d*S using the “seqinr” package (Charif and Lobry 2007) in R 3.01 (r-project.org). This program used an unbiased rate estimator to calculate synonymous and nonsynonymous changes between two protein-coding sequences (Li 1993). These metrics were calculated for the overall codon-aligned data set described and also for partitioned data to include only X-linked or autosomal genes. We compared *d*N and *d*S among pairs of taxa with nonparametric Mann-Whitney *U* tests. The ortholog data sets were analyzed with the program CodonW version 1.3 (Peden 1999) to estimate GC content of third positions for each synonymous codon (GC_3_). We also investigated sex-specific expression of genes, fragments per kb of transcript per million mapped reads (FPKM) and the associated GC content of synonymous third codon positions.

### Analysis of gene family expansion and contraction in *D. suzukii*

Using gene clusters produced by OrthologID, we computed the expansion and contraction of *D. suzukii* gene families using the difference (*∆*) between the number of *D. suzukii* genes (*N_suz_*) and the median number of genes (*Ñ*) in other groups of *Drosophila* for each family (*∆* = *N_suz_* − *Ñ*). The median numbers for three *Drosophila* groups were computed corresponding to all *Drosophila* species included in our analysis, except for *D. suzukii*, the more basal paraphyletic group encompassing *D. ananassae*, *D. pseudoobscura*, *D. persimilis*, and *D. willistoni* from the subgenus *Sophophora*, and *D. virillis*, *D. mojavensis*, and *D. grimshawi* from the subgenus *Drosophila*, and the *melanogaster* subgroup including *D. simulans*, *D. sechellia*, *D. yakuba*, *D. erecta*, *and D. melanogaster*. The expansion (∆ ≥ 2) and contraction (∆ ≤ 2) lists of *D. suzukii* genes against these three groups were then evaluated for overrepresented GO terms and functional-related gene groups using DAVID version 6.7 (Database for Annotation, Visualization, and Integrated Discovery) (Huang *et al.* 2009a; Huang *et al.* 2009b). We annotated each gene family with a representative *D. melanogaster* gene by choosing the gene with the largest number of GO terms in FlyBase’s annotation. The FlyBase gene IDs of all representative genes were used as the background list in our enrichment analysis. We used the default parameters in the DAVID Functional Annotation Clustering tool, except for a value of 0.1 for the EASE score, a modified Fisher exact *P* value. Because DAVID analysis relies on functional annotation, gene families with no annotation or known sequence features were not included in our analysis. These include gene families with no identifiable orthologs in *D. melanogaster*, as determined by OrthologID, as well as gene families with *D. melanogaster* orthologs that do not have GO/PIR annotations.

### Identification of retrogenes

We combined all assembled transcripts and annotated genes to generate a set of nonredundant genes. Genes associated with open reading frames (ORFs) <100 amino acids were discarded. All gene sequences were aligned to the genome using blastn (cutoff lE−5). To identify relatively young duplicates, we required the best blast hit to show at least 75% similarity and overlap more than 70% of the query length. We then extracted all genes that had two or more such blast hits. Candidate retrogenes were required to align to at least 70% length of the coding regions (CDS) of a parental multi-exon gene and to be intronless. Furthermore, we required putative *D. suzukii* retrogenes to show no evidence of existence of a homologous retrogene in the *Drosophila* 12 genomes annotations (Drosophila 12 Genomes Consortium 2007). For all candidates, we checked for an ORF and used Genewise version 2.2.0 (Birney and Durbin 2000) to define the beginning and the end of the gene.

### Transposable element detection

Transposable elements (TEs) were detected by first aligning the set of 6003 TEs found in the *D. melanogaster* reference sequence plus the common elements *p* and *kp* to the *D. suzukii* scaffolds (blastall -p tblastx -f 999 -F “" -E0.00001) (Altschul *et al.* 1990). We aligned only to scaffolds that were a minimum of 5 kb in length. The -F option prevented blastall from using its complexity filter and the -f and -e options were our stringency requirements for keeping alignments. After the initial blastall procedure, we identified all regions of contiguous sequence with a minimum 50% identity to any TE in the *D. melanogaster* reference. These sections of contiguous sequence were then extracted along with 500 bp upstream and downstream of the identified sequence and aligned to the set of *D. melanogaster* TEs using the same parameters as noted.

### TE family identification

We then used this output to identify contiguous regions aligning to TEs at two levels, 50% identity and 80% identity. These sequences were then extracted and realigned to the set of *D. melanogaster* TEs to determine the TE family to which they were most similar. We identified TE family based on the highest alignment, with a minimum 80% identity, over the longest portion of the identified TE sequence. We also required a minimum of 80 bp of contiguous sequence to be considered a TE. We then calculated the total number of base pairs of each TE family at both 50% identity and 80% identity and calculated the percentage of the scaffolds represented by each family at these two alignment criteria. We also plotted the number of base pairs of TE per 100 kb of scaffold for all scaffolds of 5 Mb or more.

## Results and Discussion

### Genome assembly and annotation

The genome assembly described here is of substantially higher quality by all measures compared to the previously published assembly (Ometto *et al.* 2013). Chiu *et al.* (2006) described the following: contig N50 = 23.2 kb; contig maximum size = 472 kb; and *D. melanogaster* ortholog identified = 12,389. This was compared with the work of Ometto *et al.* (2013), who described the following: contig N50 = 4.5 kb; contig maximum size = 92.8 kb; and *D. melanogaster* ortholog identified = 8,137. We identified 13,583 protein-coding genes, of which 12,984 (96.4%) had BLAST hits (E-value < 1E−10) in the other 14 *Drosophila* species included in this study, including 12,389 in *D. melanogaster* (91.2%). To assess the quality and completeness of our assembly, we evaluated the presence of a set of 458 CEGMA core eukaryotic genes (CEGs) as defined by Parra *et al.* (2007); 449 of 458 CEGs (98%) had BLASTP hits (E-value < 1E−10) in our annotated protein set. Synteny between *D. suzukii* and *D. melanogaster* genomes was analyzed for scaffolds >500 kb. A total of 160 synteny blocks were identified by SyMAP version 4.0 (Soderlund *et al.* 2011), covering 93% of *D. suzukii* scaffolds >500 kb and 69% of *D. melanogaster* chromosomes. Of the 160 synteny blocks, 58 were inverted. A circular representation of the synteny map is shown in Figure S2. The low number of synteny blocks that map to chromosome 3R of *D. melanogaster* may be attributable to the small size of *D. suzukii* scaffolds that are aligned to 3R.

### SpottedWingFlybase: Web portal for *D. suzukii* genomics

#### Web portal:

**Figure 1:**
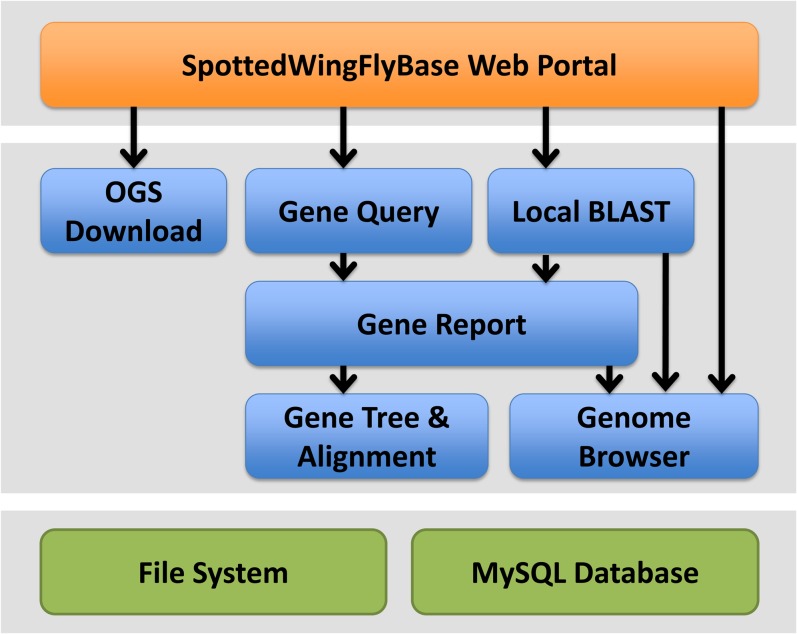
Architecture overview of the SpottedWingFlyBase web portal. The blue modules are components available on the portal menus or reachable through other modules as indicated by the arrows. The bottom layer in green shows the underlying data storage.

Our *D. suzukii* genome data are available through the SpottedWingFlyBase portal (SWFBase; http://spottedwingflybase.oregonstate.edu). The reads and genome sequence are available from Genbank. The Whole Genome Shotgun project has been deposited at DDBJ/EMBL/GenBank under the accession number AWUT00000000. The version described in this paper is version AWUT01000000. The associated reads can be found under SRA096061. The project accession number is PRJNA213258. RNA-seq reads from females and males can be found under accession numbers SRR1002988 and SRR1002989, respectively. In addition to the availability of the Official Gene Set (OGS1.0), which includes the genome assembly as well as transcript and protein sequences from our genome annotation, SWFBase incorporates multiple data mining and visualization tools that allow those researching to retrieve and visualize the *D. suzukii* genome data and OrthologID-generated gene orthology and phylogenetic results of interests. Relationships between various SWFBase components are shown in Figure 1.

#### Gene search and gene report:

*D. suzukii* genes are searchable using the flexible Gene Search function. OGS Gene ID, gene symbol, gene symbol prefix, or any key words that describe a gene of interests can be used as search terms. A matching list of *D. suzukii* genes are returned, each of which is linked to a Gene Report. This Gene Report includes description of the gene, its predicted *D. melanogaster* ortholog as identified by OrthologID, the protein and transcript sequences, and a link to the OrthologID Gene Family page of the gene cluster it belongs to.

#### Gene family:

Each Gene Family page is linked from the Gene Reports of its gene family members. The Gene Family page allows those researching to view and download the gene family tree and alignment produced by OrthologID. A gene count table that contains the number of genes belonging to each species is also presented to allow researchers to easily recognize gene expansion or contraction that may be present in any species or subgroups.

#### Genome browser:

The *D. suzukii* assembly and annotated genes in their genomic context can be viewed using the embedded genome browser. Genomic features available include gene regions, exons, introns, and untranslated regions (UTRs). *D. melanogaster* protein homologs used as evidence in gene prediction are also available as a separate track.

#### Local BLAST server:

SWFBase users can search against the *D. suzukii* assembly and the OGS1.0 transcripts and proteins using the local BLAST service. To facilitate the retrieval of BLAST hits, the results also contain link-outs to the Genome Browser showing the aligned regions of the assembly (for BLAST against assembly) or to Gene Reports of the corresponding *D. suzukii* hits (for BLAST against OGS transcripts or proteins).

### Phylogenetic placement and substitution rates in *D. suzukii*

**Figure 2:**
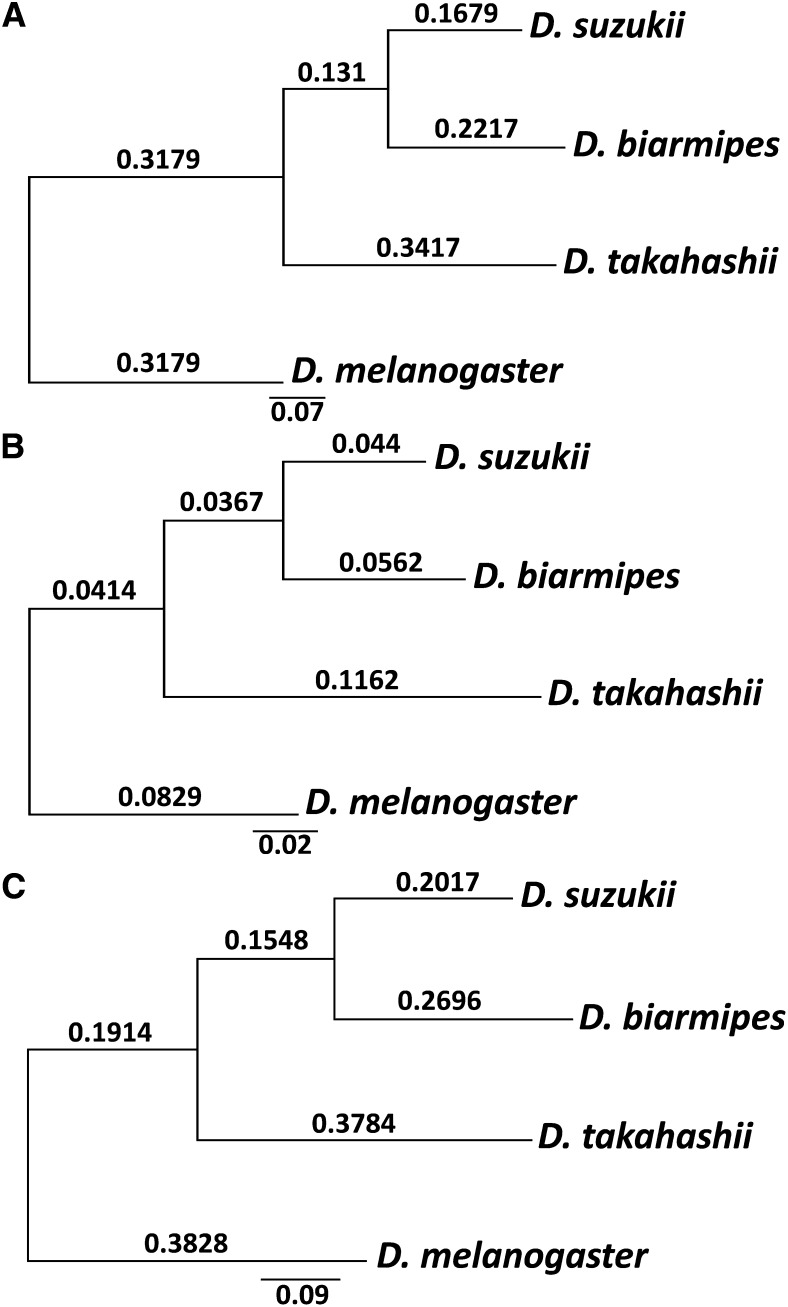
Phylogenetic relationships and evolutionary rates for *D. suzukii*, *D. biarmipes*, and *D. takahashii* with *D. melanogaster* as outgroup. Analysis performed based on (A) 4919 orthologous genes, combining genes on both X and autosomes, (B) X-linked genes only, and (C) autosomal genes only. Numbers above branches indicate branch lengths.

The phylogeny of 15 *Drosophila* species including *D. suzukii* was inferred using ML on a 5322-partition matrix with 5,199,249 sites assembled by OrthologID. Our topology is in general agreement with previously published *Drosophila* phylogenies based on genomic scale data (Drosophila 12 Genomes Consortium 2007; Ometto *et al.* 2013), with strong support at all nodes (100% bootstrap) (Figure S3). Our analysis supports a sister relationship between *D. suzukii* and *D. biarmipes*, as proposed by previous research (Yang *et al.* 2012). Previous work using 91 protein-coding genes suggested that the substitution rate in the lineage leading to *D. suzukii* was shorter than that in the lineage leading to *D. biarmipes* (Ometto *et al.* 2013). We obtained similar results using 4919 orthologous genes, with 11.5 million aligned nucleotides (for each taxon) encoding predicted proteins ranging from 37 to 9094 amino acids and a median length of 492 (Figure 2A). However, when X-linked and autosomal genes were analyzed separately, we observed that the effect was substantially greater for the autosomes (Figure 2C); X-linked substitution rates were very similar in the two lineages (Figure 2B).

### Analysis of codon usage

**Figure 3:**
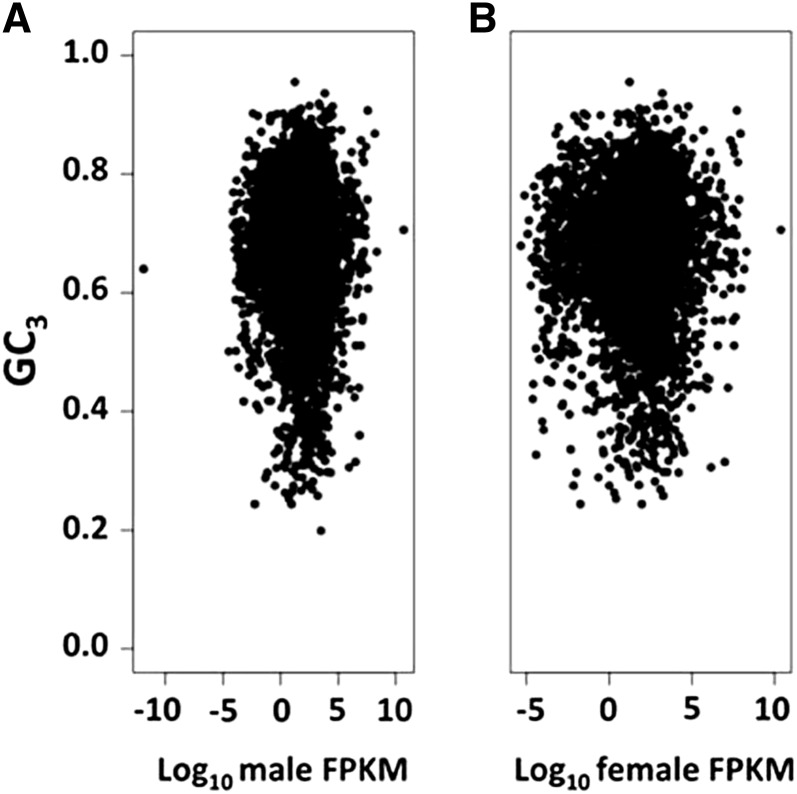
Relationship between GC content and gene expression level for *D. suzukii*. GC content at third codon position (GC_3_) as a function of log_10_ fragments per kilobase of transcript per million mapped reads (FPKM) for (A) male and (B) female *D. suzukii*.

To investigate lineage effects for protein evolution and synonymous evolution, we compared nonsynonymous (*d*N) and synonymous (*d*S) substitution rates in several pairwise comparisons (Table 1). These results show that the slower autosomal substitution rate for *D. suzukii* is similar in magnitude for *d*N and *d*S. Also notable is the observation that the two pairwise comparisons with *D. takahashii* exhibited a much greater substitution rate on the *X* than on the autosomes for nonsynonymous and synonymous sites. However, this effect was much weaker in the *D. suzukii* and *D. biarmipes* comparison, which suggested that the large faster-*X* effect is primarily a *D. takahashii* lineage phenomenon. The faster-*X* effect observed here was considerably greater than that previously observed in *Drosophila* (Mank *et al.* 2010). The *d*N/*d*S ratios observed in all comparison were similar in magnitude to those reported in other *Drosophila* lineages (Mank *et al.* 2010). Notably, the *d*N/*d*S ratios were consistently greater on the *X* chromosome than on the autosomes. Given the general observation that a substantial proportion of protein divergence is adaptive in *Drosophila* (Smith and Eyre-Walker 2002; Fay *et al.* 2002; Begun *et al.* 2007; Langley *et al.* 2012), one interpretation of increased *d*N/*d*S ratio on the *X* is more adaptive protein evolution on the *X* chromosome. To investigate possible connections between codon bias and dS, we estimated CG_3_ for *X*-linked and autosomal *D. suzukii* genes. As observed previously in *Drosophila*, we found higher GC content in the third positions of *X*-linked genes compared to autosomal genes (*P* < 2.2 × 10^−16^) (Figure S4A). Because GC-ending codons are generally enriched in high-bias genes in *Drosophila* (Shields *et al.* 1988; Moriyama and Powell 1997) and in *D. suzukii* (GC_3_ *vs.* CBI: *R*^2^ = 0.92, *P* < 2.2 × 10^−16^), our observation was consistent with increased efficacy of selection on codon bias on the *D. suzukii X* chromosome (Singh *et al.* 2005). This is consistent with the lower *d*S value for *X*-linked genes *vs.* autosomal genes in the *D. suzukii* and *D. biarmipes* comparison (Table 1). To investigate the possible effect of gene expression variation on codon bias, we compared FPKM to GC_3_ (Figure 3) and found no correlation between expression in either sex and GC_3_. We also observed no difference in GC_3_ for male-biased genes when compared to other *D. suzukii* genes (Figure S4B). We observed that male-biased X-linked genes were underrepresented relative to the number of X-linked genes in the orthologous data set (χ^2^ = 4.43, *P* = 0.0361, determined by 1 M Monte Carlo simulations) (Hope 1968). We observed no association between male and female FPKM and GC_3_ content (Figure 3).

**Table 1:** Synonymous and nonsynonymous estimates and ratios (dN/dS) for comparisons among *Drosophila suzukii*, *D. biarmipes*, *D. takahashii*, and *D. melanogaster*

	***d*N**		***d*S**		***d*n/*d*S**	
	**X*****^a^***	**A*****^b^***	**X**	**A**	**X**	**A**
Dsuz-Dbia	0.022836	0.021361	0.241297	0.251222	0.094965	0.089532
Dsuz-Dtak	0.047266	0.032531	0.433947	0.377741	0.115737	0.090204
Dbia-Dtak	0.048836	0.035515	0.435043	0.404029	0.117354	0.093660
Dsuz-Dmel	0.056061	0.048341	0.520079	0.569554	0.110473	0.090889

*d*N, nonsynonymous; *d*S, synonymous; Dsuz, *Drosophila suzukii*; Dbia, *D. biarmipes*; Dtak, *D. takahashii*; Dmel, *D. melanogaster*.

a X-linked genes.

b Autosomal genes.

We also examined the 25 genes with the highest *d*N rates for functional similarities that may suggest a biological context for the observed rates. We used DAVID version 6.7 (http://david.abcc.ncifcrf.gov) to conduct functional gene classification (Huang *et al.* 2009a; Huang *et al.* 2009b). The majority of these genes (16) had no known molecular function, only five had gene names, and the DAVID analysis failed to detect any functional commonalities among them (Table S4).

### Sexual dimorphic expression in *D. suzukii*

**Figure 4:**
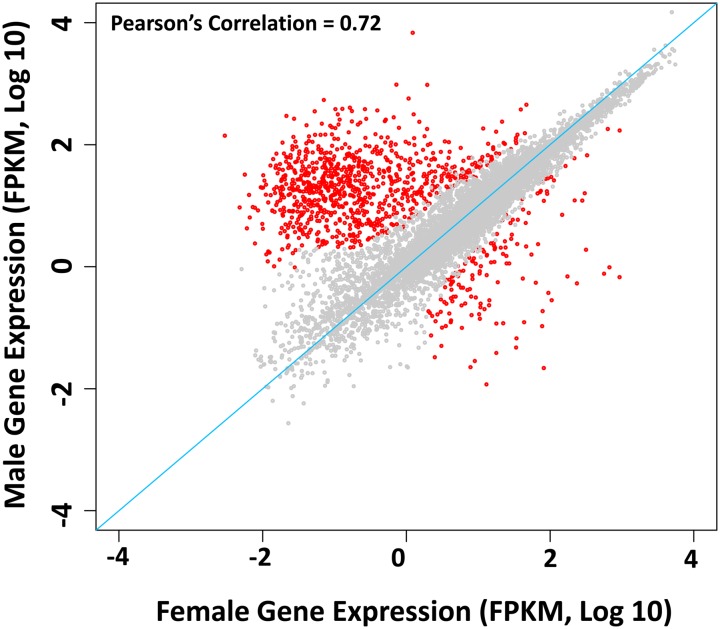
Sex-biased genes of *D. suzukii*. Correlations between whole female and whole male expression (FPKM, log_10_) are plotted (red, sex-biased genes; gray, nonbiased genes). Pearson correlation *r* = 0.72.

Sexual dimorphic traits play key roles in animal evolution and behavior. The development of a trait in one sex and not the other must be the result of differential gene expression between males and females (Williams *et al.* 2008). To understand sex-biased gene expression in *D. suzukii*, we compared the RNA expression of 2-d-old adult females and males. We identified 1399 genes that showed sexually dimorphic expression, which we define as at least a two-fold difference between sexes (Figure 4 and Table S5). This is approximately 10.3% of the total annotated genes (n = 13,583) and 13.1% of the genes expressed in adults (n = 10,705). Of these 1399 genes, 150 showed female-biased expression and 1249 showed male-biased expression. To identify possible cases of genes that evolved in sex-biased expression between *D. melanogaster* and *D. suzukii*, we compared the expression pattern of all orthologs expressed in both species and found 22 genes that showed sex-biased expression switch (male to female or female to male) (Table S6). Four genes were male-biased in *D. melanogaster* but were female-biased in *D. suzukii*, whereas 18 were female-biased in *D. melanogaster* but were male-biased in *D. suzukii*.

### Gene family expansion and contraction in *D. suzukii*

We assessed gene family expansions and contractions by comparing gene counts per gene family/cluster in all *Drosophila* species included in this study. We identified GO terms and functional annotations (SwissProt PIR and InterPRO) that were enriched in gene families that expanded (Table 2) or contracted (Table 3) in *D. suzukii* as compared to other *Drosophila* species. Three separate comparisons were performed for expansion and contraction, respectively. First, gene count for each gene family in *D. suzukii* was compared to the median gene count computed from all other *Drosophila* species used in this study (Table 2A, Table 3A, Table S7, and Table S10). Gene counts of all *D. suzukii* gene families were also compared to median gene counts for corresponding gene families computed from the more basal paraphyletic group, including six species outside of the *melanogaster* group plus *D. ananassae* (Table 2B, Table 3B, Table S8, and Table S11), as well as that from five species in the *melanogaster* subgroup, respectively (Table 2C, Table 3C, Table S9, and Table S12). GO terms and functional annotations that are enriched in gene families that were identified to be expanded or contracted in *D. suzukii* based on all three comparisons are more likely related to adaptation specific to the *D. suzukii* lineage, whereas those that are only present or absent in more restrictive comparisons may reflect gene family evolution events in a larger group of species.

**Table 2:** GO term and functional classification enrichment analysis using DAVID for gene families that are expanded in the *Drosophila suzukii* genome as compared to other *Drosophila* species

**Annotation Cluster**	**Enrichment Score**	**Category***^**a**^*	**Term***^**b**^*	**Count***^**c**^*	***P***	**Gene Families: Representative Gene Symbols***^**d**^*
**A. Comparison to 14 *Drosophila* genomes**						
1	1.81	GOTERM BP	GO:0006071 glycerol metabolic process	3	8.49E−03	Gpdh, CG18135, Gyk
2	1.75	GOTERM BP	GO:0032268 regulation of cellular protein metabolic process	9	3.85E−03	Acp76A, PEK, Adam, mRpL11, Tollo, Spn77Bc, Su(var)205, nos, elav
		GOTERM BP	GO:0010605 negative regulation of macromolecule metabolic process	13	4.11E−03	WRNexo, Acp76A, mRpL11, Spn77Bc, Su(var)205, elav, mael, Hsc70-4, PEK, E(spl)m8-HLH, fkh, lolal, nos
3	1.58	SwissProt PIR	Chaperone	5	1.54E−02	CG7394, CG11267, Cnx99A, Tcp-1zeta
		GOTERM BP	GO:0006457 protein folding	6	2.69E−02	Hsc70-4, CG11267, Cnx99A, Tcp-1zeta, FKBP59
4	1.42	GOTERM BP	GO:0051606 detection of stimulus	6	1.26E−02	Or49a, Calx, CheB42a, Galphaq, FKBP59
		SwissProt PIR	Sensory transduction	6	3.13E−02	Or49a, Or69a, Galphaq, FKBP59, Or22a
5	1.37	INTERPRO	IPR007087 zinc finger, C2H2-type	13	2.49E−02	CG6689, term, CG1647, CG16779, CG10669, Meics, CG5316, CG4360, CG3065, CG11966, CG4318
		INTERPRO	IPR015880 zinc finger, C2H2-like	13	2.93E−02	CG6689, CG1647, CG16779, CG10669, Meics, CG5316, CG4360, CG3065, CG11966, noi, CG4318
6	1.22	SwissProt PIR	Sensory transduction	6	3.13E−02	Or49a, Or69a, Galphaq, FKBP59, Or22a
		GOTERM MF	GO:0005549 odorant binding	6	3.17E−02	Obp83ef, Or49a, Obp51a, CheB42a, Or69a, Or22a
		GOTERM BP	GO:0007186 G-protein-coupled receptor protein signaling pathway	10	4.14E−02	Or49a, Tk, D2R, Or69a, Galphaq, Gr85a, mth, Pk1r, Or22a
7	1.2	SwissProt PIR	Mitochondrion inner membrane	5	1.41E−02	CG7394, ATPsyn-d, ATPsyn-b, Oscp, CG9603
		GOTERM CC	GO:0005753 mitochondrial proton-transporting ATP synthase complex	3	5.02E−02	ATPsyn-d, ATPsyn-b, Oscp
8	1.14	GOTERM MF	GO:0004867 serine-type endopeptidase inhibitor activity	4	4.68E−02	Acp76A, CG31515, Spn77Bc, CG3604
**B. Comparison to *D. ananassae*, *D. persimilis*, *D. pseudoobscura*, *D. willistoni*, *D. grimshawi*, *D. mojavensis*, and *D. virulis***						
1	2.32	GOTERM BP	GO:0032268 regulation of cellular protein metabolic process	10	1.56E−03	Acp76A, PEK, Acp62F, Adam, mRpL11, Tollo, Spn77Bc, Spn27A, nos, elav
		GOTERM BP	GO:0010605 negative regulation of macromolecule metabolic process	13	7.20E−03	WRNexo, Acp76A, Acp62F, mRpL11, Spn77Bc, Spn27A, elav, Hsc70-4, PEK, E(spl)m8-HLH, fkh, lolal, nos
2	2.27	GOTERM BP	GO:0006952 defense response	9	2.76E−03	CG6168, Sr-CI, Tollo, Drs, Lectin-galC1, TotA, TotM, Spn27A, GNBP1
		GOTERM BP	GO:0006955 immune response	9	3.16E−03	Sr-CI, Tollo, Drs, Lectin-galC1, Rac1, TotA, TotM, Spn27A, GNBP1
3	1.88	SwissProt PIR	Glycoprotein	17	9.71E−03	Acp76A, Acp29AB, D2R, Gr59c, Mal-A2, Gr85a, TotA, Hsc70-4, PEK, scb, y, pgant3, Drs, CG32669, rt, mth, GNBP1
		GOTERM CC	GO:0005576 extracellular region	16	1.88E−02	Acp76A, Acp62F, Acp29AB, CheB42a, CG34049, cher, TotA, Gpb5, Spn27A, Sgs3, Tk, y, beat-Va, Drs, TotM, GNBP1
4	1.75	GOTERM BP	GO:0006071 glycerol metabolic process	3	9.72E−03	Gpdh, CG18135, Gyk
5	1.58	GOTERM BP	GO:0045087 innate immune response	6	1.24E−02	Tollo, Drs, TotA, TotM, Spn27A, GNBP1
		GOTERM BP	GO:0050832 defense response to fungus	3	3.85E−02	Drs, Spn27A, GNBP1
6	1.43	SwissProt PIR	Chaperone	5	2.67E−02	CG7394, CG11267, Cnx99A, Tcp-1zeta
		GOTERM BP	GO:0006457 protein folding	6	3.49E−02	Hsc70-4, CG11267, Cnx99A, Tcp-1zeta, FKBP59
7	1.42	SwissProt PIR	Calcium	8	1.08E−02	CG4733, CG4662, pgant3, CG17271, Cad74A, sunz, Tctp
		GOTERM MF	GO:0005509 calcium ion binding	10	2.82E−02	CG4733, CG4662, pgant3, Cnx99A, CG17271, Cad74A, CG42255, sunz, Tctp
8	1.28	GOTERM BP	GO:0051606 detection of stimulus	6	1.67E−02	Or49a, Calx, CheB42a, Galphaq, FKBP59
		GOTERM BP	GO:0009628 response to abiotic stimulus	8	3.30E−02	shep, Calx, Galphaq, TotA, TotM, mth, FKBP59
9	1.21	INTERPRO	IPR007087 zinc finger, C2H2-type	13	4.40E−02	CG6689, term, CG1647, CG16779, CG10669, Meics, CG5316, CG4360, CG3065, CG11966, CG4318
		INTERPRO	IPR015880 zinc finger, C2H2-like	13	5.12E−02	CG6689, CG1647, CG16779, CG10669, Meics, CG5316, CG4360, CG3065, CG11966, noi, CG4318
10	1.2	SwissProt PIR	Mitochondrion inner membrane	5	2.45E−02	CG7394, ATPsyn-d, ATPsyn-b, Oscp, CG9603
		GOTERM BP	GO:0055085 transmembrane transport	5	6.29E−02	CG7394, ATPsyn-d, ATPsyn-b, CG32669, Oscp
11	1.16	GOTERM CC	GO:0005886 plasma membrane	20	5.15E−02	D2R, Tollo, Gr59c, Or69a, Gr85a, Cnx99A, Rac1, Cad74A, Oscp, Pk1r, Mical, Syn1, Sr-CI, scb, Or49a, Calx, Galphaq, mth, FKBP59, GNBP1
		GOTERM BP	GO:0007186 G-protein-coupled receptor protein signaling pathway	10	5.98E−02	Or49a, Tk, D2R, Gr59c, Or69a, Galphaq, Gr85a, mth, Pk1r
12	1.45	GOTERM MF	GO:0043169 cation binding	44	5.96E−02	CG6689, term, CHORD, CG9715, CG10669, CG4769, CG10916, CG5316, MICAL-like, Irk2, Cnx99A, CG17271, Cad74A, Mical, sunz, CG3065, CG11966, mud, CG4318, CG4662, CG31019, pgant3, CG33552, CG32669, Tctp, CG1647, CG16779, Meics, CG4733, x16, Mal-A2, CG4360, alph, noi, Mcm2, CD98hc, Arc1, CG42255, CG5292, nos, SF1
**C. Comparison to the *Melanogaster* subgroup***^**e**^*						
1	1.84	GOTERM BP	GO:0006071 glycerol metabolic process	3	7.90E−03	Gpdh, CG18135, Gyk
2	1.49	GOTERM BP	GO:0010605 negative regulation of macromolecule metabolic process	13	3.03E−03	WRNexo, mRpL11, Spn77Bc, Su(var)205, aub, elav, mael, Hsc70-4, PEK, E(spl)m8-HLH, fkh, lolal, nos
		GOTERM BP	GO:0032268 regulation of cellular protein metabolic process	9	3.07E−03	PEK, Adam, mRpL11, Tollo, Spn77Bc, aub, Su(var)205, nos, elav
3	1.38	INTERPRO	IPR013087 zinc finger, C2H2-type/integrase, DNA-binding	7	3.95E−02	CG11966, CG6689, CG16779, CG10669, Meics, CG4360, CG3065
		INTERPRO	IPR012934 zinc finger, AD-type	5	4.08E−02	CG6689, CG1647, CG10669, Meics, CG4318
4	1.34	GOTERM BP	GO:0007314 oocyte anterior/posterior axis specification	5	2.30E−02	mael, Tm1, lkb1, aub, nos
		GOTERM BP	GO:0007316 pole plasm RNA localization	4	2.53E−02	mael, Tm1, lkb1, aub
5	1.21	GOTERM MF	GO:0043169 cation binding	41	5.03E−02	CG6689, term, CHORD, CG9715, CG10669, CG4769, CG10916, CG5316, LpR1, Irk2, CG17271, Cad74A, Mical, CG3065, CG11966, CG4318, CG4662, CG31019, pgant3, CG33552, Tctp, Tim13, CG1647, CG16779, Meics, CG4733, x16, Mal-A2, CG4360, alph, noi, Mcm2, CG6767, CD98hc, Arc1, CG42255, CG5292, nos, SF1

BP, biological process; PIR, protein information resources; MF, molecular function; CC, cellular component.

a Categories used in DAVID analysis include GO subontologies for BP, MF, and CC, as well as key words from SwissProt PIR and protein domains from the INTERPRO database.

b Only representative terms with highly significant *P* values are shown for each annotation cluster. See Table S7, Table S8, Table S9, Table S10, Table S11, and Table S12 for complete lists.

c Count represents the number of gene families.

d Gene symbol for representative, most highly annotated member of each gene family is shown. All annotations shown here are from *D. melanogaster*.

e *D. simulans*, *D. sechellia*, *D. melanogaster*, *D. yakuba*, *and D. erecta*.

**Table 3:** GO term and functional classification enrichment analysis using DAVID for gene families that are contracted in the *Drosophila suzukii* genome as compared to other *Drosophila* species

**Annotation Cluster**	**Enrichment Score**	**Category***^**a**^*	**Term***^**b**^*	**Count***^**c**^*	***P***	**Gene Families: Representative Gene Symbols***^**d**^*
**A. Comparison to 14 *Drosophila* genomes**						
1	3.02	GOTERM CC	GO:0000786 nucleosome	5	8.14E−06	His2B, His2Av, His1, His4r, His3.3A
		SwissProt PIR	Acetylation	5	2.48E−05	His2Av, Adh, Cam, His4r, His3.3A
2	2.59	SwissProt PIR	Hydrolase	26	4.48E−06	CG1637, CG31821, S-Lap7, CG9449, Ace, CG30049, CG5731, Ance, PGRP-SA, CG14022, CG9391, ApepP, primo-2, CG14034, SPE, cathD, kraken, mag, CG2680, CG42264, CG31272, gd, LysC, Mdr49, CG6465, sda
		GOTERM MF	GO:0008233 peptidase activity	12	2.21E−03	CG31821, CG30049, ApepP, CG42264, S-Lap7, gd, SPE, CG6465, cathD, Ance, sda, PGRP-SA
3	2.54	SwissProt PIR	Disulfide bond	9	1.56E−03	crq, Adk2, gd, LysC, C1GalTA, Ace, CG5210, Ance, PGRP-SA
		SwissProt PIR	Glycoprotein	12	2.31E−03	PGRP-LE, crq, Orct2, Gr64a, pip, gd, Mdr49, C1GalTA, ninaG, Ace, CG5210, Ance
4	2.3	GOTERM MF	GO:0008238 exopeptidase activity	6	3.74E−04	CG31821, ApepP, CG42264, S-Lap7, Ance, sda
		SwissProt PIR	Carboxypeptidase	3	1.40E−02	CG31821, CG42264, Ance
5	2.24	SwissProt PIR	Aminopeptidase	3	1.62E−02	ApepP, S-Lap7, sda
6	2.03	SwissProt PIR	signal	11	4.87E−03	Acp1, Cpr47Eg, Acp53Ea, gd, LysC, ninaG, rumi, Ace, CG5210, Ance, PGRP-SA
		GOTERM CC	GO:0005576 extracellular region	12	2.79E−02	PGRP-LE, Cpr47Eg, obst-A, Acp53Ea, CG6933, gd, ninaG, CG5210, Ance, PGRP-SA, Spn27A, CG17739
7	1.83	GOTERM BP	GO:0008063 Toll signaling pathway	5	5.04E−04	pip, gd, SPE, PGRP-SA, Spn27A
8	1.55	GOTERM BP	GO:0006026 aminoglycan catabolic process	3	1.50E−02	PGRP-LE, CG5210, PGRP-SA
		GOTERM BP	GO:0006022 aminoglycan metabolic process	5	4.30E−02	PGRP-LE, obst-A, CG6933, CG5210, PGRP-SA
9	1.55	GOTERM BP	GO:0006952 defense response	7	3.29E−03	PGRP-LE, crq, Tak1, LysC, SPE, PGRP-SA, Spn27A
		GOTERM BP	GO:0006955 immune response	7	3.66E−03	PGRP-LE, crq, Tak1, LysC, SPE, PGRP-SA, Spn27A
10	1.32	SwissProt PIR	Membrane	16	3.33E−02	C1GalTA, eca, Ace, rost, Cyp6g1, crq, Orct2, Tret1-1, Gr64a, Gr93c, pip, Drip, Mdr49, ppk19, GluRIIA, Tsp42Ee
		SwissProt PIR	Transmembrane	15	3.61E−02	C1GalTA, eca, rost, crq, Orct2, Tret1-1, Gr64a, Gr93c, pip, Drip, Mdr49, ppk19, GluRIIA, Tsp42Ee, CG32053
11	1.23	SwissProt PIR	Synapse	3	4.14E−02	Snap25, Ace, GluRIIA
**B. Comparison to *D. ananassae*, *D. persimilis*, *D. pseudoobscura*, *D. willistoni*, *D. grimshawi*, *D. mojavensis*, and *D. virulis***						
1	2.61	GOTERM CC	GO:0000786 nucleosome	5	2.00E−05	His2B, His2Av, His1, His4r, His3.3A
		GOTERM BP	GO:0034728 nucleosome organization	6	2.59E−04	His2B, His2Av, His1, Nipped-A, His4r, His3.3A
		SwissProt PIR	acetylation	4	1.38E−03	His2Av, Adh, His4r, His3.3A
2	2.12	GOTERM BP	GO:0006508 proteolysis	15	1.10E−03	lwr, CG31821, S-Lap7, Roc1a, SPE, cathD, CG11864, CG30049, CG42264, CG32486, Nedd8, Prosbeta3, gd, CG6465, Ance
		GOTERM MF	GO:0008233 peptidase activity	13	3.84E−03	CG31821, ApepP, S-Lap7, SPE, cathD, CG11864, CG30049, CG42264, Prosbeta3, gd, CG6465, PGRP-SA, Ance
3	2.09	SwissProt PIR	Glycoprotein	14	1.64E−03	PGRP-LE, CG4928, C1GalTA, Ace, crq, Orct2, Gr64a, pip, gd, Mdr49, ninaG, prominin-like, CG5210, Ance
		SwissProt PIR	Membrane	18	5.62E−02	CG4928, C1GalTA, eca, Ace, rost, Cyp6g1, crq, Orct2, Tret1-1, Gr64a, Gr93c, pip, Drip, Mdr49, prominin-like, ppk19, GluRIIA, Tsp42Ee
4	1.89	SwissProt PIR	Signal	12	8.54E−03	Acp1, Cpr47Eg, Amyrel, gd, LysC, ninaG, rumi, Dpt, Ace, CG5210, Ance, PGRP-SA
		GOTERM CC	GO:0005576 extracellular region	13	5.70E−02	PGRP-LE, Cpr47Eg, Amyrel, CG17575, Dpt, obst-A, gd, ninaG, CG5210, Muc26B, PGRP-SA, Ance, CG17739
5	1.76	GOTERM MF	GO:0008238 exopeptidase activity	5	7.22E−03	CG31821, ApepP, CG42264, S-Lap7, Ance
		SwissProt PIR	carboxypeptidase	3	2.15E−02	CG31821, CG42264, Ance
6	1.49	GOTERM BP	GO:0006955 immune response	8	2.87E−03	lwr, PGRP-LE, crq, Tak1, LysC, SPE, Dpt, PGRP-SA
		GOTERM BP	GO:0042742 defense response to bacterium	5	8.54E−03	PGRP-LE, LysC, SPE, Dpt, PGRP-SA
7	1.36	GOTERM BP	GO:0006026 aminoglycan catabolic process	3	2.39E−02	PGRP-LE, CG5210, PGRP-SA
		GOTERM BP	GO:0009057 macromolecule catabolic process	8	5.95E−02	lwr, PGRP-LE, Prosbeta3, Nedd8, CG32486, Roc1a, CG5210, PGRP-SA
**C. Comparison to the *Melanogaster* subgroup***^**e**^*						
1	2.76	GOTERM BP	GO:0008063 Toll signaling pathway	6	9.04E−05	IM10, pip, gd, SPE, PGRP-SA, Spn27A
		GOTERM BP	GO:0006952 defense response	9	4.43E−04	PGRP-LE, crq, IM10, Tak1, LysC, SPE, IM2, PGRP-SA, Spn27A
2	2.73	GOTERM CC	GO:0000786 nucleosome	5	1.78E−05	His2B, His2Av, His1, His4r, His3.3A
		SwissProt PIR	Acetylation	5	6.19E−05	His2Av, Adh, Cam, His4r, His3.3A
3	2.61	SwissProt PIR	Signal	14	1.07E−03	Acp1, Cpr47Eg, IM10, Acp53Ea, Ace, gd, LysC, rumi, ninaG, IM2, CG5210, PGRP-SA, mth, Ance
		GOTERM CC	GO:0005576 extracellular region	15	9.25E−03	PGRP-LE, Cpr47Eg, IM10, CheB42a, Acp53Ea, Spn27A, CG6933, gd, ninaG, IM2, CG5210, Muc26B, PGRP-SA, Ance, CG17739
4	2.45	GOTERM CC	Disulfide bond	11	4.43E−04	crq, Adk2, gd, LysC, C1GalTA, IM2, Ace, CG5210, mth, Ance, PGRP-SA
		GOTERM CC	Glycoprotein	14	1.74E−03	PGRP-LE, IM10, C1GalTA, Ace, crq, Orct2, Gr64a, pip, gd, Mdr49, ninaG, CG5210, mth, Ance
5	2.23	GOTERM MF	GO:0008238 exopeptidase activity	6	7.73E−04	CG31821, ApepP, CG42264, S-Lap7, Ance, sda
		GOTERM MF	GO:0008233 peptidase activity	13	2.58E−03	CG31821, ApepP, S-Lap7, SPE, CG42370, cathD, CG30049, CG42264, gd, CG6465, PGRP-SA, sda, Ance
6	2.09	SwissProt PIR	carboxypeptidase	3	2.18E−02	CG31821, CG42264, Ance
7	2.03	SwissProt PIR	Aminopeptidase	3	2.51E−02	ApepP, S-Lap7, sda
8	1.89	SwissProt PIR	Transmembrane	22	1.60E−03	CG13796, Or65a, C1GalTA, eca, Gr22f, rost, crq, Orct2, Tret1-1, Gr64a, Gr93c, pip, CG32301, Drip, Mdr49, sesB, mth, ppk19, CG7255, CG32053, Tsp42Ee, GluRIIA
		SwissProt PIR	Membrane	21	8.80E−03	Or65a, C1GalTA, Gr22f, eca, Ace, rost, Cyp6g1, crq, Orct2, Tret1-1, Gr64a, Gr93c, pip, CG32301, Drip, Mdr49, sesB, mth, ppk19, Tsp42Ee, GluRIIA
9	1.43	GOTERM BP	GO:0006026 aminoglycan catabolic process	3	2.29E−02	PGRP-LE, CG5210, PGRP-SA
10	1.41	GOTERM BP	GO:0008219∼cell death	8	9.69E−03	eIF-4E, Cyt-c-d, crq, qkr58E-3, Eig71Ej, Tak1, LysC, cathD
11	1.39	GOTERM BP	GO:0045087 innate immune response	7	5.86E−04	PGRP-LE, IM10, Tak1, SPE, IM2, PGRP-SA, Spn27A
		GOTERM BP	GO:0002786 regulation of antibacterial peptide production	3	3.96E−02	Tak1, SPE, PGRP-SA

CC, cellular component; PIR, protein information resource; MF, molecular function; BP, biological process.

a Categories used in DAVID analysis include GO subontologies for BP, MF, and CC, as well as keywords from SwissProt PIR and protein domains from the INTERPRO database.

b Only representative terms with highly significant *P* values are shown for each annotation cluster. See Table S7, Table S8, Table S9, Table S10, Table S11, and Table S12 for complete lists.

c Count represents the number of gene families.

d Gene symbol for representative, most highly annotated member of each gene family is shown. All annotations shown here are from *D. melanogaster*.

e *D. simulans*, *D. sechellia*, *D. melanogaster*, *D. yakuba*, *and D. erecta*.

The most enriched categories (function or protein domains) representing gene families that are specifically expanded in *D. suzukii* (and most likely in *D. biarmipes* and *D. takahashii*), *i.e.*, significantly enriched in all three comparisons, include “glycerol metabolic process,” “regulation of cellular protein metabolic processes,” and “zinc finger proteins” (Table 2). Other notable functional annotations that are enriched in gene families expanded in *D. suzukii*, especially when compared to more basal *Drosophila* species, include “detection of stimulus,” “sensory transduction,” “G-protein-coupled receptor protein signaling,” “proton-transporting ATP synthase activity,” and “endopeptidase inhibitor.” These categories are not significantly enriched in expanded *D. suzukii* gene families as compared to species in the *melanogaster* subgroup, suggesting that expansion of these gene families predates the divergence of *D. suzukii* and the *melanogaster* subgroup. Future functional characterization will be necessary to determine if expansion in the specific gene families involved in taste and smell perception plays a role in host plant and feeding preference ( Matsuo *et al.* 2007). “Defense response” and “immune response” are two functional categories that were also significantly enriched in expanded *D. suzukii* gene families only when they were compared to more basal *Drosophila* species (Table 2B). Most of these expanded gene families encode either recognition proteins, *e.g.*, scavenger receptors and Gram-negative binding proteins, or effector molecules, *e.g.*, turandot humoral factors and drosomycin, as opposed to signal transducers. This phenomenon was also found in a detailed study of immune response gene evolution (Sackton *et al.* 2007).

The most enriched functional categories representing gene families that contracted in *D. suzukii* were quite uniform in all our comparisons and did not differ significantly when gene counts in *D. suzukii* gene families were compared to those in all *Drosophila* species or a subset (Table 3, Table S10, Table S11, and Table S12), indicating that these gene family contractions are likely restricted to *D. suzukii* or a very closely related species. Enriched annotations include “nucleosome,” “peptidase activity,” “glycoprotein,” “membrane and transmembrane protein,” “immune response,” and “defense response.” It is curious that “immune response” and “defense response” are enriched in both expanded and contracted gene families in *D. suzukii*. A closer inspection revealed that gene sets that are expanded or contracted do not overlap and represent proteins with different molecular functions (Table 2 and Table 3). Interestingly, as in the case of gene family expansion, immune and defense-related gene families that contracted in *D. suzukii* also represent recognition proteins, *e.g.*, specific peptidoglycan recognition proteins and croquemort scavenger receptors, or effector molecules, *e.g.*, immune-induced molecule. Because *D. suzukii* feed on fresh fruits as opposed to rotting fruits and decaying matter, the microorganisms it encounters may be different from those encountered by most fruit-associated *Drosophila*; this ecological difference may be the driving force in altering the repertoire of defense systems.

Other notable classes of gene families that contracted in *D. suzukii* and are represented under multiple enriched annotation clusters include those that are involved in detoxification of endogenous and xenobiotic substances, *e.g.*, esterases and cytochrome P450 (Cyp) (Table S13). *D. suzukii* types 3 and 4 Cyps are reduced in numbers relative to most other *Drosophila* species included in this analysis, except in the closely related *D. biarmipes* and *D. takahashii*. The type 3 Cyp contains many P450s that are involved in detoxification of xenobiotics and endobiotics (Baldwin *et al.* 2009), including families that are known to confer insecticide resistance when upregulated, such as Cyp4, Cyp6, and Cyp9 (Li *et al.* 2007). The Cyp4 clade is less well-studied in insects but is thought to be involved in fatty acid metabolism (Feyereisen 2005; Baldwin *et al.* 2009). The gene count reduction observed in *D. suzukii* may be a reduction in the Cyp3, Cyp4, or both clades. Because cytochrome P450s are considered to be the only metabolic system in insects that can mediate resistance to all classes of insecticides (Feyereisen 2005; Li *et al.* 2007), it will be interesting and of applied importance to examine the consequences of this gene reduction with respect to insecticide tolerance. In addition to a reduction in type 3 and 4 Cyps, *D. suzukii* and the closely related *D. biarmipes* and *D. takahashii* have reduced numbers of glutathione-S-transferases (GSTs), which are also involved in detoxification processes (Table S13), although GSTs were not classified into one of the enriched annotation categories.

### Origination of novel retrogenes

Retroposed genes contribute to new gene evolution (Long *et al.* 2003) and may often evolve adaptively (Long and Langley 1993). In *Drosophila*, the origination rate is approximately 0.5 gene/myr (Bai *et al.* 2007). We found seven lineage-specific new retrogenes in the *D. suzukii* genome (Table 4), all of which have a complete ORF. Three of the new retrogenes originated on *X* chromosome, whereas the other four new retrogenes originated from Muller elements E (homologous to *3R* of *D. melanogaster*). The parental genes are *RpS14a*, *Sce*, *T-cp1*, *βTub97EF* (with two new copies), *RpL36*, and *VhaAC39*. All of the new copies are located on autosomes, which is consistent with the previous research on retrogenes indicating an “off-the-X” bias (Betran *et al.* 2002).

**Table 4:** Lineage-specific novel retrogenes in *Drosophila suzukii*

**Gene Symbol**	**Parental Gene**	**Parental Scaffold**	**Parental Location**	**New Retrogene**	**New Scaffold**	**New Location**
RpS14a	DS10_00007035	Scaffold10	X	DS10_00010264	Scaffold238	2R
Sce	DS10_00011581	Scaffold309	3R	DS10_00013241	Scaffold1447	3R
T-cp1	DS10_00011776	Scaffold294	3R	DS10_00010739	Scaffold133	3L
betaTub97EF	DS10_00007439	Scaffold39	3R	DS10_00007439_dup1	Scaffold2	2R
betaTub97EF	DS10_00007439	Scaffold39	3R	DS10_00007439_dup2	Scaffold433	3R
RpL36	DS10_00006960	Scaffold10	X	DS10_00012180	Scaffold334	2R
VhaAC39	DS10_00006116	Scaffold7	X	DS10_00010518	Scaffold182	3R

### Identified transposable element sequence

TEs represent 4.9% of contig sequences for all scaffolds more than 5 kb in length when identified at 50% similarity to known *D. melanogaster* TEs (Table S14). We found that 46.8% of the total base pairs identified as TEs appear to belong to DNA elements, with the rest belonging to RNA elements. Although this is a greater proportion than the amount of sequence belonging to DNA elements in *D. melanogaster*, (17%), it is difficult to directly compare these results. First, different TE elements can be of quite different lengths and TE abundance is usually calculated by number of insertions. We calculated bp of sequence because elements were only identified computationally. Second, it can be difficult to properly identify TEs based on short sequences because of sequence similarities between many different element types.

**Figure 5:**
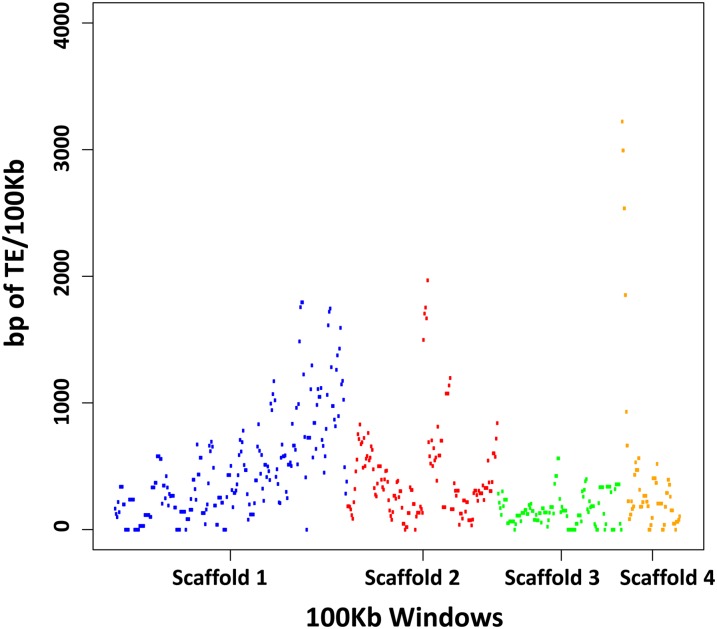
Distribution of transposable elements in scaffolds ≥5 Mb. The total number of base pairs identified as belonging to TE sequence per 100-kb window. There is an increase in TE density toward the end of scaffold 1 as well as a peak in scaffold 2 and scaffold 4.

We also identified regions of increased TE density (Figure 5) using the 50% identity information. In *D. melanogaster*, most TEs are found near the ends of chromosomes and near the centromere. We noticed increased TE densities at one end of scaffold 1 and one end of scaffold 4, as well as a spike in TE density near the middle of scaffold 2.

## Conclusion

To enable and accelerate basic and applied research on *D. suzukii*, a new invasive pest with rapidly expanding range as well as interesting biological adaptations with respect to anatomy and feeding preferences, we sequenced its genome to high coverage and performed a comparative genomic analysis of *D. suzukii* with other species in the *Sophophora* and *Drosophila* subgenera. More importantly, we have created a web portal, SpottedWingFlyBase, to facilitate public access to our data analyses and annotation of the *D. suzukii* genome. In addition to the many aspects of insect biology that can be elucidated using functional genomics, the value of the *D. suzukii* genome can be extended to the improvement of applied research and pest management efforts. Development of RNAi-based pest control (Grimmelikhuijzen and Hauser 2012), new insecticide targets (Grimmelikhuijzen *et al.* 2007; Grimmelikhuijzen and Hauser 2012), markers for insecticide resistance (Li *et al.* 2007), more effective pheromone-based attractants (Tittiger 2004), as well as more targeted arthropod or microbial biological control agents may benefit from *D. suzukii* genomic resources.

## Supplementary Material

Supporting Information
