# Supplementary material for: Genome of Drosophila suzukii, the Spotted Wing Drosophila
Source: G3 (Bethesda). 2013 Oct 18;3(12):2257–71. doi: 10.1534/g3.113.008185 (PMC3852387; doi:10.1534/g3.113.008185)
Supplement: Supporting Information [file supp_3_12_2257__index.html]

Genome of Drosophila suzukii, the Spotted Wing Drosophila — Supporting Information 

# Genome of *Drosophila suzukii*, the Spotted Wing *Drosophila*

## Supporting Information for Chiu *et al.*, 2013

**Files in this Data Supplement:**

- Supporting Information - Figures S1-S4 and Tables S1-S14 (PDF, 1 MB)
- Figure S1 - 17-kmer estimation of genome size. (PDF, 412 KB)
- Figure S2 - Synteny map between *D. suzukii* and *D. melanogaster* genomes. (PDF, 510 KB)
- Figure S3 - Best-scoring maximum likelihood (ML) tree of 15 *Drosophila* species with outgroup *A. gambiae* (not shown) using 5,322 gene partitions with 5,199,249 sites. (PDF, 476 KB)
- Figure S4 - (A) GC3 content for *D. suzukii* X and autosomal positions. (B) GC3 content for *Drosophila suzukii* male biased genes (left) and the overall *D. suzukii* GC3 content. (PDF, 447 KB)
- Table S1 - Data production for *Drosophila suzukii* genome sequencing. (PDF, 516 KB)
- Table S2 - *Drosophila suzukii* 17-k-mer statistics. (PDF, 516 KB)
- Table S3 - Statistics of the assembled genome. (PDF, 408 KB)
- Table S4 - A list of 25 genes in *Drosophila suzukii* with the highest non-synonymous (dN) substitution rates. (PDF, 522 KB)
- Table S5 - A list of *Drosophila suzukii* genes with sex-biased expression pattern. (PDF, 756 KB)
- Table S6 - A list of genes showing sex-biased shift in expression between *Drosophila suzukii* and *Drosophila melanogaster*. (PDF, 527 KB)
- Table S7 - GO term and functional classification enrichment analysis using DAVID for gene families that are expanded in the *Drosophila suzukii* genome as compared to 14 *Drosophila* species analyzed in this study. (PDF, 536 KB)
- Table S8 - GO term and functional classification enrichment analysis using DAVID for gene families that are expanded in the *Drosophila suzukii* genome as compared to the basal paraphyletic group of Drosophilidae including *D. ananassae, D. persimilis, D. pseudoobscura, D. willistoni, D. grimshawi, D. mojavensis*, and *D. virulis*. (PDF, 542 KB)
- Table S9 - GO term and functional classification enrichment analysis using DAVID for gene families that are expanded in the *Drosophila suzukii* genome as compared to species in the melanogaster subgroup, including *D. simulans, D. sechellia, D. yakuba, D. erecta*, and *D. melanogaster*. (PDF, 532 KB)
- Table S10 - GO term and functional classification enrichment analysis using DAVID for gene families that are contracted in the *Drosophila suzukii* genome as compared to 14 *Drosophila* species analyzed in this study. (PDF, 542 KB)
- Table S11 - GO term and functional classification enrichment analysis using DAVID for gene families that are contracted in the *Drosophila suzukii* genome as compared to the basal paraphyletic group of Drosophilidae including *D. ananassae, D. persimilis, D. pseudoobscura, D. willistoni, D. grimshawi, D. mojavensis*, and *D. virulis*. (PDF, 538 KB)
- Table S12 - GO term and functional classification enrichment analysis using DAVID for gene families that are contracted in the *Drosophila suzukii* genome as compared to species in the melanogaster subgroup, including *D. simulans, D. sechellia, D. yakuba, D. erecta*, and *D. melanogaster*. (PDF, 544 KB)
- Table S13 - Gene families of enzymes involved in metabolism and transport of xenobiotics. (PDF, 411 KB)
- Table S14 - Total base pairs of transposable element families. (PDF, 439 KB)
