# Supplementary material for: Genome of Drosophila suzukii, the Spotted Wing Drosophila
Source: G3 (Bethesda). 2013 Oct 18;3(12):2257–71. doi: 10.1534/g3.113.008185 (PMC3852387; doi:10.1534/g3.113.008185)
Supplement: Supporting Information [file supp_g3.113.008185_008185SI.pdf]

G3 · Genes | Genomes | Genetics

Genome of *Drosophila suzukii*, the Spotted Wing Drosophila

Joanna C. Chiu*, Xuanting Jiang§, Li Zhao†, Christopher A. Hamm†, Julie M. Cridland†, Perot Saelao†, Kelly A. Hamby*, Ernest K. Lee‡, Rosanna S. Kwok*, Guojie Zhang§, Frank G. Zalom*, Vaughn M. Walton¶, David J. Begun†

*Department of Entomology and Nematology, College of Agricultural and Environmental Sciences, University of California, Davis, CA 95616, USA.

§China National Genebank, BGI-Shenzhen, 518083, China.

†Department of Evolution and Ecology, College of Biological Sciences, University of California, Davis, CA 95616, USA.

‡Sackler Institute for Comparative Genomics, American Museum of Natural History, New York, NY 10024, USA.

¶Department of Horticulture, Oregon State University, Corvallis, OR 97331, USA.

Corresponding author: Joanna C. Chiu, University of California, Department of Entomology and Nematology, Davis, CA 95616. Email: jcchiu@ucdavis.edu.

DOI: 10.1534/g3.113.008185

Table S1 Data production for *Drosophila suzukii* genome sequencing.

INSERT SIZE (BP)	TOTAL DATA (GB)	READ LENGTH	SEQUENCE COVERAGE*	PHYSICAL COVERAGE
250	9.35	150	42.49	37.94
300	4.50	146	20.47	36.26
500	6.64	90	30.19	88.79
2k	5.99	49	27.22	555.55
9k	0.94	160	4.27	284.35
10k	9.58	49	43.55	4443.37
20k	14.45	49	65.67	13402.20

*Genome size was estimated at 220Mbp

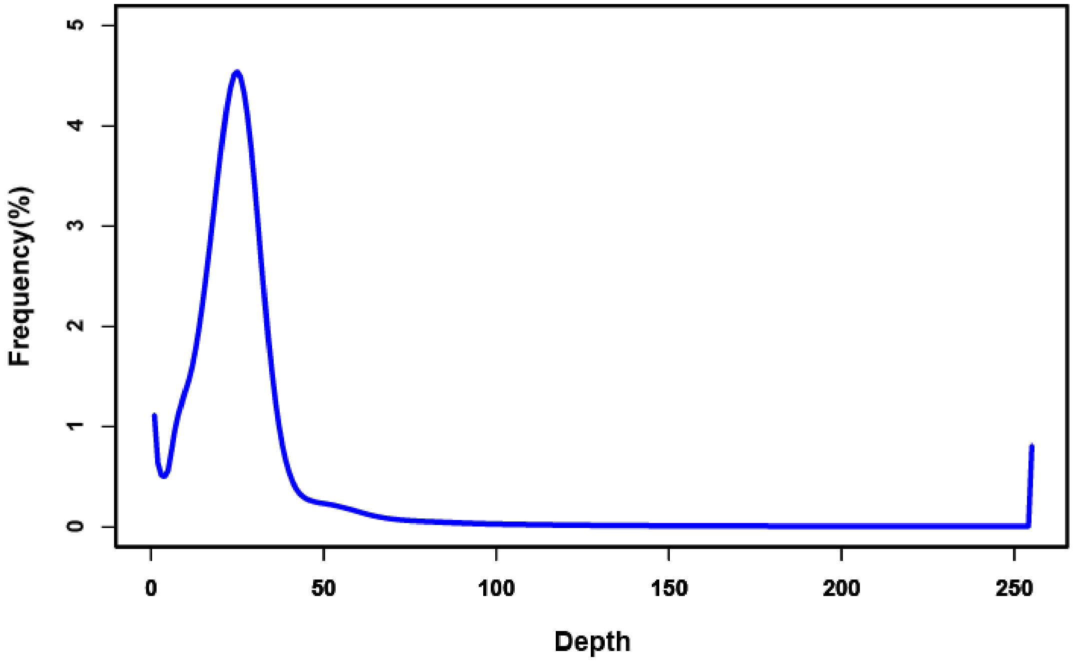

Figure S1 17-kmer estimation of genome size. The genome size of *Drosophila suzukii* was estimated to be 220Mb based on reads from short insert size libraries.

Table S2 ***Drosophila suzukii*** **17-k-mer statistics.**

SPECIES	K-MER	K-MER NUM	K-MER DEPTH	GENOME SIZE (BP)	SEQUENCE COVERAGE
D. suzukii	17	5,515,021,508	25	220,600,860	30.07

Table S3   Statistics of the assembled genome.

	CONTIG		SCAFFOLD	
	LENGTH (BP)	NUMBER	LENGTH (BP)	NUMBER
N90	3,736	10,573	39,620	812
N80	7,638	6,817	102,455	446
N70	11,943	4,678	163,657	267
N60	16,903	3,230	254,714	151
N50	23,216	2,194	385,236	73
Total Size	204,921,988		235,559,420	
Longest	472,152		22,559,587	
Total Number (>100bp)		46,329		29,113
Total Number (>2kb)		14,013		2,723

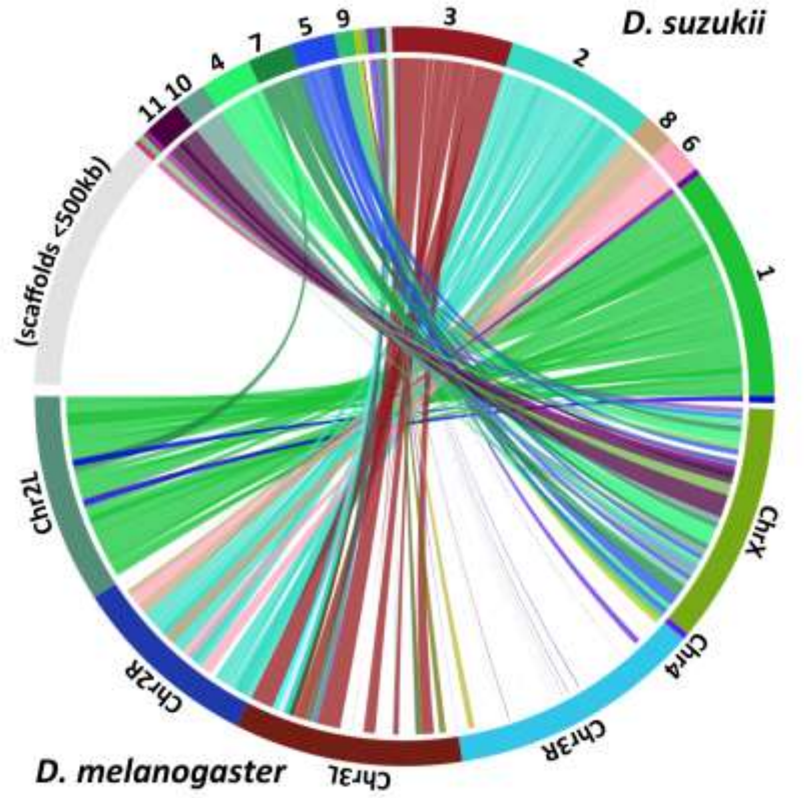

Figure S2 Synteny map between *D. suzukii* and *D. melanogaster* genomes. Only scaffolds >500kb in sizes are used in the synteny analysis. Scaffolds >2Mb in sizes are labeled.

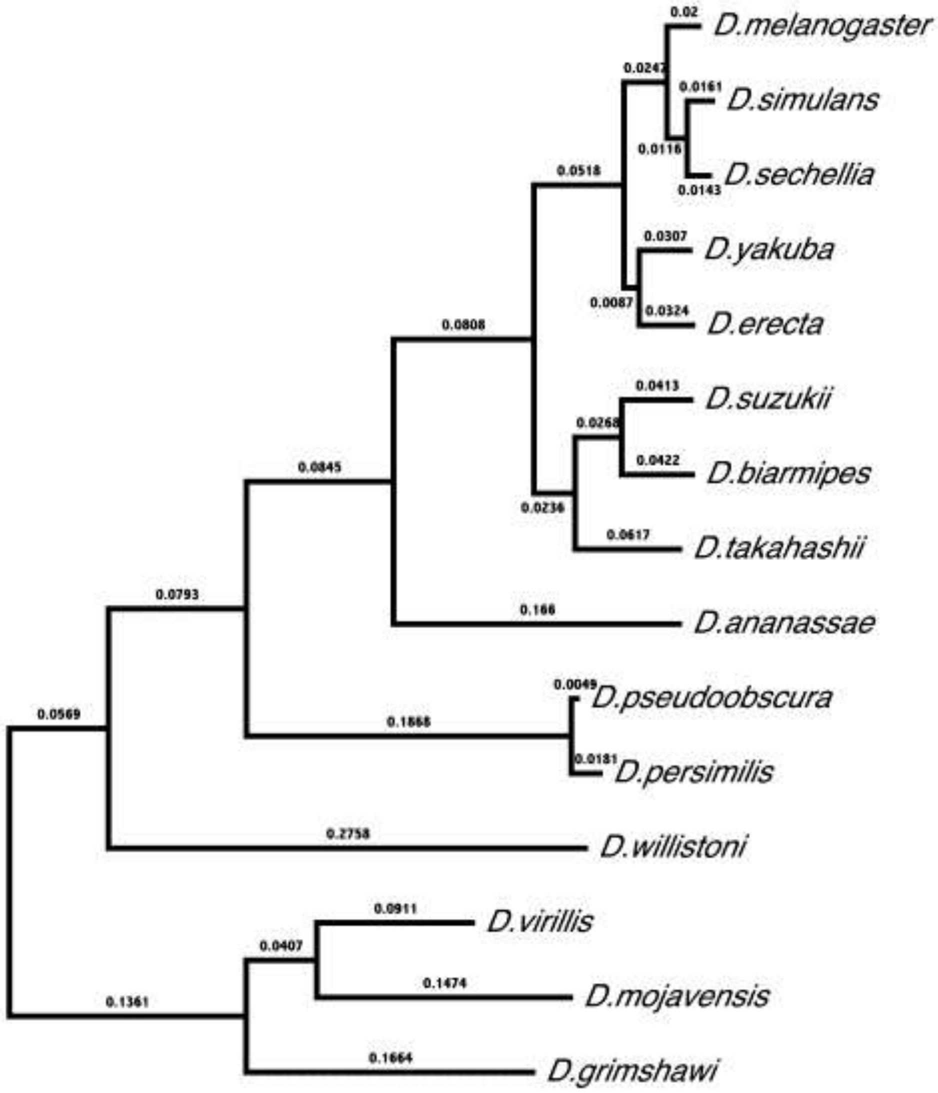

Figure S3 Best-scoring maximum likelihood (ML) tree of 15 *Drosophila* species with outgroup *A. gambiae* (not shown) using 5,322 gene partitions with 5,199,249 sites. Best model was selected individually for each partition. Bootstrap support values from 250 bootstrap replicates are 100 for all nodes. Branch labels indicate the mean number of substitutions per site.

A B

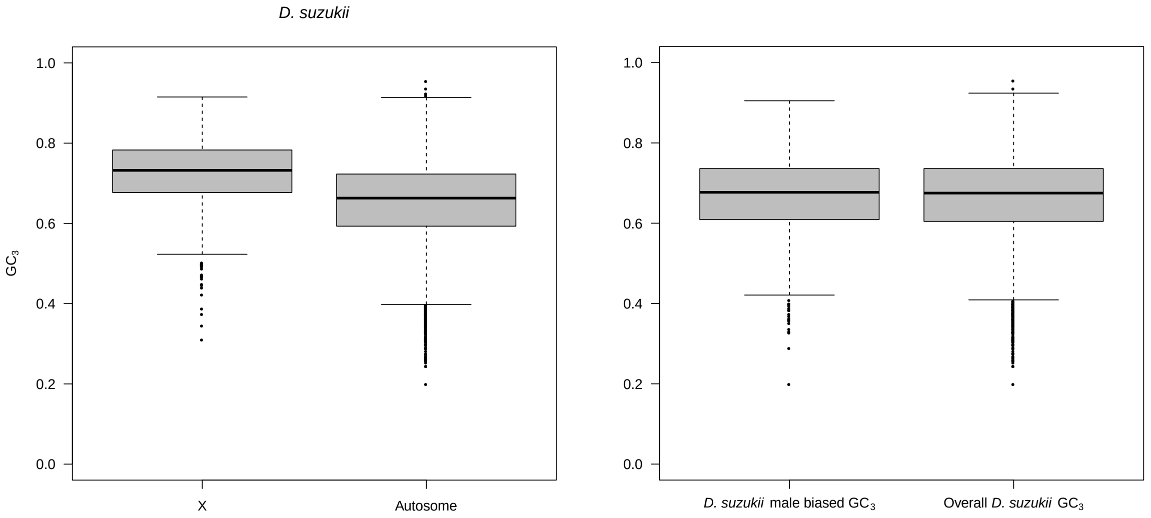

Figure S4 (A) GC_3 content for *D. suzukii* X and autosomal positions. The two data sets were significantly different (Mann-Whitney U test, p = < $2.2e^{-16}$). (B) GC_3 content for *Drosophila suzukii* male biased genes (left) and the overall *D. suzukii* GC_3 content. The two data sets were not significantly different (Mann-Whitney U test, p = 0.706).

Table S4 A list of 25 genes in *Drosophila suzukii* with the highest non-synonymous (dN) substitution rates.

FLYBASE_ID OF *D. MEL* ORTHOLOG	LOCATION OF *D. MEL* ORTHOLOG	GENE NAME OF *D. MEL* ORTHOLOG	MOLECULAR FUNCTION
FBpp0300230	X	CG43386	function unknown
FBpp0292981	2L	CG42848	function unknown
FBpp0292728	3R	CG34006-PB	function unknown
FBpp0077038	X	CG14619-PE	ubiquitin-specific protease activity
FBpp0083206	3R	branchless-PA	fibroblast growth factor receptor binding
FBpp0075387	3L	CTPsyn-PC	CTP synthase activity
FBpp0075087	3L	nudC-PA	nuclear migration
FBpp0289636	3R	CG34034-PB	function unknown
FBpp0080350	2L	CG13244-PA	function unknown
FBpp0076948	X	CG14579-PA	function unknown
FBpp0080236	2L	CG33309-PA	function unknown
FBpp0293396	2L	bruno-2-PE	RNA/mRNA binding
FBpp0087089	2R	CG13186-PA	function unknown
FBpp0099816	4	Dyrk3-PA	protein serine/threonine kinase activity
FBpp0297068	3L	CG43168-PA	function unknown
FBpp0082217	3R	CheA87a-PA	function unknown
FBpp0075635	3L	CG10752-PA	function unknown
FBpp0111417	3R	CG34308-PA	zinc ion binding
FBpp0289540	2R	Sfp53D-PA	multicellular organism reproduction
FBpp0073917	X	CG8565-PA	protein kinase activity
FBpp0075636	3L	CG32115-PA	function unknown
FBpp0073239	X	CG15208-PA	function unknown (sperm competition)
FBpp0110475	2L	CG17490-PA	function unknown
FBpp0081175	3R	CG15185-PA	function unknown
FBpp0083140	3R	CG14294-PA	function unknown

Table S5 A list of *Drosophila suzukii* genes with sex-biased expression pattern.

GENE	GENE SYMBOL	LOCUS	FEMALE EXPRESSION	MALE EXPRESSION	SEX-BIAS
DS10_00000010		scaffold1:50412-58902	0.95	6.56	male
DS10_00000019		scaffold1:138485-171077	0.03	26.59	male
DS10_00000024		scaffold1:208563-211507	0.18	67.75	male
DS10_00000034		scaffold1:254095-298173	0.02	67.56	male
DS10_00000041		scaffold1:360867-361911	5.54	0.84	female
DS10_00000058	rempA	scaffold1:515827-521458	0.46	3.54	male
DS10_00000074	Hsp60B	scaffold1:697183-699130	0.02	108.25	male
DS10_00000078		scaffold1:761457-763179	2.10	15.50	male
DS10_00000099		scaffold1:871629-881205	0.03	5.99	male
DS10_00000105		scaffold1:1102514-1103180	0.00	79.89	male
DS10_00000122	fred	scaffold1:1473153-1521996	0.51	12.23	male
DS10_00000126	Cep97	scaffold1:1572523-1589908	3.72	29.93	male
DS10_00000144	cutlet	scaffold1:1765690-1770175	2.29	0.16	female
DS10_00000165		scaffold1:1986921-1987956	0.00	31.00	male
DS10_00000167		scaffold1:2014864-2015641	0.05	352.66	male
DS10_00000169	CSN1a	scaffold1:2018789-2019899	0.00	31.63	male
DS10_00000170		scaffold1:2022179-2023328	0.00	34.89	male
DS10_00000174		scaffold1:2063231-2066173	0.00	53.04	male
DS10_00000175		scaffold1:2067012-2069688	0.00	21.41	male
DS10_00000179	Mst35Ba	scaffold1:2148730-2149477	0.00	195.63	male
DS10_00000182		scaffold1:2198872-2205568	0.01	6.42	male
DS10_00000211	yuri	scaffold1:2569244-2576794	10.59	41.74	male
DS10_00000212	Cul-3	scaffold1:2579423-2584418	13.58	67.34	male
DS10_00000215		scaffold1:2602592-2608625	0.00	44.94	male
DS10_00000217		scaffold1:2615357-2616662	0.03	9.53	male
DS10_00000220		scaffold1:2690357-2690957	0.00	33.39	male
DS10_00000222		scaffold1:2773177-2774635	0.00	37.67	male
DS10_00000224	Tim17b2	scaffold1:2823377-2823911	0.00	76.72	male
DS10_00000238		scaffold1:3079851-3086635	0.01	2.41	male
DS10_00000254		scaffold1:3267469-3268920	0.65	24.52	male
DS10_00000255		scaffold1:3269899-3272212	0.79	46.01	male
DS10_00000266		scaffold1:3440769-3441051	41.70	0.64	female
DS10_00000269		scaffold1:3445463-3448095	2.52	36.37	male
DS10_00000273	SP555	scaffold1:3457514-3459842	2.32	21.30	male
DS10_00000294		scaffold1:3560513-3571213	0.02	18.79	male
DS10_00000296		scaffold1:3605345-3606320	0.00	44.96	male
DS10_00000304		scaffold1:3722034-3725787	0.16	4.83	male
DS10_00000307		scaffold1:3740175-3749073	0.03	4.94	male
DS10_00000309		scaffold1:3779516-3780587	0.16	31.41	male
DS10_00000318		scaffold1:3876299-3877209	0.58	6.62	male
DS10_00000319		scaffold1:3886337-3886889	0.10	64.44	male
DS10_00000320	robl22E	scaffold1:3894132-3899177	29.35	3.36	female
DS10_00000322		scaffold1:3902887-3903463	0.00	73.76	male
DS10_00000326		scaffold1:3931791-3932414	0.00	69.84	male
DS10_00000328		scaffold1:3946326-3952310	3.07	0.05	female
DS10_00000330	Tengl2	scaffold1:3966381-3969940	0.00	25.88	male
DS10_00000347	d	scaffold1:4098412-4112931	0.30	2.93	male
DS10_00000359		scaffold1:4188097-4189399	0.22	46.49	male
DS10_00000361		scaffold1:4192145-4197064	0.04	52.21	male
DS10_00000390	Spn53F	scaffold1:4313749-4318843	0.02	71.31	male
DS10_00000393	Spn88Ea	scaffold1:4381235-4384495	0.00	140.71	male

DS10_00000394		scaffold1:4385278-4408009	6.21	54.38	male
DS10_00000397		scaffold1:4426946-4427808	0.33	42.64	male
DS10_00000430		scaffold1:4560916-4565048	0.01	19.69	male
DS10_00000431		scaffold1:4576587-4578798	0.00	9.38	male
DS10_00000433	Cpr31A	scaffold1:4607767-4609822	0.07	2.38	male
DS10_00000434	Pen	scaffold1:4611336-4614571	46.77	452.30	male
DS10_00000441	Sur	scaffold1:4756455-4777707	1.48	7.07	male
DS10_00000454	me31B	scaffold1:4827568-4830580	163.78	44.71	female
DS10_00000479		scaffold1:5150721-5151760	0.10	36.62	male
DS10_00000481		scaffold1:5161289-5161816	0.00	102.11	male
DS10_00000483		scaffold1:5232015-5234980	0.02	30.76	male
DS10_00000490		scaffold1:5326075-5326591	0.28	7.90	male
DS10_00000491		scaffold1:5327006-5327508	0.58	10.16	male
DS10_00000512		scaffold1:5462863-5464890	0.72	32.82	male
DS10_00000521		scaffold1:5539688-5540267	0.15	65.05	male
DS10_00000533		scaffold1:5668931-5669615	0.00	52.07	male
DS10_00000541		scaffold1:5854926-5876637	1.24	7.14	male
DS10_00000576	Ts	scaffold1:6127436-6128402	16.39	2.45	female
DS10_00000586		scaffold1:6242755-6249255	0.55	131.66	male
DS10_00000590		scaffold1:6276938-6289732	0.39	6.08	male
DS10_00000596		scaffold1:6327789-6329442	0.00	34.78	male
DS10_00000600		scaffold1:6367817-6368950	0.74	4.98	male
DS10_00000611		scaffold1:6398665-6400298	0.21	8.39	male
DS10_00000618		scaffold1:6494012-6495069	0.04	22.05	male
DS10_00000619	Prosbeta4R2	scaffold1:6503497-6504261	0.00	39.13	male
DS10_00000623		scaffold1:6513863-6518058	0.00	19.66	male
DS10_00000636		scaffold1:6620414-6629074	326.70	67.07	female
DS10_00000644		scaffold1:6692924-6694037	0.04	13.10	male
DS10_00000662		scaffold1:6875129-6881940	6.03	37.22	male
DS10_00000666		scaffold1:6924216-6928845	0.00	32.00	male
DS10_00000669		scaffold1:6964797-6974972	0.01	45.62	male
DS10_00000689		scaffold1:7206514-7207146	0.11	313.63	male
DS10_00000690		scaffold1:7211637-7212237	0.11	214.16	male
DS10_00000703		scaffold1:7279997-7281056	0.00	26.81	male
DS10_00000706		scaffold1:7326855-7328232	0.27	29.76	male
DS10_00000711		scaffold1:7414311-7420219	0.19	5.51	male
DS10_00000714	Iris	scaffold1:7442050-7444144	17.88	0.12	female
DS10_00000731		scaffold1:7524992-7525553	0.00	73.44	male
DS10_00000737		scaffold1:7678680-7679566	0.00	28.80	male
DS10_00000739	ctp	scaffold1:7690085-7690355	0.51	61.81	male
DS10_00000740		scaffold1:7698220-7702443	0.00	31.86	male
DS10_00000742		scaffold1:7718971-7719909	0.05	20.21	male
DS10_00000755		scaffold1:7970935-7973109	0.00	107.67	male
DS10_00000771		scaffold1:8206137-8206524	0.15	28.68	male
DS10_00000776		scaffold1:8284525-8286547	0.00	10.72	male
DS10_00000778	c-cup	scaffold1:8299122-8299783	0.00	32.55	male
DS10_00000781		scaffold1:8331692-8356503	0.16	89.58	male
DS10_00000786		scaffold1:8435102-8435919	0.08	7.76	male
DS10_00000787		scaffold1:8435967-8437123	0.08	5.73	male
DS10_00000795	Obp22a	scaffold1:8495826-8496246	0.00	225.51	male
DS10_00000805		scaffold1:8621325-8622156	0.00	52.20	male
DS10_00000807		scaffold1:8633007-8633674	0.00	73.13	male
DS10_00000810		scaffold1:8651876-8657774	0.87	13.31	male
DS10_00000822		scaffold1:8745371-8746992	0.00	28.47	male

DS10_00000832		scaffold1:8829866-8830802	0.11	10.52	male
DS10_00000835		scaffold1:8836938-8837589	0.00	198.78	male
DS10_00000836	Sfp38D	scaffold1:8838750-8840272	0.00	113.15	male
DS10_00000838		scaffold1:8864471-8865227	0.11	43.34	male
DS10_00000841		scaffold1:8940690-8941319	0.18	60.10	male
DS10_00000845		scaffold1:9020037-9020408	0.25	40.43	male
DS10_00000873		scaffold1:9449321-9449744	0.63	14.92	male
DS10_00000876	ms(2)34Fe	scaffold1:9504184-9509762	0.09	29.23	male
DS10_00000888		scaffold1:9608095-9608759	0.00	38.01	male
DS10_00000893		scaffold1:9754813-9755793	0.05	111.14	male
DS10_00000894	lwr	scaffold1:9772060-9772516	0.49	35.85	male
DS10_00000909		scaffold1:9949655-9955048	0.03	266.58	male
DS10_00000912	Skadu	scaffold1:10012026-10012794	0.00	35.60	male
DS10_00000913		scaffold1:10015591-10017479	0.03	36.26	male
DS10_00000916		scaffold1:10078900-10080540	0.00	37.41	male
DS10_00000926	CycE	scaffold1:10162392-10174917	6.56	1.12	female
DS10_00000936	Tektin-A	scaffold1:10294199-10298428	0.00	78.92	male
DS10_00000938		scaffold1:10323809-10326346	0.00	14.13	male
DS10_00000940		scaffold1:10341650-10343710	0.00	36.49	male
DS10_00000943		scaffold1:10365261-10366281	0.15	30.74	male
DS10_00000945		scaffold1:10393533-10395188	0.00	43.02	male
DS10_00000946		scaffold1:10411396-10412901	0.00	15.03	male
DS10_00000949		scaffold1:10461604-10467818	0.00	554.21	male
DS10_00000956		scaffold1:10691521-10693877	0.00	45.27	male
DS10_00000978		scaffold1:10818901-10825709	0.35	15.33	male
DS10_00000988		scaffold1:10935831-10936547	4.31	30.47	male
DS10_00001032	tj	scaffold1:11444035-11445586	8.28	0.72	female
DS10_00001060	del	scaffold1:11815869-11819758	13.23	1.56	female
DS10_00001071	TfIIA-S-2	scaffold1:11860444-11860983	0.13	9.85	male
DS10_00001072		scaffold1:11872440-11872977	0.00	404.40	male
DS10_00001073		scaffold1:11876513-11877122	0.07	2.61	male
DS10_00001090		scaffold1:12052001-12053291	0.00	22.63	male
DS10_00001092	qjt	scaffold1:12109369-12110145	0.12	3.59	male
DS10_00001093		scaffold1:12150392-12151253	0.00	73.95	male
DS10_00001097		scaffold1:12236965-12237340	1.93	26.55	male
DS10_00001104		scaffold1:12372747-12373632	0.00	73.37	male
DS10_00001111		scaffold1:12513654-12516235	0.16	24.24	male
DS10_00001114		scaffold1:12540671-12542563	0.05	19.84	male
DS10_00001117		scaffold1:12564943-12571070	0.03	27.51	male
DS10_00001125	qua	scaffold1:12603321-12608905	4.70	43.79	male
DS10_00001127		scaffold1:12617564-12619432	23.46	102.92	male
DS10_00001128	Dhc36C	scaffold1:12620587-12656720	0.09	12.89	male
DS10_00001129		scaffold1:12620587-12656720	0.03	11.69	male
DS10_00001131		scaffold1:12682644-12683862	0.00	40.50	male
DS10_00001143		scaffold1:13147534-13149973	0.00	11.32	male
DS10_00001147		scaffold1:13174291-13176011	0.00	257.07	male
DS10_00001148	elfless	scaffold1:13177214-13177938	0.30	5.92	male
DS10_00001152		scaffold1:13265086-13267501	0.26	7.04	male
DS10_00001153		scaffold1:13272518-13273850	0.00	30.88	male
DS10_00001163		scaffold1:13379551-13408856	1.10	6.19	male
DS10_00001167		scaffold1:13500684-13502643	0.02	63.10	male
DS10_00001173		scaffold1:13620705-13624622	0.00	27.68	male
DS10_00001174	l(1)G0334	scaffold1:13667341-13668909	0.00	15.90	male
DS10_00001195		scaffold1:13919916-13930867	0.12	13.06	male

DS10_00001223		scaffold1:14235658-14236571	0.17	39.42	male
DS10_00001239	robl37BC	scaffold1:14308370-14308715	0.16	26.50	male
DS10_00001256		scaffold1:14408948-14410365	0.04	115.15	male
DS10_00001258		scaffold1:14423600-14425226	11.40	62.62	male
DS10_00001267		scaffold1:14488208-14488361	1.26	314.63	male
DS10_00001268	gammaTub37C	scaffold1:14489987-14492075	14.28	0.28	female
DS10_00001271		scaffold1:14579294-14581436	0.00	7.50	male
DS10_00001272		scaffold1:14622591-14624394	0.00	54.81	male
DS10_00001273		scaffold1:14624751-14626123	0.00	49.21	male
DS10_00001276		scaffold1:14695981-14698503	0.08	74.05	male
DS10_00001281		scaffold1:14725329-14727470	0.24	12.88	male
DS10_00001293		scaffold1:14824400-14825954	0.39	364.35	male
DS10_00001294		scaffold1:14826622-14829529	0.16	159.44	male
DS10_00001298		scaffold1:14864839-14867093	0.00	47.88	male
DS10_00001299	Acp26Ab	scaffold1:14867412-14868010	0.12	257.75	male
DS10_00001300		scaffold1:14875396-14875809	1.95	956.93	male
DS10_00001302	Sfp26Ac	scaffold1:14877644-14878083	0.00	90.49	male
DS10_00001305	TrissinR	scaffold1:14909677-14923768	1.92	23.48	male
DS10_00001306		scaffold1:14945461-14946244	0.00	96.49	male
DS10_00001307		scaffold1:14953862-14954066	1.79	137.78	male
DS10_00001312		scaffold1:14989314-14989959	0.06	74.04	male
DS10_00001319	sip1	scaffold1:15061338-15063975	1.83	22.93	male
DS10_00001325	Hsp60C	scaffold1:15128605-15143614	0.00	102.00	male
DS10_00001329		scaffold1:15225682-15227332	0.00	57.22	male
DS10_00001330		scaffold1:15238464-15240060	0.03	60.09	male
DS10_00001332		scaffold1:15285554-15286617	0.04	19.39	male
DS10_00001346	dtr	scaffold1:15435401-15441851	0.29	19.95	male
DS10_00001353		scaffold1:15486208-15487423	0.00	231.84	male
DS10_00001354		scaffold1:15489924-15490083	1.07	571.71	male
DS10_00001355		scaffold1:15491173-15491335	0.00	540.95	male
DS10_00001361		scaffold1:15562014-15564233	0.00	107.38	male
DS10_00001371		scaffold1:15826132-15827524	0.00	29.30	male
DS10_00001374		scaffold1:15844543-15845443	0.00	17.97	male
DS10_00001375	Su(var)205	scaffold1:15913446-15914124	1.22	9.07	male
DS10_00001376		scaffold1:15927913-15930104	0.02	26.94	male
DS10_00001377		scaffold1:15952696-15954613	0.07	24.84	male
DS10_00001381		scaffold1:15983489-15986459	0.06	19.34	male
DS10_00001383		scaffold1:16001653-16002351	0.00	44.41	male
DS10_00001386		scaffold1:16031692-16032325	0.11	124.26	male
DS10_00001387		scaffold1:16033625-16034100	0.00	668.15	male
DS10_00001402		scaffold1:16096964-16098535	0.15	70.53	male
DS10_00001420		scaffold1:16306743-16308247	0.06	7.55	male
DS10_00001431		scaffold1:16349206-16351153	4.82	0.79	female
DS10_00001441	nop5	scaffold1:16394749-16396611	74.26	17.56	female
DS10_00001451		scaffold1:16527878-16529966	0.14	16.40	male
DS10_00001453		scaffold1:16532317-16536999	0.00	205.72	male
DS10_00001456		scaffold1:16564774-16568840	4.80	28.25	male
DS10_00001460		scaffold1:16600280-16601277	0.29	48.62	male
DS10_00001463	Liprin-alpha	scaffold1:16613343-16625514	5.74	31.34	male
DS10_00001467	Tsp	scaffold1:16644357-16672183	6.97	36.11	male
DS10_00001468		scaffold1:16672782-16673421	0.00	31.04	male
DS10_00001473	Pcp	scaffold1:16714274-16723749	0.22	2.61	male
DS10_00001479		scaffold1:16745773-16775279	0.07	33.71	male
DS10_00001505		scaffold1:17172952-17175907	0.11	18.35	male

DS10_00001518		scaffold1:17279585-17303868	0.15	69.12	male
DS10_00001520	Spn28B	scaffold1:17403108-17404425	0.00	48.84	male
DS10_00001521	Spn88Ea	scaffold1:17420901-17423658	0.00	65.68	male
DS10_00001533		scaffold1:17580913-17618475	0.11	41.14	male
DS10_00001534		scaffold1:17619523-17634956	0.48	23.18	male
DS10_00001542		scaffold1:17685688-17693475	0.01	9.52	male
DS10_00001545		scaffold1:17703076-17715202	0.31	93.87	male
DS10_00001551		scaffold1:17745852-17746755	0.00	42.01	male
DS10_00001562	lectin-30A	scaffold1:18020862-18021946	0.07	44.15	male
DS10_00001565		scaffold1:18106884-18109374	0.04	17.08	male
DS10_00001568		scaffold1:18124136-18125555	0.00	40.04	male
DS10_00001569		scaffold1:18132962-18133364	0.00	73.65	male
DS10_00001570	Acp24A4	scaffold1:18134499-18134814	0.00	228.74	male
DS10_00001577	Fbp2	scaffold1:18225807-18227923	0.26	36.84	male
DS10_00001578	FucTB	scaffold1:18228101-18230576	3.96	30.48	male
DS10_00001580		scaffold1:18235757-18237598	0.49	3.94	male
DS10_00001593	gcm	scaffold1:18422669-18423494	0.07	220.99	male
DS10_00001604	hoip	scaffold1:18562625-18563276	113.13	26.02	female
DS10_00001635		scaffold1:18861158-18866815	4.00	76.64	male
DS10_00001645	Prosalpha6T	scaffold1:18898224-18899094	0.05	19.14	male
DS10_00001646		scaffold1:18899594-18901151	0.32	33.62	male
DS10_00001659		scaffold1:18975384-18981414	0.19	30.67	male
DS10_00001660	Ucp4C	scaffold1:18985266-18986082	0.00	24.42	male
DS10_00001676		scaffold1:19094745-19095327	0.00	32.90	male
DS10_00001677		scaffold1:19095953-19098291	0.02	38.60	male
DS10_00001679	Vm34Ca	scaffold1:19107521-19107878	105.17	0.28	female
DS10_00001685	Hsp60D	scaffold1:19149544-19151230	0.00	50.76	male
DS10_00001687		scaffold1:19154998-19155340	0.18	7.51	male
DS10_00001694		scaffold1:19223474-19227356	0.03	23.11	male
DS10_00001696		scaffold1:19281760-19283209	0.03	70.71	male
DS10_00001699		scaffold1:19341351-19342922	0.03	13.86	male
DS10_00001702		scaffold1:19403488-19406038	0.00	61.89	male
DS10_00001703		scaffold1:19426112-19427569	0.00	53.76	male
DS10_00001740		scaffold1:19808177-19808696	0.00	80.58	male
DS10_00001742		scaffold1:19842949-19851626	103.58	29.62	female
DS10_00001744		scaffold1:19904436-19905688	0.04	19.25	male
DS10_00001764		scaffold1:19996298-19997631	10.29	0.17	female
DS10_00001768		scaffold1:20009781-20011099	14.16	0.23	female
DS10_00001776		scaffold1:20038245-20041243	3.18	42.51	male
DS10_00001783		scaffold1:20084116-20085435	4.45	0.57	female
DS10_00001808		scaffold1:20173961-20182678	8.31	60.69	male
DS10_00001829		scaffold1:20339245-20339857	0.00	42.92	male
DS10_00001830	w-cup	scaffold1:20349641-20351098	0.00	235.62	male
DS10_00001831		scaffold1:20353162-20353759	0.00	116.34	male
DS10_00001832		scaffold1:20354521-20355070	0.00	71.25	male
DS10_00001835		scaffold1:20407272-20421519	0.01	32.39	male
DS10_00001836		scaffold1:20423786-20426596	0.00	37.57	male
DS10_00001838		scaffold1:20454806-20456951	1.89	11.72	male
DS10_00001855		scaffold1:20598695-20599353	0.00	74.35	male
DS10_00001863		scaffold1:20691300-20691723	0.13	2.47	male
DS10_00001864		scaffold1:20704197-20704608	0.13	30.30	male
DS10_00001865		scaffold1:20718861-20719735	0.06	3.42	male
DS10_00001866		scaffold1:20726523-20729044	0.09	34.46	male
DS10_00001867		scaffold1:20730754-20731060	0.42	9.51	male

DS10_00001869		scaffold1:20756901-20762840	0.85	60.76	male
DS10_00001878	piwi	scaffold1:20814284-20824763	28.77	2.86	female
DS10_00001880		scaffold1:20828766-20837432	15.54	57.75	male
DS10_00001881	aub	scaffold1:20838533-20842679	54.97	1.58	female
DS10_00001885	Acp32CD	scaffold1:20862175-20863099	0.09	193.28	male
DS10_00001898		scaffold1:20997871-20999307	0.12	15.97	male
DS10_00001920		scaffold1:21269583-21270266	0.00	360.03	male
DS10_00001922	salr	scaffold1:21298332-21308046	1.29	16.39	male
DS10_00001931		scaffold1:21493839-21496360	0.00	6.80	male
DS10_00001947		scaffold1:21647463-21651035	0.00	7.79	male
DS10_00001948		scaffold1:21654724-21655297	0.00	91.12	male
DS10_00001949		scaffold1:21656050-21657148	0.00	74.19	male
DS10_00001952		scaffold1:21704962-21706981	0.00	43.66	male
DS10_00001973	zuc	scaffold1:22051225-22051987	4.17	0.31	female
DS10_00001990		scaffold1:22154884-22155724	0.10	9.97	male
DS10_00001991	prd	scaffold1:22162179-22164615	0.02	7.24	male
DS10_00002013		scaffold1:22505173-22506657	0.00	49.20	male
DS10_00002016	Pkd2	scaffold1:22552603-22556315	0.09	14.76	male
DS10_00002017		scaffold2:14677-18608	22.54	107.24	male
DS10_00002022		scaffold2:44861-48334	0.02	71.80	male
DS10_00002024	isopeptidase-T-3	scaffold2:63092-65153	4.01	36.50	male
DS10_00002026		scaffold2:73475-74506	0.09	57.83	male
DS10_00002027		scaffold2:78739-81343	0.06	24.00	male
DS10_00002033		scaffold2:158142-161329	0.11	47.38	male
DS10_00002037		scaffold2:206839-207324	0.27	129.44	male
DS10_00002038	sm	scaffold2:213486-234977	13.72	103.43	male
DS10_00002066		scaffold2:463203-467424	0.04	136.96	male
DS10_00002073		scaffold2:533492-535040	0.00	28.54	male
DS10_00002084		scaffold2:688453-688936	0.19	61.61	male
DS10_00002094		scaffold2:769263-775586	0.19	3.18	male
DS10_00002108		scaffold2:956158-957046	0.42	39.14	male
DS10_00002114		scaffold2:1002736-1007223	0.08	18.64	male
DS10_00002132	Obp58b	scaffold2:1157907-1158808	0.07	2.63	male
DS10_00002134		scaffold2:1160298-1171074	0.00	9.36	male
DS10_00002138		scaffold2:1225750-1226934	0.00	17.50	male
DS10_00002139		scaffold2:1252190-1252821	0.00	80.37	male
DS10_00002140		scaffold2:1252987-1254378	0.00	51.82	male
DS10_00002142	CycB	scaffold2:1278946-1281987	66.95	15.91	female
DS10_00002144		scaffold2:1297126-1308431	0.91	4.56	male
DS10_00002161		scaffold2:1411869-1437002	0.60	18.54	male
DS10_00002165		scaffold2:1508562-1509087	0.00	38.40	male
DS10_00002166		scaffold2:1521053-1528920	0.03	37.91	male
DS10_00002178	bgcn	scaffold2:1584278-1589462	1.12	5.97	male
DS10_00002213		scaffold2:1963509-1964529	0.04	24.17	male
DS10_00002223		scaffold2:2004686-2019416	3.02	0.34	female
DS10_00002245		scaffold2:2352575-2354504	0.03	34.59	male
DS10_00002265	Ir60a	scaffold2:2588842-2606040	0.04	3.42	male
DS10_00002270	Nop60B	scaffold2:2684730-2688122	91.11	15.71	female
DS10_00002280		scaffold2:2737032-2745422	2.51	20.12	male
DS10_00002283		scaffold2:2754615-2755200	0.00	82.41	male
DS10_00002312		scaffold2:2857101-2859598	0.29	39.89	male
DS10_00002318		scaffold2:2889840-2893223	0.09	14.09	male
DS10_00002335	CkIIbeta2	scaffold2:2935932-2938050	0.00	30.16	male

DS10_00002341		scaffold2:3002205-3003178	0.00	118.24	male
DS10_00002342		scaffold2:3004181-3007389	0.02	20.23	male
DS10_00002366		scaffold2:3352183-3358727	0.13	23.99	male
DS10_00002373		scaffold2:3369615-3378795	0.13	88.10	male
DS10_00002396		scaffold2:3537316-3547471	0.22	31.73	male
DS10_00002424	MCPH1	scaffold2:3833619-3841465	3.86	60.74	male
DS10_00002428	cuff	scaffold2:3851039-3852659	2.55	0.13	female
DS10_00002472		scaffold2:4443127-4446268	0.00	26.28	male
DS10_00002481		scaffold2:4653208-4654876	0.04	31.65	male
DS10_00002516	Obp50b	scaffold2:4937486-4939507	0.21	18.95	male
DS10_00002546		scaffold2:5230636-5232276	0.00	33.17	male
DS10_00002549	Adgf-E	scaffold2:5253502-5255757	0.00	44.52	male
DS10_00002550		scaffold2:5262702-5263425	0.00	378.41	male
DS10_00002551		scaffold2:5264370-5265216	0.00	370.91	male
DS10_00002552		scaffold2:5266769-5270730	0.04	8.46	male
DS10_00002570		scaffold2:5428369-5430710	0.00	30.21	male
DS10_00002581	wash	scaffold2:5487751-5489248	11.22	54.15	male
DS10_00002592		scaffold2:5570456-5572146	0.17	38.25	male
DS10_00002593	Vha36-2	scaffold2:5574244-5575858	0.25	45.02	male
DS10_00002595		scaffold2:5601014-5601558	0.00	44.39	male
DS10_00002599	SIP2	scaffold2:5626295-5630037	0.06	144.90	male
DS10_00002614		scaffold2:5721125-5725304	0.02	4.80	male
DS10_00002617	Cpr49Ah	scaffold2:5734938-5742886	4.33	0.69	female
DS10_00002661		scaffold2:6212789-6215027	3.96	0.17	female
DS10_00002698	exu	scaffold2:6391016-6393338	39.09	377.65	male
DS10_00002703		scaffold2:6461951-6483313	0.00	52.56	male
DS10_00002724		scaffold2:6713526-6714957	0.06	74.65	male
DS10_00002729		scaffold2:6751461-6754739	0.96	55.75	male
DS10_00002756		scaffold2:6947397-6948342	0.00	61.64	male
DS10_00002759		scaffold2:6953016-6953435	0.54	55.49	male
DS10_00002768		scaffold2:7007243-7007969	1.10	50.82	male
DS10_00002779	sut4	scaffold2:7046925-7048263	0.00	29.12	male
DS10_00002794		scaffold2:7225435-7226843	0.00	24.41	male
DS10_00002795		scaffold2:7227505-7228646	0.22	12.90	male
DS10_00002805		scaffold2:7299587-7300881	0.04	7.25	male
DS10_00002807		scaffold2:7301906-7314120	0.04	3.08	male
DS10_00002809		scaffold2:7351437-7353162	0.09	96.02	male
DS10_00002810		scaffold2:7357118-7357678	0.22	14.75	male
DS10_00002841		scaffold2:7755777-7757827	0.06	40.70	male
DS10_00002843		scaffold2:7763179-7763783	8.25	47.34	male
DS10_00002875		scaffold2:7970176-7987381	3.59	0.61	female
DS10_00002909		scaffold2:8333777-8334477	16.12	90.85	male
DS10_00002913	Mef2	scaffold2:8371710-8372059	2.22	19.83	male
DS10_00002925		scaffold2:8516277-8517081	236.19	0.53	female
DS10_00002926		scaffold2:8517294-8519459	0.03	133.96	male
DS10_00002927		scaffold2:8520292-8521653	33.62	0.05	female
DS10_00002932		scaffold2:8577523-8579121	2.35	73.06	male
DS10_00002942	St1	scaffold2:8639392-8648490	11.98	1.76	female
DS10_00002946		scaffold2:8737882-8739357	0.26	53.04	male
DS10_00002948		scaffold2:8740590-8741487	11.00	55.68	male
DS10_00002949		scaffold2:8741790-8743021	0.06	50.86	male
DS10_00002965		scaffold2:8849835-8850183	0.33	10.71	male
DS10_00002971	skpF	scaffold2:8916367-8916877	0.00	39.26	male
DS10_00002973		scaffold2:8946200-8948457	0.03	5.24	male

DS10_00002993		scaffold2:9082515-9083572	0.10	14.95	male
DS10_00002995		scaffold2:9087818-9088912	0.09	20.95	male
DS10_00002998		scaffold2:9118856-9120032	0.00	45.00	male
DS10_00002999		scaffold2:9125306-9127655	4.52	0.32	female
DS10_00003000		scaffold2:9152044-9153259	0.03	92.42	male
DS10_00003001		scaffold2:9153888-9154608	0.00	187.88	male
DS10_00003005	Crtp	scaffold2:9177672-9180267	0.00	18.94	male
DS10_00003006	Ssl	scaffold2:9183788-9184581	0.00	31.43	male
DS10_00003008	Prosalpha4T2	scaffold2:9187703-9189054	0.00	30.04	male
DS10_00003010		scaffold2:9196654-9197308	0.07	72.46	male
DS10_00003019		scaffold2:9233012-9233456	17.17	2.04	female
DS10_00003026		scaffold2:9273187-9281381	0.07	46.03	male
DS10_00003028		scaffold2:9284143-9304973	0.03	27.69	male
DS10_00003040		scaffold2:9358305-9359743	0.60	96.10	male
DS10_00003046	Pof	scaffold2:9386290-9388019	3.90	110.25	male
DS10_00003086		scaffold2:9778365-9780930	0.95	24.19	male
DS10_00003110		scaffold2:9956399-9958970	0.03	30.97	male
DS10_00003134	krimp	scaffold2:10271865-10275146	7.65	0.43	female
DS10_00003135		scaffold2:10276119-10276801	0.00	79.79	male
DS10_00003180		scaffold2:10575066-10580434	0.00	57.10	male
DS10_00003183		scaffold2:10584357-10585112	0.00	43.47	male
DS10_00003184		scaffold2:10585441-10586581	0.00	33.35	male
DS10_00003185		scaffold2:10587098-10587593	0.00	106.34	male
DS10_00003201		scaffold2:10667547-10672173	0.03	112.61	male
DS10_00003203		scaffold2:10680266-10680833	0.00	70.17	male
DS10_00003205	Menl-2	scaffold2:10739386-10744035	0.00	53.50	male
DS10_00003208		scaffold2:10761093-10762968	0.00	29.52	male
DS10_00003210		scaffold2:10778389-10779478	0.08	23.23	male
DS10_00003215		scaffold2:10828590-10831055	0.35	4.56	male
DS10_00003216	S-Lap8	scaffold2:10833708-10835831	0.02	296.27	male
DS10_00003217		scaffold2:10841384-10843963	0.00	19.82	male
DS10_00003219	loopin-1	scaffold2:10886806-10888390	0.07	541.13	male
DS10_00003238	gom	scaffold2:11004625-11005929	0.00	43.40	male
DS10_00003247	ste24c	scaffold2:11044609-11047994	0.12	26.02	male
DS10_00003249		scaffold2:11091079-11092159	0.00	43.38	male
DS10_00003256		scaffold2:11128303-11128542	0.72	965.34	male
DS10_00003268		scaffold2:11272783-11273952	0.15	22.58	male
DS10_00003269		scaffold2:11274406-11275341	0.36	4.05	male
DS10_00003277	Arp53D	scaffold2:11342746-11344057	0.03	17.27	male
DS10_00003279		scaffold2:11350001-11353087	0.02	8.14	male
DS10_00003281		scaffold2:11354700-11369207	1.06	18.16	male
DS10_00003282	Acp53C14b	scaffold2:11369871-11371158	0.00	37.25	male
DS10_00003284	Sfp53D	scaffold2:11374832-11375273	0.63	79.78	male
DS10_00003285		scaffold2:11380119-11382912	0.00	18.15	male
DS10_00003291		scaffold2:11460518-11462766	0.07	20.37	male
DS10_00003298		scaffold2:11536393-11545983	0.17	355.13	male
DS10_00003300		scaffold2:11547454-11592379	0.00	87.56	male
DS10_00003302		scaffold2:11627269-11628881	0.00	20.00	male
DS10_00003311	Pmm45A	scaffold2:11678895-11680879	33.82	106.28	male
DS10_00003348	l(2)03659	scaffold2:11980019-11984766	0.01	4.19	male
DS10_00003373	clos	scaffold2:12183626-12198411	10.83	1.43	female
DS10_00003384		scaffold2:12356745-12368351	0.39	92.82	male
DS10_00003389	lectin-46Cb	scaffold2:12422928-12426360	2.16	103.49	male
DS10_00003390		scaffold2:12428496-12429098	0.10	56.92	male

DS10_00003403	Ntmt	scaffold2:12475912-12476743	7.43	38.15	male
DS10_00003418	Klp59C	scaffold2:12567992-12570720	0.00	38.41	male
DS10_00003419		scaffold2:12572353-12573698	0.00	41.97	male
DS10_00003421		scaffold2:12585959-12591758	0.02	15.22	male
DS10_00003423	Klp59D	scaffold2:12595094-12596789	0.00	39.60	male
DS10_00003425		scaffold2:12614812-12615814	0.11	3.11	male
DS10_00003427		scaffold2:12642566-12643325	0.07	16.99	male
DS10_00003429		scaffold2:12696861-12697926	0.00	57.86	male
DS10_00003431	RpL22-like	scaffold2:12714142-12715397	0.70	25.09	male
DS10_00003433	ord	scaffold2:12745070-12746900	0.14	10.88	male
DS10_00003434		scaffold2:12758929-12759967	0.27	382.34	male
DS10_00003435		scaffold2:12760314-12760854	0.00	52.97	male
DS10_00003437	Prosbeta5R1	scaffold2:12792841-12793839	0.00	49.91	male
DS10_00003439		scaffold2:12805081-12806002	0.00	57.36	male
DS10_00003440		scaffold2:12808985-12811143	0.00	13.61	male
DS10_00003441		scaffold2:12811492-12814394	0.01	58.07	male
DS10_00003446		scaffold2:12840658-12841384	0.00	27.60	male
DS10_00003453		scaffold2:12885756-12886839	0.07	28.26	male
DS10_00003475	HmgD	scaffold2:12958007-12961514	217.66	59.90	female
DS10_00003477		scaffold2:12962197-12965287	0.62	22.76	male
DS10_00003479	Grx-1	scaffold2:13001839-13002190	0.00	65.53	male
DS10_00003482		scaffold2:13018797-13019605	0.00	130.93	male
DS10_00003483		scaffold2:13020145-13021828	0.00	46.41	male
DS10_00003486		scaffold2:13053998-13054511	0.00	46.16	male
DS10_00003492	PpN58A	scaffold2:13158551-13159502	0.04	34.00	male
DS10_00003509		scaffold2:13373110-13374804	0.10	17.13	male
DS10_00003512		scaffold2:13389962-13391588	15.31	63.36	male
DS10_00003526		scaffold2:13458169-13479805	0.10	35.94	male
DS10_00003531		scaffold2:13496366-13499718	5.75	0.45	female
DS10_00003532		scaffold2:13500615-13511312	9.24	28.96	male
DS10_00003544		scaffold2:13630292-13632020	0.00	25.66	male
DS10_00003546		scaffold2:13687435-13688106	0.00	61.65	male
DS10_00003547		scaffold2:13688801-13690784	0.00	125.84	male
DS10_00003548		scaffold2:13696133-13704226	0.00	116.05	male
DS10_00003551		scaffold2:13716667-13717087	0.00	77.12	male
DS10_00003599	Tpc2	scaffold2:14087333-14124835	0.12	26.03	male
DS10_00003628		scaffold2:14485143-14486316	0.04	24.49	male
DS10_00003633		scaffold3:3970-4296	10.53	0.14	female
DS10_00003643		scaffold3:130284-132529	0.04	105.42	male
DS10_00003669		scaffold3:520047-521010	1.37	15.63	male
DS10_00003676		scaffold3:561848-564347	3.61	20.69	male
DS10_00003698		scaffold3:811404-815716	0.26	18.59	male
DS10_00003703		scaffold3:843637-845034	0.17	42.76	male
DS10_00003706		scaffold3:853625-854789	0.00	26.89	male
DS10_00003708		scaffold3:864009-865698	0.00	38.00	male
DS10_00003710	eIF4E-3	scaffold3:885196-886532	0.09	73.92	male
DS10_00003731	Jon66Ci	scaffold3:1062493-1063276	6.58	0.25	female
DS10_00003734	mkg-p	scaffold3:1068780-1078053	2.79	44.60	male
DS10_00003757		scaffold3:1276479-1295476	1.51	237.25	male
DS10_00003784		scaffold3:1523041-1525246	0.07	50.91	male
DS10_00003786		scaffold3:1566250-1581393	2.41	59.75	male
DS10_00003787	Mcm7	scaffold3:1582368-1584870	13.14	1.53	female
DS10_00003792	orb2	scaffold3:1638581-1645965	7.37	60.99	male
DS10_00003795		scaffold3:1659214-1664311	0.92	18.61	male

DS10_00003810		scaffold3:1755067-1756240	3.01	76.29	male
DS10_00003817	bol	scaffold3:1809475-1816010	35.12	145.68	male
DS10_00003824		scaffold3:1921152-1924790	16.64	2.19	female
DS10_00003825		scaffold3:1945648-1946956	0.03	6.71	male
DS10_00003826		scaffold3:1962882-1964118	0.00	23.68	male
DS10_00003838	Hsp67Bb	scaffold3:2100761-2101624	2.08	34.12	male
DS10_00003875		scaffold3:2328935-2330078	0.00	40.06	male
DS10_00003877		scaffold3:2331635-2332508	0.26	43.39	male
DS10_00003884	alphaTub67C	scaffold3:2413121-2416756	34.86	2.35	female
DS10_00003886		scaffold3:2417963-2428980	2.22	56.69	male
DS10_00003887	scramb1	scaffold3:2432895-2434590	9.24	0.31	female
DS10_00003889		scaffold3:2437681-2441083	0.80	26.99	male
DS10_00003926		scaffold3:2912658-2914352	0.00	19.81	male
DS10_00003928		scaffold3:2937230-2947723	0.04	12.27	male
DS10_00003929		scaffold3:2956405-2957363	0.00	37.52	male
DS10_00003931		scaffold3:3010879-3012227	0.03	27.00	male
DS10_00003932		scaffold3:3014002-3017008	0.06	2.60	male
DS10_00003933	scramb1	scaffold3:3017248-3018082	0.07	42.24	male
DS10_00003935	Kap-alpha3	scaffold3:3042723-3044253	0.00	18.51	male
DS10_00003939		scaffold3:3239393-3240938	0.00	55.79	male
DS10_00003943		scaffold3:3294024-3294672	0.00	85.39	male
DS10_00003944	S-Lap3	scaffold3:3296715-3298329	0.00	253.12	male
DS10_00003946	S-Lap4	scaffold3:3328872-3330447	0.10	382.76	male
DS10_00003947		scaffold3:3404623-3405340	0.00	62.93	male
DS10_00003957		scaffold3:3492803-3495020	21.46	238.76	male
DS10_00003959		scaffold3:3496070-3503076	0.26	36.88	male
DS10_00003975		scaffold3:3803732-3807680	0.20	75.28	male
DS10_00003990		scaffold3:3925417-3926032	0.00	59.92	male
DS10_00003994		scaffold3:4003459-4004089	0.00	47.96	male
DS10_00003995		scaffold3:4014490-4015640	0.09	49.78	male
DS10_00004016		scaffold3:4642024-4642762	0.11	2.12	male
DS10_00004031		scaffold3:4764032-4767761	3.11	27.28	male
DS10_00004043		scaffold3:4950615-4951680	0.36	83.25	male
DS10_00004044		scaffold3:4958614-4959515	0.79	189.26	male
DS10_00004065		scaffold3:5345419-5345843	0.00	134.45	male
DS10_00004071		scaffold3:5430847-5432310	0.52	257.58	male
DS10_00004074		scaffold3:5455137-5455668	0.00	36.64	male
DS10_00004076		scaffold3:5482391-5483522	0.05	11.53	male
DS10_00004084	gnu	scaffold3:5556233-5557030	24.15	0.00	female
DS10_00004091	Ocho	scaffold3:5735391-5735841	0.62	5.00	male
DS10_00004100	Rpn12R	scaffold3:5795805-5796594	0.00	25.99	male
DS10_00004113	lectin-24Db	scaffold3:5882521-5895209	0.06	10.56	male
DS10_00004117	toe	scaffold3:5942906-5953364	1.62	14.73	male
DS10_00004146	Lsp2	scaffold3:6271262-6273563	68.00	2.34	female
DS10_00004176		scaffold3:6571749-6580674	0.11	22.33	male
DS10_00004183		scaffold3:6618227-6619136	0.28	5.07	male
DS10_00004192	Sfp65A	scaffold3:6749117-6749841	0.00	100.03	male
DS10_00004200	ndl	scaffold3:6995291-7004595	2.20	0.07	female
DS10_00004201		scaffold3:7005218-7010898	16.13	84.66	male
DS10_00004202	zpg	scaffold3:7005218-7010898	26.94	1.13	female
DS10_00004203		scaffold3:7011712-7023326	5.68	26.13	male
DS10_00004205	eIF4E-4	scaffold3:7075080-7076505	0.00	38.52	male
DS10_00004214		scaffold3:7180709-7187875	1.03	23.87	male
DS10_00004227		scaffold3:7414409-7417358	0.13	14.39	male

DS10_00004229		scaffold3:7446778-7446943	0.98	111.62	male
DS10_00004243		scaffold3:7582952-7586504	0.00	40.41	male
DS10_00004245		scaffold3:7589686-7601271	0.04	84.38	male
DS10_00004254		scaffold3:7639978-7640666	0.26	22.30	male
DS10_00004267		scaffold3:7732415-7734430	0.00	23.79	male
DS10_00004273	chb	scaffold3:7762500-7766979	10.81	45.18	male
DS10_00004287		scaffold3:7940271-7941250	0.15	6.44	male
DS10_00004289		scaffold3:7959920-7983629	0.01	16.15	male
DS10_00004292		scaffold3:7991999-8017925	0.22	52.29	male
DS10_00004295	Sfp77F	scaffold3:8051831-8052519	0.00	54.16	male
DS10_00004304	Mst77F	scaffold3:8143803-8145052	0.28	265.62	male
DS10_00004311	Six4	scaffold3:8172273-8176484	4.23	0.42	female
DS10_00004332		scaffold3:8487526-8493511	0.14	7.27	male
DS10_00004339		scaffold3:8521059-8521685	0.20	25.88	male
DS10_00004349	aly	scaffold3:8575113-8576973	0.02	2.07	male
DS10_00004350		scaffold3:8586099-8588716	0.00	14.86	male
DS10_00004351	prominin-like	scaffold3:8589634-8594144	0.00	36.39	male
DS10_00004353		scaffold3:8615183-8615784	0.00	75.51	male
DS10_00004359		scaffold3:8646824-8672958	0.67	9.38	male
DS10_00004369		scaffold3:8937926-8978121	0.41	20.59	male
DS10_00004383		scaffold3:9125068-9126202	0.04	22.82	male
DS10_00004393		scaffold3:9263724-9264339	0.00	40.81	male
DS10_00004394		scaffold3:9264423-9264882	0.16	44.42	male
DS10_00004413		scaffold3:9471216-9472590	0.00	15.16	male
DS10_00004420		scaffold3:9655112-9656033	0.00	93.92	male
DS10_00004425		scaffold3:9730176-9731430	0.10	11.07	male
DS10_00004428		scaffold3:9738367-9753236	7.23	22.17	male
DS10_00004443		scaffold3:9859877-9864038	3.13	0.10	female
DS10_00004464		scaffold3:9995826-10014984	0.04	31.51	male
DS10_00004485		scaffold3:10123194-10136277	1.09	104.64	male
DS10_00004486		scaffold3:10136518-10138428	1.04	23.82	male
DS10_00004497		scaffold3:10201058-10208612	0.52	7.50	male
DS10_00004503		scaffold3:10259956-10260246	0.71	6.39	male
DS10_00004505	robl62A	scaffold3:10263879-10267183	0.39	78.53	male
DS10_00004506		scaffold3:10271046-10271688	0.00	33.05	male
DS10_00004509		scaffold3:10321354-10322659	0.00	48.31	male
DS10_00004511	FucTD	scaffold3:10327778-10329158	0.00	39.00	male
DS10_00004531		scaffold3:10514367-10518986	0.04	208.44	male
DS10_00004532		scaffold3:10519376-10520132	0.00	71.02	male
DS10_00004542	LysP	scaffold3:10553364-10553787	303.45	16.01	female
DS10_00004543	LysP	scaffold3:10556685-10557108	147.09	7.08	female
DS10_00004561		scaffold3:10886124-10890330	0.43	10.92	male
DS10_00004594	Ppm1	scaffold3:11236162-11237224	0.00	36.18	male
DS10_00004598		scaffold3:11295584-11297193	0.00	38.10	male
DS10_00004600		scaffold3:11400901-11401375	0.19	54.56	male
DS10_00004602		scaffold3:11468934-11469426	1.24	34.53	male
DS10_00004605	Roc1b	scaffold3:11474304-11477707	0.56	39.91	male
DS10_00004612		scaffold3:11593288-11593873	0.00	51.82	male
DS10_00004615	Dic61B	scaffold3:11697763-11700358	0.06	12.39	male
DS10_00004644		scaffold3:11881758-11903540	0.11	3.87	male
DS10_00004650		scaffold4:57260-58349	0.90	15.65	male
DS10_00004653		scaffold4:100959-101557	0.00	67.34	male
DS10_00004669	HLH3B	scaffold4:304317-307971	0.24	22.56	male
DS10_00004685		scaffold4:411913-415347	0.12	15.26	male

DS10_00004691	SPR	scaffold4:556439-565963	20.58	2.05	female
DS10_00004701		scaffold4:752316-753305	0.05	4.03	male
DS10_00004711		scaffold4:902744-904694	0.22	53.59	male
DS10_00004715		scaffold4:932896-933499	0.00	27.11	male
DS10_00004727	Caf1-180	scaffold4:1076950-1080959	8.43	1.82	female
DS10_00004732	Oxp	scaffold4:1114962-1117407	8.24	1.23	female
DS10_00004734		scaffold4:1124769-1125465	0.82	39.52	male
DS10_00004752		scaffold4:1249358-1251358	0.06	10.10	male
DS10_00004767		scaffold4:1351682-1352255	0.07	13.01	male
DS10_00004776	pcm	scaffold4:1397463-1397940	0.26	8.02	male
DS10_00004801	Inx5	scaffold4:1714383-1720696	0.45	3.85	male
DS10_00004803	Cam	scaffold4:1732809-1733253	0.34	75.32	male
DS10_00004812		scaffold4:1995926-2007371	0.03	26.83	male
DS10_00004826		scaffold4:2121190-2122000	0.00	19.06	male
DS10_00004833	unc	scaffold4:2239240-2243952	0.03	4.02	male
DS10_00004834		scaffold4:2244690-2250853	0.19	2.51	male
DS10_00004848		scaffold4:2402563-2404370	0.00	15.57	male
DS10_00004850		scaffold4:2411174-2414067	0.00	23.62	male
DS10_00004851	l-3	scaffold4:2463752-2464376	0.00	29.47	male
DS10_00004852		scaffold4:2476184-2477483	0.00	27.19	male
DS10_00004853		scaffold4:2515309-2516590	0.00	22.39	male
DS10_00004855		scaffold4:2611027-2612073	0.00	22.87	male
DS10_00004856		scaffold4:2621906-2622770	0.09	147.90	male
DS10_00004858	hydra	scaffold4:2684094-2685796	0.03	53.05	male
DS10_00004859		scaffold4:2686189-2686936	0.51	108.57	male
DS10_00004880		scaffold4:3079809-3080361	0.09	204.48	male
DS10_00004890	Yp1	scaffold4:3245167-3246706	925.42	0.67	female
DS10_00004891	Yp2	scaffold4:3248354-3250103	669.63	0.98	female
DS10_00004939		scaffold4:4069912-4071067	0.16	25.00	male
DS10_00004946	Erk7	scaffold4:4138145-4147237	0.07	25.58	male
DS10_00004965		scaffold4:4231045-4231714	72.34	16.69	female
DS10_00004970	png	scaffold4:4270815-4271691	2.70	0.17	female
DS10_00004977	fs(1)N	scaffold4:4304662-4311637	7.96	0.32	female
DS10_00004981		scaffold4:4353482-4355199	1.42	16.60	male
DS10_00005024	Mcm6	scaffold4:4997520-4999974	8.69	1.38	female
DS10_00005032		scaffold4:5046541-5048752	0.04	43.99	male
DS10_00005034		scaffold4:5090551-5091689	0.05	36.88	male
DS10_00005045	swa	scaffold4:5313748-5315682	18.22	3.43	female
DS10_00005071	p-cup	scaffold5:96579-99346	0.00	11.44	male
DS10_00005074		scaffold5:109861-125165	0.22	18.36	male
DS10_00005077		scaffold5:240969-254057	1.26	26.96	male
DS10_00005148		scaffold5:968819-970141	0.09	12.34	male
DS10_00005176		scaffold5:1235561-1236615	3.62	46.99	male
DS10_00005188		scaffold5:1355219-1356100	0.46	4.72	male
DS10_00005207	Klp3A	scaffold5:1452417-1457002	3.80	19.45	male
DS10_00005224		scaffold5:1749156-1752925	0.05	41.00	male
DS10_00005226		scaffold5:1764588-1766318	0.71	17.27	male
DS10_00005283		scaffold5:2319840-2321844	7.17	34.95	male
DS10_00005286		scaffold5:2343520-2344580	0.27	13.59	male
DS10_00005299		scaffold5:2518831-2520166	0.00	52.65	male
DS10_00005314		scaffold5:2693378-2695661	0.00	28.62	male
DS10_00005325		scaffold5:2837038-2838380	0.00	32.54	male
DS10_00005326		scaffold5:2838737-2839334	0.07	146.55	male
DS10_00005338		scaffold5:3050210-3052844	0.13	3.29	male

DS10_00005352	Dhc16F	scaffold5:3359433-3375718	0.03	4.01	male
DS10_00005364		scaffold5:3451915-3452695	0.00	34.60	male
DS10_00005381		scaffold5:3634736-3636215	0.18	20.08	male
DS10_00005391		scaffold5:3799239-3799869	0.07	12.79	male
DS10_00005392	SIP3	scaffold5:3834406-3835420	0.04	11.65	male
DS10_00005398		scaffold5:3884726-3886494	7.79	0.02	female
DS10_00005402		scaffold5:3926392-3928336	0.00	24.82	male
DS10_00005411		scaffold5:4066295-4080925	0.00	110.69	male
DS10_00005424		scaffold5:4202081-4203933	0.05	18.08	male
DS10_00005429		scaffold5:4281440-4282945	0.04	44.34	male
DS10_00005444		scaffold5:4606540-4608955	0.02	41.52	male
DS10_00005446		scaffold5:4618309-4620439	23.49	2.61	female
DS10_00005449		scaffold5:4642468-4645754	0.00	16.65	male
DS10_00005450		scaffold5:4663419-4667436	0.00	18.60	male
DS10_00005452		scaffold5:4684560-4686120	0.00	22.45	male
DS10_00005456		scaffold5:4714950-4715274	129.67	0.00	female
DS10_00005460		scaffold5:4741363-4745847	0.00	41.77	male
DS10_00005461		scaffold5:4747302-4748125	0.00	37.16	male
DS10_00005462		scaffold5:4748533-4749158	0.00	49.73	male
DS10_00005467		scaffold6:167763-169439	0.32	5.13	male
DS10_00005469		scaffold6:281634-282420	0.00	47.55	male
DS10_00005479	mus209	scaffold6:346137-347938	24.60	1.50	female
DS10_00005498		scaffold6:505803-507264	0.03	10.30	male
DS10_00005507	dup	scaffold6:595052-599157	6.19	0.86	female
DS10_00005511		scaffold6:640582-654232	0.05	10.11	male
DS10_00005517		scaffold6:765630-767261	1.37	71.57	male
DS10_00005535		scaffold6:859363-863123	1.04	13.82	male
DS10_00005540		scaffold6:978850-994039	0.22	3.64	male
DS10_00005563	Ctf4	scaffold6:1309515-1312197	4.51	0.72	female
DS10_00005565		scaffold6:1322710-1323727	1.29	98.94	male
DS10_00005587		scaffold6:1532093-1546571	0.04	3.71	male
DS10_00005594		scaffold6:1579258-1582390	7.07	0.42	female
DS10_00005596	phtf	scaffold6:1604180-1608628	9.07	50.54	male
DS10_00005638		scaffold6:2120638-2130217	0.01	14.87	male
DS10_00005656		scaffold6:2347775-2359657	0.22	43.84	male
DS10_00005659		scaffold6:2382765-2383276	0.11	33.01	male
DS10_00005665		scaffold6:2409273-2413075	3.55	39.52	male
DS10_00005674	dpa	scaffold6:2467521-2470493	10.14	2.03	female
DS10_00005675	didum	scaffold6:2470883-2478784	13.37	46.46	male
DS10_00005684		scaffold6:2509668-2516164	1.24	46.58	male
DS10_00005709		scaffold6:2686797-2694477	1.10	40.37	male
DS10_00005721		scaffold6:2871897-2873517	0.05	10.79	male
DS10_00005741		scaffold6:3044041-3044392	0.17	33.93	male
DS10_00005753	nopo	scaffold6:3192916-3194570	11.55	1.89	female
DS10_00005764		scaffold6:3216073-3279212	0.45	81.19	male
DS10_00005771		scaffold6:3410580-3411207	0.08	30.46	male
DS10_00005785	Jabba	scaffold6:3579264-3583975	50.41	7.61	female
DS10_00005809	Vha100-3	scaffold6:3758153-3760913	0.00	7.89	male
DS10_00005812	Topors	scaffold6:3832176-3839731	4.00	18.99	male
DS10_00005817		scaffold6:3931755-3932996	0.00	96.65	male
DS10_00005818		scaffold6:3937794-3939434	0.05	74.67	male
DS10_00005831	wbl	scaffold6:4166813-4168367	3.84	32.62	male
DS10_00005858		scaffold6:4350770-4354027	0.60	17.52	male
DS10_00005863		scaffold7:32163-34888	0.05	9.81	male

DS10_00005896		scaffold7:246227-247538	0.09	7.00	male
DS10_00005904		scaffold7:274639-286405	2.10	10.85	male
DS10_00005908		scaffold7:295878-305867	0.18	6.44	male
DS10_00005930		scaffold7:488707-497765	0.06	8.87	male
DS10_00005931		scaffold7:488707-497765	0.09	7.60	male
DS10_00005937		scaffold7:530966-534276	0.00	23.63	male
DS10_00005950		scaffold7:795990-796998	0.00	21.39	male
DS10_00005951		scaffold7:797548-798855	0.28	11.37	male
DS10_00005969		scaffold7:896058-898446	0.04	9.40	male
DS10_00005973		scaffold7:937028-943591	219.58	12.21	female
DS10_00005978		scaffold7:1105615-1109068	0.00	35.97	male
DS10_00005979		scaffold7:1152159-1152606	0.18	3.21	male
DS10_00005980		scaffold7:1153163-1155396	0.17	2.06	male
DS10_00005981		scaffold7:1156411-1161004	0.03	4.49	male
DS10_00005982		scaffold7:1161523-1162894	0.04	17.25	male
DS10_00005983		scaffold7:1164805-1166625	0.12	2.39	male
DS10_00006002		scaffold7:1464949-1470274	0.18	14.19	male
DS10_00006003		scaffold7:1470670-1471714	0.10	15.30	male
DS10_00006004		scaffold7:1473261-1476671	0.09	23.91	male
DS10_00006006		scaffold7:1485537-1486492	0.22	12.40	male
DS10_00006010		scaffold7:1524710-1527737	0.11	83.95	male
DS10_00006018		scaffold7:1545063-1546484	0.09	30.79	male
DS10_00006022		scaffold7:1582892-1583465	0.00	68.79	male
DS10_00006031		scaffold7:1738332-1747665	1.99	99.02	male
DS10_00006053	fs(1)Ya	scaffold7:1862755-1866700	10.78	0.25	female
DS10_00006059		scaffold7:1899591-1900182	0.36	5.13	male
DS10_00006063		scaffold7:1968385-1969775	0.18	2.66	male
DS10_00006089		scaffold7:2733091-2734225	0.29	15.86	male
DS10_00006090		scaffold7:2776832-2777408	0.17	15.01	male
DS10_00006091		scaffold7:2811397-2812000	0.00	100.65	male
DS10_00006093		scaffold7:2816629-2817862	0.00	15.23	male
DS10_00006099	Gas8	scaffold7:2852486-2857649	0.12	9.53	male
DS10_00006101		scaffold7:2867249-2888812	0.00	24.02	male
DS10_00006114	yin	scaffold7:3294104-3299262	30.26	5.89	female
DS10_00006122		scaffold7:3417935-3420215	4.15	0.51	female
DS10_00006138		scaffold7:3843152-3856415	0.00	8.74	male
DS10_00006139		scaffold7:3856848-3858343	0.00	13.91	male
DS10_00006149		scaffold7:3983703-3985774	0.02	3.24	male
DS10_00006158		scaffold7:4074658-4075270	0.00	42.21	male
DS10_00006185		scaffold11:56497-63456	0.16	20.73	male
DS10_00006188		scaffold11:75238-82731	1.64	16.68	male
DS10_00006202		scaffold11:158355-159378	0.10	7.79	male
DS10_00006229	Pp1-13C	scaffold11:453105-454014	0.05	16.15	male
DS10_00006230	acj6	scaffold11:459867-463048	0.44	5.78	male
DS10_00006240		scaffold11:560407-562288	0.00	24.48	male
DS10_00006250		scaffold11:774143-795196	0.31	13.63	male
DS10_00006267		scaffold11:972787-982214	0.09	48.13	male
DS10_00006283	Cyp28c1	scaffold11:1122430-1125230	4.12	20.75	male
DS10_00006284		scaffold11:1129788-1130741	0.00	18.08	male
DS10_00006300		scaffold11:1270333-1271632	0.22	16.43	male
DS10_00006318		scaffold11:1412414-1420327	0.08	37.38	male
DS10_00006329	mkg-p	scaffold11:1518835-1520708	0.04	3.63	male
DS10_00006338		scaffold11:1607493-1609515	0.02	20.53	male
DS10_00006358		scaffold11:1757036-1757398	0.19	16.98	male

DS10_00006368		scaffold11:1848668-1851063	4.34	42.24	male
DS10_00006377		scaffold11:1977831-1979594	0.10	19.85	male
DS10_00006382		scaffold11:2081365-2082096	0.07	34.45	male
DS10_00006387		scaffold11:2095456-2103248	0.00	37.61	male
DS10_00006391	mei-218	scaffold11:2160941-2168892	1.60	16.68	male
DS10_00006394	xmas-2	scaffold11:2182182-2190490	1.77	13.17	male
DS10_00006409		scaffold11:2404538-2405392	0.07	42.47	male
DS10_00006424		scaffold11:2508184-2512393	0.22	5.22	male
DS10_00006425		scaffold11:2567529-2569632	0.08	7.94	male
DS10_00006436		scaffold11:2844106-2844441	0.00	202.70	male
DS10_00006444		scaffold11:2911869-2913510	0.05	6.07	male
DS10_00006461		scaffold11:3226405-3227743	0.00	34.22	male
DS10_00006462		scaffold11:3237768-3238962	0.03	17.55	male
DS10_00006465		scaffold11:3272668-3273703	0.00	30.03	male
DS10_00006467		scaffold11:3467501-3468726	0.00	21.06	male
DS10_00006468		scaffold11:3608323-3614946	0.01	15.00	male
DS10_00006471		scaffold11:3640734-3641100	0.00	435.05	male
DS10_00006478	Uch-L5R	scaffold11:3698403-3699408	0.00	25.25	male
DS10_00006503		scaffold11:3987213-3987858	0.00	46.08	male
DS10_00006505		scaffold11:4044313-4045234	0.00	25.59	male
DS10_00006511		scaffold8:86066-87863	0.35	19.90	male
DS10_00006516		scaffold8:184290-184662	0.17	8.18	male
DS10_00006517		scaffold8:188653-189954	0.66	7.20	male
DS10_00006521	tomboy40	scaffold8:241957-242983	0.04	57.99	male
DS10_00006535		scaffold8:502194-520298	0.03	18.46	male
DS10_00006545	S-Lap7	scaffold8:592606-594190	0.00	410.26	male
DS10_00006550		scaffold8:688696-702290	0.07	30.72	male
DS10_00006551		scaffold8:704682-706317	0.15	39.17	male
DS10_00006554	S-Lap5	scaffold8:779985-781644	0.02	48.04	male
DS10_00006559		scaffold8:824897-825980	0.00	41.41	male
DS10_00006567		scaffold8:872761-894121	0.19	2.73	male
DS10_00006568		scaffold8:872761-894121	0.34	15.43	male
DS10_00006585		scaffold8:1013289-1017948	0.01	7.92	male
DS10_00006587		scaffold8:1019441-1023931	0.02	9.27	male
DS10_00006589		scaffold8:1062168-1063322	0.00	45.06	male
DS10_00006595		scaffold8:1174784-1181650	0.03	42.47	male
DS10_00006608		scaffold8:1224914-1231408	0.27	96.30	male
DS10_00006622		scaffold8:1320716-1321331	0.00	37.62	male
DS10_00006624		scaffold8:1336410-1343378	0.02	61.27	male
DS10_00006632		scaffold8:1391344-1410707	6.55	34.56	male
DS10_00006637		scaffold8:1487451-1488915	927.70	171.07	female
DS10_00006643	mos	scaffold8:1499518-1500782	3.46	0.46	female
DS10_00006681		scaffold8:1786221-1804472	0.97	16.79	male
DS10_00006695	Odc2	scaffold8:1888192-1916422	0.32	4.79	male
DS10_00006735		scaffold8:2210195-2210543	0.00	68.56	male
DS10_00006737		scaffold8:2214005-2214746	0.05	144.98	male
DS10_00006739		scaffold8:2324771-2325608	0.00	59.74	male
DS10_00006740	spaw	scaffold8:2325660-2326968	0.00	127.34	male
DS10_00006741	cola	scaffold8:2327278-2334014	0.01	25.75	male
DS10_00006742		scaffold8:2334015-2334495	0.00	212.76	male
DS10_00006743		scaffold8:2335957-2336790	0.00	210.05	male
DS10_00006744		scaffold8:2338617-2339358	0.00	329.26	male
DS10_00006772		scaffold8:2630379-2631113	0.00	40.80	male
DS10_00006784		scaffold8:2715490-2734019	0.75	64.24	male

DS10_00006789		scaffold8:2752004-2755975	0.05	28.06	male
DS10_00006792	PGRP-SC1b	scaffold8:2758829-2765154	4.85	0.32	female
DS10_00006793	PGRP-SC1b	scaffold8:2765972-2766530	8.40	1.22	female
DS10_00006800		scaffold8:2847772-2848792	0.00	42.31	male
DS10_00006801	sns	scaffold8:2869724-2917327	1.38	10.77	male
DS10_00006815		scaffold10:93548-94198	634.50	180.92	female
DS10_00006829	vanin-like	scaffold10:218581-227122	5.22	1.03	female
DS10_00006852	otu	scaffold10:470344-475526	15.06	1.18	female
DS10_00006883		scaffold10:917672-918833	0.10	187.09	male
DS10_00006899		scaffold10:1143119-1145333	5.42	24.90	male
DS10_00006906		scaffold10:1186123-1187038	0.67	8.57	male
DS10_00006907	Klp10A	scaffold10:1187489-1191967	15.22	59.49	male
DS10_00006920		scaffold10:1275423-1275912	0.00	329.86	male
DS10_00006921		scaffold10:1299238-1299658	0.00	94.73	male
DS10_00006925		scaffold10:1355058-1355451	0.14	58.78	male
DS10_00006937	Rpn13R	scaffold10:1523785-1524742	0.00	16.70	male
DS10_00006939		scaffold10:1582003-1584248	0.04	12.39	male
DS10_00006942		scaffold10:1752751-1759185	0.07	23.51	male
DS10_00006943		scaffold10:1759372-1760089	0.06	20.47	male
DS10_00006946	TrxT	scaffold10:1764826-1766244	0.37	148.68	male
DS10_00006947	dhd	scaffold10:1766471-1766795	563.60	0.77	female
DS10_00006948		scaffold10:1767674-1770394	0.39	70.98	male
DS10_00006949		scaffold10:1771336-1774800	3.60	0.25	female
DS10_00006952	Mcm3	scaffold10:1781046-1785372	5.23	0.70	female
DS10_00006967		scaffold10:2086843-2087542	0.00	34.08	male
DS10_00006974		scaffold10:2156844-2167311	0.09	20.59	male
DS10_00006975		scaffold10:2167425-2168372	22.25	0.12	female
DS10_00006976	CBP	scaffold10:2175729-2181693	13.10	0.73	female
DS10_00006978		scaffold10:2202081-2202669	0.00	105.05	male
DS10_00006981		scaffold10:2252184-2253324	0.00	25.47	male
DS10_00006984		scaffold10:2265180-2265710	472.55	0.00	female
DS10_00006992		scaffold10:2314453-2315167	0.20	4.65	male
DS10_00006993		scaffold10:2356761-2361432	0.05	50.15	male
DS10_00006994		scaffold10:2363333-2364212	0.00	47.36	male
DS10_00006995		scaffold10:2377555-2382719	0.02	4.94	male
DS10_00006997		scaffold10:2411128-2412124	0.00	24.56	male
DS10_00006998		scaffold10:2457284-2459345	0.00	19.29	male
DS10_00006999		scaffold10:2472252-2472858	0.00	86.50	male
DS10_00007014		scaffold10:2790155-2791340	0.00	13.07	male
DS10_00007017	1-Dec	scaffold10:2808812-2815061	8.95	0.03	female
DS10_00007038		scaffold10:2902159-2905893	0.00	14.02	male
DS10_00007083	fd102C	scaffold9:798124-800113	0.19	10.92	male
DS10_00007087	ey	scaffold9:949904-976416	1.28	12.00	male
DS10_00007146	JYalpha	scaffold9:2329358-2339823	0.03	6.99	male
DS10_00007159	zye	scaffold12:203463-211816	4.28	0.54	female
DS10_00007176	Spn77Bc	scaffold12:287584-291150	0.00	38.30	male
DS10_00007179		scaffold12:294676-295998	0.57	37.23	male
DS10_00007207		scaffold12:501124-502015	0.00	21.29	male
DS10_00007251	SMSr	scaffold12:939057-941884	7.63	34.78	male
DS10_00007253		scaffold12:947233-949264	0.00	103.10	male
DS10_00007258		scaffold12:1033069-1033842	0.00	200.76	male
DS10_00007261		scaffold12:1049219-1052543	1.19	7.33	male
DS10_00007263	Tsp66A	scaffold12:1063018-1067310	0.00	8.77	male
DS10_00007278		scaffold12:1203302-1211004	0.00	4.21	male

DS10_00007279		scaffold12:1213473-1215603	0.00	15.35	male
DS10_00007298	wisp	scaffold13:300371-305834	17.84	0.04	female
DS10_00007346		scaffold13:808686-811463	0.14	15.28	male
DS10_00007350		scaffold13:834369-835128	0.13	18.95	male
DS10_00007351	Muc14A	scaffold13:835186-840669	0.06	2.79	male
DS10_00007352		scaffold13:842257-844629	0.08	5.80	male
DS10_00007369		scaffold21:126089-135509	0.34	3.01	male
DS10_00007372	gskt	scaffold21:264365-265874	0.00	42.55	male
DS10_00007380		scaffold21:366987-373020	0.06	16.06	male
DS10_00007397		scaffold21:653267-656496	0.02	33.96	male
DS10_00007417		scaffold21:766202-767462	0.72	72.09	male
DS10_00007424		scaffold21:1052080-1053100	0.00	18.99	male
DS10_00007432	Mst98Ca	scaffold39:137777-151585	0.03	94.88	male
DS10_00007454		scaffold39:782650-783434	0.54	56.76	male
DS10_00007468		scaffold14:187579-190606	0.00	45.07	male
DS10_00007476	yl	scaffold14:438787-446137	81.69	0.02	female
DS10_00007480		scaffold14:475635-477550	46.40	2.15	female
DS10_00007494	betaNACtes6	scaffold14:657059-657968	0.00	39.58	male
DS10_00007520	armi	scaffold27:38241-43694	16.91	1.93	female
DS10_00007530		scaffold27:281243-282310	0.00	21.62	male
DS10_00007540		scaffold27:503128-505114	6.27	30.01	male
DS10_00007552		scaffold27:673756-674146	0.00	167.53	male
DS10_00007568	Dpy-30L2	scaffold27:808008-808308	0.00	138.89	male
DS10_00007570		scaffold27:825846-827998	1.17	32.84	male
DS10_00007594		scaffold26:255852-259092	0.00	34.01	male
DS10_00007622		scaffold26:659685-660738	0.08	21.20	male
DS10_00007631		scaffold26:752709-755949	0.02	4.34	male
DS10_00007634		scaffold26:795630-797175	0.00	30.55	male
DS10_00007640		scaffold18:143187-145497	0.00	14.53	male
DS10_00007652		scaffold18:464432-465914	0.00	64.24	male
DS10_00007653		scaffold18:466695-467574	0.28	36.51	male
DS10_00007654		scaffold18:490931-491510	1.25	12.40	male
DS10_00007656		scaffold18:552722-553343	0.00	28.59	male
DS10_00007674		scaffold18:835626-836821	0.18	2.10	male
DS10_00007675		scaffold28:10419-12209	0.00	13.37	male
DS10_00007677	ACXC	scaffold28:172306-175303	0.00	26.36	male
DS10_00007678	ACXD	scaffold28:191504-199215	0.03	7.99	male
DS10_00007679	Vm26Aa	scaffold28:213387-213822	77.54	0.11	female
DS10_00007681	ACXE	scaffold28:262124-296560	0.01	4.26	male
DS10_00007694		scaffold28:542350-547277	0.02	26.95	male
DS10_00007702	DnaJ-H	scaffold28:590455-596067	10.52	53.39	male
DS10_00007713		scaffold28:641488-642969	1.40	15.61	male
DS10_00007749		scaffold31:340360-340837	0.00	61.48	male
DS10_00007776		scaffold31:685286-686087	0.08	57.89	male
DS10_00007777		scaffold31:691055-691931	0.00	47.95	male
DS10_00007783		scaffold15:142532-145109	0.00	7.48	male
DS10_00007784	His2Av	scaffold15:219270-219699	0.12	24.82	male
DS10_00007800	Clk	scaffold15:405294-411508	2.50	57.57	male
DS10_00007804		scaffold15:510198-553746	2.41	20.09	male
DS10_00007821		scaffold15:665820-669478	0.14	42.52	male
DS10_00007863		scaffold16:289821-299121	0.00	11.18	male
DS10_00007915		scaffold16:660199-661654	0.10	15.01	male
DS10_00007918		scaffold16:731965-734517	0.05	20.18	male
DS10_00007919		scaffold16:740429-740858	0.31	5.09	male

DS10_00007922		scaffold17:39678-48875	0.00	18.77	male
DS10_00007937		scaffold17:391248-392399	0.04	78.21	male
DS10_00007945		scaffold17:594674-611204	0.02	8.08	male
DS10_00007946		scaffold17:648977-649992	0.49	10.82	male
DS10_00007949	Trxr-2	scaffold17:654436-655807	0.03	17.82	male
DS10_00007952		scaffold41:23561-25109	0.26	11.79	male
DS10_00007978	Andorra	scaffold20:89341-90088	0.06	5.65	male
DS10_00007986	os	scaffold20:237242-238809	0.21	4.44	male
DS10_00007998		scaffold20:363882-364281	0.36	2.78	male
DS10_00007999		scaffold20:379292-381321	0.02	42.96	male
DS10_00008008		scaffold20:447615-449364	0.02	5.29	male
DS10_00008018		scaffold20:518419-521421	0.00	23.30	male
DS10_00008025	Fcp1	scaffold20:659406-661955	0.53	4.09	male
DS10_00008027		scaffold20:681528-687751	0.00	7.00	male
DS10_00008030		scaffold20:727921-732287	0.13	9.54	male
DS10_00008037		scaffold19:234383-236495	0.00	7.85	male
DS10_00008075		scaffold45:346519-349433	0.16	71.71	male
DS10_00008089		scaffold45:566655-571358	0.10	21.69	male
DS10_00008090		scaffold45:598326-599512	0.00	53.82	male
DS10_00008093		scaffold45:623154-624574	0.00	31.17	male
DS10_00008107		scaffold25:107177-108701	0.05	6.67	male
DS10_00008132	Tektin-C	scaffold25:405659-408530	0.07	22.53	male
DS10_00008166		scaffold22:443864-445907	0.02	25.39	male
DS10_00008187	Prosbeta2R1	scaffold23:135811-137935	0.00	19.11	male
DS10_00008188		scaffold23:144580-145231	3.37	0.26	female
DS10_00008194		scaffold23:222209-223205	0.04	33.26	male
DS10_00008206	fs(1)M3	scaffold23:323485-329519	13.01	0.01	female
DS10_00008249		scaffold24:507863-512537	0.22	35.16	male
DS10_00008256		scaffold98:69591-71523	0.00	11.60	male
DS10_00008262		scaffold98:88556-98843	0.25	10.26	male
DS10_00008264		scaffold98:99116-101048	0.00	12.97	male
DS10_00008276		scaffold98:235744-238007	5.86	0.23	female
DS10_00008280		scaffold98:317579-319098	0.00	26.75	male
DS10_00008319		scaffold29:209029-211147	0.77	45.39	male
DS10_00008325		scaffold29:344469-346317	0.00	26.52	male
DS10_00008326		scaffold29:347519-351614	4.19	0.13	female
DS10_00008338	rha	scaffold29:533155-537963	0.05	21.51	male
DS10_00008361	vfl	scaffold30:191843-204776	4.85	0.76	female
DS10_00008368	PPP4R2r	scaffold30:240196-246311	0.29	2.78	male
DS10_00008378	BthD	scaffold30:291833-292575	10.51	0.84	female
DS10_00008390		scaffold30:388758-412662	5.93	39.69	male
DS10_00008398		scaffold30:457143-457673	12.20	0.20	female
DS10_00008400	Yp3	scaffold30:460197-462643	316.43	1.90	female
DS10_00008404		scaffold30:503614-504793	0.00	14.79	male
DS10_00008431		scaffold33:432457-458737	0.02	6.88	male
DS10_00008432		scaffold33:502416-503568	0.00	63.78	male
DS10_00008434		scaffold34:53240-55513	0.65	4.82	male
DS10_00008437		scaffold34:57354-60680	0.25	38.91	male
DS10_00008441		scaffold34:80041-82738	0.03	2.96	male
DS10_00008444	t-cup	scaffold34:139804-140518	0.00	57.36	male
DS10_00008454		scaffold34:412975-417512	0.11	32.21	male
DS10_00008464	Vm26Ab	scaffold34:456995-457748	43.54	0.12	female
DS10_00008483	fig	scaffold40:151432-152392	0.26	53.15	male
DS10_00008519		scaffold40:363444-381709	2.29	17.94	male

DS10_00008538		scaffold32:268722-271382	0.02	10.25	male
DS10_00008539		scaffold32:271800-276593	2.99	13.74	male
DS10_00008544		scaffold32:348583-350197	0.02	12.26	male
DS10_00008545		scaffold32:361381-362413	0.06	15.93	male
DS10_00008568		scaffold70:118442-119915	0.04	23.82	male
DS10_00008573		scaffold70:151401-152657	0.00	21.35	male
DS10_00008576	dpr17	scaffold70:248465-254109	1.57	51.24	male
DS10_00008579		scaffold70:305770-310381	0.00	39.78	male
DS10_00008580		scaffold70:330088-333568	0.00	28.77	male
DS10_00008581		scaffold70:346722-348473	0.00	18.97	male
DS10_00008589	mfas	scaffold70:400846-410772	33.56	129.40	male
DS10_00008598		scaffold120:91566-96391	0.02	15.00	male
DS10_00008608	laf	scaffold120:236362-252695	0.01	11.30	male
DS10_00008629	KP78a	scaffold123:98821-104413	0.12	6.03	male
DS10_00008631		scaffold123:225965-235590	0.50	243.27	male
DS10_00008636		scaffold123:250654-251488	0.23	230.60	male
DS10_00008650	Ugt35b	scaffold123:305153-314429	24.40	7.06	female
DS10_00008657		scaffold123:367469-370120	0.02	39.79	male
DS10_00008666		scaffold35:216844-219659	0.00	43.13	male
DS10_00008670		scaffold35:343716-354824	0.15	10.92	male
DS10_00008672		scaffold35:362460-363192	0.00	28.34	male
DS10_00008677		scaffold35:395139-402254	4.06	37.63	male
DS10_00008689		scaffold36:37528-38686	0.04	14.02	male
DS10_00008692	Dhc16F	scaffold36:78105-139944	0.07	7.69	male
DS10_00008698		scaffold36:256944-257379	0.11	27.40	male
DS10_00008702		scaffold36:445802-446062	3.02	0.29	female
DS10_00008717		scaffold251:8685-16705	0.08	2.51	male
DS10_00008728		scaffold251:76817-82902	21.65	5.78	female
DS10_00008735		scaffold251:212534-213719	0.94	15.73	male
DS10_00008750		scaffold251:401467-414158	0.17	8.63	male
DS10_00008772		scaffold37:163617-164765	0.45	131.80	male
DS10_00008785		scaffold37:311334-312150	0.00	23.23	male
DS10_00008799		scaffold37:440538-441649	0.00	31.20	male
DS10_00008822		scaffold97:84674-85712	0.00	58.72	male
DS10_00008826		scaffold97:96371-105536	0.12	26.94	male
DS10_00008843	Spn75F	scaffold97:397906-399153	0.04	21.30	male
DS10_00008851		scaffold38:53555-54995	0.15	2.80	male
DS10_00008859		scaffold38:152093-154658	0.00	7.46	male
DS10_00008860		scaffold38:156299-179786	0.02	8.19	male
DS10_00008866	Rpt4R	scaffold38:258850-260124	1.05	11.90	male
DS10_00008867	Rpt4R	scaffold38:260463-262245	2.34	25.25	male
DS10_00008877		scaffold38:319254-320306	0.15	26.05	male
DS10_00008884		scaffold38:375777-376164	0.14	101.87	male
DS10_00008888	His3:CG33842	scaffold48:3946-4345	8.06	0.16	female
DS10_00008890	His3:CG33842	scaffold48:11554-11965	24.78	1.13	female
DS10_00008891	His4:CG33905	scaffold48:12258-12569	22.11	1.62	female
DS10_00008892	His2Av	scaffold48:13056-13431	81.85	12.01	female
DS10_00008894	His1:CG33807	scaffold48:14512-15276	10.44	0.77	female
DS10_00008895	His3:CG33842	scaffold48:16743-17100	10.72	0.22	female
DS10_00008912		scaffold48:405341-407451	0.06	6.68	male
DS10_00008925		scaffold200:231980-234802	0.00	29.42	male
DS10_00008928		scaffold200:247345-248260	0.00	36.83	male
DS10_00008929		scaffold200:250047-254091	1.73	30.34	male
DS10_00008936		scaffold200:349125-356203	0.00	37.08	male

DS10_00008939		scaffold200:369438-387436	0.03	10.59	male
DS10_00008960		scaffold42:148243-149131	0.00	17.22	male
DS10_00008981		scaffold42:419462-420296	0.00	39.46	male
DS10_00009070		scaffold59:135866-138046	0.60	29.23	male
DS10_00009098		scaffold44:27144-28113	0.00	24.47	male
DS10_00009099		scaffold44:28734-30085	0.00	14.89	male
DS10_00009101		scaffold44:44631-53720	0.00	19.78	male
DS10_00009107		scaffold44:214464-215355	0.06	16.92	male
DS10_00009122		scaffold68:43836-44574	0.00	47.65	male
DS10_00009124		scaffold68:75515-76332	0.00	72.81	male
DS10_00009132		scaffold68:216057-218436	0.00	25.25	male
DS10_00009150		scaffold290:243408-245898	0.00	25.45	male
DS10_00009153		scaffold290:278961-283356	0.00	45.59	male
DS10_00009163		scaffold46:85273-86068	0.11	12.18	male
DS10_00009179		scaffold46:250815-256222	0.30	5.58	male
DS10_00009181		scaffold46:250815-256222	1.45	53.40	male
DS10_00009264	twin	scaffold94:61408-71774	17.72	72.33	male
DS10_00009286		scaffold94:238463-242205	2.16	15.00	male
DS10_00009288		scaffold94:243971-244685	0.00	619.42	male
DS10_00009311		scaffold93:223133-224702	0.00	23.35	male
DS10_00009322		scaffold52:21154-31495	0.01	15.17	male
DS10_00009334		scaffold52:195615-197757	0.00	125.71	male
DS10_00009344		scaffold52:279966-285767	0.03	10.47	male
DS10_00009357		scaffold52:369175-371206	0.13	38.69	male
DS10_00009358	djl	scaffold52:372584-373539	0.60	51.67	male
DS10_00009359		scaffold52:375922-379108	2.24	36.31	male
DS10_00009373		scaffold55:219284-221504	0.02	19.23	male
DS10_00009374	Cyp313a3	scaffold55:241786-251486	0.00	10.80	male
DS10_00009377		scaffold55:258712-259754	0.03	20.31	male
DS10_00009381		scaffold55:291684-294875	0.00	19.47	male
DS10_00009382		scaffold55:297363-300909	0.03	7.04	male
DS10_00009405	topi	scaffold203:230594-238019	0.20	7.70	male
DS10_00009428	Ppi1	scaffold49:38228-50052	0.07	82.82	male
DS10_00009441		scaffold49:159503-160044	0.13	386.53	male
DS10_00009454	Hsc70-2	scaffold64:169-2649	0.60	28.19	male
DS10_00009472	stg	scaffold160:63395-74011	3.01	0.18	female
DS10_00009476		scaffold160:127212-128809	0.16	3.33	male
DS10_00009479		scaffold160:147314-149494	0.21	3.18	male
DS10_00009497	osk	scaffold65:2-24372	78.69	0.19	female
DS10_00009516		scaffold65:328067-329391	0.00	18.85	male
DS10_00009517		scaffold65:331190-332609	0.32	51.62	male
DS10_00009520		scaffold65:339703-341181	0.06	18.66	male
DS10_00009543	spdo	scaffold53:91953-108890	40.95	12.86	female
DS10_00009546		scaffold53:128168-143001	0.00	17.53	male
DS10_00009553	Rpt6R	scaffold53:289803-292925	0.02	13.65	male
DS10_00009563		scaffold53:349138-350334	0.03	77.74	male
DS10_00009572		scaffold156:286496-287458	0.00	46.05	male
DS10_00009573		scaffold156:296299-302947	0.00	29.25	male
DS10_00009576	c(3)G	scaffold156:351831-354645	7.53	1.40	female
DS10_00009579		scaffold56:30589-31197	0.06	3.68	male
DS10_00009605	mura	scaffold56:116677-124125	8.54	30.07	male
DS10_00009628		scaffold56:301649-305711	1.56	18.57	male
DS10_00009629	soti	scaffold56:325038-325485	0.00	39.73	male
DS10_00009679		scaffold106:290801-299745	0.01	19.82	male

DS10_00009687		scaffold106:324568-336811	0.17	240.89	male
DS10_00009694		scaffold89:22065-22974	0.00	80.89	male
DS10_00009712		scaffold89:267348-269625	0.03	23.24	male
DS10_00009726		scaffold61:17395-18076	0.31	5.44	male
DS10_00009761		scaffold72:247594-249297	0.04	12.36	male
DS10_00009762		scaffold72:280742-281690	0.00	34.41	male
DS10_00009772		scaffold66:107969-119420	0.00	8.58	male
DS10_00009776	cdm	scaffold66:145727-151183	11.71	184.51	male
DS10_00009781		scaffold66:176919-177210	0.77	10.89	male
DS10_00009806		scaffold66:286752-294581	9.59	44.52	male
DS10_00009824		scaffold162:290063-292217	0.05	16.61	male
DS10_00009825		scaffold162:292659-294788	0.02	31.58	male
DS10_00009864		scaffold177:176434-177535	0.00	53.89	male
DS10_00009865		scaffold177:177815-178727	0.00	57.04	male
DS10_00009870		scaffold177:296955-297488	0.00	47.13	male
DS10_00009876	psd	scaffold71:248836-249877	16.79	1.20	female
DS10_00009910		scaffold77:176401-184878	0.09	3.06	male
DS10_00009924		scaffold78:239572-249250	19.61	71.02	male
DS10_00009943		scaffold223:156300-157091	0.07	6.48	male
DS10_00009958		scaffold81:75968-76864	0.04	5.94	male
DS10_00009959		scaffold81:96847-97743	0.13	20.31	male
DS10_00009962	Actn3	scaffold81:280255-282078	0.00	11.74	male
DS10_00009971		scaffold102:47632-49118	0.19	5.49	male
DS10_00009973		scaffold102:49271-53515	0.16	4.17	male
DS10_00010001		scaffold102:164888-182559	0.76	18.11	male
DS10_00010014		scaffold149:47008-54915	0.08	17.63	male
DS10_00010085		scaffold112:57773-61045	44.46	154.49	male
DS10_00010128		scaffold86:50749-55094	3.35	33.81	male
DS10_00010130		scaffold86:65973-67624	0.00	35.93	male
DS10_00010131		scaffold86:67708-68544	0.05	12.31	male
DS10_00010141		scaffold86:156476-157376	0.00	165.76	male
DS10_00010148		scaffold99:133048-138813	15.01	2.42	female
DS10_00010153	Art6	scaffold99:165086-169152	0.02	13.68	male
DS10_00010158		scaffold99:274727-275110	63.16	0.55	female
DS10_00010214		scaffold305:57576-58276	0.07	12.77	male
DS10_00010225		scaffold126:34865-35894	0.00	13.94	male
DS10_00010229		scaffold126:181406-201708	0.03	37.78	male
DS10_00010232		scaffold126:266725-266904	0.00	2831.16	male
DS10_00010239		scaffold92:93449-100061	1.70	82.34	male
DS10_00010241		scaffold92:103578-107973	6.08	37.10	male
DS10_00010245		scaffold92:128664-132032	0.23	9.70	male
DS10_00010257		scaffold92:190888-194227	93.14	28.20	female
DS10_00010261		scaffold92:222528-222918	0.00	228.06	male
DS10_00010279		scaffold158:241959-243871	5.74	28.71	male
DS10_00010295		scaffold155:124221-128338	2.64	16.10	male
DS10_00010297		scaffold155:132106-133095	0.22	5.78	male
DS10_00010318		scaffold166:84779-86149	0.00	19.23	male
DS10_00010332	asun	scaffold166:220418-228770	0.74	7.61	male
DS10_00010357	CheB93b	scaffold308:40146-40868	0.18	3.41	male
DS10_00010389		scaffold204:57788-60733	0.08	19.63	male
DS10_00010397		scaffold204:173229-174735	1.48	17.05	male
DS10_00010398		scaffold204:175418-176924	1.52	16.56	male
DS10_00010417		scaffold100:38608-42565	0.04	17.45	male
DS10_00010433		scaffold100:237051-241729	6.83	29.23	male

DS10_00010435		scaffold152:256-685	0.31	5.09	male
DS10_00010436		scaffold152:5051-5987	0.09	24.03	male
DS10_00010437		scaffold152:6465-7371	0.03	18.87	male
DS10_00010438		scaffold152:28172-30796	0.03	20.36	male
DS10_00010444		scaffold132:127811-128275	0.00	64.64	male
DS10_00010463	ss	scaffold103:216116-250189	0.52	6.60	male
DS10_00010468	tilB	scaffold108:60888-63128	0.24	10.47	male
DS10_00010474	Cp110	scaffold108:112542-118034	6.07	26.89	male
DS10_00010495	Tim17b1	scaffold241:219783-253951	0.41	7.92	male
DS10_00010509		scaffold247:29491-31267	0.17	63.36	male
DS10_00010512		scaffold247:210881-211214	0.70	12.38	male
DS10_00010513		scaffold247:215241-216739	35.56	5.88	female
DS10_00010514		scaffold247:217044-225020	1.14	8.57	male
DS10_00010516		scaffold247:226350-227016	0.00	45.49	male
DS10_00010523	hale	scaffold110:18923-24373	0.75	25.86	male
DS10_00010524	schuy	scaffold110:18923-24373	0.02	52.81	male
DS10_00010538		scaffold110:171510-180331	0.01	5.03	male
DS10_00010547		scaffold111:26366-26639	0.25	26.83	male
DS10_00010548		scaffold111:28224-28416	0.58	41.39	male
DS10_00010571		scaffold117:186-4405	4.63	21.34	male
DS10_00010589		scaffold121:60997-61498	0.10	5.10	male
DS10_00010617	Hex-t1	scaffold214:12326-17587	1.20	14.46	male
DS10_00010624	DppIII	scaffold124:27724-31377	14.98	52.21	male
DS10_00010628		scaffold124:54019-54559	94.69	0.37	female
DS10_00010634		scaffold124:75126-82708	0.07	2.30	male
DS10_00010646	Mcm2	scaffold124:129465-132187	10.87	1.02	female
DS10_00010650		scaffold124:192074-193976	0.42	4.98	male
DS10_00010658		scaffold127:22357-23736	0.22	7.65	male
DS10_00010674		scaffold127:147778-158181	0.07	29.11	male
DS10_00010745	h-cup	scaffold135:88017-88821	0.05	35.76	male
DS10_00010777		scaffold220:123559-131724	2.12	0.18	female
DS10_00010786		scaffold209:20621-21560	0.05	21.84	male
DS10_00010789		scaffold209:33274-34263	0.06	84.18	male
DS10_00010795		scaffold209:192668-194503	0.00	38.89	male
DS10_00010817		scaffold353:126456-132019	0.40	3.45	male
DS10_00010846		scaffold145:35017-36351	0.12	17.24	male
DS10_00010862		scaffold146:16118-17168	0.04	5.17	male
DS10_00010863		scaffold146:17802-18849	0.00	33.38	male
DS10_00010864		scaffold146:38509-43724	0.02	17.31	male
DS10_00010877		scaffold146:88171-89707	1.04	29.64	male
DS10_00010894		scaffold147:71152-71934	0.09	40.95	male
DS10_00010896		scaffold147:75787-76582	1.77	33.18	male
DS10_00010903		scaffold147:145289-147402	0.07	8.48	male
DS10_00010912		scaffold150:55871-58612	0.03	83.67	male
DS10_00010937		scaffold243:72385-81902	0.00	206.43	male
DS10_00010938		scaffold243:92955-93591	0.00	30.31	male
DS10_00010940		scaffold243:115635-116617	0.00	51.80	male
DS10_00010943		scaffold243:147091-148935	0.02	20.26	male
DS10_00010951		scaffold153:102314-115449	28.54	84.83	male
DS10_00010953	Tim17a2	scaffold153:128317-129013	2.70	21.07	male
DS10_00010973	Rcd7	scaffold205:68609-85074	0.14	35.75	male
DS10_00010987		scaffold205:177808-185547	0.02	15.53	male
DS10_00011005		scaffold260:188950-191898	0.02	22.62	male
DS10_00011021		scaffold185:194484-195549	0.00	16.14	male

DS10_00011064		scaffold404:99539-100135	4.08	26.27	male
DS10_00011093		scaffold315:136162-143950	0.11	2.53	male
DS10_00011105	Ran	scaffold202:70943-71594	2.07	46.04	male
DS10_00011117	DNApol-alpha180	scaffold394:165099-170549	3.97	0.61	female
DS10_00011120		scaffold460:16754-18422	3.09	30.81	male
DS10_00011121		scaffold460:18785-21528	0.02	3.55	male
DS10_00011122		scaffold460:43588-43921	0.68	12.18	male
DS10_00011124	dnd	scaffold460:49477-55370	28.10	5.20	female
DS10_00011125		scaffold460:55678-64535	1.82	8.94	male
DS10_00011128		scaffold460:70326-71402	0.23	81.04	male
DS10_00011129	Cby	scaffold460:71715-72246	0.54	14.21	male
DS10_00011130		scaffold460:72651-75183	0.48	37.37	male
DS10_00011156		scaffold172:147145-149124	0.47	8.78	male
DS10_00011159		scaffold172:151068-154184	0.21	11.36	male
DS10_00011160		scaffold172:161490-163022	0.05	7.87	male
DS10_00011183		scaffold173:157289-170290	0.04	10.46	male
DS10_00011193		scaffold175:65599-65791	1.23	6839.53	male
DS10_00011194		scaffold175:87632-89424	0.03	7.75	male
DS10_00011195	Acam	scaffold175:92458-92905	0.00	64.07	male
DS10_00011196		scaffold175:93055-95026	0.00	19.33	male
DS10_00011197	BG642167	scaffold175:99551-100078	0.00	126.40	male
DS10_00011201		scaffold175:127652-135042	0.04	7.79	male
DS10_00011223	RpS5b	scaffold179:45421-47100	280.31	12.12	female
DS10_00011249		scaffold531:95645-116013	0.14	5.22	male
DS10_00011264		scaffold183:26340-34092	0.03	8.22	male
DS10_00011265		scaffold183:34441-38278	0.10	10.19	male
DS10_00011306	Dic2	scaffold186:78875-80641	0.00	40.66	male
DS10_00011345		scaffold188:90795-91298	0.16	404.53	male
DS10_00011363		scaffold189:47533-58563	1.28	127.89	male
DS10_00011368	Glt	scaffold189:95280-103587	75.50	24.92	female
DS10_00011400		scaffold192:104-1195	0.00	17.74	male
DS10_00011406		scaffold192:49108-53361	1.22	34.08	male
DS10_00011415		scaffold192:103560-104607	9.42	43.20	male
DS10_00011429	Best4	scaffold193:52020-56279	0.00	6.53	male
DS10_00011464		scaffold198:18467-19340	0.05	16.38	male
DS10_00011465		scaffold198:38093-42448	0.00	54.70	male
DS10_00011466		scaffold198:69580-83730	0.00	19.52	male
DS10_00011467	JIL-1	scaffold198:93970-98644	0.01	22.13	male
DS10_00011468		scaffold198:143861-145603	0.00	10.84	male
DS10_00011470		scaffold401:13361-14964	0.03	8.04	male
DS10_00011471	Adgf-A	scaffold401:18611-27049	3.07	25.15	male
DS10_00011472		scaffold401:42569-47553	0.02	2.23	male
DS10_00011505		scaffold199:80392-83215	0.38	5.47	male
DS10_00011513		scaffold199:153186-153594	0.42	33.86	male
DS10_00011521		scaffold368:14731-16662	0.03	6.42	male
DS10_00011546		scaffold206:22465-25339	0.23	2.26	male
DS10_00011560		scaffold206:112529-117782	0.54	3.59	male
DS10_00011566	Acp76A	scaffold267:133551-134850	0.00	14.32	male
DS10_00011608		scaffold215:118375-120155	0.74	30.58	male
DS10_00011633		scaffold227:4908-11149	0.04	39.81	male
DS10_00011635		scaffold227:59208-60018	0.00	97.86	male
DS10_00011651		scaffold221:54198-57285	0.02	45.64	male
DS10_00011660		scaffold221:140042-140549	0.00	92.44	male

DS10_00011668		scaffold259:14594-17355	0.08	20.03	male
DS10_00011675		scaffold259:36185-42587	0.02	3.82	male
DS10_00011676		scaffold259:36185-42587	0.23	5.01	male
DS10_00011677		scaffold259:45776-48302	4.37	0.27	female
DS10_00011695		scaffold229:58519-71572	0.00	8.57	male
DS10_00011703		scaffold405:43498-47428	0.00	25.08	male
DS10_00011709	Tengl4	scaffold232:12655-14530	0.00	17.60	male
DS10_00011742		scaffold447:8126-13029	0.00	4.82	male
DS10_00011759		scaffold425:66629-69379	0.00	11.08	male
DS10_00011787		scaffold328:136706-138148	0.00	29.10	male
DS10_00011797		scaffold439:82749-83331	0.08	4.54	male
DS10_00011802	gogo	scaffold372:35945-49010	0.23	5.18	male
DS10_00011825		scaffold337:74166-74757	0.00	38.67	male
DS10_00011838		scaffold261:25162-25715	0.00	60.60	male
DS10_00011851	tral	scaffold266:39607-45474	87.18	27.58	female
DS10_00011858		scaffold266:70127-74352	1.01	15.45	male
DS10_00011860		scaffold266:82359-85896	0.07	61.24	male
DS10_00011877		scaffold264:50776-63525	0.39	3.02	male
DS10_00011899		scaffold307:5905-6793	0.30	15.83	male
DS10_00011901	nos	scaffold307:28532-38037	8.32	0.63	female
DS10_00011906		scaffold307:93464-94052	0.08	3.93	male
DS10_00011919		scaffold676:107624-108073	0.00	60.52	male
DS10_00011921		scaffold676:124145-125937	0.04	2.23	male
DS10_00011922		scaffold676:126181-131112	0.01	18.70	male
DS10_00011927		scaffold451:44093-48875	0.17	21.15	male
DS10_00011928		scaffold451:49186-55232	6.90	24.59	male
DS10_00011935	Dhc98D	scaffold412:56833-87205	0.87	4.26	male
DS10_00011950	eIF4G2	scaffold275:47653-63367	8.06	61.13	male
DS10_00011959		scaffold325:47024-47749	0.07	25.52	male
DS10_00011966		scaffold325:95966-96404	0.16	21.06	male
DS10_00011983		scaffold383:25949-37005	0.01	31.57	male
DS10_00011990		scaffold383:88906-89422	0.20	5.94	male
DS10_00012026		scaffold283:62036-63367	0.10	125.41	male
DS10_00012029		scaffold284:124392-128640	0.00	31.10	male
DS10_00012031		scaffold285:10168-12872	0.06	58.43	male
DS10_00012042	Pxt	scaffold285:84767-90994	24.01	2.06	female
DS10_00012043	Atg8b	scaffold285:92349-92712	0.43	71.52	male
DS10_00012074	His2Av	scaffold494:108177-108552	45.79	5.21	female
DS10_00012076		scaffold301:31657-33273	0.02	11.51	male
DS10_00012077		scaffold301:53221-54250	0.04	75.49	male
DS10_00012079		scaffold301:83895-85047	0.00	80.69	male
DS10_00012107	alpha-Est6	scaffold310:88129-109380	0.06	2.11	male
DS10_00012114	CcapR	scaffold477:10193-31613	0.10	9.90	male
DS10_00012122		scaffold375:55647-56669	0.70	6.55	male
DS10_00012137		scaffold510:95128-96632	0.02	8.49	male
DS10_00012141	Prosalpha3T	scaffold321:39581-40172	0.04	3.33	male
DS10_00012157	klhl10	scaffold335:110691-112203	0.03	51.71	male
DS10_00012159	djl	scaffold324:105705-111171	0.71	101.15	male
DS10_00012160		scaffold324:112769-114721	0.11	48.43	male
DS10_00012177		scaffold802:113447-114425	0.58	30.03	male
DS10_00012204	AGO3	scaffold653:17919-19443	18.67	0.56	female
DS10_00012224		scaffold511:3407-5307	1.26	26.59	male
DS10_00012239		scaffold379:21082-22225	34.07	0.07	female
DS10_00012244		scaffold379:60765-67481	0.06	21.12	male

DS10_00012264		scaffold668:61040-62380	5.70	88.60	male
DS10_00012290		scaffold787:308-2811	0.00	39.39	male
DS10_00012295		scaffold390:31689-32205	2.51	29.40	male
DS10_00012305		scaffold389:54631-60288	0.02	4.31	male
DS10_00012307		scaffold389:61230-62265	0.01	8.36	male
DS10_00012314		scaffold391:80144-85472	0.01	8.38	male
DS10_00012323		scaffold393:81848-83501	0.00	31.30	male
DS10_00012324		scaffold393:84311-85129	0.06	94.22	male
DS10_00012337		scaffold400:53348-56401	0.00	13.13	male
DS10_00012338	Dic4	scaffold400:58326-62077	0.00	8.82	male
DS10_00012350	Or42a	scaffold402:17057-18278	0.12	5.69	male
DS10_00012359		scaffold406:48537-50101	2.03	0.19	female
DS10_00012364	Pk1r	scaffold529:5477-11876	0.18	14.71	male
DS10_00012374		scaffold529:70756-82267	0.02	48.17	male
DS10_00012375		scaffold529:84331-85318	0.00	47.17	male
DS10_00012412		scaffold449:26301-27202	0.00	78.90	male
DS10_00012414		scaffold449:39340-43890	0.03	26.80	male
DS10_00012415	glob2	scaffold449:72762-73289	0.00	51.29	male
DS10_00012432		scaffold574:12431-15647	0.02	41.69	male
DS10_00012435	Rbp4	scaffold574:34902-37608	0.25	8.31	male
DS10_00012455		scaffold418:63862-65326	0.00	28.58	male
DS10_00012460		scaffold420:65118-70240	0.11	16.52	male
DS10_00012461		scaffold420:70789-82348	0.01	8.23	male
DS10_00012464	FK506-bp1	scaffold419:36920-37991	42.04	8.64	female
DS10_00012470		scaffold422:63544-64865	0.00	45.27	male
DS10_00012471		scaffold422:71398-76936	0.01	20.01	male
DS10_00012485	Task7	scaffold433:40967-44683	3.43	113.01	male
DS10_00012521		scaffold444:48264-48573	0.00	91.47	male
DS10_00012535	WRNexo	scaffold562:59879-61400	5.35	0.51	female
DS10_00012536		scaffold562:61836-63938	0.02	30.16	male
DS10_00012539	qin	scaffold448:61052-69402	6.14	0.45	female
DS10_00012561	topi	scaffold462:7099-8336	0.08	4.56	male
DS10_00012568		scaffold462:33865-36001	0.00	65.41	male
DS10_00012584		scaffold771:61565-62397	0.00	81.77	male
DS10_00012620	rt	scaffold483:11842-33996	0.40	4.74	male
DS10_00012623		scaffold483:50411-63253	0.01	9.25	male
DS10_00012629		scaffold488:34842-42012	0.08	40.64	male
DS10_00012638		scaffold575:39596-40484	0.00	16.30	male
DS10_00012639	nos	scaffold575:54062-54554	3.53	0.11	female
DS10_00012641	nos	scaffold575:55086-66076	5.92	0.10	female
DS10_00012657	Vha14-2	scaffold493:23075-24653	0.03	4.31	male
DS10_00012667		scaffold513:55072-56083	0.16	14.79	male
DS10_00012674	Prosalpha3T	scaffold516:36444-37212	0.03	3.69	male
DS10_00012693	nmdyn-D7	scaffold528:419-1918	1.64	20.14	male
DS10_00012712		scaffold547:44895-45630	0.06	59.07	male
DS10_00012718		scaffold549:37407-56508	0.22	25.63	male
DS10_00012733	Hex-t1	scaffold890:403-3301	0.00	20.61	male
DS10_00012748		scaffold577:54582-56848	0.02	15.56	male
DS10_00012801		scaffold622:32106-33323	0.00	64.56	male
DS10_00012804		scaffold626:53227-55180	4.14	0.12	female
DS10_00012807	Hsc70-2	scaffold979:18630-21046	0.48	29.44	male
DS10_00012813		scaffold642:45051-45360	0.00	90.44	male
DS10_00012843		scaffold1441:14531-17764	0.03	18.33	male
DS10_00012856		scaffold1197:64-846	0.09	46.72	male

DS10_00012864		scaffold674:19065-20462	0.90	8.69	male
DS10_00012868		scaffold674:37017-39783	0.02	3.04	male
DS10_00012875		scaffold677:35247-36753	1.19	19.89	male
DS10_00012876		scaffold677:37036-37417	1.93	30.13	male
DS10_00012886		scaffold687:43528-50012	0.18	18.10	male
DS10_00012896	janA	scaffold705:4050-6369	17.72	163.39	male
DS10_00012913		scaffold722:46066-49669	0.02	19.08	male
DS10_00012918		scaffold955:46501-47476	0.61	108.92	male
DS10_00012926		scaffold724:21745-23307	0.07	14.52	male
DS10_00012933	gdl	scaffold724:39197-40310	4.63	29.71	male
DS10_00012942	Cyp312a1	scaffold729:30252-32755	0.62	26.05	male
DS10_00012949		scaffold849:9062-11927	0.00	120.78	male
DS10_00012972		scaffold763:20057-33894	0.16	3.20	male
DS10_00012973		scaffold763:20057-33894	0.51	4.33	male
DS10_00012974		scaffold763:34534-35713	0.47	6.07	male
DS10_00012980		scaffold769:12520-13465	0.04	35.48	male
DS10_00012988	Mcm5	scaffold1377:42551-45173	9.22	1.12	female
DS10_00013022	sina	scaffold815:25338-32410	4.76	35.15	male
DS10_00013032		scaffold830:25209-39938	0.00	4.57	male
DS10_00013048	lobo	scaffold2221:37881-40458	0.04	4.41	male
DS10_00013098		scaffold949:23337-24267	1.88	59.94	male
DS10_00013099		scaffold949:24394-25969	0.08	55.54	male
DS10_00013104		scaffold957:9361-10390	0.00	25.63	male
DS10_00013105		scaffold957:12042-12900	0.00	31.19	male
DS10_00013137		scaffold1017:31678-32580	0.02	61.91	male
DS10_00013140		scaffold1760:8827-12907	2.48	0.03	female
DS10_00013168		scaffold1078:16475-16862	0.22	60.06	male
DS10_00013176		scaffold1104:6079-6587	0.67	280.96	male
DS10_00013223	PH4alphaNE3	scaffold3067:0-5244	0.10	40.55	male
DS10_00013231	Sfp84E	scaffold1258:12797-15866	0.00	30.93	male
DS10_00013242		scaffold1447:18173-19325	0.00	28.73	male
DS10_00013276		scaffold1556:10-1090	0.06	9.41	male
DS10_00013305		scaffold1675:69-4434	0.06	8.16	male
DS10_00013308		scaffold1674:206-1427	0.00	85.44	male
DS10_00013310	Klp59C	scaffold1674:5083-6519	0.00	31.85	male
DS10_00013311	prt	scaffold1680:1505-3930	0.21	213.76	male
DS10_00013321		scaffold1794:7066-8167	0.04	65.05	male
DS10_00013328		scaffold1812:6077-7264	0.04	35.13	male
DS10_00013353		scaffold2145:5859-6462	0.00	77.46	male
DS10_00013357	Taf12L	scaffold2209:9609-10086	0.39	26.52	male
DS10_00013411	mud	scaffold3377:48-4422	0.24	4.84	male
DS10_00013413		scaffold3315:3817-4960	0.07	11.98	male
DS10_00013423	ssp5	scaffold3454:2-1563	0.00	49.28	male
DS10_00013426		scaffold3474:1985-3173	0.86	10.30	male
DS10_00013434		scaffold3680:660-2317	1.94	181.01	male
DS10_00013452		scaffold3819:27-955	0.25	12.31	male
DS10_00013473		scaffold4415:24-2388	0.34	30.35	male
DS10_00013476		scaffold4528:1763-2301	0.18	43.49	male
DS10_00013487	tomboy20	scaffold5051:1397-1847	0.00	133.65	male
DS10_00013498	dhd	scaffold5800:280-604	174.07	0.69	female
DS10_00013500		scaffold6090:341-2044	33.32	116.29	male
DS10_00013525		scaffold6382:0-1596	0.06	5.63	male
DS10_00013532		scaffold6530:1014-1173	1.13	58.68	male

DS10_00013540	CycA	scaffold6719:606-1038	90.22	17.25	female

Table S6 A list of genes showing sex-biased shift in expression between *Drosophila suzukii* and *Drosophila melanogaster*.

Gene Name D.suz	Location	Female FPKM	Male FPKM	Bias in D.suz	Best Hit in D.mel	Gene Name D.mel	Gene Symbol	Female FPKM	Male FPKM	Bias in D.mel	Shift Pattern (D.suz compare with D.mel)
DS10_00001473	scaffold1:16714274-16723749	0.22	2.61	Male	FBgn0003046	CG3440	Pcp	11.41	0.09	Female	Female-to-Male
DS10_00001542	scaffold1:17685688-17693475	0.01	9.52	Male	FBgn0032082	CG18088		9.84	0.55	Female	Female-to-Male
DS10_00005904	scaffold7:274639-286405	2.10	10.85	Male	FBgn0053172	CG33172		50.21	5.52	Female	Female-to-Male
DS10_00012620	scaffold483:11842-33996	0.40	4.74	Male	FBgn0003292	CG6097	rt	12.82	1.69	Female	Female-to-Male
DS10_00007784	scaffold15:219270-219699	0.12	24.82	Male	FBgn0001197	CG5499	His2Av	428.69	64.77	Female	Female-to-Male
DS10_00007804	scaffold15:510198-553746	2.41	20.09	Male	FBgn0052365	CG32365		24.78	4.22	Female	Female-to-Male
DS10_00011906	scaffold307:93464-94052	0.08	3.93	Male	FBgn0032354	CG4788		20.46	4.34	Female	Female-to-Male
DS10_00009286	scaffold94:238463-242205	2.16	15.00	Male	FBgn0039189	CG18528		12.29	2.72	Female	Female-to-Male
DS10_00008539	scaffold32:271800-276593	2.99	13.74	Male	FBgn0039417	CG6073		25.28	6.14	Female	Female-to-Male
DS10_00004076	scaffold3:5482391-5483522	0.05	11.53	Male	FBgn0031422	CG9870		4.88	1.23	Female	Female-to-Male
DS10_00008025	scaffold20:659406-661955	0.53	4.09	Male	FBgn0035026	CG12252	Fcp1	26.34	7.71	Female	Female-to-Male
DS10_00001375	scaffold1:15913446-15914124	1.22	9.07	Male	FBgn0003607	CG8409	Su(var)205	134.55	41.00	Female	Female-to-Male
DS10_00011093	scaffold315:136162-143950	0.11	2.53	Male	FBgn0052095	CG32095		22.09	7.07	Female	Female-to-Male
DS10_00010332	scaffold166:220418-228770	0.74	7.61	Male	FBgn0020407	CG6814	asun	11.89	3.91	Female	Female-to-Male
DS10_00006394	scaffold11:2182182-2190490	1.77	13.17	Male	FBgn0028974	CG32562	xmas-2	66.40	24.95	Female	Female-to-Male
DS10_00005283	scaffold5:2319840-2321844	7.17	34.95	Male	FBgn0029929	CG4593		221.97	83.58	Female	Female-to-Male
DS10_00009776	scaffold66:145727-151183	11.71	184.51	Male	FBgn0261532	CG7212	cdm	45.60	20.05	Female	Female-to-Male
DS10_00000894	scaffold1:9772060-9772516	0.49	35.85	Male	FBgn0010602	CG3018	lwr	139.93	63.28	Female	Female-to-Male
DS10_00006949	scaffold10:1771336-1774800	3.60	0.25	Female	FBgn0029754	CG15930		2.56	39.23	Male	Male-to-Female
DS10_00008650	scaffold123:305153-314429	24.40	7.06	Female	FBgn0026314	CG6649	Ugt35b	17.43	66.12	Male	Male-to-Female
DS10_00008326	scaffold29:347519-351614	4.19	0.13	Female	FBgn0039342	CG5107		151.04	438.57	Male	Male-to-Female
DS10_00002942	scaffold2:8639392-8648490	11.98	1.76	Female	FBgn0034887	CG5428	St1	8.82	18.02	Male	Male-to-Female

Table S7 GO term and functional classification enrichment analysis using DAVID for gene families that are expanded in the *Drosophila suzukii* genome as compared to 14 *Drosophila* species analyzed in this study.

CATEGORY[a]	TERM	COUNT[b]	P VALUE	GENE FAMILIES: REPRESENTATIVE GENE SYMBOLS[c]
**Annotation Cluster 1**	**Enrichment Score: 1.8103805538093147**			
GOTERM BP	GO:0006071 glycerol metabolic process	3	8.49E-03	Gpdh, CG18135, Gyk
GOTERM BP	GO:0019400 alditol metabolic process	3	8.49E-03	Gpdh, CG18135, Gyk
GOTERM BP	GO:0019751 polyol metabolic process	3	5.14E-02	Gpdh, CG18135, Gyk
**Annotation Cluster 2**	**Enrichment Score: 1.7459845947230437**			
GOTERM BP	GO:0032268 regulation of cellular protein metabolic process	9	3.85E-03	Acp76A, PEK, Adam, mRpL11, Tollo, Spn77Bc, Su(var)205, nos, elav
GOTERM BP	GO:0010605 negative regulation of macromolecule metabolic process	13	4.11E-03	WRNexo, Acp76A, mRpL11, Spn77Bc, Su(var)205, elav, mael, Hsc70-4, PEK, E(spl)m8-HLH, fkh, lolal, nos
GOTERM BP	GO:0032269 negative regulation of cellular protein metabolic process	5	1.36E-02	Acp76A, PEK, Spn77Bc, nos, elav
GOTERM BP	GO:0051248 negative regulation of protein metabolic process	5	1.49E-02	Acp76A, PEK, Spn77Bc, nos, elav
SWISSPROT PIR	translation regulation	3	4.00E-02	PEK, mRpL11, nos

[a] Categories used in DAVID analysis include GO sub-ontologies for Biological Process (BP), Molecular Function (MF), and CC (Cellular Component), as well as keywords from SwissProt PIR (Protein Information Resource) and protein domains from the Interpro database.

[b] Count represents the number of gene families

[c] Gene symbol for representative, most highly annotated member of each gene family is shown. All annotations shown here are from *D. melanogaster*.

GOTERM BP	GO:0006417 regulation of translation	5	4.84E-02	PEK, Adam, mRpL11, nos, elav
GOTERM BP	GO:0010608 posttranscriptional regulation of gene expression	6	9.66E-02	Hsc70-4, PEK, Adam, mRpL11, nos, elav
**Annotation Cluster 3**	**Enrichment Score: 1.5795892033529708**			
SWISSPROT PIR	Chaperone	5	1.54E-02	CG7394, CG11267, Cnx99A, Tcp-1zeta
GOTERM BP	GO:0006457 protein folding	6	2.69E-02	Hsc70-4, CG11267, Cnx99A, Tcp-1zeta, FKBP59
GOTERM MF	GO:0051082 unfolded protein binding	5	4.40E-02	Hsc70-4, CG11267, Cnx99A, Tcp-1zeta
**Annotation Cluster 4**	**Enrichment Score: 1.420233335387723**			
GOTERM BP	GO:0051606 detection of stimulus	6	1.26E-02	Or49a, Calx, CheB42a, Galphaq, FKBP59
SWISSPROT PIR	sensory transduction	6	3.13E-02	Or49a, Or69a, Galphaq, FKBP59, Or22a
GOTERM BP	GO:0009583 detection of light stimulus	4	4.32E-02	Calx, Galphaq, FKBP59
GOTERM BP	GO:0009582 detection of abiotic stimulus	4	6.20E-02	Calx, Galphaq, FKBP59
GOTERM BP	GO:0009581 detection of external stimulus	4	7.48E-02	Calx, Galphaq, FKBP59
**Annotation Cluster 5**	**Enrichment Score: 1.3722382305056715**			
INTERPRO	IPR007087:Zinc finger, C2H2-type	13	2.49E-02	CG6689, term, CG1647, CG16779, CG10669, Meics, CG5316, CG4360, CG3065, CG11966, CG4318
INTERPRO	IPR015880:Zinc finger, C2H2-like	13	2.93E-02	CG6689, CG1647, CG16779, CG10669, Meics, CG5316, CG4360, CG3065, CG11966, noi, CG4318

INTERPRO	IPR012934:Zinc finger, AD-type	5	4.66E-02	CG6689, CG1647, CG10669, Meics, CG4318
INTERPRO	IPR013087:Zinc finger, C2H2-type/integrase, DNA-binding	7	4.70E-02	CG11966, CG6689, CG16779, CG10669, Meics, CG4360, CG3065
SMART	SM00355:ZnF_C2H2	13	8.63E-02	CG6689, CG1647, CG16779, CG10669, Meics, CG5316, CG4360, CG3065, CG11966, noi, CG4318
**Annotation Cluster 6**	**Enrichment Score: 1.2224866596583404**			
SWISSPROT PIR	sensory transduction	6	3.13E-02	Or49a, Or69a, Galphaq, FKBP59, Or22a
GOTERM MF	GO:0005549 odorant binding	6	3.17E-02	Obp83ef, Or49a, Obp51a, CheB42a, Or69a, Or22a
GOTERM BP	GO:0007186 G-protein coupled receptor protein signaling pathway	10	4.14E-02	Or49a, Tk, D2R, Or69a, Galphaq, Gr85a, mth, Pk1r, Or22a
SWISSPROT PIR	transducer	8	4.29E-02	Or49a, D2R, Or69a, Galphaq, Gr85a, mth, Pk1r, Or22a
GOTERM BP	GO:0007600 sensory perception	9	5.60E-02	Obp83ef, Or49a, Obp51a, Or69a, Galphaq, Gr85a, FKBP59, Or22a
SWISSPROT PIR	g-protein coupled receptor	7	8.04E-02	Or49a, D2R, Or69a, Gr85a, mth, Pk1r, Or22a
INTERPRO	IPR004117:Olfactory receptor, Drosophila	3	8.44E-02	Or49a, Or69a, Or22a
GOTERM CC	GO:0005886 plasma membrane	17	9.45E-02	D2R, Tollo, Or69a, Gr85a, Rac1, Cnx99A, Cad74A, Oscp, Pk1r, Mical, Syn1, Or49a, Calx, Galphaq, mth, FKBP59, Or22a
PIR_SUPERFAMILY	PIRSF008678:odorant receptor	3	9.64E-02	Or49a, Or69a, Or22a
GOTERM BP	GO:0050877 neurological system process	14	9.76E-02	Obp83ef, scramb1, Obp51a, cher, Or69a, Gr85a, Rac1, Hsc70-4, Or49a, Galphaq, mth, FKBP59, Or22a

**Annotation Cluster 7**	**Enrichment Score: 1.1978454386369843**			
SWISSPROT PIR	mitochondrion inner membrane	5	1.41E-02	CG7394, ATPsyn-d, ATPsyn-b, Oscp, CG9603
GOTERM CC	GO:0005753 mitochondrial proton-transporting ATP synthase complex	3	5.02E-02	ATPsyn-d, ATPsyn-b, Oscp
SWISSPROT PIR	Hydrogen ion transport	3	5.21E-02	ATPsyn-d, ATPsyn-b, Oscp
GOTERM BP	GO:0009165 nucleotide biosynthetic process	6	5.54E-02	ATPsyn-d, pug, CG6767, Ada, ATPsyn-b, Oscp
GOTERM BP	GO:0009260 ribonucleotide biosynthetic process	5	5.67E-02	ATPsyn-d, CG6767, Ada, ATPsyn-b, Oscp
GOTERM BP	GO:0009259 ribonucleotide metabolic process	5	6.27E-02	ATPsyn-d, CG6767, Ada, ATPsyn-b, Oscp
GOTERM CC	GO:0045259 proton-transporting ATP synthase complex	3	6.31E-02	ATPsyn-d, ATPsyn-b, Oscp
GOTERM BP	GO:0034404 nucleobase, nucleoside and nucleotide biosynthetic process	6	7.04E-02	ATPsyn-d, pug, CG6767, Ada, ATPsyn-b, Oscp
GOTERM BP	GO:0034654 nucleobase, nucleoside, nucleotide and nucleic acid biosynthetic process	6	7.04E-02	ATPsyn-d, pug, CG6767, Ada, ATPsyn-b, Oscp
GOTERM BP	GO:0044271 nitrogen compound biosynthetic process	8	7.82E-02	ATPsyn-d, CG11899, pug, CG6767, Ada, ATPsyn-b, Odc1, Oscp

GOTERM BP	GO:0006164	purine nucleotide biosynthetic process	5	8.25E-02	ATPsyn-d, pug, Ada, ATPsyn-b, Oscp
GOTERM CC	GO:0044455	mitochondrial membrane part	6	8.29E-02	CG7394, ATPsyn-d, CG4769, ATPsyn-b, Oscp, CG9603
GOTERM BP	GO:0006163	purine nucleotide metabolic process	5	9.34E-02	ATPsyn-d, pug, Ada, ATPsyn-b, Oscp
GOTERM CC	GO:0044429	mitochondrial part	12	9.70E-02	CG7394, ATPsyn-d, mRpL11, CG4769, ATPsyn-b, Hmt-1, Dic1, CG11267, CG9547, mRpL20, Oscp, CG9603
GOTERM CC	GO:0005743	mitochondrial inner membrane	7	9.76E-02	CG7394, ATPsyn-d, CG4769, ATPsyn-b, Hmt-1, Oscp, CG9603
**Annotation Cluster 8**	**Enrichment Score: 1.1438990309544648**				
GOTERM MF	GO:0004867	serine-type endopeptidase inhibitor activity	4	4.68E-02	Acp76A, CG31515, Spn77Bc, CG3604
GOTERM MF	GO:0004866	endopeptidase inhibitor activity	4	8.08E-02	Acp76A, CG31515, Spn77Bc, CG3604
GOTERM MF	GO:0030414	peptidase inhibitor activity	4	9.79E-02	Acp76A, CG31515, Spn77Bc, CG3604

Table S8 GO term and functional classification enrichment analysis using DAVID for gene families that are expanded in the *Drosophila suzukii* genome as compared to the basal paraphyletic group of Drosophilidae including *D. ananassae, D. persimilis, D. pseudoobscura, D. willistoni, D. grimshawi, D. mojavensis*, and *D. virulis*.

CATEGORY	TERM	COUNT	P VALUE	GENE FAMILIES: REPRESENTATIVE GENE SYMBOLS
**Annotation Cluster 1**	**Enrichment Score: 2.3163292177655386**			
GOTERM BP	GO:0032269 negative regulation of cellular protein metabolic process	7	4.18E-04	Acp76A, PEK, Acp62F, Spn77Bc, Spn27A, nos, elav
GOTERM BP	GO:0051248 negative regulation of protein metabolic process	7	4.83E-04	Acp76A, PEK, Acp62F, Spn77Bc, Spn27A, nos, elav
GOTERM BP	GO:0045861 negative regulation of proteolysis	4	9.18E-04	Acp76A, Acp62F, Spn77Bc, Spn27A
GOTERM MF	GO:0004867 serine-type endopeptidase inhibitor activity	6	1.50E-03	Acp76A, CG31515, Acp62F, Spn77Bc, CG3604, Spn27A
GOTERM BP	GO:0032268 regulation of cellular protein metabolic process	10	1.56E-03	Acp76A, PEK, Acp62F, Adam, mRpL11, Tollo, Spn77Bc, Spn27A, nos, elav
GOTERM MF	GO:0004866 endopeptidase inhibitor activity	6	4.15E-03	Acp76A, CG31515, Acp62F, Spn77Bc, CG3604, Spn27A
GOTERM MF	GO:0030414 peptidase inhibitor activity	6	5.96E-03	Acp76A, CG31515, Acp62F, Spn77Bc, CG3604, Spn27A
GOTERM BP	GO:0010605 negative regulation of macromolecule metabolic process	13	7.20E-03	WRNexo, Acp76A, Acp62F, mRpL11, Spn77Bc, Spn27A, elav, Hsc70-4, PEK, E(spl)m8-HLH, fkh, lolal, nos
GOTERM MF	GO:0004857 enzyme inhibitor	6	2.91E-02	Acp76A, CG31515, Acp62F, Spn77Bc, CG3604, Spn27A

	activity			
INTERPRO	IPR000215:Protease inhibitor I4, serpin	3	3.64E-02	Acp76A, Spn77Bc, Spn27A
GOTERM BP	GO:0030162 regulation of proteolysis	4	4.37E-02	Acp76A, Acp62F, Spn77Bc, Spn27A
SMART	SM00093:SERPIN	3	4.49E-02	Acp76A, Spn77Bc, Spn27A
**Annotation Cluster 2**	**Enrichment Score: 2.268060950272713**			
GOTERM BP	GO:0006952 defense response	9	2.76E-03	CG6168, Sr-CI, Tollo, Drs, Lectin-galC1, TotA, TotM, Spn27A, GNBP1
GOTERM BP	GO:0006955 immune response	9	3.16E-03	Sr-CI, Tollo, Drs, Lectin-galC1, Rac1, TotA, TotM, Spn27A, GNBP1
GOTERM BP	GO:0042742 defense response to bacterium	6	3.74E-03	CG6168, Sr-CI, Drs, Lectin-galC1, TotA, GNBP1
GOTERM BP	GO:0009617 response to bacterium	6	8.23E-03	CG6168, Sr-CI, Drs, Lectin-galC1, TotA, GNBP1
GOTERM BP	GO:0050829 defense response to Gram-negative bacterium	4	1.70E-02	CG6168, Sr-CI, Drs, GNBP1
**Annotation Cluster 3**	**Enrichment Score: 1.875257851646743**			
UP_SEQ_FEATURE	glycosylation site:N-linked (GlcNAc...)	17	2.24E-03	Acp76A, Acp29AB, D2R, Gr59c, Mal-A2, Gr85a, TotA, Hsc70-4, PEK, scb, y, pgant3, Drs, CG32669, rt, mth, GNBP1
SWISSPROT PIR	glycoprotein	17	9.71E-03	Acp76A, Acp29AB, D2R, Gr59c, Mal-A2, Gr85a, TotA, Hsc70-4, PEK, scb, y, pgant3, Drs, CG32669, rt, mth, GNBP1

UP_SEQ_FEATURE	signal peptide	15	1.13E-02	Acp76A, Acp62F, Acp29AB, Mal-A2, TotA, Gpb5, Sgs3, PEK, scb, y, Tk, Drs, TotM, mth, GNBP1
GOTERM CC	GO:0005576 extracellular region	16	1.88E-02	Acp76A, Acp62F, Acp29AB, CheB42a, CG34049, cher, TotA, Gpb5, Spn27A, Sgs3, Tk, y, beat-Va, Drs, TotM, GNBP1
SWISSPROT PIR	signal	15	2.78E-02	Acp76A, Acp62F, Acp29AB, Mal-A2, TotA, Gpb5, Sgs3, PEK, scb, y, Tk, Drs, TotM, mth, GNBP1
SWISSPROT PIR	Secreted	9	4.34E-02	Sgs3, Acp76A, Acp62F, y, Tk, Acp29AB, Drs, TotA, TotM
**Annotation Cluster 4**	**Enrichment Score: 1.7532690160826376**			
GOTERM BP	GO:0006071 glycerol metabolic process	3	9.72E-03	Gpdh, CG18135, Gyk
GOTERM BP	GO:0019400 alditol metabolic process	3	9.72E-03	Gpdh, CG18135, Gyk
GOTERM BP	GO:0019751 polyol metabolic process	3	5.82E-02	Gpdh, CG18135, Gyk
**Annotation Cluster 5**	**Enrichment Score: 1.5779149847475604**			
GOTERM BP	GO:0045087 innate immune response	6	1.24E-02	Tollo, Drs, TotA, TotM, Spn27A, GNBP1
GOTERM BP	GO:0050832 defense response to fungus	3	3.85E-02	Drs, Spn27A, GNBP1

GOTERM BP	GO:0009620 response to fungus	3	3.85E-02	Drs, Spn27A, GNBP1
**Annotation Cluster 6**	**Enrichment Score: 1.4342861994852356**			
SWISSPROT PIR	Chaperone	5	2.67E-02	CG7394, CG11267, Cnx99A, Tcp-1zeta
GOTERM BP	GO:0006457 protein folding	6	3.49E-02	Hsc70-4, CG11267, Cnx99A, Tcp-1zeta, FKBP59
GOTERM MF	GO:0051082 unfolded protein binding	5	5.34E-02	Hsc70-4, CG11267, Cnx99A, Tcp-1zeta
**Annotation Cluster 7**	**Enrichment Score: 1.420967720788353**			
SWISSPROT PIR	calcium	8	1.08E-02	CG4733, CG4662, pgant3, CG17271, Cad74A, sunz, Tctp
GOTERM MF	GO:0005509 calcium ion binding	10	2.82E-02	CG4733, CG4662, pgant3, Cnx99A, CG17271, Cad74A, CG42255, sunz, Tctp
INTERPRO	IPR018249:EF-HAND 2	5	4.46E-02	CG4733, CG4662, CG17271, sunz
INTERPRO	IPR011992:EF-Hand type	5	6.38E-02	CG4733, CG4662, CG17271, sunz
INTERPRO	IPR018247:EF-HAND 1	5	9.08E-02	CG4733, CG4662, CG17271, sunz
**Annotation Cluster 8**	**Enrichment Score: 1.2794935055644372**			
GOTERM BP	GO:0051606 detection of stimulus	6	1.67E-02	Or49a, Calx, CheB42a, Galphaq, FKBP59
GOTERM BP	GO:0009628 response to abiotic stimulus	8	3.30E-02	shep, Calx, Galphaq, TotA, TotM, mth, FKBP59
GOTERM BP	GO:0009583 detection of light	4	5.14E-02	Calx, Galphaq, FKBP59

	stimulus			
GOTERM BP	GO:0009416 response to light stimulus	5	6.29E-02	Calx, Galphaq, TotA, FKBP59
GOTERM BP	GO:0009582 detection of abiotic stimulus	4	7.32E-02	Calx, Galphaq, FKBP59
GOTERM BP	GO:0009581 detection of external stimulus	4	8.80E-02	Calx, Galphaq, FKBP59
GOTERM BP	GO:0009314 response to radiation	5	9.62E-02	Calx, Galphaq, TotA, FKBP59
**Annotation Cluster 9**	**Enrichment Score: 1.211795352683188**			
INTERPRO	IPR007087:Zinc finger, C2H2-type	13	4.40E-02	CG6689, term, CG1647, CG16779, CG10669, Meics, CG5316, CG4360, CG3065, CG11966, CG4318
INTERPRO	IPR015880:Zinc finger, C2H2-like	13	5.12E-02	CG6689, CG1647, CG16779, CG10669, Meics, CG5316, CG4360, CG3065, CG11966, noi, CG4318
INTERPRO	IPR012934:Zinc finger, AD-type	5	6.03E-02	CG6689, CG1647, CG10669, Meics, CG4318
INTERPRO	IPR013087:Zinc finger, C2H2-type/integrase, DNA-binding	7	6.59E-02	CG11966, CG6689, CG16779, CG10669, Meics, CG4360, CG3065
SMART	SM00355:ZnF_C2H2	13	9.73E-02	CG6689, CG1647, CG16779, CG10669, Meics, CG5316, CG4360, CG3065, CG11966, noi, CG4318
**Annotation Cluster 10**	**Enrichment Score: 1.2042724915072422**			

SWISSPROT PIR	mitochondrion inner membrane	5	2.45E-02	CG7394, ATPsyn-d, ATPsyn-b, Oscp, CG9603
GOTERM BP	GO:0055085 transmembrane transport	5	6.29E-02	CG7394, ATPsyn-d, ATPsyn-b, CG32669, Oscp
GOTERM CC	GO:0005753 mitochondrial proton-transporting ATP synthase complex	3	6.36E-02	ATPsyn-d, ATPsyn-b, Oscp
GOTERM CC	GO:0005743 mitochondrial inner membrane	8	6.97E-02	CG7394, ATPsyn-d, CG5265, CG4769, ATPsyn-b, Hmt-1, Oscp, CG9603
SWISSPROT PIR	Hydrogen ion transport	3	7.01E-02	ATPsyn-d, ATPsyn-b, Oscp
GOTERM CC	GO:0045259 proton-transporting ATP synthase complex	3	7.95E-02	ATPsyn-d, ATPsyn-b, Oscp
GOTERM CC	GO:0005740 mitochondrial envelope	9	9.75E-02	CG7394, ATPsyn-d, CG5265, CG4769, ATPsyn-b, Hmt-1, Dic1, Oscp, CG9603
**Annotation Cluster 11**	**Enrichment Score: 1.1591583013355033**			
GOTERM CC	GO:0005886 plasma membrane	20	5.15E-02	D2R, Tollo, Gr59c, Or69a, Gr85a, Cnx99A, Rac1, Cad74A, Oscp, Pk1r, Mical, Syn1, Sr-CI, scb, Or49a, Calx, Galphaq, mth, FKBP59, GNBP1
GOTERM BP	GO:0007186 G-protein coupled receptor protein signaling pathway	10	5.98E-02	Or49a, Tk, D2R, Gr59c, Or69a, Galphaq, Gr85a, mth, Pk1r
GOTERM BP	GO:0050890 cognition	11	7.63E-02	Obp83ef, scb, Or49a, Obp51a, cher, Gr59c, Or69a, Galphaq, Gr85a, FKBP59

GOTERM BP	GO:0007600 sensory perception	9	7.76E-02	Obp83ef, Or49a, Obp51a, Gr59c, Or69a, Galphaq, Gr85a, FKBP59
SWISSPROT PIR	transducer	8	8.78E-02	Or49a, D2R, Gr59c, Or69a, Galphaq, Gr85a, mth, Pk1r
**Annotation Cluster 12**	**Enrichment Score: 1.145383017959024**			
GOTERM MF	GO:0043169 cation binding	44	5.96E-02	CG6689, term, CHORD, CG9715, CG10669, CG4769, CG10916, CG5316, MICAL-like, Irk2, Cnx99A, CG17271, Cad74A, Mical, sunz, CG3065, CG11966, mud, CG4318, CG4662, CG31019, pgant3, CG33552, CG32669, Tctp, CG1647, CG16779, Meics, CG4733, x16, Mal-A2, CG4360, alph, noi, Mcm2, CD98hc, Arc1, CG42255, CG5292, nos, SF1
GOTERM MF	GO:0043167 ion binding	44	6.26E-02	CG6689, term, CHORD, CG9715, CG10669, CG4769, CG10916, CG5316, MICAL-like, Irk2, Cnx99A, CG17271, Cad74A, Mical, sunz, CG3065, CG11966, mud, CG4318, CG4662, CG31019, pgant3, CG33552, CG32669, Tctp, CG1647, CG16779, Meics, CG4733, x16, Mal-A2, CG4360, alph, noi, Mcm2, CD98hc, Arc1, CG42255, CG5292, nos, SF1
GOTERM MF	GO:0046872 metal ion binding	42	9.82E-02	CG6689, term, CHORD, CG9715, CG10669, CG4769, CG10916, CG5316, MICAL-like, Irk2, Cnx99A, CG17271, Cad74A, Mical, sunz, CG3065, CG11966, mud, CG4318, CG4662, CG31019, pgant3, CG33552, CG32669, Tctp, CG1647, CG16779, Meics, CG4733, x16, CG4360, alph, noi, Mcm2, Arc1, CG42255, CG5292, nos, SF1

Table S9 GO term and functional classification enrichment analysis using DAVID for gene families that are expanded in the *Drosophila suzukii* genome as compared to species in the melanogaster subgroup, including *D. simulans, D. sechellia, D. yakuba, D. erecta, and D. melanogaster*.

CATEGORY	TERM	COUNT	P VALUE	GENE FAMILIES: REPRESENTATIVE GENE SYMBOLS
**Annotation Cluster 1**	**Enrichment Score: 1.840605394222266**			
GOTERM BP	GO:0006071 glycerol metabolic process	3	7.90E-03	Gpdh, CG18135, Gyk
GOTERM BP	GO:0019400 alditol metabolic process	3	7.90E-03	Gpdh, CG18135, Gyk
GOTERM BP	GO:0019751 polyol metabolic process	3	4.81E-02	Gpdh, CG18135, Gyk
**Annotation Cluster 2**	**Enrichment Score: 1.4947053163114195**			
GOTERM BP	GO:0010605 negative regulation of macromolecule metabolic process	13	3.03E-03	WRNexo, mRpL11, Spn77Bc, Su(var)205, aub, elav, mael, Hsc70-4, PEK, E(spl)m8-HLH, fkh, lolal, nos
GOTERM BP	GO:0032268 regulation of cellular protein metabolic process	9	3.07E-03	PEK, Adam, mRpL11, Tollo, Spn77Bc, aub, Su(var)205, nos, elav
GOTERM BP	GO:0006417 regulation of translation	6	1.01E-02	PEK, Adam, mRpL11, aub, nos, elav
GOTERM BP	GO:0006446 regulation of translational initiation	3	2.68E-02	PEK, Adam, aub
GOTERM BP	GO:0016458 gene silencing	6	2.90E-02	mael, Hsc70-4, mRpL11, aub, Su(var)205, lolal
GOTERM BP	GO:0010608 posttranscriptional regulation of gene expression	7	2.96E-02	Hsc70-4, PEK, Adam, mRpL11, aub, nos, elav
GOTERM BP	GO:0010629 negative regulation of	9	3.45E-02	mael, Hsc70-4, E(spl)m8-HLH, mRpL11, fkh, aub, Su(var)205, lolal,

	gene expression			nos
SWISSPROT PIR	translation regulation	3	3.66E-02	PEK, mRpL11, nos
GOTERM BP	GO:0031047 gene silencing by RNA	4	4.60E-02	mael, Hsc70-4, mRpL11, aub
GOTERM BP	GO:0032269 negative regulation of cellular protein metabolic process	4	6.06E-02	PEK, Spn77Bc, nos, elav
GOTERM BP	GO:0010558 negative regulation of macromolecule biosynthetic process	8	6.32E-02	E(spl)m8-HLH, PEK, fkh, aub, Su(var)205, lolal, nos, elav
GOTERM BP	GO:0051248 negative regulation of protein metabolic process	4	6.45E-02	PEK, Spn77Bc, nos, elav
GOTERM BP	GO:0009890 negative regulation of biosynthetic process	8	6.89E-02	E(spl)m8-HLH, PEK, fkh, aub, Su(var)205, lolal, nos, elav
GOTERM BP	GO:0031327 negative regulation of cellular biosynthetic process	8	6.89E-02	E(spl)m8-HLH, PEK, fkh, aub, Su(var)205, lolal, nos, elav
GOTERM BP	GO:0051172 negative regulation of nitrogen compound metabolic process	7	9.07E-02	WRNexo, E(spl)m8-HLH, fkh, aub, Su(var)205, lolal, nos
GOTERM BP	GO:0045934 negative regulation of nucleobase, nucleoside, nucleotide and nucleic acid metabolic process	7	9.07E-02	WRNexo, E(spl)m8-HLH, fkh, aub, Su(var)205, lolal, nos
**Annotation Cluster 3**	**Enrichment Score: 1.3768919446351504**			
INTERPRO	IPR013087:Zinc finger, C2H2-	7	3.95E-02	CG11966, CG6689, CG16779, CG10669, Meics, CG4360, CG3065

	type/integrase, DNA-binding			
INTERPRO	IPR012934:Zinc finger, AD-type	5	4.08E-02	CG6689, CG1647, CG10669, Meics, CG4318
INTERPRO	IPR007087:Zinc finger, C2H2-type	12	4.09E-02	CG11966, term, CG6689, CG1647, CG16779, CG10669, Meics, CG4318, CG5316, CG4360, CG3065
INTERPRO	IPR015880:Zinc finger, C2H2-like	12	4.72E-02	CG11966, CG6689, CG1647, CG16779, CG10669, Meics, noi, CG4318, CG5316, CG4360, CG3065
**Annotation Cluster 4**	**Enrichment Score: 1.3356641246312047**			
GOTERM BP	GO:0007314 oocyte anterior/posterior axis specification	5	2.30E-02	mael, Tm1, lkb1, aub, nos
GOTERM BP	GO:0007316 pole plasm RNA localization	4	2.53E-02	mael, Tm1, lkb1, aub
GOTERM BP	GO:0019094 pole plasm mRNA localization	4	2.53E-02	mael, Tm1, lkb1, aub
GOTERM BP	GO:0008358 maternal determination of anterior/posterior axis, embryo	5	2.64E-02	mael, Tm1, lkb1, aub, nos
GOTERM BP	GO:0007315 pole plasm assembly	4	3.94E-02	mael, Tm1, lkb1, aub
GOTERM CC	GO:0045495 pole plasm	3	4.31E-02	mael, aub, nos
GOTERM BP	GO:0009994 oocyte differentiation	6	5.08E-02	mael, asp, Tm1, lkb1, aub, nos
GOTERM BP	GO:0007028 cytoplasm organization	4	5.67E-02	mael, Tm1, lkb1, aub
GOTERM BP	GO:0007281 germ cell development	8	6.32E-02	mael, asp, Tm1, Klp64D, lkb1, aub, nos, elav

GOTERM BP	GO:0008298	intracellular mRNA localization	4	7.28E-02	mael, Tm1, lkb1, aub
GOTERM BP	GO:0017145	stem cell division	4	7.28E-02	asp, lkb1, aub, nos
GOTERM BP	GO:0007309	oocyte axis specification	5	7.75E-02	mael, Tm1, lkb1, aub, nos
GOTERM BP	GO:0007308	oocyte construction	5	8.77E-02	mael, Tm1, lkb1, aub, nos
**Annotation Cluster 5**	**Enrichment Score: 1.2102180736678618**				
GOTERM MF	GO:0043169	cation binding	41	5.03E-02	CG6689, term, CHORD, CG9715, CG10669, CG4769, CG10916, CG5316, LpR1, Irk2, CG17271, Cad74A, Mical, CG3065, CG11966, CG4318, CG4662, CG31019, pgant3, CG33552, Tctp, Tim13, CG1647, CG16779, Meics, CG4733, x16, Mal-A2, CG4360, alph, noi, Mcm2, CG6767, CD98hc, Arc1, CG42255, CG5292, nos, SF1
GOTERM MF	GO:0043167	ion binding	41	5.29E-02	CG6689, term, CHORD, CG9715, CG10669, CG4769, CG10916, CG5316, LpR1, Irk2, CG17271, Cad74A, Mical, CG3065, CG11966, CG4318, CG4662, CG31019, pgant3, CG33552, Tctp, Tim13, CG1647, CG16779, Meics, CG4733, x16, Mal-A2, CG4360, alph, noi, Mcm2, CG6767, CD98hc, Arc1, CG42255, CG5292, nos, SF1
GOTERM MF	GO:0046872	metal ion binding	39	8.79E-02	CG6689, term, CHORD, CG9715, CG10669, CG4769, CG10916, CG5316, LpR1, Irk2, CG17271, Cad74A, Mical, CG3065, CG11966, CG4318, CG4662, CG31019, pgant3, CG33552, Tctp, Tim13, CG1647, CG16779, Meics, CG4733, x16, CG4360, alph, noi, Mcm2, CG6767,

Arc1, CG42255, CG5292, nos, SF1

Table S10 GO term and functional classification enrichment analysis using DAVID for gene families that are contracted in the *Drosophila suzukii* genome as compared to 14 *Drosophila* species analyzed in this study.

CATEGORY	TERM	COUNT	P VALUE	GENE FAMILIES: REPRESENTATIVE GENE SYMBOLS
**Annotation Cluster 1**	**Enrichment Score: 3.023489574033364**			
GOTERM CC	GO:0000786 nucleosome	5	8.14E-06	His2B:CG33882, His2Av, His1:CG33834, His4r, His3.3A
SWISSPROT PIR	acetylation	5	2.48E-05	His2Av, Adh, Cam, His4r, His3.3A
SWISSPROT PIR	nucleosome core	4	2.71E-05	His2B:CG33882, His2Av, His4r, His3.3A
INTERPRO	IPR007125:Histone core	4	8.38E-05	His2B:CG33882, His2Av, His4r, His3.3A
GOTERM BP	GO:0006334 nucleosome assembly	5	9.51E-05	His2B:CG33882, His2Av, His1:CG33834, His4r, His3.3A
GOTERM BP	GO:0031497 chromatin assembly	5	3.55E-04	His2B:CG33882, His2Av, His1:CG33834, His4r, His3.3A
SWISSPROT PIR	chromosomal protein	5	7.88E-04	His2B:CG33882, His2Av, His1:CG33834, His4r, His3.3A
GOTERM BP	GO:0006323 DNA packaging	6	8.32E-04	eIF-4E, His2B:CG33882, His2Av, His1:CG33834, His4r, His3.3A
GOTERM BP	GO:0065004 protein-DNA complex assembly	5	9.25E-04	His2B:CG33882, His2Av, His1:CG33834, His4r, His3.3A
GOTERM CC	GO:0032993 protein-DNA complex	5	9.82E-04	His2B:CG33882, His2Av, His1:CG33834, His4r, His3.3A
GOTERM BP	GO:0034728 nucleosome organization	5	1.06E-03	His2B:CG33882, His2Av, His1:CG33834, His4r, His3.3A
GOTERM BP	GO:0006333 chromatin assembly or disassembly	5	2.96E-03	His2B:CG33882, His2Av, His1:CG33834, His4r, His3.3A
INTERPRO	IPR009072:Histone-fold	4	3.47E-03	His2B:CG33882, His2Av, His4r, His3.3A
GOTERM BP	GO:0034621 cellular macromolecular	7	2.60E-02	His2B:CG33882, His2Av, His1:CG33834, gammaTub23C, Cam, His4r,

	complex subunit organization			His3.3A
GOTERM BP	GO:0034622 cellular macromolecular complex assembly	6	3.27E-02	His2B:CG33882, His2Av, His1:CG33834, gammaTub23C, His4r, His3.3A
GOTERM BP	GO:0006325 chromatin organization	6	6.55E-02	eIF-4E, His2B:CG33882, His2Av, His1:CG33834, His4r, His3.3A
GOTERM CC	GO:0000785 chromatin	5	7.12E-02	His2B:CG33882, His2Av, His1:CG33834, His4r, His3.3A
**Annotation Cluster 2**	**Enrichment Score: 2.5911442503164333**			
SWISSPROT PIR	hydrolase	26	4.48E-06	CG1637, CG31821, S-Lap7, CG9449, Ace, CG30049, CG5731, Ance, PGRP-SA, CG14022, CG9391, ApepP, primo-2, CG14034, SPE, cathD, kraken, mag, CG2680, CG42264, CG31272, gd, LysC, Mdr49, CG6465, sda
GOTERM MF	GO:0008238 exopeptidase activity	6	3.74E-04	CG31821, ApepP, CG42264, S-Lap7, Ance, sda
GOTERM MF	GO:0008233 peptidase activity	12	2.21E-03	CG31821, CG30049, ApepP, CG42264, S-Lap7, gd, SPE, CG6465, cathD, Ance, sda, PGRP-SA
GOTERM BP	GO:0006508 proteolysis	11	1.06E-02	CG31821, CG30049, CG42264, Nedd8, S-Lap7, gd, SPE, CG6465, cathD, Ance, sda
GOTERM MF	GO:0070011 peptidase activity, acting on L-amino acid peptides	10	1.47E-02	CG31821, ApepP, CG42264, S-Lap7, gd, SPE, CG6465, cathD, Ance, sda
GOTERM MF	GO:0008237 metallopeptidase activity	5	3.28E-02	CG42264, S-Lap7, CG6465, Ance, sda
SWISSPROT PIR	Protease	5	3.85E-02	gd, SPE, cathD, Ance, PGRP-SA

**Annotation Cluster 3**	**Enrichment Score: 2.535780529609017**			
SWISSPROT PIR	disulfide bond	9	1.56E-03	crq, Adk2, gd, LysC, C1GalTA, Ace, CG5210, Ance, PGRP-SA
SWISSPROT PIR	glycoprotein	12	2.31E-03	PGRP-LE, crq, Orct2, Gr64a, pip, gd, Mdr49, C1GalTA, ninaG, Ace, CG5210, Ance
UP_SEQ_FEATURE	glycosylation site:N-linked (GlcNAc...)	12	6.87E-03	PGRP-LE, crq, Orct2, Gr64a, pip, gd, Mdr49, C1GalTA, ninaG, Ace, CG5210, Ance
**Annotation Cluster 4**	**Enrichment Score: 2.302060885904615**			
GOTERM MF	GO:0008238 exopeptidase activity	6	3.74E-04	CG31821, ApepP, CG42264, S-Lap7, Ance, sda
SWISSPROT PIR	carboxypeptidase	3	1.40E-02	CG31821, CG42264, Ance
GOTERM MF	GO:0004180 carboxypeptidase activity	3	2.37E-02	CG31821, CG42264, Ance
**Annotation Cluster 5**	**Enrichment Score: 2.2415486548709147**			
GOTERM MF	GO:0008238 exopeptidase activity	6	3.74E-04	CG31821, ApepP, CG42264, S-Lap7, Ance, sda
SWISSPROT PIR	Aminopeptidase	3	1.62E-02	ApepP, S-Lap7, sda
GOTERM MF	GO:0004177 aminopeptidase activity	3	3.11E-02	ApepP, S-Lap7, sda
**Annotation Cluster 6**	**Enrichment Score: 2.0347426002055675**			
SWISSPROT PIR	disulfide bond	9	1.56E-03	crq, Adk2, gd, LysC, C1GalTA, Ace, CG5210, Ance, PGRP-SA

SWISSPROT PIR	signal	11	4.87E-03	Acp1, Cpr47Eg, Acp53Ea, gd, LysC, ninaG, rumi, Ace, CG5210, Ance, PGRP-SA
SWISSPROT PIR	Secreted	7	1.17E-02	PGRP-LE, Acp53Ea, gd, ninaG, CG5210, Ance, PGRP-SA
UP_SEQ_FEATURE	disulfide bond	8	1.55E-02	crq, Adk2, gd, LysC, Ace, CG5210, Ance, PGRP-SA
UP_SEQ_FEATURE	signal peptide	11	1.60E-02	Acp1, Cpr47Eg, Acp53Ea, gd, LysC, ninaG, rumi, Ace, CG5210, Ance, PGRP-SA
GOTERM CC	GO:0005576 extracellular region	12	2.79E-02	PGRP-LE, Cpr47Eg, obst-A, Acp53Ea, CG6933, gd, ninaG, CG5210, Ance, PGRP-SA, Spn27A, CG17739
**Annotation Cluster 7**	**Enrichment Score: 1.8301156780099452**			
GOTERM BP	GO:0008063 Toll signaling pathway	5	5.04E-04	pip, gd, SPE, PGRP-SA, Spn27A
GOTERM BP	GO:0016485 protein processing	3	7.78E-02	pip, gd, SPE
GOTERM BP	GO:0051604 protein maturation	3	8.25E-02	pip, gd, SPE
**Annotation Cluster 8**	**Enrichment Score: 1.5505648241814785**			
GOTERM BP	GO:0006026 aminoglycan catabolic process	3	1.50E-02	PGRP-LE, CG5210, PGRP-SA
GOTERM BP	GO:0000272 polysaccharide catabolic process	3	1.76E-02	PGRP-LE, CG5210, PGRP-SA
GOTERM BP	GO:0006022 aminoglycan metabolic process	5	4.30E-02	PGRP-LE, obst-A, CG6933, CG5210, PGRP-SA

GOTERM BP	GO:0005976 polysaccharide metabolic process	5	5.51E-02	PGRP-LE, obst-A, CG6933, CG5210, PGRP-SA
**Annotation Cluster 9**	**Enrichment Score: 1.5454649543144068**			
GOTERM BP	GO:0006952 defense response	7	3.29E-03	PGRP-LE, crq, Tak1, LysC, SPE, PGRP-SA, Spn27A
GOTERM BP	GO:0006955 immune response	7	3.66E-03	PGRP-LE, crq, Tak1, LysC, SPE, PGRP-SA, Spn27A
GOTERM BP	GO:0045087 innate immune response	5	9.80E-03	PGRP-LE, Tak1, SPE, PGRP-SA, Spn27A
GOTERM BP	GO:0050830 defense response to Gram-positive bacterium	3	1.76E-02	PGRP-LE, SPE, PGRP-SA
GOTERM BP	GO:0042742 defense response to bacterium	4	2.55E-02	PGRP-LE, LysC, SPE, PGRP-SA
GOTERM BP	GO:0002786 regulation of antibacterial peptide production	3	2.62E-02	Tak1, SPE, PGRP-SA
GOTERM BP	GO:0002808 regulation of antibacterial peptide biosynthetic process	3	2.62E-02	Tak1, SPE, PGRP-SA
GOTERM BP	GO:0006963 positive regulation of antibacterial peptide biosynthetic process	3	2.62E-02	Tak1, SPE, PGRP-SA
GOTERM BP	GO:0002697 regulation of immune	3	3.99E-02	Tak1, SPE, PGRP-SA

	effector process			
GOTERM BP	GO:0002700 regulation of production of molecular mediator of immune response	3	3.99E-02	Tak1, SPE, PGRP-SA
GOTERM BP	GO:0002784 regulation of antimicrobial peptide production	3	3.99E-02	Tak1, SPE, PGRP-SA
GOTERM BP	GO:0002805 regulation of antimicrobial peptide biosynthetic process	3	3.99E-02	Tak1, SPE, PGRP-SA
GOTERM BP	GO:0002807 positive regulation of antimicrobial peptide biosynthetic process	3	3.99E-02	Tak1, SPE, PGRP-SA
GOTERM BP	GO:0009617 response to bacterium	4	4.07E-02	PGRP-LE, LysC, SPE, PGRP-SA
GOTERM BP	GO:0002759 regulation of antimicrobial humoral response	3	5.15E-02	Tak1, SPE, PGRP-SA
GOTERM BP	GO:0043900 regulation of multi-organism process	3	5.15E-02	Tak1, SPE, PGRP-SA
GOTERM BP	GO:0002831 regulation of response to biotic stimulus	3	5.15E-02	Tak1, SPE, PGRP-SA
GOTERM BP	GO:0002920 regulation of humoral immune response	3	5.15E-02	Tak1, SPE, PGRP-SA

SWISSPROT PIR	innate immunity	3	6.30E-02	PGRP-LE, Tak1, PGRP-SA
SWISSPROT PIR	immune response	3	7.08E-02	PGRP-LE, Tak1, PGRP-SA
**Annotation Cluster 10**	**Enrichment Score: 1.3185781035185091**			
SWISSPROT PIR	membrane	16	3.33E-02	C1GalTA, eca, Ace, rost, Cyp6g1, crq, Orct2, Tret1-1, Gr64a, Gr93c, pip, Drip, Mdr49, ppk19, GluRIIA, Tsp42Ee
SWISSPROT PIR	transmembrane	15	3.61E-02	C1GalTA, eca, rost, crq, Orct2, Tret1-1, Gr64a, Gr93c, pip, Drip, Mdr49, ppk19, GluRIIA, Tsp42Ee, CG32053
GOTERM CC	GO:0031224 intrinsic to membrane	21	9.22E-02	PGRP-LE, CG13078, C1GalTA, eca, CG11601, Ace, rost, crq, Orct2, Tret1-1, Gr64a, Gr93c, pip, Drip, Mdr49, PGRP-SA, Elo68alpha, ppk19, CG32053, Tsp42Ee, GluRIIA
**Annotation Cluster 11**	**Enrichment Score: 1.2323161117351618**			
SWISSPROT PIR	synapse	3	4.14E-02	Snap25, Ace, GluRIIA
GOTERM CC	GO:0045202 synapse	5	6.14E-02	eIF-4E, Snap25, CG31272, Ace, GluRIIA
SWISSPROT PIR	cell junction	3	7.90E-02	Snap25, Ace, GluRIIA

Table S11 GO term and functional classification enrichment analysis using DAVID for gene families that are contracted in the *Drosophila suzukii* genome as compared to the basal paraphyletic group of Drosophilidae including *D. ananassae, D. persimilis, D. pseudoobscura, D. willistoni, D. grimshawi, D. mojavensis*, and *D. virulis*.

CATEGORY	TERM	COUNT	P VALUE	GENE FAMILIES: REPRESENTATIVE GENE SYMBOLS
**Annotation Cluster 1**	**Enrichment Score: 2.6163350631215847**			
GOTERM CC	GO:0000786 nucleosome	5	2.00E-05	His2B:CG33882, His2Av, His1:CG33834, His4r, His3.3A
SWISSPROT PIR	nucleosome core	4	5.35E-05	His2B:CG33882, His2Av, His4r, His3.3A
INTERPRO	IPR007125:Histone core	4	1.79E-04	His2B:CG33882, His2Av, His4r, His3.3A
GOTERM BP	GO:0006334 nucleosome assembly	5	2.47E-04	His2B:CG33882, His2Av, His1:CG33834, His4r, His3.3A
GOTERM BP	GO:0034728 nucleosome organization	6	2.59E-04	His2B:CG33882, His2Av, His1:CG33834, Nipped-A, His4r, His3.3A
GOTERM BP	GO:0031497 chromatin assembly	5	9.05E-04	His2B:CG33882, His2Av, His1:CG33834, His4r, His3.3A
SWISSPROT PIR	acetylation	4	1.38E-03	His2Av, Adh, His4r, His3.3A
SWISSPROT PIR	chromosomal protein	5	1.83E-03	His2B:CG33882, His2Av, His1:CG33834, His4r, His3.3A
GOTERM CC	GO:0032993 protein-DNA complex	5	2.27E-03	His2B:CG33882, His2Av, His1:CG33834, His4r, His3.3A
GOTERM BP	GO:0065004 protein-DNA complex assembly	5	2.31E-03	His2B:CG33882, His2Av, His1:CG33834, His4r, His3.3A
GOTERM BP	GO:0006323 DNA packaging	6	2.50E-03	eIF-4E, His2B:CG33882, His2Av, His1:CG33834, His4r, His3.3A
INTERPRO	IPR009072:Histone-fold	4	7.08E-03	His2B:CG33882, His2Av, His4r, His3.3A
GOTERM BP	GO:0006333 chromatin assembly or disassembly	5	7.12E-03	His2B:CG33882, His2Av, His1:CG33834, His4r, His3.3A
GOTERM CC	GO:0035267 NuA4 histone	3	2.73E-02	His2B:CG33882, His2Av, Nipped-A

	acetyltransferase complex			
GOTERM CC	GO:0043189 H4/H2A histone acetyltransferase complex	3	4.89E-02	His2B:CG33882, His2Av, Nipped-A
GOTERM BP	GO:0006325 chromatin organization	7	5.87E-02	eIF-4E, His2B:CG33882, His2Av, His1:CG33834, Nipped-A, His4r, His3.3A
GOTERM BP	GO:0034621 cellular macromolecular complex subunit organization	7	7.13E-02	His2B:CG33882, His2Av, His1:CG33834, gammaTub23C, Nipped-A, His4r, His3.3A
GOTERM BP	GO:0034622 cellular macromolecular complex assembly	6	7.82E-02	His2B:CG33882, His2Av, His1:CG33834, gammaTub23C, His4r, His3.3A
**Annotation Cluster 2**	**Enrichment Score: 2.1187388547153736**			
GOTERM BP	GO:0006508 proteolysis	15	1.10E-03	lwr, CG31821, S-Lap7, Roc1a, SPE, cathD, CG11864, CG30049, CG42264, CG32486, Nedd8, Prosbeta3, gd, CG6465, Ance
GOTERM MF	GO:0008233 peptidase activity	13	3.84E-03	CG31821, ApepP, S-Lap7, SPE, cathD, CG11864, CG30049, CG42264, Prosbeta3, gd, CG6465, PGRP-SA, Ance
SWISSPROT PIR	Protease	7	5.19E-03	Prosbeta3, gd, SPE, cathD, CG11864, Ance, PGRP-SA
GOTERM MF	GO:0008238 exopeptidase activity	5	7.22E-03	CG31821, ApepP, CG42264, S-Lap7, Ance
GOTERM MF	GO:0070011 peptidase activity, acting on L-amino acid peptides	11	1.99E-02	CG31821, ApepP, CG42264, Prosbeta3, S-Lap7, gd, SPE, CG6465, cathD, CG11864, Ance
GOTERM MF	GO:0008237 metallopeptidase activity	5	6.15E-02	CG42264, S-Lap7, CG6465, CG11864, Ance

**Annotation Cluster 3**	**Enrichment Score: 2.085600214435509**			
SWISSPROT PIR	glycoprotein	14	1.64E-03	PGRP-LE, CG4928, C1GalTA, Ace, crq, Orct2, Gr64a, pip, gd, Mdr49, ninaG, prominin-like, CG5210, Ance
UP_SEQ_FEATURE	glycosylation site:N-linked (GlcNAc...)	14	6.01E-03	PGRP-LE, CG4928, C1GalTA, Ace, crq, Orct2, Gr64a, pip, gd, Mdr49, ninaG, prominin-like, CG5210, Ance
SWISSPROT PIR	membrane	18	5.62E-02	CG4928, C1GalTA, eca, Ace, rost, Cyp6g1, crq, Orct2, Tret1-1, Gr64a, Gr93c, pip, Drip, Mdr49, prominin-like, ppk19, GluRIIA, Tsp42Ee
**Annotation Cluster 4**	**Enrichment Score: 1.8933035516707737**			
SWISSPROT PIR	disulfide bond	10	1.72E-03	crq, Adk2, Amyrel, gd, LysC, C1GalTA, Ace, CG5210, Ance, PGRP-SA
SWISSPROT PIR	signal	12	8.54E-03	Acp1, Cpr47Eg, Amyrel, gd, LysC, ninaG, rumi, Dpt, Ace, CG5210, Ance, PGRP-SA
SWISSPROT PIR	Secreted	8	9.74E-03	PGRP-LE, Amyrel, gd, ninaG, Dpt, CG5210, Ance, PGRP-SA
UP_SEQ_FEATURE	disulfide bond	9	1.73E-02	crq, Adk2, Amyrel, gd, LysC, Ace, CG5210, Ance, PGRP-SA
UP_SEQ_FEATURE	signal peptide	12	3.10E-02	Acp1, Cpr47Eg, Amyrel, gd, LysC, ninaG, rumi, Dpt, Ace, CG5210, Ance, PGRP-SA
GOTERM CC	GO:0005576 extracellular region	13	5.70E-02	PGRP-LE, Cpr47Eg, Amyrel, CG17575, Dpt, obst-A, gd, ninaG, CG5210, Muc26B, PGRP-SA, Ance, CG17739
**Annotation Cluster 5**	**Enrichment Score: 1.7564554169324464**			

GOTERM MF	GO:0008238 exopeptidase activity	5	7.22E-03	CG31821, ApepP, CG42264, S-Lap7, Ance
SWISSPROT PIR	carboxypeptidase	3	2.15E-02	CG31821, CG42264, Ance
GOTERM MF	GO:0004180 carboxypeptidase activity	3	3.47E-02	CG31821, CG42264, Ance
**Annotation Cluster 6**	**Enrichment Score: 1.4889465105264814**			
GOTERM BP	GO:0006955 immune response	8	2.87E-03	lwr, PGRP-LE, crq, Tak1, LysC, SPE, Dpt, PGRP-SA
GOTERM BP	GO:0042742 defense response to bacterium	5	8.54E-03	PGRP-LE, LysC, SPE, Dpt, PGRP-SA
GOTERM BP	GO:0006952 defense response	7	1.08E-02	PGRP-LE, crq, Tak1, LysC, SPE, Dpt, PGRP-SA
SWISSPROT PIR	innate immunity	4	1.43E-02	PGRP-LE, Tak1, Dpt, PGRP-SA
GOTERM BP	GO:0009617 response to bacterium	5	1.61E-02	PGRP-LE, LysC, SPE, Dpt, PGRP-SA
SWISSPROT PIR	immune response	4	1.71E-02	PGRP-LE, Tak1, Dpt, PGRP-SA
GOTERM BP	GO:0045087 innate immune response	5	2.24E-02	PGRP-LE, Tak1, SPE, Dpt, PGRP-SA
GOTERM BP	GO:0050830 defense response to Gram-positive bacterium	3	2.79E-02	PGRP-LE, SPE, PGRP-SA
GOTERM BP	GO:0002786 regulation of antibacterial peptide production	3	4.12E-02	Tak1, SPE, PGRP-SA
GOTERM BP	GO:0002808 regulation of antibacterial peptide biosynthetic	3	4.12E-02	Tak1, SPE, PGRP-SA

	process			
GOTERM BP	GO:0006963 positive regulation of antibacterial peptide biosynthetic process	3	4.12E-02	Tak1, SPE, PGRP-SA
GOTERM BP	GO:0002697 regulation of immune effector process	3	6.20E-02	Tak1, SPE, PGRP-SA
GOTERM BP	GO:0002700 regulation of production of molecular mediator of immune response	3	6.20E-02	Tak1, SPE, PGRP-SA
GOTERM BP	GO:0002784 regulation of antimicrobial peptide production	3	6.20E-02	Tak1, SPE, PGRP-SA
GOTERM BP	GO:0002805 regulation of antimicrobial peptide biosynthetic process	3	6.20E-02	Tak1, SPE, PGRP-SA
GOTERM BP	GO:0002807 positive regulation of antimicrobial peptide biosynthetic process	3	6.20E-02	Tak1, SPE, PGRP-SA
GOTERM BP	GO:0043900 regulation of multi-organism process	3	7.94E-02	Tak1, SPE, PGRP-SA
GOTERM BP	GO:0002759 regulation of antimicrobial humoral response	3	7.94E-02	Tak1, SPE, PGRP-SA

GOTERM BP	GO:0002831 regulation of response to biotic stimulus	3	7.94E-02	Tak1, SPE, PGRP-SA
GOTERM BP	GO:0002920 regulation of humoral immune response	3	7.94E-02	Tak1, SPE, PGRP-SA
**Annotation Cluster 7**	**Enrichment Score: 1.3629891034285229**			
GOTERM BP	GO:0006026 aminoglycan catabolic process	3	2.39E-02	PGRP-LE, CG5210, PGRP-SA
GOTERM BP	GO:0000272 polysaccharide catabolic process	3	2.79E-02	PGRP-LE, CG5210, PGRP-SA
GOTERM BP	GO:0009057 macromolecule catabolic process	8	5.95E-02	lwr, PGRP-LE, Prosbeta3, Nedd8, CG32486, Roc1a, CG5210, PGRP-SA
GOTERM BP	GO:0006022 aminoglycan metabolic process	5	8.90E-02	PGRP-LE, obst-A, Muc26B, CG5210, PGRP-SA

Table S12 **GO term and functional classification enrichment analysis using DAVID for gene families that are contracted in the *Drosophila suzukii* genome as compared to species in the melanogaster subgroup, including *D. simulans, D. sechellia, D. yakuba, D. erecta, and D. melanogaster*.**

CATEGORY	TERM	COUNT	P VALUE	GENE FAMILIES: REPRESENTATIVE GENE SYMBOLS
**Annotation Cluster 1**	**Enrichment Score: 2.764122751765743**			
GOTERM BP	GO:0008063 Toll signaling pathway	6	9.04E-05	IM10, pip, gd, SPE, PGRP-SA, Spn27A
GOTERM BP	GO:0006952 defense response	9	4.43E-04	PGRP-LE, crq, IM10, Tak1, LysC, SPE, IM2, PGRP-SA, Spn27A
GOTERM BP	GO:0006955 immune response	9	5.13E-04	PGRP-LE, crq, IM10, Tak1, LysC, SPE, IM2, PGRP-SA, Spn27A
GOTERM BP	GO:0045087 innate immune response	7	5.86E-04	PGRP-LE, IM10, Tak1, SPE, IM2, PGRP-SA, Spn27A
SWISSPROT PIR	innate immunity	5	1.65E-03	PGRP-LE, IM10, Tak1, IM2, PGRP-SA
SWISSPROT PIR	immune response	5	2.12E-03	PGRP-LE, IM10, Tak1, IM2, PGRP-SA
GOTERM BP	GO:0042742 defense response to bacterium	5	7.91E-03	PGRP-LE, IM10, LysC, SPE, PGRP-SA
GOTERM BP	GO:0009617 response to bacterium	5	1.49E-02	PGRP-LE, IM10, LysC, SPE, PGRP-SA
GOTERM BP	GO:0050830 defense response to Gram-positive bacterium	3	2.68E-02	PGRP-LE, SPE, PGRP-SA
**Annotation Cluster 2**	**Enrichment Score: 2.735311860428723**			
GOTERM CC	GO:0000786 nucleosome	5	1.78E-05	His2B:CG33882, His2Av, His1:CG33834, His4r, His3.3A
SWISSPROT PIR	nucleosome core	4	5.45E-05	His2B:CG33882, His2Av, His4r, His3.3A
SWISSPROT PIR	acetylation	5	6.19E-05	His2Av, Adh, Cam, His4r, His3.3A

INTERPRO	IPR007125:Histone core	4	1.50E-04	His2B:CG33882, His2Av, His4r, His3.3A
GOTERM BP	GO:0006334 nucleosome assembly	5	2.27E-04	His2B:CG33882, His2Av, His1:CG33834, His4r, His3.3A
GOTERM BP	GO:0031497 chromatin assembly	5	8.33E-04	His2B:CG33882, His2Av, His1:CG33834, His4r, His3.3A
SWISSPROT PIR	chromosomal protein	5	1.87E-03	His2B:CG33882, His2Av, His1:CG33834, His4r, His3.3A
GOTERM CC	GO:0032993 protein-DNA complex	5	2.04E-03	His2B:CG33882, His2Av, His1:CG33834, His4r, His3.3A
GOTERM BP	GO:0065004 protein-DNA complex assembly	5	2.13E-03	His2B:CG33882, His2Av, His1:CG33834, His4r, His3.3A
GOTERM BP	GO:0006323 DNA packaging	6	2.27E-03	eIF-4E, His2B:CG33882, His2Av, His1:CG33834, His4r, His3.3A
GOTERM BP	GO:0034728 nucleosome organization	5	2.43E-03	His2B:CG33882, His2Av, His1:CG33834, His4r, His3.3A
INTERPRO	IPR009072:Histone-fold	4	6.00E-03	His2B:CG33882, His2Av, His4r, His3.3A
GOTERM BP	GO:0006333 chromatin assembly or disassembly	5	6.59E-03	His2B:CG33882, His2Av, His1:CG33834, His4r, His3.3A
GOTERM CC	GO:0000785 chromatin	6	4.10E-02	His2B:CG33882, His2Av, CG34317, His1:CG33834, His4r, His3.3A
GOTERM BP	GO:0006325 chromatin organization	7	5.38E-02	eIF-4E, His2B:CG33882, His2Av, CG34317, His1:CG33834, His4r, His3.3A
GOTERM BP	GO:0034621 cellular macromolecular complex subunit organization	7	6.55E-02	His2B:CG33882, His2Av, His1:CG33834, gammaTub23C, Cam, His4r, His3.3A
GOTERM BP	GO:0034622 cellular macromolecular complex assembly	6	7.26E-02	His2B:CG33882, His2Av, His1:CG33834, gammaTub23C, His4r, His3.3A

**Annotation Cluster 3**	**Enrichment Score: 2.6131075171385785**			
SWISSPROT PIR	disulfide bond	11	4.43E-04	crq, Adk2, gd, LysC, C1GalTA, IM2, Ace, CG5210, mth, Ance, PGRP-SA
SWISSPROT PIR	signal	14	1.07E-03	Acp1, Cpr47Eg, IM10, Acp53Ea, Ace, gd, LysC, rumi, ninaG, IM2, CG5210, PGRP-SA, mth, Ance
SWISSPROT PIR	Secreted	9	2.70E-03	PGRP-LE, IM10, Acp53Ea, gd, ninaG, IM2, CG5210, Ance, PGRP-SA
UP_SEQ_FEATURE	signal peptide	14	3.81E-03	Acp1, Cpr47Eg, IM10, Acp53Ea, Ace, gd, LysC, rumi, ninaG, IM2, CG5210, PGRP-SA, mth, Ance
UP_SEQ_FEATURE	disulfide bond	10	4.65E-03	crq, Adk2, gd, LysC, IM2, Ace, CG5210, mth, Ance, PGRP-SA
GOTERM CC	GO:0005576 extracellular region	15	9.25E-03	PGRP-LE, Cpr47Eg, IM10, CheB42a, Acp53Ea, Spn27A, CG6933, gd, ninaG, IM2, CG5210, Muc26B, PGRP-SA, Ance, CG17739
**Annotation Cluster 4**	**Enrichment Score: 2.452373611334107**			
SWISSPROT PIR	disulfide bond	11	4.43E-04	crq, Adk2, gd, LysC, C1GalTA, IM2, Ace, CG5210, mth, Ance, PGRP-SA
SWISSPROT PIR	glycoprotein	14	1.74E-03	PGRP-LE, IM10, C1GalTA, Ace, crq, Orct2, Gr64a, pip, gd, Mdr49, ninaG, CG5210, mth, Ance
UP_SEQ_FEATURE	glycosylation site:N-linked (GlcNAc...)	14	4.96E-03	PGRP-LE, IM10, C1GalTA, Ace, crq, Orct2, Gr64a, pip, gd, Mdr49, ninaG, CG5210, mth, Ance
UP_SEQ_FEATURE	topological domain:Cytoplasmic	11	4.06E-02	crq, Orct2, Gr64a, Gr93c, Or65a, pip, Mdr49, C1GalTA, Drip, Gr22f, mth
**Annotation Cluster 5**	**Enrichment Score: 2.2260947064240506**			

GOTERM MF	GO:0008238 exopeptidase activity	6	7.73E-04	CG31821, ApepP, CG42264, S-Lap7, Ance, sda
GOTERM MF	GO:0008233 peptidase activity	13	2.58E-03	CG31821, ApepP, S-Lap7, SPE, CG42370, cathD, CG30049, CG42264, gd, CG6465, PGRP-SA, sda, Ance
GOTERM MF	GO:0008237 metallopeptidase activity	6	1.34E-02	CG42264, S-Lap7, CG42370, CG6465, Ance, sda
GOTERM MF	GO:0070011 peptidase activity, acting on L-amino acid peptides	11	1.46E-02	CG31821, ApepP, CG42264, S-Lap7, gd, CG42370, SPE, CG6465, cathD, Ance, sda
GOTERM BP	GO:0006508 proteolysis	12	1.88E-02	CG31821, CG30049, CG42264, Nedd8, S-Lap7, gd, CG42370, SPE, CG6465, cathD, Ance, sda
**Annotation Cluster 6**	**Enrichment Score: 2.0907188176313323**			
GOTERM MF	GO:0008238 exopeptidase activity	6	7.73E-04	CG31821, ApepP, CG42264, S-Lap7, Ance, sda
SWISSPROT PIR	carboxypeptidase	3	2.18E-02	CG31821, CG42264, Ance
GOTERM MF	GO:0004180 carboxypeptidase activity	3	3.18E-02	CG31821, CG42264, Ance
**Annotation Cluster 7**	**Enrichment Score: 2.0311596068651054**			
GOTERM MF	GO:0008238 exopeptidase activity	6	7.73E-04	CG31821, ApepP, CG42264, S-Lap7, Ance, sda
SWISSPROT PIR	Aminopeptidase	3	2.51E-02	ApepP, S-Lap7, sda
GOTERM MF	GO:0004177 aminopeptidase activity	3	4.16E-02	ApepP, S-Lap7, sda

**Annotation Cluster 8**	**Enrichment Score: 1.8933328846785107**			
SWISSPROT PIR	transmembrane	22	1.60E-03	CG13796, Or65a, C1GalTA, eca, Gr22f, rost, crq, Orct2, Tret1-1, Gr64a, Gr93c, pip, CG32301, Drip, Mdr49, sesB, mth, ppk19, CG7255, CG32053, Tsp42Ee, GluRIIA
SWISSPROT PIR	membrane	21	8.80E-03	Or65a, C1GalTA, Gr22f, eca, Ace, rost, Cyp6g1, crq, Orct2, Tret1-1, Gr64a, Gr93c, pip, CG32301, Drip, Mdr49, sesB, mth, ppk19, Tsp42Ee, GluRIIA
GOTERM CC	GO:0031224 intrinsic to membrane	28	1.40E-02	PGRP-LE, CG13078, Or65a, eca, Gr22f, Ace, Gr93c, PGRP-SA, mth, ppk19, Elo68alpha, CG7255, CG32053, GluRIIA, Tsp42Ee, CG13796, C1GalTA, CG11601, rost, crq, Orct2, Tret1-1, Gr64a, pip, Drip, Mdr49, CG32301, sesB
GOTERM CC	GO:0016021 integral to membrane	27	2.10E-02	PGRP-LE, CG13078, Or65a, eca, Gr22f, Gr93c, PGRP-SA, mth, ppk19, Elo68alpha, CG32053, GluRIIA, Tsp42Ee, CG7255, CG13796, C1GalTA, CG11601, rost, crq, Orct2, Tret1-1, Gr64a, pip, Drip, Mdr49, CG32301, sesB
SWISSPROT PIR	cell membrane	8	2.59E-02	crq, Gr64a, Gr93c, Or65a, Gr22f, Ace, mth, GluRIIA
UP_SEQ_FEATURE	topological domain:Cytoplasmic	11	4.06E-02	crq, Orct2, Gr64a, Gr93c, Or65a, pip, Mdr49, C1GalTA, Drip, Gr22f, mth
**Annotation Cluster 9**	**Enrichment Score: 1.4296819844314266**			
GOTERM BP	GO:0006026 aminoglycan catabolic	3	2.29E-02	PGRP-LE, CG5210, PGRP-SA

		process			
GOTERM BP	GO:0000272	polysaccharide catabolic process	3	2.68E-02	PGRP-LE, CG5210, PGRP-SA
GOTERM BP	GO:0006022	aminoglycan metabolic process	5	8.37E-02	PGRP-LE, CG6933, Muc26B, CG5210, PGRP-SA
**Annotation Cluster 10**	**Enrichment Score: 1.4055451400858099**				
GOTERM BP	GO:0008219	cell death	8	9.69E-03	eIF-4E, Cyt-c-d, crq, qkr58E-3, Eig71Ej, Tak1, LysC, cathD
GOTERM BP	GO:0016265	death	8	1.01E-02	eIF-4E, Cyt-c-d, crq, qkr58E-3, Eig71Ej, Tak1, LysC, cathD
GOTERM BP	GO:0012501	programmed cell death	7	2.78E-02	eIF-4E, Cyt-c-d, crq, qkr58E-3, Eig71Ej, Tak1, cathD
GOTERM BP	GO:0035070	salivary gland histolysis	4	7.03E-02	eIF-4E, crq, Eig71Ej, cathD
GOTERM BP	GO:0035071	salivary gland cell autophagic cell death	4	7.03E-02	eIF-4E, crq, Eig71Ej, cathD
GOTERM BP	GO:0048102	autophagic cell death	4	7.03E-02	eIF-4E, crq, Eig71Ej, cathD
GOTERM BP	GO:0016271	tissue death	4	7.75E-02	eIF-4E, crq, Eig71Ej, cathD
GOTERM BP	GO:0007559	histolysis	4	7.75E-02	eIF-4E, crq, Eig71Ej, cathD
**Annotation Cluster 11**	**Enrichment Score: 1.3869132158526516**				
GOTERM BP	GO:0045087	innate immune response	7	5.86E-04	PGRP-LE, IM10, Tak1, SPE, IM2, PGRP-SA, Spn27A
GOTERM BP	GO:0002786	regulation of	3	3.96E-02	Tak1, SPE, PGRP-SA

	antibacterial peptide production			
GOTERM BP	GO:0002808 regulation of antibacterial peptide biosynthetic process	3	3.96E-02	Tak1, SPE, PGRP-SA
GOTERM BP	GO:0006963 positive regulation of antibacterial peptide biosynthetic process	3	3.96E-02	Tak1, SPE, PGRP-SA
GOTERM BP	GO:0002697 regulation of immune effector process	3	5.96E-02	Tak1, SPE, PGRP-SA
GOTERM BP	GO:0002700 regulation of production of molecular mediator of immune response	3	5.96E-02	Tak1, SPE, PGRP-SA
GOTERM BP	GO:0002784 regulation of antimicrobial peptide production	3	5.96E-02	Tak1, SPE, PGRP-SA
GOTERM BP	GO:0002805 regulation of antimicrobial peptide biosynthetic process	3	5.96E-02	Tak1, SPE, PGRP-SA
GOTERM BP	GO:0002807 positive regulation of antimicrobial peptide biosynthetic process	3	5.96E-02	Tak1, SPE, PGRP-SA
GOTERM BP	GO:0002759 regulation of	3	7.64E-02	Tak1, SPE, PGRP-SA

	antimicrobial humoral response			
GOTERM BP	GO:0002831 regulation of response to biotic stimulus	3	7.64E-02	Tak1, SPE, PGRP-SA
GOTERM BP	GO:0043900 regulation of multi-organism process	3	7.64E-02	Tak1, SPE, PGRP-SA
GOTERM BP	GO:0002920 regulation of humoral immune response	3	7.64E-02	Tak1, SPE, PGRP-SA

Table S13 Gene families of enzymes involved in metabolism and transport of xenobiotics.

Gene Family	Representative Genes	Dmel	Dsec	Dsim	Dere	Dyak	Dbia	Dsuz	Dtak	Dana	Dper	Dpse	Dwil	Dgri	Dmoj	Dvir
Esterases	Ace, α-Est2, α-Est7	31	34	28	32	35	23	**27**	23	34	34	35	43	28	36	35
GST	GstD2, GstD7, GstE12	25	28	25	26	28	8	**9**	7	31	20	21	29	21	19	21
GST	GstO1, GstO2, GstO3	4	5	5	5	5	1	**2**	1	5	5	5	5	3	3	4
GST	GstT1, GstT2, GstT3	4	4	4	4	4	1	**3**	2	5	4	4	4	3	3	3
GST	GstZ2,GstZ1	2	2	2	2	2	2	**3**	2	2	2	2	2	3	2	2
GST	GstS1	1	1	1	1	1	1	**1**	1	1	1	1	1	1	1	1
Cyp type 3, 4	Cyp4e1, Cyp6g1, Cyp6a2	68	78	72	67	74	52	**55**	56	79	65	66	78	72	61	64
mt Cyp	Cyp12d1, Cyp49a1	11	11	11	12	11	7	**10**	8	11	13	14	13	11	10	9
Cyp	Cyp1, Cyp33	8	9	7	8	8	5	**7**	6	7	6	7	8	11	12	10
Cyp type 2	Cyp18a1, Cyp303a1	4	4	4	4	5	3	**4**	3	4	6	4	4	5	5	6
UGT	Ugt35a, Ugt86Dd	35	37	34	32	41	24	**28**	26	39	30	31	43	30	29	32
UGT	Ugt	1	1	1	1	3	1	**1**	1	1	2	1	1	1	1	1

Table S14 Total base pairs of transposable element families.

≥50% ALIGNMENT SCORE				≥80% ALIGNMENT SCORE		
**TE**	**bp of TE**	**% Genome**		**TE**	**bp of TE**	**% Genome**
17.6	7795	3.31E-05		17.6	1033	4.39E-06
297	40503	1.72E-04		297	23676	1.01E-04
412	89430	3.80E-04		412	25241	1.07E-04
1360	17993	7.64E-05		1360	3173	1.35E-05
1731	17344	7.36E-05		1731	452	1.92E-06
3S18	54262	2.30E-04		3S18	8250	3.50E-05
accord	10494	4.45E-05		accord	981	4.16E-06
accord2	89195	3.79E-04		accord2	13350	5.67E-05
aurora	4156	1.76E-05		aurora	1252	5.32E-06
baggins	47296	2.01E-04		baggins	2802	1.19E-05
Bari1	4052	1.72E-05		Bari1	996	4.23E-06
blood	39870	1.69E-04		blood	18479	7.84E-05
Burdock	21218	9.01E-05		Burdock	2415	1.03E-05
Circe	47357	2.01E-04		Circe	6955	2.95E-05
copia	55228	2.34E-04		copia	14472	6.14E-05
Cr1a	541563	2.30E-03		Cr1a	81608	3.46E-04
diver2	289945	1.23E-03		diver2	26290	1.12E-04
Dm88	23210	9.85E-05		Dm88	1388	5.89E-06
Doc	9907	4.21E-05		Doc	1788	7.59E-06
Doc2	42396	1.80E-04		Doc2	4842	2.06E-05
Doc3	10373	4.40E-05		Doc3	2200	9.34E-06
Doc4	44326	1.88E-04		Doc4	3121	1.32E-05
F	208622	8.86E-04		F	13812	5.86E-05
FB	5512	2.34E-05		FB	2656	1.13E-05
flea	43854	1.86E-04		flea	4294	1.82E-05
Fw2	167825	7.12E-04		Fw2	13998	5.94E-05
Fw3	41439	1.76E-04		Fw3	2412	1.02E-05
G	2196	9.32E-06		G	0	0
G3	594	2.52E-06		G3	531	2.25E-06
G4	7218	3.06E-05		G4	2368	1.01E-05
G5	15951	6.77E-05		G5	1722	7.31E-06
G5A	78918	3.35E-04		G5A	8587	3.65E-05
G7	1520	6.45E-06		G7	109	4.63E-07
GATE	317288	1.35E-03		GATE	77748	3.30E-04
gtwin	70219	2.98E-04		gtwin	27040	1.15E-04
gypsy	70978	3.01E-04		gypsy	5478	2.33E-05
gypsy10	323555	1.37E-03		gypsy10	66652	2.83E-04
gypsy11	32727	1.39E-04		gypsy11	2630	1.12E-05

gypsy12	294106	1.25E-03
gypsy2	10478	4.45E-05
gypsy3	36524	1.55E-04
gypsy4	410628	1.74E-03
gypsy5	30021	1.27E-04
gypsy6	108613	4.61E-04
gypsy7	833	3.54E-06
gypsy8	342094	1.45E-03
gypsy9	8012	3.40E-05
H	5008	2.13E-05
HB	32745	1.39E-04
HeT-A	333	1.41E-06
HMS-Beagle	26650	1.13E-04
HMS-Beagle2	10678	4.53E-05
hopper	4361	1.85E-05
hopper2	9088	3.86E-05
I	175413	7.45E-04
Idefix	78608	3.34E-04
INE-1	5216305	2.21E-02
invader1	64950	2.76E-04
invader2	73534	3.12E-04
invader3	86528	3.67E-04
invader4	8146	3.46E-05
invader5	12282	5.21E-05
invader6	39958	1.70E-04
Ivk	61341	2.60E-04
jockey	31126	1.32E-04
jockey2	107	4.54E-07
KP	185	7.85E-07
looper1	1935	8.21E-06
mariner2	970	4.12E-06
Max	153290	6.51E-04
McClintock	1457	6.19E-06
mdg1	52859	2.24E-04
mdg3	15103	6.41E-05
micropia	15739	6.68E-05
ninja-Dsim-like	110289	4.68E-04
NOF	393	1.67E-06
opus	3177	1.35E-05
qbert	1881	7.99E-06
Quasimodo	133834	5.68E-04

gypsy12	53660	2.28E-04
gypsy2	2041	8.66E-06
gypsy3	8921	3.79E-05
gypsy4	118804	5.04E-04
gypsy5	3430	1.46E-05
gypsy6	30156	1.28E-04
gypsy7	113	4.80E-07
gypsy8	45774	1.94E-04
gypsy9	2176	9.24E-06
H	0	0
HB	433	1.84E-06
HeT-A	0	0
HMS-Beagle	9670	4.11E-05
HMS-Beagle2	83	3.52E-07
hopper	0	0
hopper2	735	3.12E-06
I	49824	2.12E-04
Idefix	13748	5.84E-05
INE-1	1438828	6.11E-03
invader1	7766	3.30E-05
invader2	25244	1.07E-04
invader3	33186	1.41E-04
invader4	3718	1.58E-05
invader5	3909	1.66E-05
invader6	10630	4.51E-05
Ivk	15462	6.56E-05
jockey	6908	2.93E-05
jockey2	0	0
KP	0	0
looper1	250	1.06E-06
mariner2	0	0
Max	29677	1.26E-04
McClintock	85	3.61E-07
mdg1	5507	2.34E-05
mdg3	6040	2.56E-05
micropia	5766	2.45E-05
ninja-Dsim-like	44565	1.89E-04
NOF	0	0
opus	771	3.27E-06
qbert	0	0
Quasimodo	12155	5.16E-05

R1	5225	2.22E-05
R1-2	3144	1.33E-05
R1-element	9080	3.85E-05
roo	102780	4.36E-04
rooA	119349	5.07E-04
rover	38812	1.65E-04
Rt1a	273	1.16E-06
Rt1b	9200	3.91E-05
Rt1c	14821	6.29E-05
S	5668	2.41E-05
S2	400	1.70E-06
springer	25730	1.09E-04
Stalker	10628	4.51E-05
Stalker2	137186	5.82E-04
Stalker4	108913	4.62E-04
Tabor	256912	1.09E-03
TART-A	1406	5.97E-06
TART-B	3682	1.56E-05
Tc1	12605	5.35E-05
Tc1-2	3073	1.30E-05
Tc3	2926	1.24E-05
Tirant	17853	7.58E-05
transib1	18138	7.70E-05
transib2	20048	8.51E-05
transib3	41264	1.75E-04
transib4	1384	5.88E-06
Transpac	2560	1.09E-05
X	3605	1.53E-05
total	11542576	4.90E-02

R1	588	2.50E-06
R1-2	166	7.05E-07
R1-element	2392	1.02E-05
roo	25656	1.09E-04
rooA	18105	7.69E-05
rover	18695	7.94E-05
Rt1a	0	0
Rt1b	897	3.81E-06
Rt1c	0	0
S	462	1.96E-06
S2	0	0
springer	5239	2.22E-05
Stalker	2237	9.50E-06
Stalker2	49025	2.08E-04
Stalker4	11657	4.95E-05
Tabor	92855	3.94E-04
TART-A	437	1.86E-06
TART-B	468	1.99E-06
Tc1	472	2.00E-06
Tc1-2	0	0
Tc3	326	1.38E-06
Tirant	680	2.89E-06
transib1	3684	1.56E-05
transib2	1832	7.78E-06
transib3	6329	2.69E-05
transib4	0	0
Transpac	0	0
X	285	1.21E-06
total	2707323	1.15E-02
