# Supplementary material for: Genome of Drosophila suzukii, the Spotted Wing Drosophila
Source: G3 (Bethesda). 2013 Oct 18;3(12):2257–71. doi: 10.1534/g3.113.008185 (PMC3852387; doi:10.1534/g3.113.008185)
Supplement: Supporting Information [file supp_g3.113.008185_FigureS1.pdf]

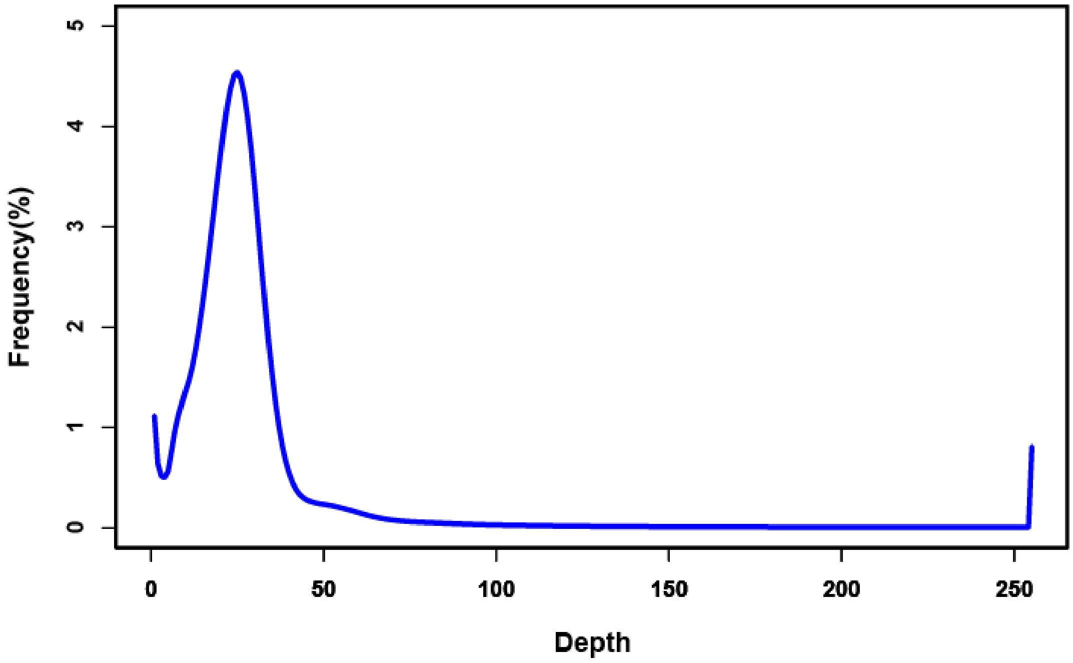

Figure S1 17-kmer estimation of genome size. The genome size of *Drosophila suzukii* was estimated to be 220Mb based on reads from short insert size libraries.
