# Supplementary material for: Genome of Drosophila suzukii, the Spotted Wing Drosophila
Source: G3 (Bethesda). 2013 Oct 18;3(12):2257–71. doi: 10.1534/g3.113.008185 (PMC3852387; doi:10.1534/g3.113.008185)
Supplement: Supporting Information [file supp_g3.113.008185_FigureS2.pdf]

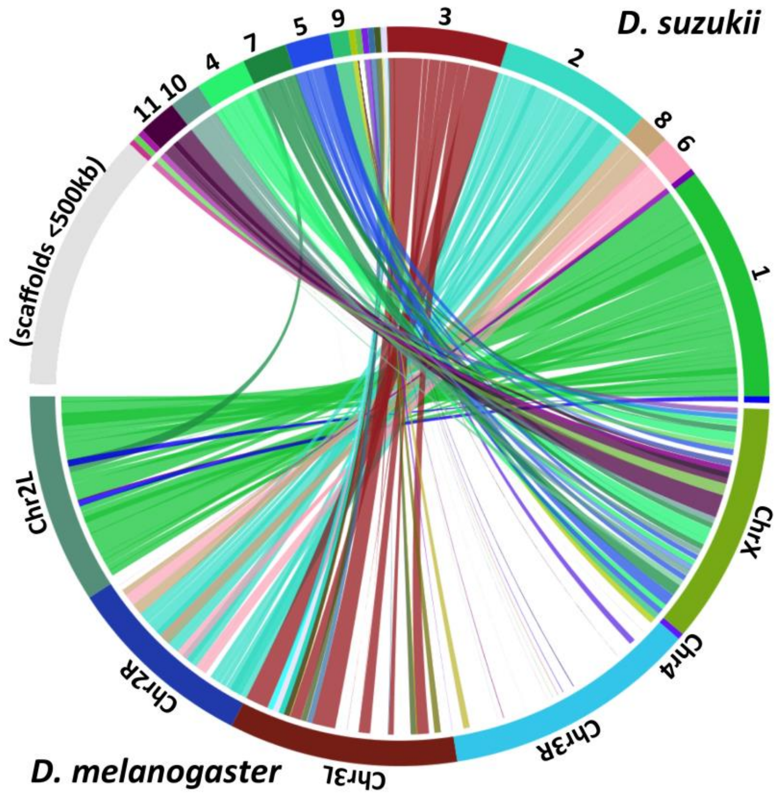

Figure S2 Synteny map between *D. suzukii* and *D. melanogaster* genomes. Only scaffolds >500kb in sizes are used in the synteny analysis. Scaffolds >2Mb in sizes are labeled.
