# Supplementary material for: Genome of Drosophila suzukii, the Spotted Wing Drosophila
Source: G3 (Bethesda). 2013 Oct 18;3(12):2257–71. doi: 10.1534/g3.113.008185 (PMC3852387; doi:10.1534/g3.113.008185)
Supplement: Supporting Information [file supp_g3.113.008185_FigureS3.pdf]

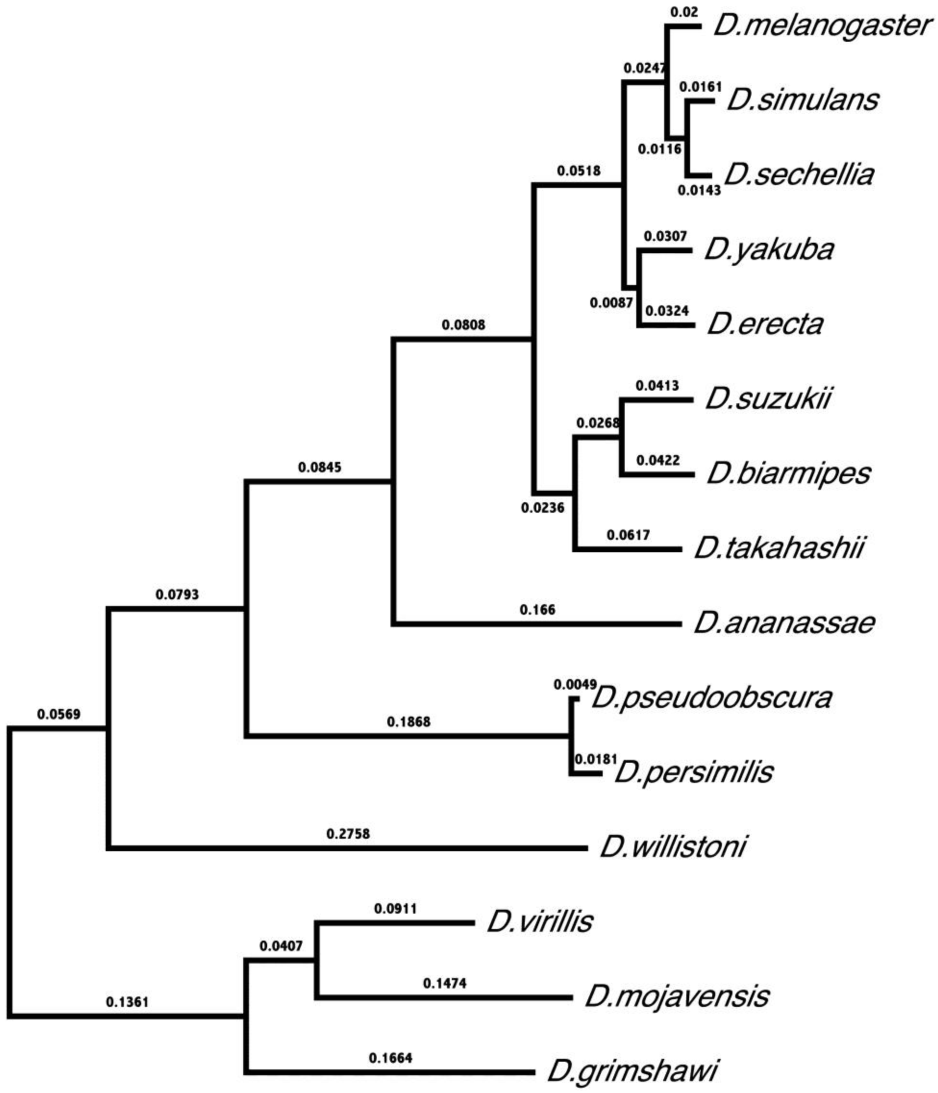

Figure S3 Best-scoring maximum likelihood (ML) tree of 15 *Drosophila* species with outgroup *A. gambiae* (not shown) using 5,322 gene partitions with 5,199,249 sites. Best model was selected individually for each partition. Bootstrap support values from 250 bootstrap replicates are 100 for all nodes. Branch labels indicate the mean number of substitutions per site.
