# Supplementary material for: Genome of Drosophila suzukii, the Spotted Wing Drosophila
Source: G3 (Bethesda). 2013 Oct 18;3(12):2257–71. doi: 10.1534/g3.113.008185 (PMC3852387; doi:10.1534/g3.113.008185)
Supplement: Supporting Information [file supp_g3.113.008185_FigureS4.pdf]

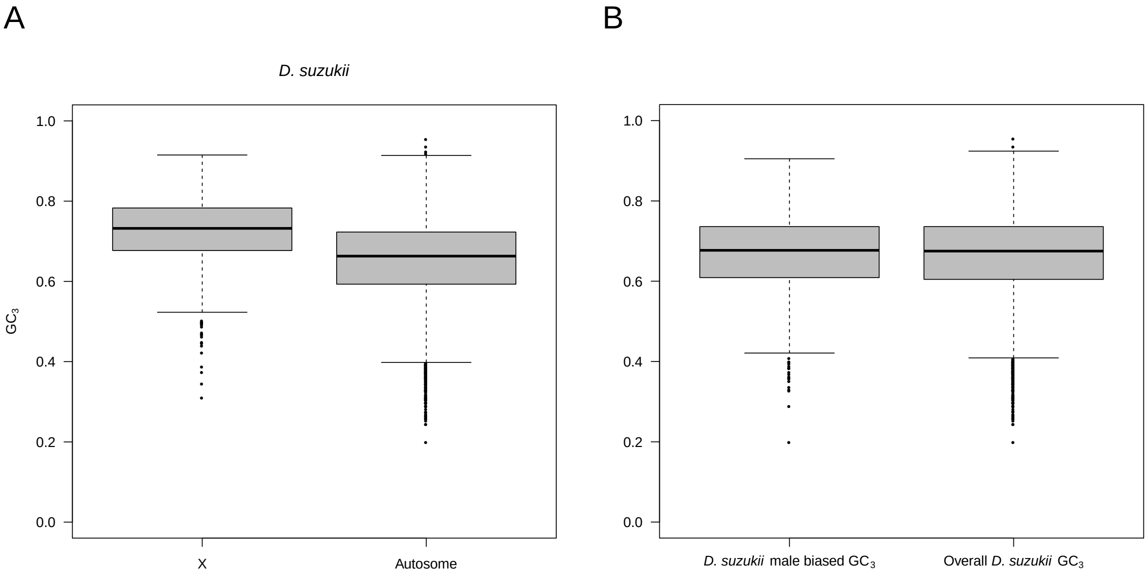

Figure S4 (A) GC_3 content for *D. suzukii* X and autosomal positions. The two data sets were significantly different (Mann-Whitney U test, p = < $2.2e^{-16}$). (B) GC_3 content for *Drosophila suzukii* male biased genes (left) and the overall *D. suzukii* GC_3 content. The two data sets were not significantly different (Mann-Whitney U test, p = 0.706).
