# Supplementary material for: Genome of Drosophila suzukii, the Spotted Wing Drosophila
Source: G3 (Bethesda). 2013 Oct 18;3(12):2257–71. doi: 10.1534/g3.113.008185 (PMC3852387; doi:10.1534/g3.113.008185)
Supplement: Supporting Information [file supp_g3.113.008185_TableS1.pdf]

Table S1   Data production for *Drosophila suzukii* genome sequencing.

INSERT SIZE (BP)	TOTAL DATA (GB)	READ LENGTH	SEQUENCE COVERAGE*	PHYSICAL COVERAGE
250	9.35	150	42.49	37.94
300	4.50	146	20.47	36.26
500	6.64	90	30.19	88.79
2k	5.99	49	27.22	555.55
9k	0.94	160	4.27	284.35
10k	9.58	49	43.55	4443.37
20k	14.45	49	65.67	13402.20

*Genome size was estimated at 220Mbp
