# Supplementary material for: Genome of Drosophila suzukii, the Spotted Wing Drosophila
Source: G3 (Bethesda). 2013 Oct 18;3(12):2257–71. doi: 10.1534/g3.113.008185 (PMC3852387; doi:10.1534/g3.113.008185)
Supplement: Supporting Information [file supp_g3.113.008185_TableS2.pdf]

Table S2 ***Drosophila suzukii*** **17-k-mer statistics.**

SPECIES	K-MER	K-MER NUM	K-MER DEPTH	GENOME SIZE (BP)	SEQUENCE COVERAGE
D. suzukii	17	5,515,021,508	25	220,600,860	30.07
