# Supplementary material for: Genome of Drosophila suzukii, the Spotted Wing Drosophila
Source: G3 (Bethesda). 2013 Oct 18;3(12):2257–71. doi: 10.1534/g3.113.008185 (PMC3852387; doi:10.1534/g3.113.008185)
Supplement: Supporting Information [file supp_g3.113.008185_TableS3.pdf]

**Table S3 Statistics of the assembled genome.**

| | CONTIG | | SCAFFOLD | |
|---|---|---|---|---|
| | LENGTH (BP) | NUMBER | LENGTH (BP) | NUMBER |
| N90 | 3,736 | 10,573 | 39,620 | 812 |
| N80 | 7,638 | 6,817 | 102,455 | 446 |
| N70 | 11,943 | 4,678 | 163,657 | 267 |
| N60 | 16,903 | 3,230 | 254,714 | 151 |
| N50 | 23,216 | 2,194 | 385,236 | 73 |
| Total Size | 204,921,988 | | 235,559,420 | |
| Longest | 472,152 | | 22,559,587 | |
| Total Number (>100bp) | | 46,329 | | 29,113 |
| Total Number (>2kb) | | 14,013 | | 2,723 |
