# Supplementary material for: Genome of Drosophila suzukii, the Spotted Wing Drosophila
Source: G3 (Bethesda). 2013 Oct 18;3(12):2257–71. doi: 10.1534/g3.113.008185 (PMC3852387; doi:10.1534/g3.113.008185)
Supplement: Supporting Information [file supp_g3.113.008185_TableS4.pdf]

Table S4 A list of 25 genes in *Drosophila suzukii* with the highest non-synonymous (dN) substitution rates.

FLYBASE_ID OF *D. MEL* ORTHOLOG	LOCATION OF *D. MEL* ORTHOLOG	GENE NAME OF *D. ME*L ORTHOLOG	MOLECULAR FUNCTION
FBpp0300230	X	CG43386	function unknown
FBpp0292981	2L	CG42848	function unknown
FBpp0292728	3R	CG34006-PB	function unknown
FBpp0077038	X	CG14619-PE	ubiquitin-specific protease activity
FBpp0083206	3R	branchless-PA	fibroblast growth factor receptor binding
FBpp0075387	3L	CTPsyn-PC	CTP synthase activity
FBpp0075087	3L	nudC-PA	nuclear migration
FBpp0289636	3R	CG34034-PB	function unknown
FBpp0080350	2L	CG13244-PA	function unknown
FBpp0076948	X	CG14579-PA	function unknown
FBpp0080236	2L	CG33309-PA	function unknown
FBpp0293396	2L	bruno-2-PE	RNA/mRNA binding
FBpp0087089	2R	CG13186-PA	function unknown
FBpp0099816	4	Dyrk3-PA	protein serine/threonine kinase activity
FBpp0297068	3L	CG43168-PA	function unknown
FBpp0082217	3R	CheA87a-PA	function unknown
FBpp0075635	3L	CG10752-PA	function unknown
FBpp0111417	3R	CG34308-PA	zinc ion binding
FBpp0289540	2R	Sfp53D-PA	multicellular organism reproduction
FBpp0073917	X	CG8565-PA	protein kinase activity
FBpp0075636	3L	CG32115-PA	function unknown
FBpp0073239	X	CG15208-PA	function unknown (sperm competition)
FBpp0110475	2L	CG17490-PA	function unknown
FBpp0081175	3R	CG15185-PA	function unknown
FBpp0083140	3R	CG14294-PA	function unknown
