# Supplementary material for: Genome of Drosophila suzukii, the Spotted Wing Drosophila
Source: G3 (Bethesda). 2013 Oct 18;3(12):2257–71. doi: 10.1534/g3.113.008185 (PMC3852387; doi:10.1534/g3.113.008185)
Supplement: Supporting Information [file supp_g3.113.008185_TableS5.pdf]

Table S5 A list of *Drosophila suzukii* genes with sex-biased expression pattern.

GENE	GENE SYMBOL	LOCUS	FEMALE EXPRESSION	MALE EXPRESSION	SEX-BIAS
DS10_00000010		scaffold1:50412-58902	0.95	6.56	male
DS10_00000019		scaffold1:138485-171077	0.03	26.59	male
DS10_00000024		scaffold1:208563-211507	0.18	67.75	male
DS10_00000034		scaffold1:254095-298173	0.02	67.56	male
DS10_00000041		scaffold1:360867-361911	5.54	0.84	female
DS10_00000058	rempA	scaffold1:515827-521458	0.46	3.54	male
DS10_00000074	Hsp60B	scaffold1:697183-699130	0.02	108.25	male
DS10_00000078		scaffold1:761457-763179	2.10	15.50	male
DS10_00000099		scaffold1:871629-881205	0.03	5.99	male
DS10_00000105		scaffold1:1102514-1103180	0.00	79.89	male
DS10_00000122	fred	scaffold1:1473153-1521996	0.51	12.23	male
DS10_00000126	Cep97	scaffold1:1572523-1589908	3.72	29.93	male
DS10_00000144	cutlet	scaffold1:1765690-1770175	2.29	0.16	female
DS10_00000165		scaffold1:1986921-1987956	0.00	31.00	male
DS10_00000167		scaffold1:2014864-2015641	0.05	352.66	male
DS10_00000169	CSN1a	scaffold1:2018789-2019899	0.00	31.63	male
DS10_00000170		scaffold1:2022179-2023328	0.00	34.89	male
DS10_00000174		scaffold1:2063231-2066173	0.00	53.04	male
DS10_00000175		scaffold1:2067012-2069688	0.00	21.41	male
DS10_00000179	Mst35Ba	scaffold1:2148730-2149477	0.00	195.63	male
DS10_00000182		scaffold1:2198872-2205568	0.01	6.42	male
DS10_00000211	yuri	scaffold1:2569244-2576794	10.59	41.74	male
DS10_00000212	Cul-3	scaffold1:2579423-2584418	13.58	67.34	male
DS10_00000215		scaffold1:2602592-2608625	0.00	44.94	male
DS10_00000217		scaffold1:2615357-2616662	0.03	9.53	male
DS10_00000220		scaffold1:2690357-2690957	0.00	33.39	male
DS10_00000222		scaffold1:2773177-2774635	0.00	37.67	male
DS10_00000224	Tim17b2	scaffold1:2823377-2823911	0.00	76.72	male
DS10_00000238		scaffold1:3079851-3086635	0.01	2.41	male
DS10_00000254		scaffold1:3267469-3268920	0.65	24.52	male
DS10_00000255		scaffold1:3269899-3272212	0.79	46.01	male
DS10_00000266		scaffold1:3440769-3441051	41.70	0.64	female
DS10_00000269		scaffold1:3445463-3448095	2.52	36.37	male
DS10_00000273	SP555	scaffold1:3457514-3459842	2.32	21.30	male
DS10_00000294		scaffold1:3560513-3571213	0.02	18.79	male
DS10_00000296		scaffold1:3605345-3606320	0.00	44.96	male
DS10_00000304		scaffold1:3722034-3725787	0.16	4.83	male
DS10_00000307		scaffold1:3740175-3749073	0.03	4.94	male
DS10_00000309		scaffold1:3779516-3780587	0.16	31.41	male
DS10_00000318		scaffold1:3876299-3877209	0.58	6.62	male
DS10_00000319		scaffold1:3886337-3886889	0.10	64.44	male
DS10_00000320	robl22E	scaffold1:3894132-3899177	29.35	3.36	female
DS10_00000322		scaffold1:3902887-3903463	0.00	73.76	male
DS10_00000326		scaffold1:3931791-3932414	0.00	69.84	male
DS10_00000328		scaffold1:3946326-3952310	3.07	0.05	female
DS10_00000330	Tengl2	scaffold1:3966381-3969940	0.00	25.88	male
DS10_00000347	d	scaffold1:4098412-4112931	0.30	2.93	male
DS10_00000359		scaffold1:4188097-4189399	0.22	46.49	male
DS10_00000361		scaffold1:4192145-4197064	0.04	52.21	male
DS10_00000390	Spn53F	scaffold1:4313749-4318843	0.02	71.31	male
DS10_00000393	Spn88Ea	scaffold1:4381235-4384495	0.00	140.71	male

DS10_00000394		scaffold1:4385278-4408009	6.21	54.38	male
DS10_00000397		scaffold1:4426946-4427808	0.33	42.64	male
DS10_00000430		scaffold1:4560916-4565048	0.01	19.69	male
DS10_00000431		scaffold1:4576587-4578798	0.00	9.38	male
DS10_00000433	Cpr31A	scaffold1:4607767-4609822	0.07	2.38	male
DS10_00000434	Pen	scaffold1:4611336-4614571	46.77	452.30	male
DS10_00000441	Sur	scaffold1:4756455-4777707	1.48	7.07	male
DS10_00000454	me31B	scaffold1:4827568-4830580	163.78	44.71	female
DS10_00000479		scaffold1:5150721-5151760	0.10	36.62	male
DS10_00000481		scaffold1:5161289-5161816	0.00	102.11	male
DS10_00000483		scaffold1:5232015-5234980	0.02	30.76	male
DS10_00000490		scaffold1:5326075-5326591	0.28	7.90	male
DS10_00000491		scaffold1:5327006-5327508	0.58	10.16	male
DS10_00000512		scaffold1:5462863-5464890	0.72	32.82	male
DS10_00000521		scaffold1:5539688-5540267	0.15	65.05	male
DS10_00000533		scaffold1:5668931-5669615	0.00	52.07	male
DS10_00000541		scaffold1:5854926-5876637	1.24	7.14	male
DS10_00000576	Ts	scaffold1:6127436-6128402	16.39	2.45	female
DS10_00000586		scaffold1:6242755-6249255	0.55	131.66	male
DS10_00000590		scaffold1:6276938-6289732	0.39	6.08	male
DS10_00000596		scaffold1:6327789-6329442	0.00	34.78	male
DS10_00000600		scaffold1:6367817-6368950	0.74	4.98	male
DS10_00000611		scaffold1:6398665-6400298	0.21	8.39	male
DS10_00000618		scaffold1:6494012-6495069	0.04	22.05	male
DS10_00000619	Prosbeta4R2	scaffold1:6503497-6504261	0.00	39.13	male
DS10_00000623		scaffold1:6513863-6518058	0.00	19.66	male
DS10_00000636		scaffold1:6620414-6629074	326.70	67.07	female
DS10_00000644		scaffold1:6692924-6694037	0.04	13.10	male
DS10_00000662		scaffold1:6875129-6881940	6.03	37.22	male
DS10_00000666		scaffold1:6924216-6928845	0.00	32.00	male
DS10_00000669		scaffold1:6964797-6974972	0.01	45.62	male
DS10_00000689		scaffold1:7206514-7207146	0.11	313.63	male
DS10_00000690		scaffold1:7211637-7212237	0.11	214.16	male
DS10_00000703		scaffold1:7279997-7281056	0.00	26.81	male
DS10_00000706		scaffold1:7326855-7328232	0.27	29.76	male
DS10_00000711		scaffold1:7414311-7420219	0.19	5.51	male
DS10_00000714	Iris	scaffold1:7442050-7444144	17.88	0.12	female
DS10_00000731		scaffold1:7524992-7525553	0.00	73.44	male
DS10_00000737		scaffold1:7678680-7679566	0.00	28.80	male
DS10_00000739	ctp	scaffold1:7690085-7690355	0.51	61.81	male
DS10_00000740		scaffold1:7698220-7702443	0.00	31.86	male
DS10_00000742		scaffold1:7718971-7719909	0.05	20.21	male
DS10_00000755		scaffold1:7970935-7973109	0.00	107.67	male
DS10_00000771		scaffold1:8206137-8206524	0.15	28.68	male
DS10_00000776		scaffold1:8284525-8286547	0.00	10.72	male
DS10_00000778	c-cup	scaffold1:8299122-8299783	0.00	32.55	male
DS10_00000781		scaffold1:8331692-8356503	0.16	89.58	male
DS10_00000786		scaffold1:8435102-8435919	0.08	7.76	male
DS10_00000787		scaffold1:8435967-8437123	0.08	5.73	male
DS10_00000795	Obp22a	scaffold1:8495826-8496246	0.00	225.51	male
DS10_00000805		scaffold1:8621325-8622156	0.00	52.20	male
DS10_00000807		scaffold1:8633007-8633674	0.00	73.13	male
DS10_00000810		scaffold1:8651876-8657774	0.87	13.31	male
DS10_00000822		scaffold1:8745371-8746992	0.00	28.47	male

DS10_00000832		scaffold1:8829866-8830802	0.11	10.52	male
DS10_00000835		scaffold1:8836938-8837589	0.00	198.78	male
DS10_00000836	Sfp38D	scaffold1:8838750-8840272	0.00	113.15	male
DS10_00000838		scaffold1:8864471-8865227	0.11	43.34	male
DS10_00000841		scaffold1:8940690-8941319	0.18	60.10	male
DS10_00000845		scaffold1:9020037-9020408	0.25	40.43	male
DS10_00000873		scaffold1:9449321-9449744	0.63	14.92	male
DS10_00000876	ms(2)34Fe	scaffold1:9504184-9509762	0.09	29.23	male
DS10_00000888		scaffold1:9608095-9608759	0.00	38.01	male
DS10_00000893		scaffold1:9754813-9755793	0.05	111.14	male
DS10_00000894	lwr	scaffold1:9772060-9772516	0.49	35.85	male
DS10_00000909		scaffold1:9949655-9955048	0.03	266.58	male
DS10_00000912	Skadu	scaffold1:10012026-10012794	0.00	35.60	male
DS10_00000913		scaffold1:10015591-10017479	0.03	36.26	male
DS10_00000916		scaffold1:10078900-10080540	0.00	37.41	male
DS10_00000926	CycE	scaffold1:10162392-10174917	6.56	1.12	female
DS10_00000936	Tektin-A	scaffold1:10294199-10298428	0.00	78.92	male
DS10_00000938		scaffold1:10323809-10326346	0.00	14.13	male
DS10_00000940		scaffold1:10341650-10343710	0.00	36.49	male
DS10_00000943		scaffold1:10365261-10366281	0.15	30.74	male
DS10_00000945		scaffold1:10393533-10395188	0.00	43.02	male
DS10_00000946		scaffold1:10411396-10412901	0.00	15.03	male
DS10_00000949		scaffold1:10461604-10467818	0.00	554.21	male
DS10_00000956		scaffold1:10691521-10693877	0.00	45.27	male
DS10_00000978		scaffold1:10818901-10825709	0.35	15.33	male
DS10_00000988		scaffold1:10935831-10936547	4.31	30.47	male
DS10_00001032	tj	scaffold1:11444035-11445586	8.28	0.72	female
DS10_00001060	del	scaffold1:11815869-11819758	13.23	1.56	female
DS10_00001071	TfIIA-S-2	scaffold1:11860444-11860983	0.13	9.85	male
DS10_00001072		scaffold1:11872440-11872977	0.00	404.40	male
DS10_00001073		scaffold1:11876513-11877122	0.07	2.61	male
DS10_00001090		scaffold1:12052001-12053291	0.00	22.63	male
DS10_00001092	qjt	scaffold1:12109369-12110145	0.12	3.59	male
DS10_00001093		scaffold1:12150392-12151253	0.00	73.95	male
DS10_00001097		scaffold1:12236965-12237340	1.93	26.55	male
DS10_00001104		scaffold1:12372747-12373632	0.00	73.37	male
DS10_00001111		scaffold1:12513654-12516235	0.16	24.24	male
DS10_00001114		scaffold1:12540671-12542563	0.05	19.84	male
DS10_00001117		scaffold1:12564943-12571070	0.03	27.51	male
DS10_00001125	qua	scaffold1:12603321-12608905	4.70	43.79	male
DS10_00001127		scaffold1:12617564-12619432	23.46	102.92	male
DS10_00001128	Dhc36C	scaffold1:12620587-12656720	0.09	12.89	male
DS10_00001129		scaffold1:12620587-12656720	0.03	11.69	male
DS10_00001131		scaffold1:12682644-12683862	0.00	40.50	male
DS10_00001143		scaffold1:13147534-13149973	0.00	11.32	male
DS10_00001147		scaffold1:13174291-13176011	0.00	257.07	male
DS10_00001148	elfless	scaffold1:13177214-13177938	0.30	5.92	male
DS10_00001152		scaffold1:13265086-13267501	0.26	7.04	male
DS10_00001153		scaffold1:13272518-13273850	0.00	30.88	male
DS10_00001163		scaffold1:13379551-13408856	1.10	6.19	male
DS10_00001167		scaffold1:13500684-13502643	0.02	63.10	male
DS10_00001173		scaffold1:13620705-13624622	0.00	27.68	male
DS10_00001174	l(1)G0334	scaffold1:13667341-13668909	0.00	15.90	male
DS10_00001195		scaffold1:13919916-13930867	0.12	13.06	male

DS10_00001223		scaffold1:14235658-14236571	0.17	39.42	male
DS10_00001239	robl37BC	scaffold1:14308370-14308715	0.16	26.50	male
DS10_00001256		scaffold1:14408948-14410365	0.04	115.15	male
DS10_00001258		scaffold1:14423600-14425226	11.40	62.62	male
DS10_00001267		scaffold1:14488208-14488361	1.26	314.63	male
DS10_00001268	gammaTub37C	scaffold1:14489987-14492075	14.28	0.28	female
DS10_00001271		scaffold1:14579294-14581436	0.00	7.50	male
DS10_00001272		scaffold1:14622591-14624394	0.00	54.81	male
DS10_00001273		scaffold1:14624751-14626123	0.00	49.21	male
DS10_00001276		scaffold1:14695981-14698503	0.08	74.05	male
DS10_00001281		scaffold1:14725329-14727470	0.24	12.88	male
DS10_00001293		scaffold1:14824400-14825954	0.39	364.35	male
DS10_00001294		scaffold1:14826622-14829529	0.16	159.44	male
DS10_00001298		scaffold1:14864839-14867093	0.00	47.88	male
DS10_00001299	Acp26Ab	scaffold1:14867412-14868010	0.12	257.75	male
DS10_00001300		scaffold1:14875396-14875809	1.95	956.93	male
DS10_00001302	Sfp26Ac	scaffold1:14877644-14878083	0.00	90.49	male
DS10_00001305	TrissinR	scaffold1:14909677-14923768	1.92	23.48	male
DS10_00001306		scaffold1:14945461-14946244	0.00	96.49	male
DS10_00001307		scaffold1:14953862-14954066	1.79	137.78	male
DS10_00001312		scaffold1:14989314-14989959	0.06	74.04	male
DS10_00001319	sip1	scaffold1:15061338-15063975	1.83	22.93	male
DS10_00001325	Hsp60C	scaffold1:15128605-15143614	0.00	102.00	male
DS10_00001329		scaffold1:15225682-15227332	0.00	57.22	male
DS10_00001330		scaffold1:15238464-15240060	0.03	60.09	male
DS10_00001332		scaffold1:15285554-15286617	0.04	19.39	male
DS10_00001346	dtr	scaffold1:15435401-15441851	0.29	19.95	male
DS10_00001353		scaffold1:15486208-15487423	0.00	231.84	male
DS10_00001354		scaffold1:15489924-15490083	1.07	571.71	male
DS10_00001355		scaffold1:15491173-15491335	0.00	540.95	male
DS10_00001361		scaffold1:15562014-15564233	0.00	107.38	male
DS10_00001371		scaffold1:15826132-15827524	0.00	29.30	male
DS10_00001374		scaffold1:15844543-15845443	0.00	17.97	male
DS10_00001375	Su(var)205	scaffold1:15913446-15914124	1.22	9.07	male
DS10_00001376		scaffold1:15927913-15930104	0.02	26.94	male
DS10_00001377		scaffold1:15952696-15954613	0.07	24.84	male
DS10_00001381		scaffold1:15983489-15986459	0.06	19.34	male
DS10_00001383		scaffold1:16001653-16002351	0.00	44.41	male
DS10_00001386		scaffold1:16031692-16032325	0.11	124.26	male
DS10_00001387		scaffold1:16033625-16034100	0.00	668.15	male
DS10_00001402		scaffold1:16096964-16098535	0.15	70.53	male
DS10_00001420		scaffold1:16306743-16308247	0.06	7.55	male
DS10_00001431		scaffold1:16349206-16351153	4.82	0.79	female
DS10_00001441	nop5	scaffold1:16394749-16396611	74.26	17.56	female
DS10_00001451		scaffold1:16527878-16529966	0.14	16.40	male
DS10_00001453		scaffold1:16532317-16536999	0.00	205.72	male
DS10_00001456		scaffold1:16564774-16568840	4.80	28.25	male
DS10_00001460		scaffold1:16600280-16601277	0.29	48.62	male
DS10_00001463	Liprin-alpha	scaffold1:16613343-16625514	5.74	31.34	male
DS10_00001467	Tsp	scaffold1:16644357-16672183	6.97	36.11	male
DS10_00001468		scaffold1:16672782-16673421	0.00	31.04	male
DS10_00001473	Pcp	scaffold1:16714274-16723749	0.22	2.61	male
DS10_00001479		scaffold1:16745773-16775279	0.07	33.71	male
DS10_00001505		scaffold1:17172952-17175907	0.11	18.35	male

DS10_00001518		scaffold1:17279585-17303868	0.15	69.12	male
DS10_00001520	Spn28B	scaffold1:17403108-17404425	0.00	48.84	male
DS10_00001521	Spn88Ea	scaffold1:17420901-17423658	0.00	65.68	male
DS10_00001533		scaffold1:17580913-17618475	0.11	41.14	male
DS10_00001534		scaffold1:17619523-17634956	0.48	23.18	male
DS10_00001542		scaffold1:17685688-17693475	0.01	9.52	male
DS10_00001545		scaffold1:17703076-17715202	0.31	93.87	male
DS10_00001551		scaffold1:17745852-17746755	0.00	42.01	male
DS10_00001562	lectin-30A	scaffold1:18020862-18021946	0.07	44.15	male
DS10_00001565		scaffold1:18106884-18109374	0.04	17.08	male
DS10_00001568		scaffold1:18124136-18125555	0.00	40.04	male
DS10_00001569		scaffold1:18132962-18133364	0.00	73.65	male
DS10_00001570	Acp24A4	scaffold1:18134499-18134814	0.00	228.74	male
DS10_00001577	Fbp2	scaffold1:18225807-18227923	0.26	36.84	male
DS10_00001578	FucTB	scaffold1:18228101-18230576	3.96	30.48	male
DS10_00001580		scaffold1:18235757-18237598	0.49	3.94	male
DS10_00001593	gcm	scaffold1:18422669-18423494	0.07	220.99	male
DS10_00001604	hoip	scaffold1:18562625-18563276	113.13	26.02	female
DS10_00001635		scaffold1:18861158-18866815	4.00	76.64	male
DS10_00001645	Prosalpha6T	scaffold1:18898224-18899094	0.05	19.14	male
DS10_00001646		scaffold1:18899594-18901151	0.32	33.62	male
DS10_00001659		scaffold1:18975384-18981414	0.19	30.67	male
DS10_00001660	Ucp4C	scaffold1:18985266-18986082	0.00	24.42	male
DS10_00001676		scaffold1:19094745-19095327	0.00	32.90	male
DS10_00001677		scaffold1:19095953-19098291	0.02	38.60	male
DS10_00001679	Vm34Ca	scaffold1:19107521-19107878	105.17	0.28	female
DS10_00001685	Hsp60D	scaffold1:19149544-19151230	0.00	50.76	male
DS10_00001687		scaffold1:19154998-19155340	0.18	7.51	male
DS10_00001694		scaffold1:19223474-19227356	0.03	23.11	male
DS10_00001696		scaffold1:19281760-19283209	0.03	70.71	male
DS10_00001699		scaffold1:19341351-19342922	0.03	13.86	male
DS10_00001702		scaffold1:19403488-19406038	0.00	61.89	male
DS10_00001703		scaffold1:19426112-19427569	0.00	53.76	male
DS10_00001740		scaffold1:19808177-19808696	0.00	80.58	male
DS10_00001742		scaffold1:19842949-19851626	103.58	29.62	female
DS10_00001744		scaffold1:19904436-19905688	0.04	19.25	male
DS10_00001764		scaffold1:19996298-19997631	10.29	0.17	female
DS10_00001768		scaffold1:20009781-20011099	14.16	0.23	female
DS10_00001776		scaffold1:20038245-20041243	3.18	42.51	male
DS10_00001783		scaffold1:20084116-20085435	4.45	0.57	female
DS10_00001808		scaffold1:20173961-20182678	8.31	60.69	male
DS10_00001829		scaffold1:20339245-20339857	0.00	42.92	male
DS10_00001830	w-cup	scaffold1:20349641-20351098	0.00	235.62	male
DS10_00001831		scaffold1:20353162-20353759	0.00	116.34	male
DS10_00001832		scaffold1:20354521-20355070	0.00	71.25	male
DS10_00001835		scaffold1:20407272-20421519	0.01	32.39	male
DS10_00001836		scaffold1:20423786-20426596	0.00	37.57	male
DS10_00001838		scaffold1:20454806-20456951	1.89	11.72	male
DS10_00001855		scaffold1:20598695-20599353	0.00	74.35	male
DS10_00001863		scaffold1:20691300-20691723	0.13	2.47	male
DS10_00001864		scaffold1:20704197-20704608	0.13	30.30	male
DS10_00001865		scaffold1:20718861-20719735	0.06	3.42	male
DS10_00001866		scaffold1:20726523-20729044	0.09	34.46	male
DS10_00001867		scaffold1:20730754-20731060	0.42	9.51	male

DS10_00001869		scaffold1:20756901-20762840	0.85	60.76	male
DS10_00001878	piwi	scaffold1:20814284-20824763	28.77	2.86	female
DS10_00001880		scaffold1:20828766-20837432	15.54	57.75	male
DS10_00001881	aub	scaffold1:20838533-20842679	54.97	1.58	female
DS10_00001885	Acp32CD	scaffold1:20862175-20863099	0.09	193.28	male
DS10_00001898		scaffold1:20997871-20999307	0.12	15.97	male
DS10_00001920		scaffold1:21269583-21270266	0.00	360.03	male
DS10_00001922	salr	scaffold1:21298332-21308046	1.29	16.39	male
DS10_00001931		scaffold1:21493839-21496360	0.00	6.80	male
DS10_00001947		scaffold1:21647463-21651035	0.00	7.79	male
DS10_00001948		scaffold1:21654724-21655297	0.00	91.12	male
DS10_00001949		scaffold1:21656050-21657148	0.00	74.19	male
DS10_00001952		scaffold1:21704962-21706981	0.00	43.66	male
DS10_00001973	zuc	scaffold1:22051225-22051987	4.17	0.31	female
DS10_00001990		scaffold1:22154884-22155724	0.10	9.97	male
DS10_00001991	prd	scaffold1:22162179-22164615	0.02	7.24	male
DS10_00002013		scaffold1:22505173-22506657	0.00	49.20	male
DS10_00002016	Pkd2	scaffold1:22552603-22556315	0.09	14.76	male
DS10_00002017		scaffold2:14677-18608	22.54	107.24	male
DS10_00002022		scaffold2:44861-48334	0.02	71.80	male
DS10_00002024	isopeptidase-T-3	scaffold2:63092-65153	4.01	36.50	male
DS10_00002026		scaffold2:73475-74506	0.09	57.83	male
DS10_00002027		scaffold2:78739-81343	0.06	24.00	male
DS10_00002033		scaffold2:158142-161329	0.11	47.38	male
DS10_00002037		scaffold2:206839-207324	0.27	129.44	male
DS10_00002038	sm	scaffold2:213486-234977	13.72	103.43	male
DS10_00002066		scaffold2:463203-467424	0.04	136.96	male
DS10_00002073		scaffold2:533492-535040	0.00	28.54	male
DS10_00002084		scaffold2:688453-688936	0.19	61.61	male
DS10_00002094		scaffold2:769263-775586	0.19	3.18	male
DS10_00002108		scaffold2:956158-957046	0.42	39.14	male
DS10_00002114		scaffold2:1002736-1007223	0.08	18.64	male
DS10_00002132	Obp58b	scaffold2:1157907-1158808	0.07	2.63	male
DS10_00002134		scaffold2:1160298-1171074	0.00	9.36	male
DS10_00002138		scaffold2:1225750-1226934	0.00	17.50	male
DS10_00002139		scaffold2:1252190-1252821	0.00	80.37	male
DS10_00002140		scaffold2:1252987-1254378	0.00	51.82	male
DS10_00002142	CycB	scaffold2:1278946-1281987	66.95	15.91	female
DS10_00002144		scaffold2:1297126-1308431	0.91	4.56	male
DS10_00002161		scaffold2:1411869-1437002	0.60	18.54	male
DS10_00002165		scaffold2:1508562-1509087	0.00	38.40	male
DS10_00002166		scaffold2:1521053-1528920	0.03	37.91	male
DS10_00002178	bgcn	scaffold2:1584278-1589462	1.12	5.97	male
DS10_00002213		scaffold2:1963509-1964529	0.04	24.17	male
DS10_00002223		scaffold2:2004686-2019416	3.02	0.34	female
DS10_00002245		scaffold2:2352575-2354504	0.03	34.59	male
DS10_00002265	Ir60a	scaffold2:2588842-2606040	0.04	3.42	male
DS10_00002270	Nop60B	scaffold2:2684730-2688122	91.11	15.71	female
DS10_00002280		scaffold2:2737032-2745422	2.51	20.12	male
DS10_00002283		scaffold2:2754615-2755200	0.00	82.41	male
DS10_00002312		scaffold2:2857101-2859598	0.29	39.89	male
DS10_00002318		scaffold2:2889840-2893223	0.09	14.09	male
DS10_00002335	CkIIbeta2	scaffold2:2935932-2938050	0.00	30.16	male

DS10_00002341		scaffold2:3002205-3003178	0.00	118.24	male
DS10_00002342		scaffold2:3004181-3007389	0.02	20.23	male
DS10_00002366		scaffold2:3352183-3358727	0.13	23.99	male
DS10_00002373		scaffold2:3369615-3378795	0.13	88.10	male
DS10_00002396		scaffold2:3537316-3547471	0.22	31.73	male
DS10_00002424	MCPH1	scaffold2:3833619-3841465	3.86	60.74	male
DS10_00002428	cuff	scaffold2:3851039-3852659	2.55	0.13	female
DS10_00002472		scaffold2:4443127-4446268	0.00	26.28	male
DS10_00002481		scaffold2:4653208-4654876	0.04	31.65	male
DS10_00002516	Obp50b	scaffold2:4937486-4939507	0.21	18.95	male
DS10_00002546		scaffold2:5230636-5232276	0.00	33.17	male
DS10_00002549	Adgf-E	scaffold2:5253502-5255757	0.00	44.52	male
DS10_00002550		scaffold2:5262702-5263425	0.00	378.41	male
DS10_00002551		scaffold2:5264370-5265216	0.00	370.91	male
DS10_00002552		scaffold2:5266769-5270730	0.04	8.46	male
DS10_00002570		scaffold2:5428369-5430710	0.00	30.21	male
DS10_00002581	wash	scaffold2:5487751-5489248	11.22	54.15	male
DS10_00002592		scaffold2:5570456-5572146	0.17	38.25	male
DS10_00002593	Vha36-2	scaffold2:5574244-5575858	0.25	45.02	male
DS10_00002595		scaffold2:5601014-5601558	0.00	44.39	male
DS10_00002599	SIP2	scaffold2:5626295-5630037	0.06	144.90	male
DS10_00002614		scaffold2:5721125-5725304	0.02	4.80	male
DS10_00002617	Cpr49Ah	scaffold2:5734938-5742886	4.33	0.69	female
DS10_00002661		scaffold2:6212789-6215027	3.96	0.17	female
DS10_00002698	exu	scaffold2:6391016-6393338	39.09	377.65	male
DS10_00002703		scaffold2:6461951-6483313	0.00	52.56	male
DS10_00002724		scaffold2:6713526-6714957	0.06	74.65	male
DS10_00002729		scaffold2:6751461-6754739	0.96	55.75	male
DS10_00002756		scaffold2:6947397-6948342	0.00	61.64	male
DS10_00002759		scaffold2:6953016-6953435	0.54	55.49	male
DS10_00002768		scaffold2:7007243-7007969	1.10	50.82	male
DS10_00002779	sut4	scaffold2:7046925-7048263	0.00	29.12	male
DS10_00002794		scaffold2:7225435-7226843	0.00	24.41	male
DS10_00002795		scaffold2:7227505-7228646	0.22	12.90	male
DS10_00002805		scaffold2:7299587-7300881	0.04	7.25	male
DS10_00002807		scaffold2:7301906-7314120	0.04	3.08	male
DS10_00002809		scaffold2:7351437-7353162	0.09	96.02	male
DS10_00002810		scaffold2:7357118-7357678	0.22	14.75	male
DS10_00002841		scaffold2:7755777-7757827	0.06	40.70	male
DS10_00002843		scaffold2:7763179-7763783	8.25	47.34	male
DS10_00002875		scaffold2:7970176-7987381	3.59	0.61	female
DS10_00002909		scaffold2:8333777-8334477	16.12	90.85	male
DS10_00002913	Mef2	scaffold2:8371710-8372059	2.22	19.83	male
DS10_00002925		scaffold2:8516277-8517081	236.19	0.53	female
DS10_00002926		scaffold2:8517294-8519459	0.03	133.96	male
DS10_00002927		scaffold2:8520292-8521653	33.62	0.05	female
DS10_00002932		scaffold2:8577523-8579121	2.35	73.06	male
DS10_00002942	St1	scaffold2:8639392-8648490	11.98	1.76	female
DS10_00002946		scaffold2:8737882-8739357	0.26	53.04	male
DS10_00002948		scaffold2:8740590-8741487	11.00	55.68	male
DS10_00002949		scaffold2:8741790-8743021	0.06	50.86	male
DS10_00002965		scaffold2:8849835-8850183	0.33	10.71	male
DS10_00002971	skpF	scaffold2:8916367-8916877	0.00	39.26	male
DS10_00002973		scaffold2:8946200-8948457	0.03	5.24	male

DS10_00002993		scaffold2:9082515-9083572	0.10	14.95	male
DS10_00002995		scaffold2:9087818-9088912	0.09	20.95	male
DS10_00002998		scaffold2:9118856-9120032	0.00	45.00	male
DS10_00002999		scaffold2:9125306-9127655	4.52	0.32	female
DS10_00003000		scaffold2:9152044-9153259	0.03	92.42	male
DS10_00003001		scaffold2:9153888-9154608	0.00	187.88	male
DS10_00003005	Crtp	scaffold2:9177672-9180267	0.00	18.94	male
DS10_00003006	Ssl	scaffold2:9183788-9184581	0.00	31.43	male
DS10_00003008	Prosalpha4T2	scaffold2:9187703-9189054	0.00	30.04	male
DS10_00003010		scaffold2:9196654-9197308	0.07	72.46	male
DS10_00003019		scaffold2:9233012-9233456	17.17	2.04	female
DS10_00003026		scaffold2:9273187-9281381	0.07	46.03	male
DS10_00003028		scaffold2:9284143-9304973	0.03	27.69	male
DS10_00003040		scaffold2:9358305-9359743	0.60	96.10	male
DS10_00003046	Pof	scaffold2:9386290-9388019	3.90	110.25	male
DS10_00003086		scaffold2:9778365-9780930	0.95	24.19	male
DS10_00003110		scaffold2:9956399-9958970	0.03	30.97	male
DS10_00003134	krimp	scaffold2:10271865-10275146	7.65	0.43	female
DS10_00003135		scaffold2:10276119-10276801	0.00	79.79	male
DS10_00003180		scaffold2:10575066-10580434	0.00	57.10	male
DS10_00003183		scaffold2:10584357-10585112	0.00	43.47	male
DS10_00003184		scaffold2:10585441-10586581	0.00	33.35	male
DS10_00003185		scaffold2:10587098-10587593	0.00	106.34	male
DS10_00003201		scaffold2:10667547-10672173	0.03	112.61	male
DS10_00003203		scaffold2:10680266-10680833	0.00	70.17	male
DS10_00003205	Menl-2	scaffold2:10739386-10744035	0.00	53.50	male
DS10_00003208		scaffold2:10761093-10762968	0.00	29.52	male
DS10_00003210		scaffold2:10778389-10779478	0.08	23.23	male
DS10_00003215		scaffold2:10828590-10831055	0.35	4.56	male
DS10_00003216	S-Lap8	scaffold2:10833708-10835831	0.02	296.27	male
DS10_00003217		scaffold2:10841384-10843963	0.00	19.82	male
DS10_00003219	loopin-1	scaffold2:10886806-10888390	0.07	541.13	male
DS10_00003238	gom	scaffold2:11004625-11005929	0.00	43.40	male
DS10_00003247	ste24c	scaffold2:11044609-11047994	0.12	26.02	male
DS10_00003249		scaffold2:11091079-11092159	0.00	43.38	male
DS10_00003256		scaffold2:11128303-11128542	0.72	965.34	male
DS10_00003268		scaffold2:11272783-11273952	0.15	22.58	male
DS10_00003269		scaffold2:11274406-11275341	0.36	4.05	male
DS10_00003277	Arp53D	scaffold2:11342746-11344057	0.03	17.27	male
DS10_00003279		scaffold2:11350001-11353087	0.02	8.14	male
DS10_00003281		scaffold2:11354700-11369207	1.06	18.16	male
DS10_00003282	Acp53C14b	scaffold2:11369871-11371158	0.00	37.25	male
DS10_00003284	Sfp53D	scaffold2:11374832-11375273	0.63	79.78	male
DS10_00003285		scaffold2:11380119-11382912	0.00	18.15	male
DS10_00003291		scaffold2:11460518-11462766	0.07	20.37	male
DS10_00003298		scaffold2:11536393-11545983	0.17	355.13	male
DS10_00003300		scaffold2:11547454-11592379	0.00	87.56	male
DS10_00003302		scaffold2:11627269-11628881	0.00	20.00	male
DS10_00003311	Pmm45A	scaffold2:11678895-11680879	33.82	106.28	male
DS10_00003348	l(2)03659	scaffold2:11980019-11984766	0.01	4.19	male
DS10_00003373	clos	scaffold2:12183626-12198411	10.83	1.43	female
DS10_00003384		scaffold2:12356745-12368351	0.39	92.82	male
DS10_00003389	lectin-46Cb	scaffold2:12422928-12426360	2.16	103.49	male
DS10_00003390		scaffold2:12428496-12429098	0.10	56.92	male

DS10_00003403	Ntmt	scaffold2:12475912-12476743	7.43	38.15	male
DS10_00003418	Klp59C	scaffold2:12567992-12570720	0.00	38.41	male
DS10_00003419		scaffold2:12572353-12573698	0.00	41.97	male
DS10_00003421		scaffold2:12585959-12591758	0.02	15.22	male
DS10_00003423	Klp59D	scaffold2:12595094-12596789	0.00	39.60	male
DS10_00003425		scaffold2:12614812-12615814	0.11	3.11	male
DS10_00003427		scaffold2:12642566-12643325	0.07	16.99	male
DS10_00003429		scaffold2:12696861-12697926	0.00	57.86	male
DS10_00003431	RpL22-like	scaffold2:12714142-12715397	0.70	25.09	male
DS10_00003433	ord	scaffold2:12745070-12746900	0.14	10.88	male
DS10_00003434		scaffold2:12758929-12759967	0.27	382.34	male
DS10_00003435		scaffold2:12760314-12760854	0.00	52.97	male
DS10_00003437	Prosbeta5R1	scaffold2:12792841-12793839	0.00	49.91	male
DS10_00003439		scaffold2:12805081-12806002	0.00	57.36	male
DS10_00003440		scaffold2:12808985-12811143	0.00	13.61	male
DS10_00003441		scaffold2:12811492-12814394	0.01	58.07	male
DS10_00003446		scaffold2:12840658-12841384	0.00	27.60	male
DS10_00003453		scaffold2:12885756-12886839	0.07	28.26	male
DS10_00003475	HmgD	scaffold2:12958007-12961514	217.66	59.90	female
DS10_00003477		scaffold2:12962197-12965287	0.62	22.76	male
DS10_00003479	Grx-1	scaffold2:13001839-13002190	0.00	65.53	male
DS10_00003482		scaffold2:13018797-13019605	0.00	130.93	male
DS10_00003483		scaffold2:13020145-13021828	0.00	46.41	male
DS10_00003486		scaffold2:13053998-13054511	0.00	46.16	male
DS10_00003492	PpN58A	scaffold2:13158551-13159502	0.04	34.00	male
DS10_00003509		scaffold2:13373110-13374804	0.10	17.13	male
DS10_00003512		scaffold2:13389962-13391588	15.31	63.36	male
DS10_00003526		scaffold2:13458169-13479805	0.10	35.94	male
DS10_00003531		scaffold2:13496366-13499718	5.75	0.45	female
DS10_00003532		scaffold2:13500615-13511312	9.24	28.96	male
DS10_00003544		scaffold2:13630292-13632020	0.00	25.66	male
DS10_00003546		scaffold2:13687435-13688106	0.00	61.65	male
DS10_00003547		scaffold2:13688801-13690784	0.00	125.84	male
DS10_00003548		scaffold2:13696133-13704226	0.00	116.05	male
DS10_00003551		scaffold2:13716667-13717087	0.00	77.12	male
DS10_00003599	Tpc2	scaffold2:14087333-14124835	0.12	26.03	male
DS10_00003628		scaffold2:14485143-14486316	0.04	24.49	male
DS10_00003633		scaffold3:3970-4296	10.53	0.14	female
DS10_00003643		scaffold3:130284-132529	0.04	105.42	male
DS10_00003669		scaffold3:520047-521010	1.37	15.63	male
DS10_00003676		scaffold3:561848-564347	3.61	20.69	male
DS10_00003698		scaffold3:811404-815716	0.26	18.59	male
DS10_00003703		scaffold3:843637-845034	0.17	42.76	male
DS10_00003706		scaffold3:853625-854789	0.00	26.89	male
DS10_00003708		scaffold3:864009-865698	0.00	38.00	male
DS10_00003710	eIF4E-3	scaffold3:885196-886532	0.09	73.92	male
DS10_00003731	Jon66Ci	scaffold3:1062493-1063276	6.58	0.25	female
DS10_00003734	mkg-p	scaffold3:1068780-1078053	2.79	44.60	male
DS10_00003757		scaffold3:1276479-1295476	1.51	237.25	male
DS10_00003784		scaffold3:1523041-1525246	0.07	50.91	male
DS10_00003786		scaffold3:1566250-1581393	2.41	59.75	male
DS10_00003787	Mcm7	scaffold3:1582368-1584870	13.14	1.53	female
DS10_00003792	orb2	scaffold3:1638581-1645965	7.37	60.99	male
DS10_00003795		scaffold3:1659214-1664311	0.92	18.61	male

DS10_00003810		scaffold3:1755067-1756240	3.01	76.29	male
DS10_00003817	bol	scaffold3:1809475-1816010	35.12	145.68	male
DS10_00003824		scaffold3:1921152-1924790	16.64	2.19	female
DS10_00003825		scaffold3:1945648-1946956	0.03	6.71	male
DS10_00003826		scaffold3:1962882-1964118	0.00	23.68	male
DS10_00003838	Hsp67Bb	scaffold3:2100761-2101624	2.08	34.12	male
DS10_00003875		scaffold3:2328935-2330078	0.00	40.06	male
DS10_00003877		scaffold3:2331635-2332508	0.26	43.39	male
DS10_00003884	alphaTub67C	scaffold3:2413121-2416756	34.86	2.35	female
DS10_00003886		scaffold3:2417963-2428980	2.22	56.69	male
DS10_00003887	scramb1	scaffold3:2432895-2434590	9.24	0.31	female
DS10_00003889		scaffold3:2437681-2441083	0.80	26.99	male
DS10_00003926		scaffold3:2912658-2914352	0.00	19.81	male
DS10_00003928		scaffold3:2937230-2947723	0.04	12.27	male
DS10_00003929		scaffold3:2956405-2957363	0.00	37.52	male
DS10_00003931		scaffold3:3010879-3012227	0.03	27.00	male
DS10_00003932		scaffold3:3014002-3017008	0.06	2.60	male
DS10_00003933	scramb1	scaffold3:3017248-3018082	0.07	42.24	male
DS10_00003935	Kap-alpha3	scaffold3:3042723-3044253	0.00	18.51	male
DS10_00003939		scaffold3:3239393-3240938	0.00	55.79	male
DS10_00003943		scaffold3:3294024-3294672	0.00	85.39	male
DS10_00003944	S-Lap3	scaffold3:3296715-3298329	0.00	253.12	male
DS10_00003946	S-Lap4	scaffold3:3328872-3330447	0.10	382.76	male
DS10_00003947		scaffold3:3404623-3405340	0.00	62.93	male
DS10_00003957		scaffold3:3492803-3495020	21.46	238.76	male
DS10_00003959		scaffold3:3496070-3503076	0.26	36.88	male
DS10_00003975		scaffold3:3803732-3807680	0.20	75.28	male
DS10_00003990		scaffold3:3925417-3926032	0.00	59.92	male
DS10_00003994		scaffold3:4003459-4004089	0.00	47.96	male
DS10_00003995		scaffold3:4014490-4015640	0.09	49.78	male
DS10_00004016		scaffold3:4642024-4642762	0.11	2.12	male
DS10_00004031		scaffold3:4764032-4767761	3.11	27.28	male
DS10_00004043		scaffold3:4950615-4951680	0.36	83.25	male
DS10_00004044		scaffold3:4958614-4959515	0.79	189.26	male
DS10_00004065		scaffold3:5345419-5345843	0.00	134.45	male
DS10_00004071		scaffold3:5430847-5432310	0.52	257.58	male
DS10_00004074		scaffold3:5455137-5455668	0.00	36.64	male
DS10_00004076		scaffold3:5482391-5483522	0.05	11.53	male
DS10_00004084	gnu	scaffold3:5556233-5557030	24.15	0.00	female
DS10_00004091	Ocho	scaffold3:5735391-5735841	0.62	5.00	male
DS10_00004100	Rpn12R	scaffold3:5795805-5796594	0.00	25.99	male
DS10_00004113	lectin-24Db	scaffold3:5882521-5895209	0.06	10.56	male
DS10_00004117	toe	scaffold3:5942906-5953364	1.62	14.73	male
DS10_00004146	Lsp2	scaffold3:6271262-6273563	68.00	2.34	female
DS10_00004176		scaffold3:6571749-6580674	0.11	22.33	male
DS10_00004183		scaffold3:6618227-6619136	0.28	5.07	male
DS10_00004192	Sfp65A	scaffold3:6749117-6749841	0.00	100.03	male
DS10_00004200	ndl	scaffold3:6995291-7004595	2.20	0.07	female
DS10_00004201		scaffold3:7005218-7010898	16.13	84.66	male
DS10_00004202	zpg	scaffold3:7005218-7010898	26.94	1.13	female
DS10_00004203		scaffold3:7011712-7023326	5.68	26.13	male
DS10_00004205	eIF4E-4	scaffold3:7075080-7076505	0.00	38.52	male
DS10_00004214		scaffold3:7180709-7187875	1.03	23.87	male
DS10_00004227		scaffold3:7414409-7417358	0.13	14.39	male

DS10_00004229		scaffold3:7446778-7446943	0.98	111.62	male
DS10_00004243		scaffold3:7582952-7586504	0.00	40.41	male
DS10_00004245		scaffold3:7589686-7601271	0.04	84.38	male
DS10_00004254		scaffold3:7639978-7640666	0.26	22.30	male
DS10_00004267		scaffold3:7732415-7734430	0.00	23.79	male
DS10_00004273	chb	scaffold3:7762500-7766979	10.81	45.18	male
DS10_00004287		scaffold3:7940271-7941250	0.15	6.44	male
DS10_00004289		scaffold3:7959920-7983629	0.01	16.15	male
DS10_00004292		scaffold3:7991999-8017925	0.22	52.29	male
DS10_00004295	Sfp77F	scaffold3:8051831-8052519	0.00	54.16	male
DS10_00004304	Mst77F	scaffold3:8143803-8145052	0.28	265.62	male
DS10_00004311	Six4	scaffold3:8172273-8176484	4.23	0.42	female
DS10_00004332		scaffold3:8487526-8493511	0.14	7.27	male
DS10_00004339		scaffold3:8521059-8521685	0.20	25.88	male
DS10_00004349	aly	scaffold3:8575113-8576973	0.02	2.07	male
DS10_00004350		scaffold3:8586099-8588716	0.00	14.86	male
DS10_00004351	prominin-like	scaffold3:8589634-8594144	0.00	36.39	male
DS10_00004353		scaffold3:8615183-8615784	0.00	75.51	male
DS10_00004359		scaffold3:8646824-8672958	0.67	9.38	male
DS10_00004369		scaffold3:8937926-8978121	0.41	20.59	male
DS10_00004383		scaffold3:9125068-9126202	0.04	22.82	male
DS10_00004393		scaffold3:9263724-9264339	0.00	40.81	male
DS10_00004394		scaffold3:9264423-9264882	0.16	44.42	male
DS10_00004413		scaffold3:9471216-9472590	0.00	15.16	male
DS10_00004420		scaffold3:9655112-9656033	0.00	93.92	male
DS10_00004425		scaffold3:9730176-9731430	0.10	11.07	male
DS10_00004428		scaffold3:9738367-9753236	7.23	22.17	male
DS10_00004443		scaffold3:9859877-9864038	3.13	0.10	female
DS10_00004464		scaffold3:9995826-10014984	0.04	31.51	male
DS10_00004485		scaffold3:10123194-10136277	1.09	104.64	male
DS10_00004486		scaffold3:10136518-10138428	1.04	23.82	male
DS10_00004497		scaffold3:10201058-10208612	0.52	7.50	male
DS10_00004503		scaffold3:10259956-10260246	0.71	6.39	male
DS10_00004505	robl62A	scaffold3:10263879-10267183	0.39	78.53	male
DS10_00004506		scaffold3:10271046-10271688	0.00	33.05	male
DS10_00004509		scaffold3:10321354-10322659	0.00	48.31	male
DS10_00004511	FucTD	scaffold3:10327778-10329158	0.00	39.00	male
DS10_00004531		scaffold3:10514367-10518986	0.04	208.44	male
DS10_00004532		scaffold3:10519376-10520132	0.00	71.02	male
DS10_00004542	LysP	scaffold3:10553364-10553787	303.45	16.01	female
DS10_00004543	LysP	scaffold3:10556685-10557108	147.09	7.08	female
DS10_00004561		scaffold3:10886124-10890330	0.43	10.92	male
DS10_00004594	Ppm1	scaffold3:11236162-11237224	0.00	36.18	male
DS10_00004598		scaffold3:11295584-11297193	0.00	38.10	male
DS10_00004600		scaffold3:11400901-11401375	0.19	54.56	male
DS10_00004602		scaffold3:11468934-11469426	1.24	34.53	male
DS10_00004605	Roc1b	scaffold3:11474304-11477707	0.56	39.91	male
DS10_00004612		scaffold3:11593288-11593873	0.00	51.82	male
DS10_00004615	Dic61B	scaffold3:11697763-11700358	0.06	12.39	male
DS10_00004644		scaffold3:11881758-11903540	0.11	3.87	male
DS10_00004650		scaffold4:57260-58349	0.90	15.65	male
DS10_00004653		scaffold4:100959-101557	0.00	67.34	male
DS10_00004669	HLH3B	scaffold4:304317-307971	0.24	22.56	male
DS10_00004685		scaffold4:411913-415347	0.12	15.26	male

DS10_00004691	SPR	scaffold4:556439-565963	20.58	2.05	female
DS10_00004701		scaffold4:752316-753305	0.05	4.03	male
DS10_00004711		scaffold4:902744-904694	0.22	53.59	male
DS10_00004715		scaffold4:932896-933499	0.00	27.11	male
DS10_00004727	Caf1-180	scaffold4:1076950-1080959	8.43	1.82	female
DS10_00004732	Oxp	scaffold4:1114962-1117407	8.24	1.23	female
DS10_00004734		scaffold4:1124769-1125465	0.82	39.52	male
DS10_00004752		scaffold4:1249358-1251358	0.06	10.10	male
DS10_00004767		scaffold4:1351682-1352255	0.07	13.01	male
DS10_00004776	pcm	scaffold4:1397463-1397940	0.26	8.02	male
DS10_00004801	Inx5	scaffold4:1714383-1720696	0.45	3.85	male
DS10_00004803	Cam	scaffold4:1732809-1733253	0.34	75.32	male
DS10_00004812		scaffold4:1995926-2007371	0.03	26.83	male
DS10_00004826		scaffold4:2121190-2122000	0.00	19.06	male
DS10_00004833	unc	scaffold4:2239240-2243952	0.03	4.02	male
DS10_00004834		scaffold4:2244690-2250853	0.19	2.51	male
DS10_00004848		scaffold4:2402563-2404370	0.00	15.57	male
DS10_00004850		scaffold4:2411174-2414067	0.00	23.62	male
DS10_00004851	l-3	scaffold4:2463752-2464376	0.00	29.47	male
DS10_00004852		scaffold4:2476184-2477483	0.00	27.19	male
DS10_00004853		scaffold4:2515309-2516590	0.00	22.39	male
DS10_00004855		scaffold4:2611027-2612073	0.00	22.87	male
DS10_00004856		scaffold4:2621906-2622770	0.09	147.90	male
DS10_00004858	hydra	scaffold4:2684094-2685796	0.03	53.05	male
DS10_00004859		scaffold4:2686189-2686936	0.51	108.57	male
DS10_00004880		scaffold4:3079809-3080361	0.09	204.48	male
DS10_00004890	Yp1	scaffold4:3245167-3246706	925.42	0.67	female
DS10_00004891	Yp2	scaffold4:3248354-3250103	669.63	0.98	female
DS10_00004939		scaffold4:4069912-4071067	0.16	25.00	male
DS10_00004946	Erk7	scaffold4:4138145-4147237	0.07	25.58	male
DS10_00004965		scaffold4:4231045-4231714	72.34	16.69	female
DS10_00004970	png	scaffold4:4270815-4271691	2.70	0.17	female
DS10_00004977	fs(1)N	scaffold4:4304662-4311637	7.96	0.32	female
DS10_00004981		scaffold4:4353482-4355199	1.42	16.60	male
DS10_00005024	Mcm6	scaffold4:4997520-4999974	8.69	1.38	female
DS10_00005032		scaffold4:5046541-5048752	0.04	43.99	male
DS10_00005034		scaffold4:5090551-5091689	0.05	36.88	male
DS10_00005045	swa	scaffold4:5313748-5315682	18.22	3.43	female
DS10_00005071	p-cup	scaffold5:96579-99346	0.00	11.44	male
DS10_00005074		scaffold5:109861-125165	0.22	18.36	male
DS10_00005077		scaffold5:240969-254057	1.26	26.96	male
DS10_00005148		scaffold5:968819-970141	0.09	12.34	male
DS10_00005176		scaffold5:1235561-1236615	3.62	46.99	male
DS10_00005188		scaffold5:1355219-1356100	0.46	4.72	male
DS10_00005207	Klp3A	scaffold5:1452417-1457002	3.80	19.45	male
DS10_00005224		scaffold5:1749156-1752925	0.05	41.00	male
DS10_00005226		scaffold5:1764588-1766318	0.71	17.27	male
DS10_00005283		scaffold5:2319840-2321844	7.17	34.95	male
DS10_00005286		scaffold5:2343520-2344580	0.27	13.59	male
DS10_00005299		scaffold5:2518831-2520166	0.00	52.65	male
DS10_00005314		scaffold5:2693378-2695661	0.00	28.62	male
DS10_00005325		scaffold5:2837038-2838380	0.00	32.54	male
DS10_00005326		scaffold5:2838737-2839334	0.07	146.55	male
DS10_00005338		scaffold5:3050210-3052844	0.13	3.29	male

DS10_00005352	Dhc16F	scaffold5:3359433-3375718	0.03	4.01	male
DS10_00005364		scaffold5:3451915-3452695	0.00	34.60	male
DS10_00005381		scaffold5:3634736-3636215	0.18	20.08	male
DS10_00005391		scaffold5:3799239-3799869	0.07	12.79	male
DS10_00005392	SIP3	scaffold5:3834406-3835420	0.04	11.65	male
DS10_00005398		scaffold5:3884726-3886494	7.79	0.02	female
DS10_00005402		scaffold5:3926392-3928336	0.00	24.82	male
DS10_00005411		scaffold5:4066295-4080925	0.00	110.69	male
DS10_00005424		scaffold5:4202081-4203933	0.05	18.08	male
DS10_00005429		scaffold5:4281440-4282945	0.04	44.34	male
DS10_00005444		scaffold5:4606540-4608955	0.02	41.52	male
DS10_00005446		scaffold5:4618309-4620439	23.49	2.61	female
DS10_00005449		scaffold5:4642468-4645754	0.00	16.65	male
DS10_00005450		scaffold5:4663419-4667436	0.00	18.60	male
DS10_00005452		scaffold5:4684560-4686120	0.00	22.45	male
DS10_00005456		scaffold5:4714950-4715274	129.67	0.00	female
DS10_00005460		scaffold5:4741363-4745847	0.00	41.77	male
DS10_00005461		scaffold5:4747302-4748125	0.00	37.16	male
DS10_00005462		scaffold5:4748533-4749158	0.00	49.73	male
DS10_00005467		scaffold6:167763-169439	0.32	5.13	male
DS10_00005469		scaffold6:281634-282420	0.00	47.55	male
DS10_00005479	mus209	scaffold6:346137-347938	24.60	1.50	female
DS10_00005498		scaffold6:505803-507264	0.03	10.30	male
DS10_00005507	dup	scaffold6:595052-599157	6.19	0.86	female
DS10_00005511		scaffold6:640582-654232	0.05	10.11	male
DS10_00005517		scaffold6:765630-767261	1.37	71.57	male
DS10_00005535		scaffold6:859363-863123	1.04	13.82	male
DS10_00005540		scaffold6:978850-994039	0.22	3.64	male
DS10_00005563	Ctf4	scaffold6:1309515-1312197	4.51	0.72	female
DS10_00005565		scaffold6:1322710-1323727	1.29	98.94	male
DS10_00005587		scaffold6:1532093-1546571	0.04	3.71	male
DS10_00005594		scaffold6:1579258-1582390	7.07	0.42	female
DS10_00005596	phtf	scaffold6:1604180-1608628	9.07	50.54	male
DS10_00005638		scaffold6:2120638-2130217	0.01	14.87	male
DS10_00005656		scaffold6:2347775-2359657	0.22	43.84	male
DS10_00005659		scaffold6:2382765-2383276	0.11	33.01	male
DS10_00005665		scaffold6:2409273-2413075	3.55	39.52	male
DS10_00005674	dpa	scaffold6:2467521-2470493	10.14	2.03	female
DS10_00005675	didum	scaffold6:2470883-2478784	13.37	46.46	male
DS10_00005684		scaffold6:2509668-2516164	1.24	46.58	male
DS10_00005709		scaffold6:2686797-2694477	1.10	40.37	male
DS10_00005721		scaffold6:2871897-2873517	0.05	10.79	male
DS10_00005741		scaffold6:3044041-3044392	0.17	33.93	male
DS10_00005753	nopo	scaffold6:3192916-3194570	11.55	1.89	female
DS10_00005764		scaffold6:3216073-3279212	0.45	81.19	male
DS10_00005771		scaffold6:3410580-3411207	0.08	30.46	male
DS10_00005785	Jabba	scaffold6:3579264-3583975	50.41	7.61	female
DS10_00005809	Vha100-3	scaffold6:3758153-3760913	0.00	7.89	male
DS10_00005812	Topors	scaffold6:3832176-3839731	4.00	18.99	male
DS10_00005817		scaffold6:3931755-3932996	0.00	96.65	male
DS10_00005818		scaffold6:3937794-3939434	0.05	74.67	male
DS10_00005831	wbl	scaffold6:4166813-4168367	3.84	32.62	male
DS10_00005858		scaffold6:4350770-4354027	0.60	17.52	male
DS10_00005863		scaffold7:32163-34888	0.05	9.81	male

DS10_00005896		scaffold7:246227-247538	0.09	7.00	male
DS10_00005904		scaffold7:274639-286405	2.10	10.85	male
DS10_00005908		scaffold7:295878-305867	0.18	6.44	male
DS10_00005930		scaffold7:488707-497765	0.06	8.87	male
DS10_00005931		scaffold7:488707-497765	0.09	7.60	male
DS10_00005937		scaffold7:530966-534276	0.00	23.63	male
DS10_00005950		scaffold7:795990-796998	0.00	21.39	male
DS10_00005951		scaffold7:797548-798855	0.28	11.37	male
DS10_00005969		scaffold7:896058-898446	0.04	9.40	male
DS10_00005973		scaffold7:937028-943591	219.58	12.21	female
DS10_00005978		scaffold7:1105615-1109068	0.00	35.97	male
DS10_00005979		scaffold7:1152159-1152606	0.18	3.21	male
DS10_00005980		scaffold7:1153163-1155396	0.17	2.06	male
DS10_00005981		scaffold7:1156411-1161004	0.03	4.49	male
DS10_00005982		scaffold7:1161523-1162894	0.04	17.25	male
DS10_00005983		scaffold7:1164805-1166625	0.12	2.39	male
DS10_00006002		scaffold7:1464949-1470274	0.18	14.19	male
DS10_00006003		scaffold7:1470670-1471714	0.10	15.30	male
DS10_00006004		scaffold7:1473261-1476671	0.09	23.91	male
DS10_00006006		scaffold7:1485537-1486492	0.22	12.40	male
DS10_00006010		scaffold7:1524710-1527737	0.11	83.95	male
DS10_00006018		scaffold7:1545063-1546484	0.09	30.79	male
DS10_00006022		scaffold7:1582892-1583465	0.00	68.79	male
DS10_00006031		scaffold7:1738332-1747665	1.99	99.02	male
DS10_00006053	fs(1)Ya	scaffold7:1862755-1866700	10.78	0.25	female
DS10_00006059		scaffold7:1899591-1900182	0.36	5.13	male
DS10_00006063		scaffold7:1968385-1969775	0.18	2.66	male
DS10_00006089		scaffold7:2733091-2734225	0.29	15.86	male
DS10_00006090		scaffold7:2776832-2777408	0.17	15.01	male
DS10_00006091		scaffold7:2811397-2812000	0.00	100.65	male
DS10_00006093		scaffold7:2816629-2817862	0.00	15.23	male
DS10_00006099	Gas8	scaffold7:2852486-2857649	0.12	9.53	male
DS10_00006101		scaffold7:2867249-2888812	0.00	24.02	male
DS10_00006114	yin	scaffold7:3294104-3299262	30.26	5.89	female
DS10_00006122		scaffold7:3417935-3420215	4.15	0.51	female
DS10_00006138		scaffold7:3843152-3856415	0.00	8.74	male
DS10_00006139		scaffold7:3856848-3858343	0.00	13.91	male
DS10_00006149		scaffold7:3983703-3985774	0.02	3.24	male
DS10_00006158		scaffold7:4074658-4075270	0.00	42.21	male
DS10_00006185		scaffold11:56497-63456	0.16	20.73	male
DS10_00006188		scaffold11:75238-82731	1.64	16.68	male
DS10_00006202		scaffold11:158355-159378	0.10	7.79	male
DS10_00006229	Pp1-13C	scaffold11:453105-454014	0.05	16.15	male
DS10_00006230	acj6	scaffold11:459867-463048	0.44	5.78	male
DS10_00006240		scaffold11:560407-562288	0.00	24.48	male
DS10_00006250		scaffold11:774143-795196	0.31	13.63	male
DS10_00006267		scaffold11:972787-982214	0.09	48.13	male
DS10_00006283	Cyp28c1	scaffold11:1122430-1125230	4.12	20.75	male
DS10_00006284		scaffold11:1129788-1130741	0.00	18.08	male
DS10_00006300		scaffold11:1270333-1271632	0.22	16.43	male
DS10_00006318		scaffold11:1412414-1420327	0.08	37.38	male
DS10_00006329	mkg-p	scaffold11:1518835-1520708	0.04	3.63	male
DS10_00006338		scaffold11:1607493-1609515	0.02	20.53	male
DS10_00006358		scaffold11:1757036-1757398	0.19	16.98	male

DS10_00006368		scaffold11:1848668-1851063	4.34	42.24	male
DS10_00006377		scaffold11:1977831-1979594	0.10	19.85	male
DS10_00006382		scaffold11:2081365-2082096	0.07	34.45	male
DS10_00006387		scaffold11:2095456-2103248	0.00	37.61	male
DS10_00006391	mei-218	scaffold11:2160941-2168892	1.60	16.68	male
DS10_00006394	xmas-2	scaffold11:2182182-2190490	1.77	13.17	male
DS10_00006409		scaffold11:2404538-2405392	0.07	42.47	male
DS10_00006424		scaffold11:2508184-2512393	0.22	5.22	male
DS10_00006425		scaffold11:2567529-2569632	0.08	7.94	male
DS10_00006436		scaffold11:2844106-2844441	0.00	202.70	male
DS10_00006444		scaffold11:2911869-2913510	0.05	6.07	male
DS10_00006461		scaffold11:3226405-3227743	0.00	34.22	male
DS10_00006462		scaffold11:3237768-3238962	0.03	17.55	male
DS10_00006465		scaffold11:3272668-3273703	0.00	30.03	male
DS10_00006467		scaffold11:3467501-3468726	0.00	21.06	male
DS10_00006468		scaffold11:3608323-3614946	0.01	15.00	male
DS10_00006471		scaffold11:3640734-3641100	0.00	435.05	male
DS10_00006478	Uch-L5R	scaffold11:3698403-3699408	0.00	25.25	male
DS10_00006503		scaffold11:3987213-3987858	0.00	46.08	male
DS10_00006505		scaffold11:4044313-4045234	0.00	25.59	male
DS10_00006511		scaffold8:86066-87863	0.35	19.90	male
DS10_00006516		scaffold8:184290-184662	0.17	8.18	male
DS10_00006517		scaffold8:188653-189954	0.66	7.20	male
DS10_00006521	tomboy40	scaffold8:241957-242983	0.04	57.99	male
DS10_00006535		scaffold8:502194-520298	0.03	18.46	male
DS10_00006545	S-Lap7	scaffold8:592606-594190	0.00	410.26	male
DS10_00006550		scaffold8:688696-702290	0.07	30.72	male
DS10_00006551		scaffold8:704682-706317	0.15	39.17	male
DS10_00006554	S-Lap5	scaffold8:779985-781644	0.02	48.04	male
DS10_00006559		scaffold8:824897-825980	0.00	41.41	male
DS10_00006567		scaffold8:872761-894121	0.19	2.73	male
DS10_00006568		scaffold8:872761-894121	0.34	15.43	male
DS10_00006585		scaffold8:1013289-1017948	0.01	7.92	male
DS10_00006587		scaffold8:1019441-1023931	0.02	9.27	male
DS10_00006589		scaffold8:1062168-1063322	0.00	45.06	male
DS10_00006595		scaffold8:1174784-1181650	0.03	42.47	male
DS10_00006608		scaffold8:1224914-1231408	0.27	96.30	male
DS10_00006622		scaffold8:1320716-1321331	0.00	37.62	male
DS10_00006624		scaffold8:1336410-1343378	0.02	61.27	male
DS10_00006632		scaffold8:1391344-1410707	6.55	34.56	male
DS10_00006637		scaffold8:1487451-1488915	927.70	171.07	female
DS10_00006643	mos	scaffold8:1499518-1500782	3.46	0.46	female
DS10_00006681		scaffold8:1786221-1804472	0.97	16.79	male
DS10_00006695	Odc2	scaffold8:1888192-1916422	0.32	4.79	male
DS10_00006735		scaffold8:2210195-2210543	0.00	68.56	male
DS10_00006737		scaffold8:2214005-2214746	0.05	144.98	male
DS10_00006739		scaffold8:2324771-2325608	0.00	59.74	male
DS10_00006740	spaw	scaffold8:2325660-2326968	0.00	127.34	male
DS10_00006741	cola	scaffold8:2327278-2334014	0.01	25.75	male
DS10_00006742		scaffold8:2334015-2334495	0.00	212.76	male
DS10_00006743		scaffold8:2335957-2336790	0.00	210.05	male
DS10_00006744		scaffold8:2338617-2339358	0.00	329.26	male
DS10_00006772		scaffold8:2630379-2631113	0.00	40.80	male
DS10_00006784		scaffold8:2715490-2734019	0.75	64.24	male

DS10_00006789		scaffold8:2752004-2755975	0.05	28.06	male
DS10_00006792	PGRP-SC1b	scaffold8:2758829-2765154	4.85	0.32	female
DS10_00006793	PGRP-SC1b	scaffold8:2765972-2766530	8.40	1.22	female
DS10_00006800		scaffold8:2847772-2848792	0.00	42.31	male
DS10_00006801	sns	scaffold8:2869724-2917327	1.38	10.77	male
DS10_00006815		scaffold10:93548-94198	634.50	180.92	female
DS10_00006829	vanin-like	scaffold10:218581-227122	5.22	1.03	female
DS10_00006852	otu	scaffold10:470344-475526	15.06	1.18	female
DS10_00006883		scaffold10:917672-918833	0.10	187.09	male
DS10_00006899		scaffold10:1143119-1145333	5.42	24.90	male
DS10_00006906		scaffold10:1186123-1187038	0.67	8.57	male
DS10_00006907	Klp10A	scaffold10:1187489-1191967	15.22	59.49	male
DS10_00006920		scaffold10:1275423-1275912	0.00	329.86	male
DS10_00006921		scaffold10:1299238-1299658	0.00	94.73	male
DS10_00006925		scaffold10:1355058-1355451	0.14	58.78	male
DS10_00006937	Rpn13R	scaffold10:1523785-1524742	0.00	16.70	male
DS10_00006939		scaffold10:1582003-1584248	0.04	12.39	male
DS10_00006942		scaffold10:1752751-1759185	0.07	23.51	male
DS10_00006943		scaffold10:1759372-1760089	0.06	20.47	male
DS10_00006946	TrxT	scaffold10:1764826-1766244	0.37	148.68	male
DS10_00006947	dhd	scaffold10:1766471-1766795	563.60	0.77	female
DS10_00006948		scaffold10:1767674-1770394	0.39	70.98	male
DS10_00006949		scaffold10:1771336-1774800	3.60	0.25	female
DS10_00006952	Mcm3	scaffold10:1781046-1785372	5.23	0.70	female
DS10_00006967		scaffold10:2086843-2087542	0.00	34.08	male
DS10_00006974		scaffold10:2156844-2167311	0.09	20.59	male
DS10_00006975		scaffold10:2167425-2168372	22.25	0.12	female
DS10_00006976	CBP	scaffold10:2175729-2181693	13.10	0.73	female
DS10_00006978		scaffold10:2202081-2202669	0.00	105.05	male
DS10_00006981		scaffold10:2252184-2253324	0.00	25.47	male
DS10_00006984		scaffold10:2265180-2265710	472.55	0.00	female
DS10_00006992		scaffold10:2314453-2315167	0.20	4.65	male
DS10_00006993		scaffold10:2356761-2361432	0.05	50.15	male
DS10_00006994		scaffold10:2363333-2364212	0.00	47.36	male
DS10_00006995		scaffold10:2377555-2382719	0.02	4.94	male
DS10_00006997		scaffold10:2411128-2412124	0.00	24.56	male
DS10_00006998		scaffold10:2457284-2459345	0.00	19.29	male
DS10_00006999		scaffold10:2472252-2472858	0.00	86.50	male
DS10_00007014		scaffold10:2790155-2791340	0.00	13.07	male
DS10_00007017	1-Dec	scaffold10:2808812-2815061	8.95	0.03	female
DS10_00007038		scaffold10:2902159-2905893	0.00	14.02	male
DS10_00007083	fd102C	scaffold9:798124-800113	0.19	10.92	male
DS10_00007087	ey	scaffold9:949904-976416	1.28	12.00	male
DS10_00007146	JYalpha	scaffold9:2329358-2339823	0.03	6.99	male
DS10_00007159	zye	scaffold12:203463-211816	4.28	0.54	female
DS10_00007176	Spn77Bc	scaffold12:287584-291150	0.00	38.30	male
DS10_00007179		scaffold12:294676-295998	0.57	37.23	male
DS10_00007207		scaffold12:501124-502015	0.00	21.29	male
DS10_00007251	SMSr	scaffold12:939057-941884	7.63	34.78	male
DS10_00007253		scaffold12:947233-949264	0.00	103.10	male
DS10_00007258		scaffold12:1033069-1033842	0.00	200.76	male
DS10_00007261		scaffold12:1049219-1052543	1.19	7.33	male
DS10_00007263	Tsp66A	scaffold12:1063018-1067310	0.00	8.77	male
DS10_00007278		scaffold12:1203302-1211004	0.00	4.21	male

DS10_00007279		scaffold12:1213473-1215603	0.00	15.35	male
DS10_00007298	wisp	scaffold13:300371-305834	17.84	0.04	female
DS10_00007346		scaffold13:808686-811463	0.14	15.28	male
DS10_00007350		scaffold13:834369-835128	0.13	18.95	male
DS10_00007351	Muc14A	scaffold13:835186-840669	0.06	2.79	male
DS10_00007352		scaffold13:842257-844629	0.08	5.80	male
DS10_00007369		scaffold21:126089-135509	0.34	3.01	male
DS10_00007372	gskt	scaffold21:264365-265874	0.00	42.55	male
DS10_00007380		scaffold21:366987-373020	0.06	16.06	male
DS10_00007397		scaffold21:653267-656496	0.02	33.96	male
DS10_00007417		scaffold21:766202-767462	0.72	72.09	male
DS10_00007424		scaffold21:1052080-1053100	0.00	18.99	male
DS10_00007432	Mst98Ca	scaffold39:137777-151585	0.03	94.88	male
DS10_00007454		scaffold39:782650-783434	0.54	56.76	male
DS10_00007468		scaffold14:187579-190606	0.00	45.07	male
DS10_00007476	yl	scaffold14:438787-446137	81.69	0.02	female
DS10_00007480		scaffold14:475635-477550	46.40	2.15	female
DS10_00007494	betaNACtes6	scaffold14:657059-657968	0.00	39.58	male
DS10_00007520	armi	scaffold27:38241-43694	16.91	1.93	female
DS10_00007530		scaffold27:281243-282310	0.00	21.62	male
DS10_00007540		scaffold27:503128-505114	6.27	30.01	male
DS10_00007552		scaffold27:673756-674146	0.00	167.53	male
DS10_00007568	Dpy-30L2	scaffold27:808008-808308	0.00	138.89	male
DS10_00007570		scaffold27:825846-827998	1.17	32.84	male
DS10_00007594		scaffold26:255852-259092	0.00	34.01	male
DS10_00007622		scaffold26:659685-660738	0.08	21.20	male
DS10_00007631		scaffold26:752709-755949	0.02	4.34	male
DS10_00007634		scaffold26:795630-797175	0.00	30.55	male
DS10_00007640		scaffold18:143187-145497	0.00	14.53	male
DS10_00007652		scaffold18:464432-465914	0.00	64.24	male
DS10_00007653		scaffold18:466695-467574	0.28	36.51	male
DS10_00007654		scaffold18:490931-491510	1.25	12.40	male
DS10_00007656		scaffold18:552722-553343	0.00	28.59	male
DS10_00007674		scaffold18:835626-836821	0.18	2.10	male
DS10_00007675		scaffold28:10419-12209	0.00	13.37	male
DS10_00007677	ACXC	scaffold28:172306-175303	0.00	26.36	male
DS10_00007678	ACXD	scaffold28:191504-199215	0.03	7.99	male
DS10_00007679	Vm26Aa	scaffold28:213387-213822	77.54	0.11	female
DS10_00007681	ACXE	scaffold28:262124-296560	0.01	4.26	male
DS10_00007694		scaffold28:542350-547277	0.02	26.95	male
DS10_00007702	DnaJ-H	scaffold28:590455-596067	10.52	53.39	male
DS10_00007713		scaffold28:641488-642969	1.40	15.61	male
DS10_00007749		scaffold31:340360-340837	0.00	61.48	male
DS10_00007776		scaffold31:685286-686087	0.08	57.89	male
DS10_00007777		scaffold31:691055-691931	0.00	47.95	male
DS10_00007783		scaffold15:142532-145109	0.00	7.48	male
DS10_00007784	His2Av	scaffold15:219270-219699	0.12	24.82	male
DS10_00007800	Clk	scaffold15:405294-411508	2.50	57.57	male
DS10_00007804		scaffold15:510198-553746	2.41	20.09	male
DS10_00007821		scaffold15:665820-669478	0.14	42.52	male
DS10_00007863		scaffold16:289821-299121	0.00	11.18	male
DS10_00007915		scaffold16:660199-661654	0.10	15.01	male
DS10_00007918		scaffold16:731965-734517	0.05	20.18	male
DS10_00007919		scaffold16:740429-740858	0.31	5.09	male

DS10_00007922		scaffold17:39678-48875	0.00	18.77	male
DS10_00007937		scaffold17:391248-392399	0.04	78.21	male
DS10_00007945		scaffold17:594674-611204	0.02	8.08	male
DS10_00007946		scaffold17:648977-649992	0.49	10.82	male
DS10_00007949	Trxr-2	scaffold17:654436-655807	0.03	17.82	male
DS10_00007952		scaffold41:23561-25109	0.26	11.79	male
DS10_00007978	Andorra	scaffold20:89341-90088	0.06	5.65	male
DS10_00007986	os	scaffold20:237242-238809	0.21	4.44	male
DS10_00007998		scaffold20:363882-364281	0.36	2.78	male
DS10_00007999		scaffold20:379292-381321	0.02	42.96	male
DS10_00008008		scaffold20:447615-449364	0.02	5.29	male
DS10_00008018		scaffold20:518419-521421	0.00	23.30	male
DS10_00008025	Fcp1	scaffold20:659406-661955	0.53	4.09	male
DS10_00008027		scaffold20:681528-687751	0.00	7.00	male
DS10_00008030		scaffold20:727921-732287	0.13	9.54	male
DS10_00008037		scaffold19:234383-236495	0.00	7.85	male
DS10_00008075		scaffold45:346519-349433	0.16	71.71	male
DS10_00008089		scaffold45:566655-571358	0.10	21.69	male
DS10_00008090		scaffold45:598326-599512	0.00	53.82	male
DS10_00008093		scaffold45:623154-624574	0.00	31.17	male
DS10_00008107		scaffold25:107177-108701	0.05	6.67	male
DS10_00008132	Tektin-C	scaffold25:405659-408530	0.07	22.53	male
DS10_00008166		scaffold22:443864-445907	0.02	25.39	male
DS10_00008187	Prosbeta2R1	scaffold23:135811-137935	0.00	19.11	male
DS10_00008188		scaffold23:144580-145231	3.37	0.26	female
DS10_00008194		scaffold23:222209-223205	0.04	33.26	male
DS10_00008206	fs(1)M3	scaffold23:323485-329519	13.01	0.01	female
DS10_00008249		scaffold24:507863-512537	0.22	35.16	male
DS10_00008256		scaffold98:69591-71523	0.00	11.60	male
DS10_00008262		scaffold98:88556-98843	0.25	10.26	male
DS10_00008264		scaffold98:99116-101048	0.00	12.97	male
DS10_00008276		scaffold98:235744-238007	5.86	0.23	female
DS10_00008280		scaffold98:317579-319098	0.00	26.75	male
DS10_00008319		scaffold29:209029-211147	0.77	45.39	male
DS10_00008325		scaffold29:344469-346317	0.00	26.52	male
DS10_00008326		scaffold29:347519-351614	4.19	0.13	female
DS10_00008338	rha	scaffold29:533155-537963	0.05	21.51	male
DS10_00008361	vfl	scaffold30:191843-204776	4.85	0.76	female
DS10_00008368	PPP4R2r	scaffold30:240196-246311	0.29	2.78	male
DS10_00008378	BthD	scaffold30:291833-292575	10.51	0.84	female
DS10_00008390		scaffold30:388758-412662	5.93	39.69	male
DS10_00008398		scaffold30:457143-457673	12.20	0.20	female
DS10_00008400	Yp3	scaffold30:460197-462643	316.43	1.90	female
DS10_00008404		scaffold30:503614-504793	0.00	14.79	male
DS10_00008431		scaffold33:432457-458737	0.02	6.88	male
DS10_00008432		scaffold33:502416-503568	0.00	63.78	male
DS10_00008434		scaffold34:53240-55513	0.65	4.82	male
DS10_00008437		scaffold34:57354-60680	0.25	38.91	male
DS10_00008441		scaffold34:80041-82738	0.03	2.96	male
DS10_00008444	t-cup	scaffold34:139804-140518	0.00	57.36	male
DS10_00008454		scaffold34:412975-417512	0.11	32.21	male
DS10_00008464	Vm26Ab	scaffold34:456995-457748	43.54	0.12	female
DS10_00008483	fig	scaffold40:151432-152392	0.26	53.15	male
DS10_00008519		scaffold40:363444-381709	2.29	17.94	male

DS10_00008538		scaffold32:268722-271382	0.02	10.25	male
DS10_00008539		scaffold32:271800-276593	2.99	13.74	male
DS10_00008544		scaffold32:348583-350197	0.02	12.26	male
DS10_00008545		scaffold32:361381-362413	0.06	15.93	male
DS10_00008568		scaffold70:118442-119915	0.04	23.82	male
DS10_00008573		scaffold70:151401-152657	0.00	21.35	male
DS10_00008576	dpr17	scaffold70:248465-254109	1.57	51.24	male
DS10_00008579		scaffold70:305770-310381	0.00	39.78	male
DS10_00008580		scaffold70:330088-333568	0.00	28.77	male
DS10_00008581		scaffold70:346722-348473	0.00	18.97	male
DS10_00008589	mfas	scaffold70:400846-410772	33.56	129.40	male
DS10_00008598		scaffold120:91566-96391	0.02	15.00	male
DS10_00008608	laf	scaffold120:236362-252695	0.01	11.30	male
DS10_00008629	KP78a	scaffold123:98821-104413	0.12	6.03	male
DS10_00008631		scaffold123:225965-235590	0.50	243.27	male
DS10_00008636		scaffold123:250654-251488	0.23	230.60	male
DS10_00008650	Ugt35b	scaffold123:305153-314429	24.40	7.06	female
DS10_00008657		scaffold123:367469-370120	0.02	39.79	male
DS10_00008666		scaffold35:216844-219659	0.00	43.13	male
DS10_00008670		scaffold35:343716-354824	0.15	10.92	male
DS10_00008672		scaffold35:362460-363192	0.00	28.34	male
DS10_00008677		scaffold35:395139-402254	4.06	37.63	male
DS10_00008689		scaffold36:37528-38686	0.04	14.02	male
DS10_00008692	Dhc16F	scaffold36:78105-139944	0.07	7.69	male
DS10_00008698		scaffold36:256944-257379	0.11	27.40	male
DS10_00008702		scaffold36:445802-446062	3.02	0.29	female
DS10_00008717		scaffold251:8685-16705	0.08	2.51	male
DS10_00008728		scaffold251:76817-82902	21.65	5.78	female
DS10_00008735		scaffold251:212534-213719	0.94	15.73	male
DS10_00008750		scaffold251:401467-414158	0.17	8.63	male
DS10_00008772		scaffold37:163617-164765	0.45	131.80	male
DS10_00008785		scaffold37:311334-312150	0.00	23.23	male
DS10_00008799		scaffold37:440538-441649	0.00	31.20	male
DS10_00008822		scaffold97:84674-85712	0.00	58.72	male
DS10_00008826		scaffold97:96371-105536	0.12	26.94	male
DS10_00008843	Spn75F	scaffold97:397906-399153	0.04	21.30	male
DS10_00008851		scaffold38:53555-54995	0.15	2.80	male
DS10_00008859		scaffold38:152093-154658	0.00	7.46	male
DS10_00008860		scaffold38:156299-179786	0.02	8.19	male
DS10_00008866	Rpt4R	scaffold38:258850-260124	1.05	11.90	male
DS10_00008867	Rpt4R	scaffold38:260463-262245	2.34	25.25	male
DS10_00008877		scaffold38:319254-320306	0.15	26.05	male
DS10_00008884		scaffold38:375777-376164	0.14	101.87	male
DS10_00008888	His3:CG33842	scaffold48:3946-4345	8.06	0.16	female
DS10_00008890	His3:CG33842	scaffold48:11554-11965	24.78	1.13	female
DS10_00008891	His4:CG33905	scaffold48:12258-12569	22.11	1.62	female
DS10_00008892	His2Av	scaffold48:13056-13431	81.85	12.01	female
DS10_00008894	His1:CG33807	scaffold48:14512-15276	10.44	0.77	female
DS10_00008895	His3:CG33842	scaffold48:16743-17100	10.72	0.22	female
DS10_00008912		scaffold48:405341-407451	0.06	6.68	male
DS10_00008925		scaffold200:231980-234802	0.00	29.42	male
DS10_00008928		scaffold200:247345-248260	0.00	36.83	male
DS10_00008929		scaffold200:250047-254091	1.73	30.34	male
DS10_00008936		scaffold200:349125-356203	0.00	37.08	male

DS10_00008939		scaffold200:369438-387436	0.03	10.59	male
DS10_00008960		scaffold42:148243-149131	0.00	17.22	male
DS10_00008981		scaffold42:419462-420296	0.00	39.46	male
DS10_00009070		scaffold59:135866-138046	0.60	29.23	male
DS10_00009098		scaffold44:27144-28113	0.00	24.47	male
DS10_00009099		scaffold44:28734-30085	0.00	14.89	male
DS10_00009101		scaffold44:44631-53720	0.00	19.78	male
DS10_00009107		scaffold44:214464-215355	0.06	16.92	male
DS10_00009122		scaffold68:43836-44574	0.00	47.65	male
DS10_00009124		scaffold68:75515-76332	0.00	72.81	male
DS10_00009132		scaffold68:216057-218436	0.00	25.25	male
DS10_00009150		scaffold290:243408-245898	0.00	25.45	male
DS10_00009153		scaffold290:278961-283356	0.00	45.59	male
DS10_00009163		scaffold46:85273-86068	0.11	12.18	male
DS10_00009179		scaffold46:250815-256222	0.30	5.58	male
DS10_00009181		scaffold46:250815-256222	1.45	53.40	male
DS10_00009264	twin	scaffold94:61408-71774	17.72	72.33	male
DS10_00009286		scaffold94:238463-242205	2.16	15.00	male
DS10_00009288		scaffold94:243971-244685	0.00	619.42	male
DS10_00009311		scaffold93:223133-224702	0.00	23.35	male
DS10_00009322		scaffold52:21154-31495	0.01	15.17	male
DS10_00009334		scaffold52:195615-197757	0.00	125.71	male
DS10_00009344		scaffold52:279966-285767	0.03	10.47	male
DS10_00009357		scaffold52:369175-371206	0.13	38.69	male
DS10_00009358	djl	scaffold52:372584-373539	0.60	51.67	male
DS10_00009359		scaffold52:375922-379108	2.24	36.31	male
DS10_00009373		scaffold55:219284-221504	0.02	19.23	male
DS10_00009374	Cyp313a3	scaffold55:241786-251486	0.00	10.80	male
DS10_00009377		scaffold55:258712-259754	0.03	20.31	male
DS10_00009381		scaffold55:291684-294875	0.00	19.47	male
DS10_00009382		scaffold55:297363-300909	0.03	7.04	male
DS10_00009405	topi	scaffold203:230594-238019	0.20	7.70	male
DS10_00009428	Ppi1	scaffold49:38228-50052	0.07	82.82	male
DS10_00009441		scaffold49:159503-160044	0.13	386.53	male
DS10_00009454	Hsc70-2	scaffold64:169-2649	0.60	28.19	male
DS10_00009472	stg	scaffold160:63395-74011	3.01	0.18	female
DS10_00009476		scaffold160:127212-128809	0.16	3.33	male
DS10_00009479		scaffold160:147314-149494	0.21	3.18	male
DS10_00009497	osk	scaffold65:2-24372	78.69	0.19	female
DS10_00009516		scaffold65:328067-329391	0.00	18.85	male
DS10_00009517		scaffold65:331190-332609	0.32	51.62	male
DS10_00009520		scaffold65:339703-341181	0.06	18.66	male
DS10_00009543	spdo	scaffold53:91953-108890	40.95	12.86	female
DS10_00009546		scaffold53:128168-143001	0.00	17.53	male
DS10_00009553	Rpt6R	scaffold53:289803-292925	0.02	13.65	male
DS10_00009563		scaffold53:349138-350334	0.03	77.74	male
DS10_00009572		scaffold156:286496-287458	0.00	46.05	male
DS10_00009573		scaffold156:296299-302947	0.00	29.25	male
DS10_00009576	c(3)G	scaffold156:351831-354645	7.53	1.40	female
DS10_00009579		scaffold56:30589-31197	0.06	3.68	male
DS10_00009605	mura	scaffold56:116677-124125	8.54	30.07	male
DS10_00009628		scaffold56:301649-305711	1.56	18.57	male
DS10_00009629	soti	scaffold56:325038-325485	0.00	39.73	male
DS10_00009679		scaffold106:290801-299745	0.01	19.82	male

DS10_00009687		scaffold106:324568-336811	0.17	240.89	male
DS10_00009694		scaffold89:22065-22974	0.00	80.89	male
DS10_00009712		scaffold89:267348-269625	0.03	23.24	male
DS10_00009726		scaffold61:17395-18076	0.31	5.44	male
DS10_00009761		scaffold72:247594-249297	0.04	12.36	male
DS10_00009762		scaffold72:280742-281690	0.00	34.41	male
DS10_00009772		scaffold66:107969-119420	0.00	8.58	male
DS10_00009776	cdm	scaffold66:145727-151183	11.71	184.51	male
DS10_00009781		scaffold66:176919-177210	0.77	10.89	male
DS10_00009806		scaffold66:286752-294581	9.59	44.52	male
DS10_00009824		scaffold162:290063-292217	0.05	16.61	male
DS10_00009825		scaffold162:292659-294788	0.02	31.58	male
DS10_00009864		scaffold177:176434-177535	0.00	53.89	male
DS10_00009865		scaffold177:177815-178727	0.00	57.04	male
DS10_00009870		scaffold177:296955-297488	0.00	47.13	male
DS10_00009876	psd	scaffold71:248836-249877	16.79	1.20	female
DS10_00009910		scaffold77:176401-184878	0.09	3.06	male
DS10_00009924		scaffold78:239572-249250	19.61	71.02	male
DS10_00009943		scaffold223:156300-157091	0.07	6.48	male
DS10_00009958		scaffold81:75968-76864	0.04	5.94	male
DS10_00009959		scaffold81:96847-97743	0.13	20.31	male
DS10_00009962	Actn3	scaffold81:280255-282078	0.00	11.74	male
DS10_00009971		scaffold102:47632-49118	0.19	5.49	male
DS10_00009973		scaffold102:49271-53515	0.16	4.17	male
DS10_00010001		scaffold102:164888-182559	0.76	18.11	male
DS10_00010014		scaffold149:47008-54915	0.08	17.63	male
DS10_00010085		scaffold112:57773-61045	44.46	154.49	male
DS10_00010128		scaffold86:50749-55094	3.35	33.81	male
DS10_00010130		scaffold86:65973-67624	0.00	35.93	male
DS10_00010131		scaffold86:67708-68544	0.05	12.31	male
DS10_00010141		scaffold86:156476-157376	0.00	165.76	male
DS10_00010148		scaffold99:133048-138813	15.01	2.42	female
DS10_00010153	Art6	scaffold99:165086-169152	0.02	13.68	male
DS10_00010158		scaffold99:274727-275110	63.16	0.55	female
DS10_00010214		scaffold305:57576-58276	0.07	12.77	male
DS10_00010225		scaffold126:34865-35894	0.00	13.94	male
DS10_00010229		scaffold126:181406-201708	0.03	37.78	male
DS10_00010232		scaffold126:266725-266904	0.00	2831.16	male
DS10_00010239		scaffold92:93449-100061	1.70	82.34	male
DS10_00010241		scaffold92:103578-107973	6.08	37.10	male
DS10_00010245		scaffold92:128664-132032	0.23	9.70	male
DS10_00010257		scaffold92:190888-194227	93.14	28.20	female
DS10_00010261		scaffold92:222528-222918	0.00	228.06	male
DS10_00010279		scaffold158:241959-243871	5.74	28.71	male
DS10_00010295		scaffold155:124221-128338	2.64	16.10	male
DS10_00010297		scaffold155:132106-133095	0.22	5.78	male
DS10_00010318		scaffold166:84779-86149	0.00	19.23	male
DS10_00010332	asun	scaffold166:220418-228770	0.74	7.61	male
DS10_00010357	CheB93b	scaffold308:40146-40868	0.18	3.41	male
DS10_00010389		scaffold204:57788-60733	0.08	19.63	male
DS10_00010397		scaffold204:173229-174735	1.48	17.05	male
DS10_00010398		scaffold204:175418-176924	1.52	16.56	male
DS10_00010417		scaffold100:38608-42565	0.04	17.45	male
DS10_00010433		scaffold100:237051-241729	6.83	29.23	male

DS10_00010435		scaffold152:256-685	0.31	5.09	male
DS10_00010436		scaffold152:5051-5987	0.09	24.03	male
DS10_00010437		scaffold152:6465-7371	0.03	18.87	male
DS10_00010438		scaffold152:28172-30796	0.03	20.36	male
DS10_00010444		scaffold132:127811-128275	0.00	64.64	male
DS10_00010463	ss	scaffold103:216116-250189	0.52	6.60	male
DS10_00010468	tilB	scaffold108:60888-63128	0.24	10.47	male
DS10_00010474	Cp110	scaffold108:112542-118034	6.07	26.89	male
DS10_00010495	Tim17b1	scaffold241:219783-253951	0.41	7.92	male
DS10_00010509		scaffold247:29491-31267	0.17	63.36	male
DS10_00010512		scaffold247:210881-211214	0.70	12.38	male
DS10_00010513		scaffold247:215241-216739	35.56	5.88	female
DS10_00010514		scaffold247:217044-225020	1.14	8.57	male
DS10_00010516		scaffold247:226350-227016	0.00	45.49	male
DS10_00010523	hale	scaffold110:18923-24373	0.75	25.86	male
DS10_00010524	schuy	scaffold110:18923-24373	0.02	52.81	male
DS10_00010538		scaffold110:171510-180331	0.01	5.03	male
DS10_00010547		scaffold111:26366-26639	0.25	26.83	male
DS10_00010548		scaffold111:28224-28416	0.58	41.39	male
DS10_00010571		scaffold117:186-4405	4.63	21.34	male
DS10_00010589		scaffold121:60997-61498	0.10	5.10	male
DS10_00010617	Hex-t1	scaffold214:12326-17587	1.20	14.46	male
DS10_00010624	DppIII	scaffold124:27724-31377	14.98	52.21	male
DS10_00010628		scaffold124:54019-54559	94.69	0.37	female
DS10_00010634		scaffold124:75126-82708	0.07	2.30	male
DS10_00010646	Mcm2	scaffold124:129465-132187	10.87	1.02	female
DS10_00010650		scaffold124:192074-193976	0.42	4.98	male
DS10_00010658		scaffold127:22357-23736	0.22	7.65	male
DS10_00010674		scaffold127:147778-158181	0.07	29.11	male
DS10_00010745	h-cup	scaffold135:88017-88821	0.05	35.76	male
DS10_00010777		scaffold220:123559-131724	2.12	0.18	female
DS10_00010786		scaffold209:20621-21560	0.05	21.84	male
DS10_00010789		scaffold209:33274-34263	0.06	84.18	male
DS10_00010795		scaffold209:192668-194503	0.00	38.89	male
DS10_00010817		scaffold353:126456-132019	0.40	3.45	male
DS10_00010846		scaffold145:35017-36351	0.12	17.24	male
DS10_00010862		scaffold146:16118-17168	0.04	5.17	male
DS10_00010863		scaffold146:17802-18849	0.00	33.38	male
DS10_00010864		scaffold146:38509-43724	0.02	17.31	male
DS10_00010877		scaffold146:88171-89707	1.04	29.64	male
DS10_00010894		scaffold147:71152-71934	0.09	40.95	male
DS10_00010896		scaffold147:75787-76582	1.77	33.18	male
DS10_00010903		scaffold147:145289-147402	0.07	8.48	male
DS10_00010912		scaffold150:55871-58612	0.03	83.67	male
DS10_00010937		scaffold243:72385-81902	0.00	206.43	male
DS10_00010938		scaffold243:92955-93591	0.00	30.31	male
DS10_00010940		scaffold243:115635-116617	0.00	51.80	male
DS10_00010943		scaffold243:147091-148935	0.02	20.26	male
DS10_00010951		scaffold153:102314-115449	28.54	84.83	male
DS10_00010953	Tim17a2	scaffold153:128317-129013	2.70	21.07	male
DS10_00010973	Rcd7	scaffold205:68609-85074	0.14	35.75	male
DS10_00010987		scaffold205:177808-185547	0.02	15.53	male
DS10_00011005		scaffold260:188950-191898	0.02	22.62	male
DS10_00011021		scaffold185:194484-195549	0.00	16.14	male

DS10_00011064		scaffold404:99539-100135	4.08	26.27	male
DS10_00011093		scaffold315:136162-143950	0.11	2.53	male
DS10_00011105	Ran	scaffold202:70943-71594	2.07	46.04	male
DS10_00011117	DNApol-alpha180	scaffold394:165099-170549	3.97	0.61	female
DS10_00011120		scaffold460:16754-18422	3.09	30.81	male
DS10_00011121		scaffold460:18785-21528	0.02	3.55	male
DS10_00011122		scaffold460:43588-43921	0.68	12.18	male
DS10_00011124	dnd	scaffold460:49477-55370	28.10	5.20	female
DS10_00011125		scaffold460:55678-64535	1.82	8.94	male
DS10_00011128		scaffold460:70326-71402	0.23	81.04	male
DS10_00011129	Cby	scaffold460:71715-72246	0.54	14.21	male
DS10_00011130		scaffold460:72651-75183	0.48	37.37	male
DS10_00011156		scaffold172:147145-149124	0.47	8.78	male
DS10_00011159		scaffold172:151068-154184	0.21	11.36	male
DS10_00011160		scaffold172:161490-163022	0.05	7.87	male
DS10_00011183		scaffold173:157289-170290	0.04	10.46	male
DS10_00011193		scaffold175:65599-65791	1.23	6839.53	male
DS10_00011194		scaffold175:87632-89424	0.03	7.75	male
DS10_00011195	Acam	scaffold175:92458-92905	0.00	64.07	male
DS10_00011196		scaffold175:93055-95026	0.00	19.33	male
DS10_00011197	BG642167	scaffold175:99551-100078	0.00	126.40	male
DS10_00011201		scaffold175:127652-135042	0.04	7.79	male
DS10_00011223	RpS5b	scaffold179:45421-47100	280.31	12.12	female
DS10_00011249		scaffold531:95645-116013	0.14	5.22	male
DS10_00011264		scaffold183:26340-34092	0.03	8.22	male
DS10_00011265		scaffold183:34441-38278	0.10	10.19	male
DS10_00011306	Dic2	scaffold186:78875-80641	0.00	40.66	male
DS10_00011345		scaffold188:90795-91298	0.16	404.53	male
DS10_00011363		scaffold189:47533-58563	1.28	127.89	male
DS10_00011368	Glt	scaffold189:95280-103587	75.50	24.92	female
DS10_00011400		scaffold192:104-1195	0.00	17.74	male
DS10_00011406		scaffold192:49108-53361	1.22	34.08	male
DS10_00011415		scaffold192:103560-104607	9.42	43.20	male
DS10_00011429	Best4	scaffold193:52020-56279	0.00	6.53	male
DS10_00011464		scaffold198:18467-19340	0.05	16.38	male
DS10_00011465		scaffold198:38093-42448	0.00	54.70	male
DS10_00011466		scaffold198:69580-83730	0.00	19.52	male
DS10_00011467	JIL-1	scaffold198:93970-98644	0.01	22.13	male
DS10_00011468		scaffold198:143861-145603	0.00	10.84	male
DS10_00011470		scaffold401:13361-14964	0.03	8.04	male
DS10_00011471	Adgf-A	scaffold401:18611-27049	3.07	25.15	male
DS10_00011472		scaffold401:42569-47553	0.02	2.23	male
DS10_00011505		scaffold199:80392-83215	0.38	5.47	male
DS10_00011513		scaffold199:153186-153594	0.42	33.86	male
DS10_00011521		scaffold368:14731-16662	0.03	6.42	male
DS10_00011546		scaffold206:22465-25339	0.23	2.26	male
DS10_00011560		scaffold206:112529-117782	0.54	3.59	male
DS10_00011566	Acp76A	scaffold267:133551-134850	0.00	14.32	male
DS10_00011608		scaffold215:118375-120155	0.74	30.58	male
DS10_00011633		scaffold227:4908-11149	0.04	39.81	male
DS10_00011635		scaffold227:59208-60018	0.00	97.86	male
DS10_00011651		scaffold221:54198-57285	0.02	45.64	male
DS10_00011660		scaffold221:140042-140549	0.00	92.44	male

DS10_00011668		scaffold259:14594-17355	0.08	20.03	male
DS10_00011675		scaffold259:36185-42587	0.02	3.82	male
DS10_00011676		scaffold259:36185-42587	0.23	5.01	male
DS10_00011677		scaffold259:45776-48302	4.37	0.27	female
DS10_00011695		scaffold229:58519-71572	0.00	8.57	male
DS10_00011703		scaffold405:43498-47428	0.00	25.08	male
DS10_00011709	Tengl4	scaffold232:12655-14530	0.00	17.60	male
DS10_00011742		scaffold447:8126-13029	0.00	4.82	male
DS10_00011759		scaffold425:66629-69379	0.00	11.08	male
DS10_00011787		scaffold328:136706-138148	0.00	29.10	male
DS10_00011797		scaffold439:82749-83331	0.08	4.54	male
DS10_00011802	gogo	scaffold372:35945-49010	0.23	5.18	male
DS10_00011825		scaffold337:74166-74757	0.00	38.67	male
DS10_00011838		scaffold261:25162-25715	0.00	60.60	male
DS10_00011851	tral	scaffold266:39607-45474	87.18	27.58	female
DS10_00011858		scaffold266:70127-74352	1.01	15.45	male
DS10_00011860		scaffold266:82359-85896	0.07	61.24	male
DS10_00011877		scaffold264:50776-63525	0.39	3.02	male
DS10_00011899		scaffold307:5905-6793	0.30	15.83	male
DS10_00011901	nos	scaffold307:28532-38037	8.32	0.63	female
DS10_00011906		scaffold307:93464-94052	0.08	3.93	male
DS10_00011919		scaffold676:107624-108073	0.00	60.52	male
DS10_00011921		scaffold676:124145-125937	0.04	2.23	male
DS10_00011922		scaffold676:126181-131112	0.01	18.70	male
DS10_00011927		scaffold451:44093-48875	0.17	21.15	male
DS10_00011928		scaffold451:49186-55232	6.90	24.59	male
DS10_00011935	Dhc98D	scaffold412:56833-87205	0.87	4.26	male
DS10_00011950	eIF4G2	scaffold275:47653-63367	8.06	61.13	male
DS10_00011959		scaffold325:47024-47749	0.07	25.52	male
DS10_00011966		scaffold325:95966-96404	0.16	21.06	male
DS10_00011983		scaffold383:25949-37005	0.01	31.57	male
DS10_00011990		scaffold383:88906-89422	0.20	5.94	male
DS10_00012026		scaffold283:62036-63367	0.10	125.41	male
DS10_00012029		scaffold284:124392-128640	0.00	31.10	male
DS10_00012031		scaffold285:10168-12872	0.06	58.43	male
DS10_00012042	Pxt	scaffold285:84767-90994	24.01	2.06	female
DS10_00012043	Atg8b	scaffold285:92349-92712	0.43	71.52	male
DS10_00012074	His2Av	scaffold494:108177-108552	45.79	5.21	female
DS10_00012076		scaffold301:31657-33273	0.02	11.51	male
DS10_00012077		scaffold301:53221-54250	0.04	75.49	male
DS10_00012079		scaffold301:83895-85047	0.00	80.69	male
DS10_00012107	alpha-Est6	scaffold310:88129-109380	0.06	2.11	male
DS10_00012114	CcapR	scaffold477:10193-31613	0.10	9.90	male
DS10_00012122		scaffold375:55647-56669	0.70	6.55	male
DS10_00012137		scaffold510:95128-96632	0.02	8.49	male
DS10_00012141	Prosalpha3T	scaffold321:39581-40172	0.04	3.33	male
DS10_00012157	klhl10	scaffold335:110691-112203	0.03	51.71	male
DS10_00012159	djl	scaffold324:105705-111171	0.71	101.15	male
DS10_00012160		scaffold324:112769-114721	0.11	48.43	male
DS10_00012177		scaffold802:113447-114425	0.58	30.03	male
DS10_00012204	AGO3	scaffold653:17919-19443	18.67	0.56	female
DS10_00012224		scaffold511:3407-5307	1.26	26.59	male
DS10_00012239		scaffold379:21082-22225	34.07	0.07	female
DS10_00012244		scaffold379:60765-67481	0.06	21.12	male

DS10_00012264		scaffold668:61040-62380	5.70	88.60	male
DS10_00012290		scaffold787:308-2811	0.00	39.39	male
DS10_00012295		scaffold390:31689-32205	2.51	29.40	male
DS10_00012305		scaffold389:54631-60288	0.02	4.31	male
DS10_00012307		scaffold389:61230-62265	0.01	8.36	male
DS10_00012314		scaffold391:80144-85472	0.01	8.38	male
DS10_00012323		scaffold393:81848-83501	0.00	31.30	male
DS10_00012324		scaffold393:84311-85129	0.06	94.22	male
DS10_00012337		scaffold400:53348-56401	0.00	13.13	male
DS10_00012338	Dic4	scaffold400:58326-62077	0.00	8.82	male
DS10_00012350	Or42a	scaffold402:17057-18278	0.12	5.69	male
DS10_00012359		scaffold406:48537-50101	2.03	0.19	female
DS10_00012364	Pk1r	scaffold529:5477-11876	0.18	14.71	male
DS10_00012374		scaffold529:70756-82267	0.02	48.17	male
DS10_00012375		scaffold529:84331-85318	0.00	47.17	male
DS10_00012412		scaffold449:26301-27202	0.00	78.90	male
DS10_00012414		scaffold449:39340-43890	0.03	26.80	male
DS10_00012415	glob2	scaffold449:72762-73289	0.00	51.29	male
DS10_00012432		scaffold574:12431-15647	0.02	41.69	male
DS10_00012435	Rbp4	scaffold574:34902-37608	0.25	8.31	male
DS10_00012455		scaffold418:63862-65326	0.00	28.58	male
DS10_00012460		scaffold420:65118-70240	0.11	16.52	male
DS10_00012461		scaffold420:70789-82348	0.01	8.23	male
DS10_00012464	FK506-bp1	scaffold419:36920-37991	42.04	8.64	female
DS10_00012470		scaffold422:63544-64865	0.00	45.27	male
DS10_00012471		scaffold422:71398-76936	0.01	20.01	male
DS10_00012485	Task7	scaffold433:40967-44683	3.43	113.01	male
DS10_00012521		scaffold444:48264-48573	0.00	91.47	male
DS10_00012535	WRNexo	scaffold562:59879-61400	5.35	0.51	female
DS10_00012536		scaffold562:61836-63938	0.02	30.16	male
DS10_00012539	qin	scaffold448:61052-69402	6.14	0.45	female
DS10_00012561	topi	scaffold462:7099-8336	0.08	4.56	male
DS10_00012568		scaffold462:33865-36001	0.00	65.41	male
DS10_00012584		scaffold771:61565-62397	0.00	81.77	male
DS10_00012620	rt	scaffold483:11842-33996	0.40	4.74	male
DS10_00012623		scaffold483:50411-63253	0.01	9.25	male
DS10_00012629		scaffold488:34842-42012	0.08	40.64	male
DS10_00012638		scaffold575:39596-40484	0.00	16.30	male
DS10_00012639	nos	scaffold575:54062-54554	3.53	0.11	female
DS10_00012641	nos	scaffold575:55086-66076	5.92	0.10	female
DS10_00012657	Vha14-2	scaffold493:23075-24653	0.03	4.31	male
DS10_00012667		scaffold513:55072-56083	0.16	14.79	male
DS10_00012674	Prosalpha3T	scaffold516:36444-37212	0.03	3.69	male
DS10_00012693	nmdyn-D7	scaffold528:419-1918	1.64	20.14	male
DS10_00012712		scaffold547:44895-45630	0.06	59.07	male
DS10_00012718		scaffold549:37407-56508	0.22	25.63	male
DS10_00012733	Hex-t1	scaffold890:403-3301	0.00	20.61	male
DS10_00012748		scaffold577:54582-56848	0.02	15.56	male
DS10_00012801		scaffold622:32106-33323	0.00	64.56	male
DS10_00012804		scaffold626:53227-55180	4.14	0.12	female
DS10_00012807	Hsc70-2	scaffold979:18630-21046	0.48	29.44	male
DS10_00012813		scaffold642:45051-45360	0.00	90.44	male
DS10_00012843		scaffold1441:14531-17764	0.03	18.33	male
DS10_00012856		scaffold1197:64-846	0.09	46.72	male

DS10_00012864		scaffold674:19065-20462	0.90	8.69	male
DS10_00012868		scaffold674:37017-39783	0.02	3.04	male
DS10_00012875		scaffold677:35247-36753	1.19	19.89	male
DS10_00012876		scaffold677:37036-37417	1.93	30.13	male
DS10_00012886		scaffold687:43528-50012	0.18	18.10	male
DS10_00012896	janA	scaffold705:4050-6369	17.72	163.39	male
DS10_00012913		scaffold722:46066-49669	0.02	19.08	male
DS10_00012918		scaffold955:46501-47476	0.61	108.92	male
DS10_00012926		scaffold724:21745-23307	0.07	14.52	male
DS10_00012933	gdl	scaffold724:39197-40310	4.63	29.71	male
DS10_00012942	Cyp312a1	scaffold729:30252-32755	0.62	26.05	male
DS10_00012949		scaffold849:9062-11927	0.00	120.78	male
DS10_00012972		scaffold763:20057-33894	0.16	3.20	male
DS10_00012973		scaffold763:20057-33894	0.51	4.33	male
DS10_00012974		scaffold763:34534-35713	0.47	6.07	male
DS10_00012980		scaffold769:12520-13465	0.04	35.48	male
DS10_00012988	Mcm5	scaffold1377:42551-45173	9.22	1.12	female
DS10_00013022	sina	scaffold815:25338-32410	4.76	35.15	male
DS10_00013032		scaffold830:25209-39938	0.00	4.57	male
DS10_00013048	lobo	scaffold2221:37881-40458	0.04	4.41	male
DS10_00013098		scaffold949:23337-24267	1.88	59.94	male
DS10_00013099		scaffold949:24394-25969	0.08	55.54	male
DS10_00013104		scaffold957:9361-10390	0.00	25.63	male
DS10_00013105		scaffold957:12042-12900	0.00	31.19	male
DS10_00013137		scaffold1017:31678-32580	0.02	61.91	male
DS10_00013140		scaffold1760:8827-12907	2.48	0.03	female
DS10_00013168		scaffold1078:16475-16862	0.22	60.06	male
DS10_00013176		scaffold1104:6079-6587	0.67	280.96	male
DS10_00013223	PH4alphaNE3	scaffold3067:0-5244	0.10	40.55	male
DS10_00013231	Sfp84E	scaffold1258:12797-15866	0.00	30.93	male
DS10_00013242		scaffold1447:18173-19325	0.00	28.73	male
DS10_00013276		scaffold1556:10-1090	0.06	9.41	male
DS10_00013305		scaffold1675:69-4434	0.06	8.16	male
DS10_00013308		scaffold1674:206-1427	0.00	85.44	male
DS10_00013310	Klp59C	scaffold1674:5083-6519	0.00	31.85	male
DS10_00013311	prt	scaffold1680:1505-3930	0.21	213.76	male
DS10_00013321		scaffold1794:7066-8167	0.04	65.05	male
DS10_00013328		scaffold1812:6077-7264	0.04	35.13	male
DS10_00013353		scaffold2145:5859-6462	0.00	77.46	male
DS10_00013357	Taf12L	scaffold2209:9609-10086	0.39	26.52	male
DS10_00013411	mud	scaffold3377:48-4422	0.24	4.84	male
DS10_00013413		scaffold3315:3817-4960	0.07	11.98	male
DS10_00013423	ssp5	scaffold3454:2-1563	0.00	49.28	male
DS10_00013426		scaffold3474:1985-3173	0.86	10.30	male
DS10_00013434		scaffold3680:660-2317	1.94	181.01	male
DS10_00013452		scaffold3819:27-955	0.25	12.31	male
DS10_00013473		scaffold4415:24-2388	0.34	30.35	male
DS10_00013476		scaffold4528:1763-2301	0.18	43.49	male
DS10_00013487	tomboy20	scaffold5051:1397-1847	0.00	133.65	male
DS10_00013498	dhd	scaffold5800:280-604	174.07	0.69	female
DS10_00013500		scaffold6090:341-2044	33.32	116.29	male
DS10_00013525		scaffold6382:0-1596	0.06	5.63	male
DS10_00013532		scaffold6530:1014-1173	1.13	58.68	male

DS10_00013540	CycA	scaffold6719:606-1038	90.22	17.25	female
