# Supplementary material for: Genome of Drosophila suzukii, the Spotted Wing Drosophila
Source: G3 (Bethesda). 2013 Oct 18;3(12):2257–71. doi: 10.1534/g3.113.008185 (PMC3852387; doi:10.1534/g3.113.008185)
Supplement: Supporting Information [file supp_g3.113.008185_TableS6.pdf]

Table S6 A list of genes showing sex-biased shift in expression between *Drosophila suzukii* and *Drosophila melanogaster*.

Gene Name D.suz	Location	Female FPKM	Male FPKM	Bias in D.suz	Best Hit in D.mel	Gene Name D.mel	Gene Symbol	Female FPKM	Male FPKM	Bias in D.mel	Shift Pattern (D.suz compare with D.mel)
DS10_00001473	scaffold1:16714274-16723749	0.22	2.61	Male	FBgn0003046	CG3440	Pcp	11.41	0.09	Female	Female-to-Male
DS10_00001542	scaffold1:17685688-17693475	0.01	9.52	Male	FBgn0032082	CG18088		9.84	0.55	Female	Female-to-Male
DS10_00005904	scaffold7:274639-286405	2.10	10.85	Male	FBgn0053172	CG33172		50.21	5.52	Female	Female-to-Male
DS10_00012620	scaffold483:11842-33996	0.40	4.74	Male	FBgn0003292	CG6097	rt	12.82	1.69	Female	Female-to-Male
DS10_00007784	scaffold15:219270-219699	0.12	24.82	Male	FBgn0001197	CG5499	His2Av	428.69	64.77	Female	Female-to-Male
DS10_00007804	scaffold15:510198-553746	2.41	20.09	Male	FBgn0052365	CG32365		24.78	4.22	Female	Female-to-Male
DS10_00011906	scaffold307:93464-94052	0.08	3.93	Male	FBgn0032354	CG4788		20.46	4.34	Female	Female-to-Male
DS10_00009286	scaffold94:238463-242205	2.16	15.00	Male	FBgn0039189	CG18528		12.29	2.72	Female	Female-to-Male
DS10_00008539	scaffold32:271800-276593	2.99	13.74	Male	FBgn0039417	CG6073		25.28	6.14	Female	Female-to-Male
DS10_00004076	scaffold3:5482391-5483522	0.05	11.53	Male	FBgn0031422	CG9870		4.88	1.23	Female	Female-to-Male
DS10_00008025	scaffold20:659406-661955	0.53	4.09	Male	FBgn0035026	CG12252	Fcp1	26.34	7.71	Female	Female-to-Male
DS10_00001375	scaffold1:15913446-15914124	1.22	9.07	Male	FBgn0003607	CG8409	Su(var)205	134.55	41.00	Female	Female-to-Male
DS10_00011093	scaffold315:136162-143950	0.11	2.53	Male	FBgn0052095	CG32095		22.09	7.07	Female	Female-to-Male
DS10_00010332	scaffold166:220418-228770	0.74	7.61	Male	FBgn0020407	CG6814	asun	11.89	3.91	Female	Female-to-Male
DS10_00006394	scaffold11:2182182-2190490	1.77	13.17	Male	FBgn0028974	CG32562	xmas-2	66.40	24.95	Female	Female-to-Male
DS10_00005283	scaffold5:2319840-2321844	7.17	34.95	Male	FBgn0029929	CG4593		221.97	83.58	Female	Female-to-Male
DS10_00009776	scaffold66:145727-151183	11.71	184.51	Male	FBgn0261532	CG7212	cdm	45.60	20.05	Female	Female-to-Male
DS10_00000894	scaffold1:9772060-9772516	0.49	35.85	Male	FBgn0010602	CG3018	lwr	139.93	63.28	Female	Female-to-Male
DS10_00006949	scaffold10:1771336-1774800	3.60	0.25	Female	FBgn0029754	CG15930		2.56	39.23	Male	Male-to-Female
DS10_00008650	scaffold123:305153-314429	24.40	7.06	Female	FBgn0026314	CG6649	Ugt35b	17.43	66.12	Male	Male-to-Female
DS10_00008326	scaffold29:347519-351614	4.19	0.13	Female	FBgn0039342	CG5107		151.04	438.57	Male	Male-to-Female
DS10_00002942	scaffold2:8639392-8648490	11.98	1.76	Female	FBgn0034887	CG5428	St1	8.82	18.02	Male	Male-to-Female
