# Supplementary material for: Genome of Drosophila suzukii, the Spotted Wing Drosophila
Source: G3 (Bethesda). 2013 Oct 18;3(12):2257–71. doi: 10.1534/g3.113.008185 (PMC3852387; doi:10.1534/g3.113.008185)
Supplement: Supporting Information [file supp_g3.113.008185_TableS7.pdf]

Table S7 GO term and functional classification enrichment analysis using DAVID for gene families that are expanded in the *Drosophila suzukii* genome as compared to 14 *Drosophila* species analyzed in this study.

CATEGORY[a]	TERM	COUNT[b]	P VALUE	GENE FAMILIES: REPRESENTATIVE GENE SYMBOLS[c]
**Annotation Cluster 1**	**Enrichment Score: 1.8103805538093147**			
GOTERM BP	GO:0006071 glycerol metabolic process	3	8.49E-03	Gpdh, CG18135, Gyk
GOTERM BP	GO:0019400 alditol metabolic process	3	8.49E-03	Gpdh, CG18135, Gyk
GOTERM BP	GO:0019751 polyol metabolic process	3	5.14E-02	Gpdh, CG18135, Gyk
**Annotation Cluster 2**	**Enrichment Score: 1.7459845947230437**			
GOTERM BP	GO:0032268 regulation of cellular protein metabolic process	9	3.85E-03	Acp76A, PEK, Adam, mRpL11, Tollo, Spn77Bc, Su(var)205, nos, elav
GOTERM BP	GO:0010605 negative regulation of macromolecule metabolic process	13	4.11E-03	WRNexo, Acp76A, mRpL11, Spn77Bc, Su(var)205, elav, mael, Hsc70-4, PEK, E(spl)m8-HLH, fkh, lolal, nos
GOTERM BP	GO:0032269 negative regulation of cellular protein metabolic process	5	1.36E-02	Acp76A, PEK, Spn77Bc, nos, elav
GOTERM BP	GO:0051248 negative regulation of protein metabolic process	5	1.49E-02	Acp76A, PEK, Spn77Bc, nos, elav
SWISSPROT PIR	translation regulation	3	4.00E-02	PEK, mRpL11, nos

[a] Categories used in DAVID analysis include GO sub-ontologies for Biological Process (BP), Molecular Function (MF), and CC (Cellular Component), as well as keywords from SwissProt PIR (Protein Information Resource) and protein domains from the Interpro database.

[b] Count represents the number of gene families

[c] Gene symbol for representative, most highly annotated member of each gene family is shown. All annotations shown here are from *D. melanogaster*.

GOTERM BP	GO:0006417 regulation of translation	5	4.84E-02	PEK, Adam, mRpL11, nos, elav
GOTERM BP	GO:0010608 posttranscriptional regulation of gene expression	6	9.66E-02	Hsc70-4, PEK, Adam, mRpL11, nos, elav
**Annotation Cluster 3**	**Enrichment Score: 1.5795892033529708**			
SWISSPROT PIR	Chaperone	5	1.54E-02	CG7394, CG11267, Cnx99A, Tcp-1zeta
GOTERM BP	GO:0006457 protein folding	6	2.69E-02	Hsc70-4, CG11267, Cnx99A, Tcp-1zeta, FKBP59
GOTERM MF	GO:0051082 unfolded protein binding	5	4.40E-02	Hsc70-4, CG11267, Cnx99A, Tcp-1zeta
**Annotation Cluster 4**	**Enrichment Score: 1.420233335387723**			
GOTERM BP	GO:0051606 detection of stimulus	6	1.26E-02	Or49a, Calx, CheB42a, Galphaq, FKBP59
SWISSPROT PIR	sensory transduction	6	3.13E-02	Or49a, Or69a, Galphaq, FKBP59, Or22a
GOTERM BP	GO:0009583 detection of light stimulus	4	4.32E-02	Calx, Galphaq, FKBP59
GOTERM BP	GO:0009582 detection of abiotic stimulus	4	6.20E-02	Calx, Galphaq, FKBP59
GOTERM BP	GO:0009581 detection of external stimulus	4	7.48E-02	Calx, Galphaq, FKBP59
**Annotation Cluster 5**	**Enrichment Score: 1.3722382305056715**			
INTERPRO	IPR007087:Zinc finger, C2H2-type	13	2.49E-02	CG6689, term, CG1647, CG16779, CG10669, Meics, CG5316, CG4360, CG3065, CG11966, CG4318
INTERPRO	IPR015880:Zinc finger, C2H2-like	13	2.93E-02	CG6689, CG1647, CG16779, CG10669, Meics, CG5316, CG4360, CG3065, CG11966, noi, CG4318

INTERPRO	IPR012934:Zinc finger, AD-type	5	4.66E-02	CG6689, CG1647, CG10669, Meics, CG4318
INTERPRO	IPR013087:Zinc finger, C2H2-type/integrase, DNA-binding	7	4.70E-02	CG11966, CG6689, CG16779, CG10669, Meics, CG4360, CG3065
SMART	SM00355:ZnF_C2H2	13	8.63E-02	CG6689, CG1647, CG16779, CG10669, Meics, CG5316, CG4360, CG3065, CG11966, noi, CG4318
**Annotation Cluster 6**	**Enrichment Score: 1.2224866596583404**			
SWISSPROT PIR	sensory transduction	6	3.13E-02	Or49a, Or69a, Galphaq, FKBP59, Or22a
GOTERM MF	GO:0005549 odorant binding	6	3.17E-02	Obp83ef, Or49a, Obp51a, CheB42a, Or69a, Or22a
GOTERM BP	GO:0007186 G-protein coupled receptor protein signaling pathway	10	4.14E-02	Or49a, Tk, D2R, Or69a, Galphaq, Gr85a, mth, Pk1r, Or22a
SWISSPROT PIR	transducer	8	4.29E-02	Or49a, D2R, Or69a, Galphaq, Gr85a, mth, Pk1r, Or22a
GOTERM BP	GO:0007600 sensory perception	9	5.60E-02	Obp83ef, Or49a, Obp51a, Or69a, Galphaq, Gr85a, FKBP59, Or22a
SWISSPROT PIR	g-protein coupled receptor	7	8.04E-02	Or49a, D2R, Or69a, Gr85a, mth, Pk1r, Or22a
INTERPRO	IPR004117:Olfactory receptor, Drosophila	3	8.44E-02	Or49a, Or69a, Or22a
GOTERM CC	GO:0005886 plasma membrane	17	9.45E-02	D2R, Tollo, Or69a, Gr85a, Rac1, Cnx99A, Cad74A, Oscp, Pk1r, Mical, Syn1, Or49a, Calx, Galphaq, mth, FKBP59, Or22a
PIR_SUPERFAMILY	PIRSF008678:odorant receptor	3	9.64E-02	Or49a, Or69a, Or22a
GOTERM BP	GO:0050877 neurological system process	14	9.76E-02	Obp83ef, scramb1, Obp51a, cher, Or69a, Gr85a, Rac1, Hsc70-4, Or49a, Galphaq, mth, FKBP59, Or22a

**Annotation Cluster 7**	**Enrichment Score: 1.1978454386369843**			
SWISSPROT PIR	mitochondrion inner membrane	5	1.41E-02	CG7394, ATPsyn-d, ATPsyn-b, Oscp, CG9603
GOTERM CC	GO:0005753 mitochondrial proton-transporting ATP synthase complex	3	5.02E-02	ATPsyn-d, ATPsyn-b, Oscp
SWISSPROT PIR	Hydrogen ion transport	3	5.21E-02	ATPsyn-d, ATPsyn-b, Oscp
GOTERM BP	GO:0009165 nucleotide biosynthetic process	6	5.54E-02	ATPsyn-d, pug, CG6767, Ada, ATPsyn-b, Oscp
GOTERM BP	GO:0009260 ribonucleotide biosynthetic process	5	5.67E-02	ATPsyn-d, CG6767, Ada, ATPsyn-b, Oscp
GOTERM BP	GO:0009259 ribonucleotide metabolic process	5	6.27E-02	ATPsyn-d, CG6767, Ada, ATPsyn-b, Oscp
GOTERM CC	GO:0045259 proton-transporting ATP synthase complex	3	6.31E-02	ATPsyn-d, ATPsyn-b, Oscp
GOTERM BP	GO:0034404 nucleobase, nucleoside and nucleotide biosynthetic process	6	7.04E-02	ATPsyn-d, pug, CG6767, Ada, ATPsyn-b, Oscp
GOTERM BP	GO:0034654 nucleobase, nucleoside, nucleotide and nucleic acid biosynthetic process	6	7.04E-02	ATPsyn-d, pug, CG6767, Ada, ATPsyn-b, Oscp
GOTERM BP	GO:0044271 nitrogen compound biosynthetic process	8	7.82E-02	ATPsyn-d, CG11899, pug, CG6767, Ada, ATPsyn-b, Odc1, Oscp

GOTERM BP	GO:0006164 purine nucleotide biosynthetic process	5	8.25E-02	ATPsyn-d, pug, Ada, ATPsyn-b, Oscp
GOTERM CC	GO:0044455 mitochondrial membrane part	6	8.29E-02	CG7394, ATPsyn-d, CG4769, ATPsyn-b, Oscp, CG9603
GOTERM BP	GO:0006163 purine nucleotide metabolic process	5	9.34E-02	ATPsyn-d, pug, Ada, ATPsyn-b, Oscp
GOTERM CC	GO:0044429 mitochondrial part	12	9.70E-02	CG7394, ATPsyn-d, mRpL11, CG4769, ATPsyn-b, Hmt-1, Dic1, CG11267, CG9547, mRpL20, Oscp, CG9603
GOTERM CC	GO:0005743 mitochondrial inner membrane	7	9.76E-02	CG7394, ATPsyn-d, CG4769, ATPsyn-b, Hmt-1, Oscp, CG9603
**Annotation Cluster 8**	**Enrichment Score: 1.1438990309544648**			
GOTERM MF	GO:0004867 serine-type endopeptidase inhibitor activity	4	4.68E-02	Acp76A, CG31515, Spn77Bc, CG3604
GOTERM MF	GO:0004866 endopeptidase inhibitor activity	4	8.08E-02	Acp76A, CG31515, Spn77Bc, CG3604
GOTERM MF	GO:0030414 peptidase inhibitor activity	4	9.79E-02	Acp76A, CG31515, Spn77Bc, CG3604
