# Supplementary material for: Genome of Drosophila suzukii, the Spotted Wing Drosophila
Source: G3 (Bethesda). 2013 Oct 18;3(12):2257–71. doi: 10.1534/g3.113.008185 (PMC3852387; doi:10.1534/g3.113.008185)
Supplement: Supporting Information [file supp_g3.113.008185_TableS8.pdf]

Table S8 GO term and functional classification enrichment analysis using DAVID for gene families that are expanded in the *Drosophila suzukii* genome as compared to the basal paraphyletic group of Drosophilidae including *D. ananassae, D. persimilis, D. pseudoobscura, D. willistoni, D. grimshawi, D. mojavensis*, and *D. virulis*.

CATEGORY	TERM	COUNT	P VALUE	GENE FAMILIES: REPRESENTATIVE GENE SYMBOLS
**Annotation Cluster 1**	**Enrichment Score: 2.3163292177655386**			
GOTERM BP	GO:0032269 negative regulation of cellular protein metabolic process	7	4.18E-04	Acp76A, PEK, Acp62F, Spn77Bc, Spn27A, nos, elav
GOTERM BP	GO:0051248 negative regulation of protein metabolic process	7	4.83E-04	Acp76A, PEK, Acp62F, Spn77Bc, Spn27A, nos, elav
GOTERM BP	GO:0045861 negative regulation of proteolysis	4	9.18E-04	Acp76A, Acp62F, Spn77Bc, Spn27A
GOTERM MF	GO:0004867 serine-type endopeptidase inhibitor activity	6	1.50E-03	Acp76A, CG31515, Acp62F, Spn77Bc, CG3604, Spn27A
GOTERM BP	GO:0032268 regulation of cellular protein metabolic process	10	1.56E-03	Acp76A, PEK, Acp62F, Adam, mRpL11, Tollo, Spn77Bc, Spn27A, nos, elav
GOTERM MF	GO:0004866 endopeptidase inhibitor activity	6	4.15E-03	Acp76A, CG31515, Acp62F, Spn77Bc, CG3604, Spn27A
GOTERM MF	GO:0030414 peptidase inhibitor activity	6	5.96E-03	Acp76A, CG31515, Acp62F, Spn77Bc, CG3604, Spn27A
GOTERM BP	GO:0010605 negative regulation of macromolecule metabolic process	13	7.20E-03	WRNexo, Acp76A, Acp62F, mRpL11, Spn77Bc, Spn27A, elav, Hsc70-4, PEK, E(spl)m8-HLH, fkh, lolal, nos
GOTERM MF	GO:0004857 enzyme inhibitor	6	2.91E-02	Acp76A, CG31515, Acp62F, Spn77Bc, CG3604, Spn27A

	activity			
INTERPRO	IPR000215:Protease inhibitor I4, serpin	3	3.64E-02	Acp76A, Spn77Bc, Spn27A
GOTERM BP	GO:0030162 regulation of proteolysis	4	4.37E-02	Acp76A, Acp62F, Spn77Bc, Spn27A
SMART	SM00093:SERPIN	3	4.49E-02	Acp76A, Spn77Bc, Spn27A
**Annotation Cluster 2**	**Enrichment Score: 2.268060950272713**			
GOTERM BP	GO:0006952 defense response	9	2.76E-03	CG6168, Sr-CI, Tollo, Drs, Lectin-galC1, TotA, TotM, Spn27A, GNBP1
GOTERM BP	GO:0006955 immune response	9	3.16E-03	Sr-CI, Tollo, Drs, Lectin-galC1, Rac1, TotA, TotM, Spn27A, GNBP1
GOTERM BP	GO:0042742 defense response to bacterium	6	3.74E-03	CG6168, Sr-CI, Drs, Lectin-galC1, TotA, GNBP1
GOTERM BP	GO:0009617 response to bacterium	6	8.23E-03	CG6168, Sr-CI, Drs, Lectin-galC1, TotA, GNBP1
GOTERM BP	GO:0050829 defense response to Gram-negative bacterium	4	1.70E-02	CG6168, Sr-CI, Drs, GNBP1
**Annotation Cluster 3**	**Enrichment Score: 1.875257851646743**			
UP_SEQ_FEATURE	glycosylation site:N-linked (GlcNAc...)	17	2.24E-03	Acp76A, Acp29AB, D2R, Gr59c, Mal-A2, Gr85a, TotA, Hsc70-4, PEK, scb, y, pgant3, Drs, CG32669, rt, mth, GNBP1
SWISSPROT PIR	glycoprotein	17	9.71E-03	Acp76A, Acp29AB, D2R, Gr59c, Mal-A2, Gr85a, TotA, Hsc70-4, PEK, scb, y, pgant3, Drs, CG32669, rt, mth, GNBP1

UP_SEQ_FEATURE	signal peptide	15	1.13E-02	Acp76A, Acp62F, Acp29AB, Mal-A2, TotA, Gpb5, Sgs3, PEK, scb, y, Tk, Drs, TotM, mth, GNBP1
GOTERM CC	GO:0005576 extracellular region	16	1.88E-02	Acp76A, Acp62F, Acp29AB, CheB42a, CG34049, cher, TotA, Gpb5, Spn27A, Sgs3, Tk, y, beat-Va, Drs, TotM, GNBP1
SWISSPROT PIR	signal	15	2.78E-02	Acp76A, Acp62F, Acp29AB, Mal-A2, TotA, Gpb5, Sgs3, PEK, scb, y, Tk, Drs, TotM, mth, GNBP1
SWISSPROT PIR	Secreted	9	4.34E-02	Sgs3, Acp76A, Acp62F, y, Tk, Acp29AB, Drs, TotA, TotM
**Annotation Cluster 4**	**Enrichment Score: 1.7532690160826376**			
GOTERM BP	GO:0006071 glycerol metabolic process	3	9.72E-03	Gpdh, CG18135, Gyk
GOTERM BP	GO:0019400 alditol metabolic process	3	9.72E-03	Gpdh, CG18135, Gyk
GOTERM BP	GO:0019751 polyol metabolic process	3	5.82E-02	Gpdh, CG18135, Gyk
**Annotation Cluster 5**	**Enrichment Score: 1.5779149847475604**			
GOTERM BP	GO:0045087 innate immune response	6	1.24E-02	Tollo, Drs, TotA, TotM, Spn27A, GNBP1
GOTERM BP	GO:0050832 defense response to fungus	3	3.85E-02	Drs, Spn27A, GNBP1

GOTERM BP	GO:0009620 response to fungus	3	3.85E-02	Drs, Spn27A, GNBP1
**Annotation Cluster 6**	**Enrichment Score: 1.4342861994852356**			
SWISSPROT PIR	Chaperone	5	2.67E-02	CG7394, CG11267, Cnx99A, Tcp-1zeta
GOTERM BP	GO:0006457 protein folding	6	3.49E-02	Hsc70-4, CG11267, Cnx99A, Tcp-1zeta, FKBP59
GOTERM MF	GO:0051082 unfolded protein binding	5	5.34E-02	Hsc70-4, CG11267, Cnx99A, Tcp-1zeta
**Annotation Cluster 7**	**Enrichment Score: 1.420967720788353**			
SWISSPROT PIR	calcium	8	1.08E-02	CG4733, CG4662, pgant3, CG17271, Cad74A, sunz, Tctp
GOTERM MF	GO:0005509 calcium ion binding	10	2.82E-02	CG4733, CG4662, pgant3, Cnx99A, CG17271, Cad74A, CG42255, sunz, Tctp
INTERPRO	IPR018249:EF-HAND 2	5	4.46E-02	CG4733, CG4662, CG17271, sunz
INTERPRO	IPR011992:EF-Hand type	5	6.38E-02	CG4733, CG4662, CG17271, sunz
INTERPRO	IPR018247:EF-HAND 1	5	9.08E-02	CG4733, CG4662, CG17271, sunz
**Annotation Cluster 8**	**Enrichment Score: 1.2794935055644372**			
GOTERM BP	GO:0051606 detection of stimulus	6	1.67E-02	Or49a, Calx, CheB42a, Galphaq, FKBP59
GOTERM BP	GO:0009628 response to abiotic stimulus	8	3.30E-02	shep, Calx, Galphaq, TotA, TotM, mth, FKBP59
GOTERM BP	GO:0009583 detection of light	4	5.14E-02	Calx, Galphaq, FKBP59

	stimulus			
GOTERM BP	GO:0009416 response to light stimulus	5	6.29E-02	Calx, Galphaq, TotA, FKBP59
GOTERM BP	GO:0009582 detection of abiotic stimulus	4	7.32E-02	Calx, Galphaq, FKBP59
GOTERM BP	GO:0009581 detection of external stimulus	4	8.80E-02	Calx, Galphaq, FKBP59
GOTERM BP	GO:0009314 response to radiation	5	9.62E-02	Calx, Galphaq, TotA, FKBP59
**Annotation Cluster 9**	**Enrichment Score: 1.211795352683188**			
INTERPRO	IPR007087:Zinc finger, C2H2-type	13	4.40E-02	CG6689, term, CG1647, CG16779, CG10669, Meics, CG5316, CG4360, CG3065, CG11966, CG4318
INTERPRO	IPR015880:Zinc finger, C2H2-like	13	5.12E-02	CG6689, CG1647, CG16779, CG10669, Meics, CG5316, CG4360, CG3065, CG11966, noi, CG4318
INTERPRO	IPR012934:Zinc finger, AD-type	5	6.03E-02	CG6689, CG1647, CG10669, Meics, CG4318
INTERPRO	IPR013087:Zinc finger, C2H2-type/integrase, DNA-binding	7	6.59E-02	CG11966, CG6689, CG16779, CG10669, Meics, CG4360, CG3065
SMART	SM00355:ZnF_C2H2	13	9.73E-02	CG6689, CG1647, CG16779, CG10669, Meics, CG5316, CG4360, CG3065, CG11966, noi, CG4318
**Annotation Cluster 10**	**Enrichment Score: 1.2042724915072422**			

SWISSPROT PIR	mitochondrion inner membrane	5	2.45E-02	CG7394, ATPsyn-d, ATPsyn-b, Oscp, CG9603
GOTERM BP	GO:0055085 transmembrane transport	5	6.29E-02	CG7394, ATPsyn-d, ATPsyn-b, CG32669, Oscp
GOTERM CC	GO:0005753 mitochondrial proton-transporting ATP synthase complex	3	6.36E-02	ATPsyn-d, ATPsyn-b, Oscp
GOTERM CC	GO:0005743 mitochondrial inner membrane	8	6.97E-02	CG7394, ATPsyn-d, CG5265, CG4769, ATPsyn-b, Hmt-1, Oscp, CG9603
SWISSPROT PIR	Hydrogen ion transport	3	7.01E-02	ATPsyn-d, ATPsyn-b, Oscp
GOTERM CC	GO:0045259 proton-transporting ATP synthase complex	3	7.95E-02	ATPsyn-d, ATPsyn-b, Oscp
GOTERM CC	GO:0005740 mitochondrial envelope	9	9.75E-02	CG7394, ATPsyn-d, CG5265, CG4769, ATPsyn-b, Hmt-1, Dic1, Oscp, CG9603
**Annotation Cluster 11**	**Enrichment Score: 1.1591583013355033**			
GOTERM CC	GO:0005886 plasma membrane	20	5.15E-02	D2R, Tollo, Gr59c, Or69a, Gr85a, Cnx99A, Rac1, Cad74A, Oscp, Pk1r, Mical, Syn1, Sr-CI, scb, Or49a, Calx, Galphaq, mth, FKBP59, GNBP1
GOTERM BP	GO:0007186 G-protein coupled receptor protein signaling pathway	10	5.98E-02	Or49a, Tk, D2R, Gr59c, Or69a, Galphaq, Gr85a, mth, Pk1r
GOTERM BP	GO:0050890 cognition	11	7.63E-02	Obp83ef, scb, Or49a, Obp51a, cher, Gr59c, Or69a, Galphaq, Gr85a, FKBP59

GOTERM BP	GO:0007600 sensory perception	9	7.76E-02	Obp83ef, Or49a, Obp51a, Gr59c, Or69a, Galphaq, Gr85a, FKBP59
SWISSPROT PIR	transducer	8	8.78E-02	Or49a, D2R, Gr59c, Or69a, Galphaq, Gr85a, mth, Pk1r
**Annotation Cluster 12**	**Enrichment Score: 1.145383017959024**			
GOTERM MF	GO:0043169 cation binding	44	5.96E-02	CG6689, term, CHORD, CG9715, CG10669, CG4769, CG10916, CG5316, MICAL-like, Irk2, Cnx99A, CG17271, Cad74A, Mical, sunz, CG3065, CG11966, mud, CG4318, CG4662, CG31019, pgant3, CG33552, CG32669, Tctp, CG1647, CG16779, Meics, CG4733, x16, Mal-A2, CG4360, alph, noi, Mcm2, CD98hc, Arc1, CG42255, CG5292, nos, SF1
GOTERM MF	GO:0043167 ion binding	44	6.26E-02	CG6689, term, CHORD, CG9715, CG10669, CG4769, CG10916, CG5316, MICAL-like, Irk2, Cnx99A, CG17271, Cad74A, Mical, sunz, CG3065, CG11966, mud, CG4318, CG4662, CG31019, pgant3, CG33552, CG32669, Tctp, CG1647, CG16779, Meics, CG4733, x16, Mal-A2, CG4360, alph, noi, Mcm2, CD98hc, Arc1, CG42255, CG5292, nos, SF1
GOTERM MF	GO:0046872 metal ion binding	42	9.82E-02	CG6689, term, CHORD, CG9715, CG10669, CG4769, CG10916, CG5316, MICAL-like, Irk2, Cnx99A, CG17271, Cad74A, Mical, sunz, CG3065, CG11966, mud, CG4318, CG4662, CG31019, pgant3, CG33552, CG32669, Tctp, CG1647, CG16779, Meics, CG4733, x16, CG4360, alph, noi, Mcm2, Arc1, CG42255, CG5292, nos, SF1
