# Supplementary material for: Genome of Drosophila suzukii, the Spotted Wing Drosophila
Source: G3 (Bethesda). 2013 Oct 18;3(12):2257–71. doi: 10.1534/g3.113.008185 (PMC3852387; doi:10.1534/g3.113.008185)
Supplement: Supporting Information [file supp_g3.113.008185_TableS9.pdf]

Table S9 GO term and functional classification enrichment analysis using DAVID for gene families that are expanded in the *Drosophila suzukii* genome as compared to species in the melanogaster subgroup, including *D. simulans, D. sechellia, D. yakuba, D. erecta, and D. melanogaster*.

CATEGORY	TERM	COUNT	P VALUE	GENE FAMILIES: REPRESENTATIVE GENE SYMBOLS
**Annotation Cluster 1**	**Enrichment Score: 1.840605394222266**			
GOTERM BP	GO:0006071 glycerol metabolic process	3	7.90E-03	Gpdh, CG18135, Gyk
GOTERM BP	GO:0019400 alditol metabolic process	3	7.90E-03	Gpdh, CG18135, Gyk
GOTERM BP	GO:0019751 polyol metabolic process	3	4.81E-02	Gpdh, CG18135, Gyk
**Annotation Cluster 2**	**Enrichment Score: 1.4947053163114195**			
GOTERM BP	GO:0010605 negative regulation of macromolecule metabolic process	13	3.03E-03	WRNexo, mRpL11, Spn77Bc, Su(var)205, aub, elav, mael, Hsc70-4, PEK, E(spl)m8-HLH, fkh, lolal, nos
GOTERM BP	GO:0032268 regulation of cellular protein metabolic process	9	3.07E-03	PEK, Adam, mRpL11, Tollo, Spn77Bc, aub, Su(var)205, nos, elav
GOTERM BP	GO:0006417 regulation of translation	6	1.01E-02	PEK, Adam, mRpL11, aub, nos, elav
GOTERM BP	GO:0006446 regulation of translational initiation	3	2.68E-02	PEK, Adam, aub
GOTERM BP	GO:0016458 gene silencing	6	2.90E-02	mael, Hsc70-4, mRpL11, aub, Su(var)205, lolal
GOTERM BP	GO:0010608 posttranscriptional regulation of gene expression	7	2.96E-02	Hsc70-4, PEK, Adam, mRpL11, aub, nos, elav
GOTERM BP	GO:0010629 negative regulation of	9	3.45E-02	mael, Hsc70-4, E(spl)m8-HLH, mRpL11, fkh, aub, Su(var)205, lolal,

	gene expression			nos
SWISSPROT PIR	translation regulation	3	3.66E-02	PEK, mRpL11, nos
GOTERM BP	GO:0031047 gene silencing by RNA	4	4.60E-02	mael, Hsc70-4, mRpL11, aub
GOTERM BP	GO:0032269 negative regulation of cellular protein metabolic process	4	6.06E-02	PEK, Spn77Bc, nos, elav
GOTERM BP	GO:0010558 negative regulation of macromolecule biosynthetic process	8	6.32E-02	E(spl)m8-HLH, PEK, fkh, aub, Su(var)205, lolal, nos, elav
GOTERM BP	GO:0051248 negative regulation of protein metabolic process	4	6.45E-02	PEK, Spn77Bc, nos, elav
GOTERM BP	GO:0009890 negative regulation of biosynthetic process	8	6.89E-02	E(spl)m8-HLH, PEK, fkh, aub, Su(var)205, lolal, nos, elav
GOTERM BP	GO:0031327 negative regulation of cellular biosynthetic process	8	6.89E-02	E(spl)m8-HLH, PEK, fkh, aub, Su(var)205, lolal, nos, elav
GOTERM BP	GO:0051172 negative regulation of nitrogen compound metabolic process	7	9.07E-02	WRNexo, E(spl)m8-HLH, fkh, aub, Su(var)205, lolal, nos
GOTERM BP	GO:0045934 negative regulation of nucleobase, nucleoside, nucleotide and nucleic acid metabolic process	7	9.07E-02	WRNexo, E(spl)m8-HLH, fkh, aub, Su(var)205, lolal, nos
**Annotation Cluster 3**	**Enrichment Score: 1.3768919446351504**			
INTERPRO	IPR013087:Zinc finger, C2H2-	7	3.95E-02	CG11966, CG6689, CG16779, CG10669, Meics, CG4360, CG3065

	type/integrase, DNA-binding			
INTERPRO	IPR012934:Zinc finger, AD-type	5	4.08E-02	CG6689, CG1647, CG10669, Meics, CG4318
INTERPRO	IPR007087:Zinc finger, C2H2-type	12	4.09E-02	CG11966, term, CG6689, CG1647, CG16779, CG10669, Meics, CG4318, CG5316, CG4360, CG3065
INTERPRO	IPR015880:Zinc finger, C2H2-like	12	4.72E-02	CG11966, CG6689, CG1647, CG16779, CG10669, Meics, noi, CG4318, CG5316, CG4360, CG3065
**Annotation Cluster 4**	**Enrichment Score: 1.3356641246312047**			
GOTERM BP	GO:0007314 oocyte anterior/posterior axis specification	5	2.30E-02	mael, Tm1, lkb1, aub, nos
GOTERM BP	GO:0007316 pole plasm RNA localization	4	2.53E-02	mael, Tm1, lkb1, aub
GOTERM BP	GO:0019094 pole plasm mRNA localization	4	2.53E-02	mael, Tm1, lkb1, aub
GOTERM BP	GO:0008358 maternal determination of anterior/posterior axis, embryo	5	2.64E-02	mael, Tm1, lkb1, aub, nos
GOTERM BP	GO:0007315 pole plasm assembly	4	3.94E-02	mael, Tm1, lkb1, aub
GOTERM CC	GO:0045495 pole plasm	3	4.31E-02	mael, aub, nos
GOTERM BP	GO:0009994 oocyte differentiation	6	5.08E-02	mael, asp, Tm1, lkb1, aub, nos
GOTERM BP	GO:0007028 cytoplasm organization	4	5.67E-02	mael, Tm1, lkb1, aub
GOTERM BP	GO:0007281 germ cell development	8	6.32E-02	mael, asp, Tm1, Klp64D, lkb1, aub, nos, elav

GOTERM BP	GO:0008298	intracellular mRNA localization	4	7.28E-02	mael, Tm1, lkb1, aub
GOTERM BP	GO:0017145	stem cell division	4	7.28E-02	asp, lkb1, aub, nos
GOTERM BP	GO:0007309	oocyte axis specification	5	7.75E-02	mael, Tm1, lkb1, aub, nos
GOTERM BP	GO:0007308	oocyte construction	5	8.77E-02	mael, Tm1, lkb1, aub, nos
**Annotation Cluster 5**	**Enrichment Score: 1.2102180736678618**				
GOTERM MF	GO:0043169	cation binding	41	5.03E-02	CG6689, term, CHORD, CG9715, CG10669, CG4769, CG10916, CG5316, LpR1, Irk2, CG17271, Cad74A, Mical, CG3065, CG11966, CG4318, CG4662, CG31019, pgant3, CG33552, Tctp, Tim13, CG1647, CG16779, Meics, CG4733, x16, Mal-A2, CG4360, alph, noi, Mcm2, CG6767, CD98hc, Arc1, CG42255, CG5292, nos, SF1
GOTERM MF	GO:0043167	ion binding	41	5.29E-02	CG6689, term, CHORD, CG9715, CG10669, CG4769, CG10916, CG5316, LpR1, Irk2, CG17271, Cad74A, Mical, CG3065, CG11966, CG4318, CG4662, CG31019, pgant3, CG33552, Tctp, Tim13, CG1647, CG16779, Meics, CG4733, x16, Mal-A2, CG4360, alph, noi, Mcm2, CG6767, CD98hc, Arc1, CG42255, CG5292, nos, SF1
GOTERM MF	GO:0046872	metal ion binding	39	8.79E-02	CG6689, term, CHORD, CG9715, CG10669, CG4769, CG10916, CG5316, LpR1, Irk2, CG17271, Cad74A, Mical, CG3065, CG11966, CG4318, CG4662, CG31019, pgant3, CG33552, Tctp, Tim13, CG1647, CG16779, Meics, CG4733, x16, CG4360, alph, noi, Mcm2, CG6767,

Arc1, CG42255, CG5292, nos, SF1
