# Supplementary material for: Genome of Drosophila suzukii, the Spotted Wing Drosophila
Source: G3 (Bethesda). 2013 Oct 18;3(12):2257–71. doi: 10.1534/g3.113.008185 (PMC3852387; doi:10.1534/g3.113.008185)
Supplement: Supporting Information [file supp_g3.113.008185_TableS10.pdf]

Table S10 GO term and functional classification enrichment analysis using DAVID for gene families that are contracted in the *Drosophila suzukii* genome as compared to 14 *Drosophila* species analyzed in this study.

CATEGORY	TERM	COUNT	P VALUE	GENE FAMILIES: REPRESENTATIVE GENE SYMBOLS
**Annotation Cluster 1**	**Enrichment Score: 3.023489574033364**			
GOTERM CC	GO:0000786 nucleosome	5	8.14E-06	His2B:CG33882, His2Av, His1:CG33834, His4r, His3.3A
SWISSPROT PIR	acetylation	5	2.48E-05	His2Av, Adh, Cam, His4r, His3.3A
SWISSPROT PIR	nucleosome core	4	2.71E-05	His2B:CG33882, His2Av, His4r, His3.3A
INTERPRO	IPR007125:Histone core	4	8.38E-05	His2B:CG33882, His2Av, His4r, His3.3A
GOTERM BP	GO:0006334 nucleosome assembly	5	9.51E-05	His2B:CG33882, His2Av, His1:CG33834, His4r, His3.3A
GOTERM BP	GO:0031497 chromatin assembly	5	3.55E-04	His2B:CG33882, His2Av, His1:CG33834, His4r, His3.3A
SWISSPROT PIR	chromosomal protein	5	7.88E-04	His2B:CG33882, His2Av, His1:CG33834, His4r, His3.3A
GOTERM BP	GO:0006323 DNA packaging	6	8.32E-04	eIF-4E, His2B:CG33882, His2Av, His1:CG33834, His4r, His3.3A
GOTERM BP	GO:0065004 protein-DNA complex assembly	5	9.25E-04	His2B:CG33882, His2Av, His1:CG33834, His4r, His3.3A
GOTERM CC	GO:0032993 protein-DNA complex	5	9.82E-04	His2B:CG33882, His2Av, His1:CG33834, His4r, His3.3A
GOTERM BP	GO:0034728 nucleosome organization	5	1.06E-03	His2B:CG33882, His2Av, His1:CG33834, His4r, His3.3A
GOTERM BP	GO:0006333 chromatin assembly or disassembly	5	2.96E-03	His2B:CG33882, His2Av, His1:CG33834, His4r, His3.3A
INTERPRO	IPR009072:Histone-fold	4	3.47E-03	His2B:CG33882, His2Av, His4r, His3.3A
GOTERM BP	GO:0034621 cellular macromolecular	7	2.60E-02	His2B:CG33882, His2Av, His1:CG33834, gammaTub23C, Cam, His4r,

	complex subunit organization			His3.3A
GOTERM BP	GO:0034622 cellular macromolecular complex assembly	6	3.27E-02	His2B:CG33882, His2Av, His1:CG33834, gammaTub23C, His4r, His3.3A
GOTERM BP	GO:0006325 chromatin organization	6	6.55E-02	eIF-4E, His2B:CG33882, His2Av, His1:CG33834, His4r, His3.3A
GOTERM CC	GO:0000785 chromatin	5	7.12E-02	His2B:CG33882, His2Av, His1:CG33834, His4r, His3.3A
**Annotation Cluster 2**	**Enrichment Score: 2.5911442503164333**			
SWISSPROT PIR	hydrolase	26	4.48E-06	CG1637, CG31821, S-Lap7, CG9449, Ace, CG30049, CG5731, Ance, PGRP-SA, CG14022, CG9391, ApepP, primo-2, CG14034, SPE, cathD, kraken, mag, CG2680, CG42264, CG31272, gd, LysC, Mdr49, CG6465, sda
GOTERM MF	GO:0008238 exopeptidase activity	6	3.74E-04	CG31821, ApepP, CG42264, S-Lap7, Ance, sda
GOTERM MF	GO:0008233 peptidase activity	12	2.21E-03	CG31821, CG30049, ApepP, CG42264, S-Lap7, gd, SPE, CG6465, cathD, Ance, sda, PGRP-SA
GOTERM BP	GO:0006508 proteolysis	11	1.06E-02	CG31821, CG30049, CG42264, Nedd8, S-Lap7, gd, SPE, CG6465, cathD, Ance, sda
GOTERM MF	GO:0070011 peptidase activity, acting on L-amino acid peptides	10	1.47E-02	CG31821, ApepP, CG42264, S-Lap7, gd, SPE, CG6465, cathD, Ance, sda
GOTERM MF	GO:0008237 metallopeptidase activity	5	3.28E-02	CG42264, S-Lap7, CG6465, Ance, sda
SWISSPROT PIR	Protease	5	3.85E-02	gd, SPE, cathD, Ance, PGRP-SA

**Annotation Cluster 3**	**Enrichment Score: 2.535780529609017**			
SWISSPROT PIR	disulfide bond	9	1.56E-03	crq, Adk2, gd, LysC, C1GalTA, Ace, CG5210, Ance, PGRP-SA
SWISSPROT PIR	glycoprotein	12	2.31E-03	PGRP-LE, crq, Orct2, Gr64a, pip, gd, Mdr49, C1GalTA, ninaG, Ace, CG5210, Ance
UP_SEQ_FEATURE	glycosylation site:N-linked (GlcNAc...)	12	6.87E-03	PGRP-LE, crq, Orct2, Gr64a, pip, gd, Mdr49, C1GalTA, ninaG, Ace, CG5210, Ance
**Annotation Cluster 4**	**Enrichment Score: 2.302060885904615**			
GOTERM MF	GO:0008238 exopeptidase activity	6	3.74E-04	CG31821, ApepP, CG42264, S-Lap7, Ance, sda
SWISSPROT PIR	carboxypeptidase	3	1.40E-02	CG31821, CG42264, Ance
GOTERM MF	GO:0004180 carboxypeptidase activity	3	2.37E-02	CG31821, CG42264, Ance
**Annotation Cluster 5**	**Enrichment Score: 2.2415486548709147**			
GOTERM MF	GO:0008238 exopeptidase activity	6	3.74E-04	CG31821, ApepP, CG42264, S-Lap7, Ance, sda
SWISSPROT PIR	Aminopeptidase	3	1.62E-02	ApepP, S-Lap7, sda
GOTERM MF	GO:0004177 aminopeptidase activity	3	3.11E-02	ApepP, S-Lap7, sda
**Annotation Cluster 6**	**Enrichment Score: 2.0347426002055675**			
SWISSPROT PIR	disulfide bond	9	1.56E-03	crq, Adk2, gd, LysC, C1GalTA, Ace, CG5210, Ance, PGRP-SA

SWISSPROT PIR	signal	11	4.87E-03	Acp1, Cpr47Eg, Acp53Ea, gd, LysC, ninaG, rumi, Ace, CG5210, Ance, PGRP-SA
SWISSPROT PIR	Secreted	7	1.17E-02	PGRP-LE, Acp53Ea, gd, ninaG, CG5210, Ance, PGRP-SA
UP_SEQ_FEATURE	disulfide bond	8	1.55E-02	crq, Adk2, gd, LysC, Ace, CG5210, Ance, PGRP-SA
UP_SEQ_FEATURE	signal peptide	11	1.60E-02	Acp1, Cpr47Eg, Acp53Ea, gd, LysC, ninaG, rumi, Ace, CG5210, Ance, PGRP-SA
GOTERM CC	GO:0005576 extracellular region	12	2.79E-02	PGRP-LE, Cpr47Eg, obst-A, Acp53Ea, CG6933, gd, ninaG, CG5210, Ance, PGRP-SA, Spn27A, CG17739
**Annotation Cluster 7**	**Enrichment Score: 1.8301156780099452**			
GOTERM BP	GO:0008063 Toll signaling pathway	5	5.04E-04	pip, gd, SPE, PGRP-SA, Spn27A
GOTERM BP	GO:0016485 protein processing	3	7.78E-02	pip, gd, SPE
GOTERM BP	GO:0051604 protein maturation	3	8.25E-02	pip, gd, SPE
**Annotation Cluster 8**	**Enrichment Score: 1.5505648241814785**			
GOTERM BP	GO:0006026 aminoglycan catabolic process	3	1.50E-02	PGRP-LE, CG5210, PGRP-SA
GOTERM BP	GO:0000272 polysaccharide catabolic process	3	1.76E-02	PGRP-LE, CG5210, PGRP-SA
GOTERM BP	GO:0006022 aminoglycan metabolic process	5	4.30E-02	PGRP-LE, obst-A, CG6933, CG5210, PGRP-SA

GOTERM BP	GO:0005976 polysaccharide metabolic process	5	5.51E-02	PGRP-LE, obst-A, CG6933, CG5210, PGRP-SA
**Annotation Cluster 9**	**Enrichment Score: 1.5454649543144068**			
GOTERM BP	GO:0006952 defense response	7	3.29E-03	PGRP-LE, crq, Tak1, LysC, SPE, PGRP-SA, Spn27A
GOTERM BP	GO:0006955 immune response	7	3.66E-03	PGRP-LE, crq, Tak1, LysC, SPE, PGRP-SA, Spn27A
GOTERM BP	GO:0045087 innate immune response	5	9.80E-03	PGRP-LE, Tak1, SPE, PGRP-SA, Spn27A
GOTERM BP	GO:0050830 defense response to Gram-positive bacterium	3	1.76E-02	PGRP-LE, SPE, PGRP-SA
GOTERM BP	GO:0042742 defense response to bacterium	4	2.55E-02	PGRP-LE, LysC, SPE, PGRP-SA
GOTERM BP	GO:0002786 regulation of antibacterial peptide production	3	2.62E-02	Tak1, SPE, PGRP-SA
GOTERM BP	GO:0002808 regulation of antibacterial peptide biosynthetic process	3	2.62E-02	Tak1, SPE, PGRP-SA
GOTERM BP	GO:0006963 positive regulation of antibacterial peptide biosynthetic process	3	2.62E-02	Tak1, SPE, PGRP-SA
GOTERM BP	GO:0002697 regulation of immune	3	3.99E-02	Tak1, SPE, PGRP-SA

	effector process			
GOTERM BP	GO:0002700 regulation of production of molecular mediator of immune response	3	3.99E-02	Tak1, SPE, PGRP-SA
GOTERM BP	GO:0002784 regulation of antimicrobial peptide production	3	3.99E-02	Tak1, SPE, PGRP-SA
GOTERM BP	GO:0002805 regulation of antimicrobial peptide biosynthetic process	3	3.99E-02	Tak1, SPE, PGRP-SA
GOTERM BP	GO:0002807 positive regulation of antimicrobial peptide biosynthetic process	3	3.99E-02	Tak1, SPE, PGRP-SA
GOTERM BP	GO:0009617 response to bacterium	4	4.07E-02	PGRP-LE, LysC, SPE, PGRP-SA
GOTERM BP	GO:0002759 regulation of antimicrobial humoral response	3	5.15E-02	Tak1, SPE, PGRP-SA
GOTERM BP	GO:0043900 regulation of multi-organism process	3	5.15E-02	Tak1, SPE, PGRP-SA
GOTERM BP	GO:0002831 regulation of response to biotic stimulus	3	5.15E-02	Tak1, SPE, PGRP-SA
GOTERM BP	GO:0002920 regulation of humoral immune response	3	5.15E-02	Tak1, SPE, PGRP-SA

SWISSPROT PIR	innate immunity	3	6.30E-02	PGRP-LE, Tak1, PGRP-SA
SWISSPROT PIR	immune response	3	7.08E-02	PGRP-LE, Tak1, PGRP-SA
**Annotation Cluster 10**	**Enrichment Score: 1.3185781035185091**			
SWISSPROT PIR	membrane	16	3.33E-02	C1GalTA, eca, Ace, rost, Cyp6g1, crq, Orct2, Tret1-1, Gr64a, Gr93c, pip, Drip, Mdr49, ppk19, GluRIIA, Tsp42Ee
SWISSPROT PIR	transmembrane	15	3.61E-02	C1GalTA, eca, rost, crq, Orct2, Tret1-1, Gr64a, Gr93c, pip, Drip, Mdr49, ppk19, GluRIIA, Tsp42Ee, CG32053
GOTERM CC	GO:0031224 intrinsic to membrane	21	9.22E-02	PGRP-LE, CG13078, C1GalTA, eca, CG11601, Ace, rost, crq, Orct2, Tret1-1, Gr64a, Gr93c, pip, Drip, Mdr49, PGRP-SA, Elo68alpha, ppk19, CG32053, Tsp42Ee, GluRIIA
**Annotation Cluster 11**	**Enrichment Score: 1.2323161117351618**			
SWISSPROT PIR	synapse	3	4.14E-02	Snap25, Ace, GluRIIA
GOTERM CC	GO:0045202 synapse	5	6.14E-02	eIF-4E, Snap25, CG31272, Ace, GluRIIA
SWISSPROT PIR	cell junction	3	7.90E-02	Snap25, Ace, GluRIIA
