# Supplementary material for: Genome of Drosophila suzukii, the Spotted Wing Drosophila
Source: G3 (Bethesda). 2013 Oct 18;3(12):2257–71. doi: 10.1534/g3.113.008185 (PMC3852387; doi:10.1534/g3.113.008185)
Supplement: Supporting Information [file supp_g3.113.008185_TableS11.pdf]

Table S11 GO term and functional classification enrichment analysis using DAVID for gene families that are contracted in the *Drosophila suzukii* genome as compared to the basal paraphyletic group of Drosophilidae including *D. ananassae, D. persimilis, D. pseudoobscura, D. willistoni, D. grimshawi, D. mojavensis*, and *D. virulis*.

CATEGORY	TERM	COUNT	P VALUE	GENE FAMILIES: REPRESENTATIVE GENE SYMBOLS
**Annotation Cluster 1**	**Enrichment Score: 2.6163350631215847**			
GOTERM CC	GO:0000786 nucleosome	5	2.00E-05	His2B:CG33882, His2Av, His1:CG33834, His4r, His3.3A
SWISSPROT PIR	nucleosome core	4	5.35E-05	His2B:CG33882, His2Av, His4r, His3.3A
INTERPRO	IPR007125:Histone core	4	1.79E-04	His2B:CG33882, His2Av, His4r, His3.3A
GOTERM BP	GO:0006334 nucleosome assembly	5	2.47E-04	His2B:CG33882, His2Av, His1:CG33834, His4r, His3.3A
GOTERM BP	GO:0034728 nucleosome organization	6	2.59E-04	His2B:CG33882, His2Av, His1:CG33834, Nipped-A, His4r, His3.3A
GOTERM BP	GO:0031497 chromatin assembly	5	9.05E-04	His2B:CG33882, His2Av, His1:CG33834, His4r, His3.3A
SWISSPROT PIR	acetylation	4	1.38E-03	His2Av, Adh, His4r, His3.3A
SWISSPROT PIR	chromosomal protein	5	1.83E-03	His2B:CG33882, His2Av, His1:CG33834, His4r, His3.3A
GOTERM CC	GO:0032993 protein-DNA complex	5	2.27E-03	His2B:CG33882, His2Av, His1:CG33834, His4r, His3.3A
GOTERM BP	GO:0065004 protein-DNA complex assembly	5	2.31E-03	His2B:CG33882, His2Av, His1:CG33834, His4r, His3.3A
GOTERM BP	GO:0006323 DNA packaging	6	2.50E-03	eIF-4E, His2B:CG33882, His2Av, His1:CG33834, His4r, His3.3A
INTERPRO	IPR009072:Histone-fold	4	7.08E-03	His2B:CG33882, His2Av, His4r, His3.3A
GOTERM BP	GO:0006333 chromatin assembly or disassembly	5	7.12E-03	His2B:CG33882, His2Av, His1:CG33834, His4r, His3.3A
GOTERM CC	GO:0035267 NuA4 histone	3	2.73E-02	His2B:CG33882, His2Av, Nipped-A

	acetyltransferase complex			
GOTERM CC	GO:0043189 H4/H2A histone acetyltransferase complex	3	4.89E-02	His2B:CG33882, His2Av, Nipped-A
GOTERM BP	GO:0006325 chromatin organization	7	5.87E-02	eIF-4E, His2B:CG33882, His2Av, His1:CG33834, Nipped-A, His4r, His3.3A
GOTERM BP	GO:0034621 cellular macromolecular complex subunit organization	7	7.13E-02	His2B:CG33882, His2Av, His1:CG33834, gammaTub23C, Nipped-A, His4r, His3.3A
GOTERM BP	GO:0034622 cellular macromolecular complex assembly	6	7.82E-02	His2B:CG33882, His2Av, His1:CG33834, gammaTub23C, His4r, His3.3A
**Annotation Cluster 2**	**Enrichment Score: 2.1187388547153736**			
GOTERM BP	GO:0006508 proteolysis	15	1.10E-03	lwr, CG31821, S-Lap7, Roc1a, SPE, cathD, CG11864, CG30049, CG42264, CG32486, Nedd8, Prosbeta3, gd, CG6465, Ance
GOTERM MF	GO:0008233 peptidase activity	13	3.84E-03	CG31821, ApepP, S-Lap7, SPE, cathD, CG11864, CG30049, CG42264, Prosbeta3, gd, CG6465, PGRP-SA, Ance
SWISSPROT PIR	Protease	7	5.19E-03	Prosbeta3, gd, SPE, cathD, CG11864, Ance, PGRP-SA
GOTERM MF	GO:0008238 exopeptidase activity	5	7.22E-03	CG31821, ApepP, CG42264, S-Lap7, Ance
GOTERM MF	GO:0070011 peptidase activity, acting on L-amino acid peptides	11	1.99E-02	CG31821, ApepP, CG42264, Prosbeta3, S-Lap7, gd, SPE, CG6465, cathD, CG11864, Ance
GOTERM MF	GO:0008237 metallopeptidase activity	5	6.15E-02	CG42264, S-Lap7, CG6465, CG11864, Ance

**Annotation Cluster 3**	**Enrichment Score: 2.085600214435509**			
SWISSPROT PIR	glycoprotein	14	1.64E-03	PGRP-LE, CG4928, C1GalTA, Ace, crq, Orct2, Gr64a, pip, gd, Mdr49, ninaG, prominin-like, CG5210, Ance
UP_SEQ_FEATURE	glycosylation site:N-linked (GlcNAc...)	14	6.01E-03	PGRP-LE, CG4928, C1GalTA, Ace, crq, Orct2, Gr64a, pip, gd, Mdr49, ninaG, prominin-like, CG5210, Ance
SWISSPROT PIR	membrane	18	5.62E-02	CG4928, C1GalTA, eca, Ace, rost, Cyp6g1, crq, Orct2, Tret1-1, Gr64a, Gr93c, pip, Drip, Mdr49, prominin-like, ppk19, GluRIIA, Tsp42Ee
**Annotation Cluster 4**	**Enrichment Score: 1.8933035516707737**			
SWISSPROT PIR	disulfide bond	10	1.72E-03	crq, Adk2, Amyrel, gd, LysC, C1GalTA, Ace, CG5210, Ance, PGRP-SA
SWISSPROT PIR	signal	12	8.54E-03	Acp1, Cpr47Eg, Amyrel, gd, LysC, ninaG, rumi, Dpt, Ace, CG5210, Ance, PGRP-SA
SWISSPROT PIR	Secreted	8	9.74E-03	PGRP-LE, Amyrel, gd, ninaG, Dpt, CG5210, Ance, PGRP-SA
UP_SEQ_FEATURE	disulfide bond	9	1.73E-02	crq, Adk2, Amyrel, gd, LysC, Ace, CG5210, Ance, PGRP-SA
UP_SEQ_FEATURE	signal peptide	12	3.10E-02	Acp1, Cpr47Eg, Amyrel, gd, LysC, ninaG, rumi, Dpt, Ace, CG5210, Ance, PGRP-SA
GOTERM CC	GO:0005576 extracellular region	13	5.70E-02	PGRP-LE, Cpr47Eg, Amyrel, CG17575, Dpt, obst-A, gd, ninaG, CG5210, Muc26B, PGRP-SA, Ance, CG17739
**Annotation Cluster 5**	**Enrichment Score: 1.7564554169324464**			

GOTERM MF	GO:0008238 exopeptidase activity	5	7.22E-03	CG31821, ApepP, CG42264, S-Lap7, Ance
SWISSPROT PIR	carboxypeptidase	3	2.15E-02	CG31821, CG42264, Ance
GOTERM MF	GO:0004180 carboxypeptidase activity	3	3.47E-02	CG31821, CG42264, Ance
**Annotation Cluster 6**	**Enrichment Score: 1.4889465105264814**			
GOTERM BP	GO:0006955 immune response	8	2.87E-03	lwr, PGRP-LE, crq, Tak1, LysC, SPE, Dpt, PGRP-SA
GOTERM BP	GO:0042742 defense response to bacterium	5	8.54E-03	PGRP-LE, LysC, SPE, Dpt, PGRP-SA
GOTERM BP	GO:0006952 defense response	7	1.08E-02	PGRP-LE, crq, Tak1, LysC, SPE, Dpt, PGRP-SA
SWISSPROT PIR	innate immunity	4	1.43E-02	PGRP-LE, Tak1, Dpt, PGRP-SA
GOTERM BP	GO:0009617 response to bacterium	5	1.61E-02	PGRP-LE, LysC, SPE, Dpt, PGRP-SA
SWISSPROT PIR	immune response	4	1.71E-02	PGRP-LE, Tak1, Dpt, PGRP-SA
GOTERM BP	GO:0045087 innate immune response	5	2.24E-02	PGRP-LE, Tak1, SPE, Dpt, PGRP-SA
GOTERM BP	GO:0050830 defense response to Gram-positive bacterium	3	2.79E-02	PGRP-LE, SPE, PGRP-SA
GOTERM BP	GO:0002786 regulation of antibacterial peptide production	3	4.12E-02	Tak1, SPE, PGRP-SA
GOTERM BP	GO:0002808 regulation of antibacterial peptide biosynthetic	3	4.12E-02	Tak1, SPE, PGRP-SA

	process			
GOTERM BP	GO:0006963 positive regulation of antibacterial peptide biosynthetic process	3	4.12E-02	Tak1, SPE, PGRP-SA
GOTERM BP	GO:0002697 regulation of immune effector process	3	6.20E-02	Tak1, SPE, PGRP-SA
GOTERM BP	GO:0002700 regulation of production of molecular mediator of immune response	3	6.20E-02	Tak1, SPE, PGRP-SA
GOTERM BP	GO:0002784 regulation of antimicrobial peptide production	3	6.20E-02	Tak1, SPE, PGRP-SA
GOTERM BP	GO:0002805 regulation of antimicrobial peptide biosynthetic process	3	6.20E-02	Tak1, SPE, PGRP-SA
GOTERM BP	GO:0002807 positive regulation of antimicrobial peptide biosynthetic process	3	6.20E-02	Tak1, SPE, PGRP-SA
GOTERM BP	GO:0043900 regulation of multi-organism process	3	7.94E-02	Tak1, SPE, PGRP-SA
GOTERM BP	GO:0002759 regulation of antimicrobial humoral response	3	7.94E-02	Tak1, SPE, PGRP-SA

GOTERM BP	GO:0002831 regulation of response to biotic stimulus	3	7.94E-02	Tak1, SPE, PGRP-SA
GOTERM BP	GO:0002920 regulation of humoral immune response	3	7.94E-02	Tak1, SPE, PGRP-SA
**Annotation Cluster 7**	**Enrichment Score: 1.3629891034285229**			
GOTERM BP	GO:0006026 aminoglycan catabolic process	3	2.39E-02	PGRP-LE, CG5210, PGRP-SA
GOTERM BP	GO:0000272 polysaccharide catabolic process	3	2.79E-02	PGRP-LE, CG5210, PGRP-SA
GOTERM BP	GO:0009057 macromolecule catabolic process	8	5.95E-02	lwr, PGRP-LE, Prosbeta3, Nedd8, CG32486, Roc1a, CG5210, PGRP-SA
GOTERM BP	GO:0006022 aminoglycan metabolic process	5	8.90E-02	PGRP-LE, obst-A, Muc26B, CG5210, PGRP-SA
