# Supplementary material for: Genome of Drosophila suzukii, the Spotted Wing Drosophila
Source: G3 (Bethesda). 2013 Oct 18;3(12):2257–71. doi: 10.1534/g3.113.008185 (PMC3852387; doi:10.1534/g3.113.008185)
Supplement: Supporting Information [file supp_g3.113.008185_TableS12.pdf]

Table S12 GO term and functional classification enrichment analysis using DAVID for gene families that are contracted in the *Drosophila suzukii* genome as compared to species in the melanogaster subgroup, including *D. simulans, D. sechellia, D. yakuba, D. erecta, and D. melanogaster*.

CATEGORY	TERM	COUNT	P VALUE	GENE FAMILIES: REPRESENTATIVE GENE SYMBOLS
**Annotation Cluster 1**	**Enrichment Score: 2.764122751765743**			
GOTERM BP	GO:0008063 Toll signaling pathway	6	9.04E-05	IM10, pip, gd, SPE, PGRP-SA, Spn27A
GOTERM BP	GO:0006952 defense response	9	4.43E-04	PGRP-LE, crq, IM10, Tak1, LysC, SPE, IM2, PGRP-SA, Spn27A
GOTERM BP	GO:0006955 immune response	9	5.13E-04	PGRP-LE, crq, IM10, Tak1, LysC, SPE, IM2, PGRP-SA, Spn27A
GOTERM BP	GO:0045087 innate immune response	7	5.86E-04	PGRP-LE, IM10, Tak1, SPE, IM2, PGRP-SA, Spn27A
SWISSPROT PIR	innate immunity	5	1.65E-03	PGRP-LE, IM10, Tak1, IM2, PGRP-SA
SWISSPROT PIR	immune response	5	2.12E-03	PGRP-LE, IM10, Tak1, IM2, PGRP-SA
GOTERM BP	GO:0042742 defense response to bacterium	5	7.91E-03	PGRP-LE, IM10, LysC, SPE, PGRP-SA
GOTERM BP	GO:0009617 response to bacterium	5	1.49E-02	PGRP-LE, IM10, LysC, SPE, PGRP-SA
GOTERM BP	GO:0050830 defense response to Gram-positive bacterium	3	2.68E-02	PGRP-LE, SPE, PGRP-SA
**Annotation Cluster 2**	**Enrichment Score: 2.735311860428723**			
GOTERM CC	GO:0000786 nucleosome	5	1.78E-05	His2B:CG33882, His2Av, His1:CG33834, His4r, His3.3A
SWISSPROT PIR	nucleosome core	4	5.45E-05	His2B:CG33882, His2Av, His4r, His3.3A
SWISSPROT PIR	acetylation	5	6.19E-05	His2Av, Adh, Cam, His4r, His3.3A

INTERPRO	IPR007125:Histone core	4	1.50E-04	His2B:CG33882, His2Av, His4r, His3.3A
GOTERM BP	GO:0006334 nucleosome assembly	5	2.27E-04	His2B:CG33882, His2Av, His1:CG33834, His4r, His3.3A
GOTERM BP	GO:0031497 chromatin assembly	5	8.33E-04	His2B:CG33882, His2Av, His1:CG33834, His4r, His3.3A
SWISSPROT PIR	chromosomal protein	5	1.87E-03	His2B:CG33882, His2Av, His1:CG33834, His4r, His3.3A
GOTERM CC	GO:0032993 protein-DNA complex	5	2.04E-03	His2B:CG33882, His2Av, His1:CG33834, His4r, His3.3A
GOTERM BP	GO:0065004 protein-DNA complex assembly	5	2.13E-03	His2B:CG33882, His2Av, His1:CG33834, His4r, His3.3A
GOTERM BP	GO:0006323 DNA packaging	6	2.27E-03	eIF-4E, His2B:CG33882, His2Av, His1:CG33834, His4r, His3.3A
GOTERM BP	GO:0034728 nucleosome organization	5	2.43E-03	His2B:CG33882, His2Av, His1:CG33834, His4r, His3.3A
INTERPRO	IPR009072:Histone-fold	4	6.00E-03	His2B:CG33882, His2Av, His4r, His3.3A
GOTERM BP	GO:0006333 chromatin assembly or disassembly	5	6.59E-03	His2B:CG33882, His2Av, His1:CG33834, His4r, His3.3A
GOTERM CC	GO:0000785 chromatin	6	4.10E-02	His2B:CG33882, His2Av, CG34317, His1:CG33834, His4r, His3.3A
GOTERM BP	GO:0006325 chromatin organization	7	5.38E-02	eIF-4E, His2B:CG33882, His2Av, CG34317, His1:CG33834, His4r, His3.3A
GOTERM BP	GO:0034621 cellular macromolecular complex subunit organization	7	6.55E-02	His2B:CG33882, His2Av, His1:CG33834, gammaTub23C, Cam, His4r, His3.3A
GOTERM BP	GO:0034622 cellular macromolecular complex assembly	6	7.26E-02	His2B:CG33882, His2Av, His1:CG33834, gammaTub23C, His4r, His3.3A

**Annotation Cluster 3**	**Enrichment Score: 2.6131075171385785**			
SWISSPROT PIR	disulfide bond	11	4.43E-04	crq, Adk2, gd, LysC, C1GalTA, IM2, Ace, CG5210, mth, Ance, PGRP-SA
SWISSPROT PIR	signal	14	1.07E-03	Acp1, Cpr47Eg, IM10, Acp53Ea, Ace, gd, LysC, rumi, ninaG, IM2, CG5210, PGRP-SA, mth, Ance
SWISSPROT PIR	Secreted	9	2.70E-03	PGRP-LE, IM10, Acp53Ea, gd, ninaG, IM2, CG5210, Ance, PGRP-SA
UP_SEQ_FEATURE	signal peptide	14	3.81E-03	Acp1, Cpr47Eg, IM10, Acp53Ea, Ace, gd, LysC, rumi, ninaG, IM2, CG5210, PGRP-SA, mth, Ance
UP_SEQ_FEATURE	disulfide bond	10	4.65E-03	crq, Adk2, gd, LysC, IM2, Ace, CG5210, mth, Ance, PGRP-SA
GOTERM CC	GO:0005576 extracellular region	15	9.25E-03	PGRP-LE, Cpr47Eg, IM10, CheB42a, Acp53Ea, Spn27A, CG6933, gd, ninaG, IM2, CG5210, Muc26B, PGRP-SA, Ance, CG17739
**Annotation Cluster 4**	**Enrichment Score: 2.452373611334107**			
SWISSPROT PIR	disulfide bond	11	4.43E-04	crq, Adk2, gd, LysC, C1GalTA, IM2, Ace, CG5210, mth, Ance, PGRP-SA
SWISSPROT PIR	glycoprotein	14	1.74E-03	PGRP-LE, IM10, C1GalTA, Ace, crq, Orct2, Gr64a, pip, gd, Mdr49, ninaG, CG5210, mth, Ance
UP_SEQ_FEATURE	glycosylation site:N-linked (GlcNAc...)	14	4.96E-03	PGRP-LE, IM10, C1GalTA, Ace, crq, Orct2, Gr64a, pip, gd, Mdr49, ninaG, CG5210, mth, Ance
UP_SEQ_FEATURE	topological domain:Cytoplasmic	11	4.06E-02	crq, Orct2, Gr64a, Gr93c, Or65a, pip, Mdr49, C1GalTA, Drip, Gr22f, mth
**Annotation Cluster 5**	**Enrichment Score: 2.2260947064240506**			

GOTERM MF	GO:0008238 exopeptidase activity	6	7.73E-04	CG31821, ApepP, CG42264, S-Lap7, Ance, sda
GOTERM MF	GO:0008233 peptidase activity	13	2.58E-03	CG31821, ApepP, S-Lap7, SPE, CG42370, cathD, CG30049, CG42264, gd, CG6465, PGRP-SA, sda, Ance
GOTERM MF	GO:0008237 metallopeptidase activity	6	1.34E-02	CG42264, S-Lap7, CG42370, CG6465, Ance, sda
GOTERM MF	GO:0070011 peptidase activity, acting on L-amino acid peptides	11	1.46E-02	CG31821, ApepP, CG42264, S-Lap7, gd, CG42370, SPE, CG6465, cathD, Ance, sda
GOTERM BP	GO:0006508 proteolysis	12	1.88E-02	CG31821, CG30049, CG42264, Nedd8, S-Lap7, gd, CG42370, SPE, CG6465, cathD, Ance, sda
**Annotation Cluster 6**	**Enrichment Score: 2.0907188176313323**			
GOTERM MF	GO:0008238 exopeptidase activity	6	7.73E-04	CG31821, ApepP, CG42264, S-Lap7, Ance, sda
SWISSPROT PIR	carboxypeptidase	3	2.18E-02	CG31821, CG42264, Ance
GOTERM MF	GO:0004180 carboxypeptidase activity	3	3.18E-02	CG31821, CG42264, Ance
**Annotation Cluster 7**	**Enrichment Score: 2.0311596068651054**			
GOTERM MF	GO:0008238 exopeptidase activity	6	7.73E-04	CG31821, ApepP, CG42264, S-Lap7, Ance, sda
SWISSPROT PIR	Aminopeptidase	3	2.51E-02	ApepP, S-Lap7, sda
GOTERM MF	GO:0004177 aminopeptidase activity	3	4.16E-02	ApepP, S-Lap7, sda

**Annotation Cluster 8**	**Enrichment Score: 1.8933328846785107**			
SWISSPROT PIR	transmembrane	22	1.60E-03	CG13796, Or65a, C1GalTA, eca, Gr22f, rost, crq, Orct2, Tret1-1, Gr64a, Gr93c, pip, CG32301, Drip, Mdr49, sesB, mth, ppk19, CG7255, CG32053, Tsp42Ee, GluRIIA
SWISSPROT PIR	membrane	21	8.80E-03	Or65a, C1GalTA, Gr22f, eca, Ace, rost, Cyp6g1, crq, Orct2, Tret1-1, Gr64a, Gr93c, pip, CG32301, Drip, Mdr49, sesB, mth, ppk19, Tsp42Ee, GluRIIA
GOTERM CC	GO:0031224 intrinsic to membrane	28	1.40E-02	PGRP-LE, CG13078, Or65a, eca, Gr22f, Ace, Gr93c, PGRP-SA, mth, ppk19, Elo68alpha, CG7255, CG32053, GluRIIA, Tsp42Ee, CG13796, C1GalTA, CG11601, rost, crq, Orct2, Tret1-1, Gr64a, pip, Drip, Mdr49, CG32301, sesB
GOTERM CC	GO:0016021 integral to membrane	27	2.10E-02	PGRP-LE, CG13078, Or65a, eca, Gr22f, Gr93c, PGRP-SA, mth, ppk19, Elo68alpha, CG32053, GluRIIA, Tsp42Ee, CG7255, CG13796, C1GalTA, CG11601, rost, crq, Orct2, Tret1-1, Gr64a, pip, Drip, Mdr49, CG32301, sesB
SWISSPROT PIR	cell membrane	8	2.59E-02	crq, Gr64a, Gr93c, Or65a, Gr22f, Ace, mth, GluRIIA
UP_SEQ_FEATURE	topological domain:Cytoplasmic	11	4.06E-02	crq, Orct2, Gr64a, Gr93c, Or65a, pip, Mdr49, C1GalTA, Drip, Gr22f, mth
**Annotation Cluster 9**	**Enrichment Score: 1.4296819844314266**			
GOTERM BP	GO:0006026 aminoglycan catabolic	3	2.29E-02	PGRP-LE, CG5210, PGRP-SA

	process			
GOTERM BP	GO:0000272 polysaccharide catabolic process	3	2.68E-02	PGRP-LE, CG5210, PGRP-SA
GOTERM BP	GO:0006022 aminoglycan metabolic process	5	8.37E-02	PGRP-LE, CG6933, Muc26B, CG5210, PGRP-SA
**Annotation Cluster 10**	**Enrichment Score: 1.4055451400858099**			
GOTERM BP	GO:0008219 cell death	8	9.69E-03	eIF-4E, Cyt-c-d, crq, qkr58E-3, Eig71Ej, Tak1, LysC, cathD
GOTERM BP	GO:0016265 death	8	1.01E-02	eIF-4E, Cyt-c-d, crq, qkr58E-3, Eig71Ej, Tak1, LysC, cathD
GOTERM BP	GO:0012501 programmed cell death	7	2.78E-02	eIF-4E, Cyt-c-d, crq, qkr58E-3, Eig71Ej, Tak1, cathD
GOTERM BP	GO:0035070 salivary gland histolysis	4	7.03E-02	eIF-4E, crq, Eig71Ej, cathD
GOTERM BP	GO:0035071 salivary gland cell autophagic cell death	4	7.03E-02	eIF-4E, crq, Eig71Ej, cathD
GOTERM BP	GO:0048102 autophagic cell death	4	7.03E-02	eIF-4E, crq, Eig71Ej, cathD
GOTERM BP	GO:0016271 tissue death	4	7.75E-02	eIF-4E, crq, Eig71Ej, cathD
GOTERM BP	GO:0007559 histolysis	4	7.75E-02	eIF-4E, crq, Eig71Ej, cathD
**Annotation Cluster 11**	**Enrichment Score: 1.3869132158526516**			
GOTERM BP	GO:0045087 innate immune response	7	5.86E-04	PGRP-LE, IM10, Tak1, SPE, IM2, PGRP-SA, Spn27A
GOTERM BP	GO:0002786 regulation of	3	3.96E-02	Tak1, SPE, PGRP-SA

	antibacterial peptide production			
GOTERM BP	GO:0002808 regulation of antibacterial peptide biosynthetic process	3	3.96E-02	Tak1, SPE, PGRP-SA
GOTERM BP	GO:0006963 positive regulation of antibacterial peptide biosynthetic process	3	3.96E-02	Tak1, SPE, PGRP-SA
GOTERM BP	GO:0002697 regulation of immune effector process	3	5.96E-02	Tak1, SPE, PGRP-SA
GOTERM BP	GO:0002700 regulation of production of molecular mediator of immune response	3	5.96E-02	Tak1, SPE, PGRP-SA
GOTERM BP	GO:0002784 regulation of antimicrobial peptide production	3	5.96E-02	Tak1, SPE, PGRP-SA
GOTERM BP	GO:0002805 regulation of antimicrobial peptide biosynthetic process	3	5.96E-02	Tak1, SPE, PGRP-SA
GOTERM BP	GO:0002807 positive regulation of antimicrobial peptide biosynthetic process	3	5.96E-02	Tak1, SPE, PGRP-SA
GOTERM BP	GO:0002759 regulation of	3	7.64E-02	Tak1, SPE, PGRP-SA

	antimicrobial humoral response			
GOTERM BP	GO:0002831 regulation of response to biotic stimulus	3	7.64E-02	Tak1, SPE, PGRP-SA
GOTERM BP	GO:0043900 regulation of multi-organism process	3	7.64E-02	Tak1, SPE, PGRP-SA
GOTERM BP	GO:0002920 regulation of humoral immune response	3	7.64E-02	Tak1, SPE, PGRP-SA
