# Supplementary material for: Genome of Drosophila suzukii, the Spotted Wing Drosophila
Source: G3 (Bethesda). 2013 Oct 18;3(12):2257–71. doi: 10.1534/g3.113.008185 (PMC3852387; doi:10.1534/g3.113.008185)
Supplement: Supporting Information [file supp_g3.113.008185_TableS13.pdf]

Table S13 Gene families of enzymes involved in metabolism and transport of xenobiotics.

Gene Family	Representative Genes	Dmel	Dsec	Dsim	Dere	Dyak	Dbia	Dsuz	Dtak	Dana	Dper	Dpse	Dwil	Dgri	Dmoj	Dvir
Esterases	Ace, α-Est2, α-Est7	31	34	28	32	35	23	**27**	23	34	34	35	43	28	36	35
GST	GstD2, GstD7, GstE12	25	28	25	26	28	8	**9**	7	31	20	21	29	21	19	21
GST	GstO1, GstO2, GstO3	4	5	5	5	5	1	**2**	1	5	5	5	5	3	3	4
GST	GstT1, GstT2, GstT3	4	4	4	4	4	1	**3**	2	5	4	4	4	3	3	3
GST	GstZ2,GstZ1	2	2	2	2	2	2	**3**	2	2	2	2	2	3	2	2
GST	GstS1	1	1	1	1	1	1	**1**	1	1	1	1	1	1	1	1
Cyp type 3, 4	Cyp4e1, Cyp6g1, Cyp6a2	68	78	72	67	74	52	**55**	56	79	65	66	78	72	61	64
mt Cyp	Cyp12d1, Cyp49a1	11	11	11	12	11	7	**10**	8	11	13	14	13	11	10	9
Cyp	Cyp1, Cyp33	8	9	7	8	8	5	**7**	6	7	6	7	8	11	12	10
Cyp type 2	Cyp18a1, Cyp303a1	4	4	4	4	5	3	**4**	3	4	6	4	4	5	5	6
UGT	Ugt35a, Ugt86Dd	35	37	34	32	41	24	**28**	26	39	30	31	43	30	29	32
UGT	Ugt	1	1	1	1	3	1	**1**	1	1	2	1	1	1	1	1
