# Supplementary material for: Genome of Drosophila suzukii, the Spotted Wing Drosophila
Source: G3 (Bethesda). 2013 Oct 18;3(12):2257–71. doi: 10.1534/g3.113.008185 (PMC3852387; doi:10.1534/g3.113.008185)
Supplement: Supporting Information [file supp_g3.113.008185_TableS14.pdf]

Table S14 Total base pairs of transposable element families.

≥50% ALIGNMENT SCORE				≥80% ALIGNMENT SCORE		
**TE**	**bp of TE**	**% Genome**		**TE**	**bp of TE**	**% Genome**
17.6	7795	3.31E-05		17.6	1033	4.39E-06
297	40503	1.72E-04		297	23676	1.01E-04
412	89430	3.80E-04		412	25241	1.07E-04
1360	17993	7.64E-05		1360	3173	1.35E-05
1731	17344	7.36E-05		1731	452	1.92E-06
3S18	54262	2.30E-04		3S18	8250	3.50E-05
accord	10494	4.45E-05		accord	981	4.16E-06
accord2	89195	3.79E-04		accord2	13350	5.67E-05
aurora	4156	1.76E-05		aurora	1252	5.32E-06
baggins	47296	2.01E-04		baggins	2802	1.19E-05
Bari1	4052	1.72E-05		Bari1	996	4.23E-06
blood	39870	1.69E-04		blood	18479	7.84E-05
Burdock	21218	9.01E-05		Burdock	2415	1.03E-05
Circe	47357	2.01E-04		Circe	6955	2.95E-05
copia	55228	2.34E-04		copia	14472	6.14E-05
Cr1a	541563	2.30E-03		Cr1a	81608	3.46E-04
diver2	289945	1.23E-03		diver2	26290	1.12E-04
Dm88	23210	9.85E-05		Dm88	1388	5.89E-06
Doc	9907	4.21E-05		Doc	1788	7.59E-06
Doc2	42396	1.80E-04		Doc2	4842	2.06E-05
Doc3	10373	4.40E-05		Doc3	2200	9.34E-06
Doc4	44326	1.88E-04		Doc4	3121	1.32E-05
F	208622	8.86E-04		F	13812	5.86E-05
FB	5512	2.34E-05		FB	2656	1.13E-05
flea	43854	1.86E-04		flea	4294	1.82E-05
Fw2	167825	7.12E-04		Fw2	13998	5.94E-05
Fw3	41439	1.76E-04		Fw3	2412	1.02E-05
G	2196	9.32E-06		G	0	0
G3	594	2.52E-06		G3	531	2.25E-06
G4	7218	3.06E-05		G4	2368	1.01E-05
G5	15951	6.77E-05		G5	1722	7.31E-06
G5A	78918	3.35E-04		G5A	8587	3.65E-05
G7	1520	6.45E-06		G7	109	4.63E-07
GATE	317288	1.35E-03		GATE	77748	3.30E-04
gtwin	70219	2.98E-04		gtwin	27040	1.15E-04
gypsy	70978	3.01E-04		gypsy	5478	2.33E-05
gypsy10	323555	1.37E-03		gypsy10	66652	2.83E-04
gypsy11	32727	1.39E-04		gypsy11	2630	1.12E-05

gypsy12	294106	1.25E-03
gypsy2	10478	4.45E-05
gypsy3	36524	1.55E-04
gypsy4	410628	1.74E-03
gypsy5	30021	1.27E-04
gypsy6	108613	4.61E-04
gypsy7	833	3.54E-06
gypsy8	342094	1.45E-03
gypsy9	8012	3.40E-05
H	5008	2.13E-05
HB	32745	1.39E-04
HeT-A	333	1.41E-06
HMS-Beagle	26650	1.13E-04
HMS-Beagle2	10678	4.53E-05
hopper	4361	1.85E-05
hopper2	9088	3.86E-05
I	175413	7.45E-04
Idefix	78608	3.34E-04
INE-1	5216305	2.21E-02
invader1	64950	2.76E-04
invader2	73534	3.12E-04
invader3	86528	3.67E-04
invader4	8146	3.46E-05
invader5	12282	5.21E-05
invader6	39958	1.70E-04
Ivk	61341	2.60E-04
jockey	31126	1.32E-04
jockey2	107	4.54E-07
KP	185	7.85E-07
looper1	1935	8.21E-06
mariner2	970	4.12E-06
Max	153290	6.51E-04
McClintock	1457	6.19E-06
mdg1	52859	2.24E-04
mdg3	15103	6.41E-05
micropia	15739	6.68E-05
ninja-Dsim-like	110289	4.68E-04
NOF	393	1.67E-06
opus	3177	1.35E-05
qbert	1881	7.99E-06
Quasimodo	133834	5.68E-04

gypsy12	53660	2.28E-04
gypsy2	2041	8.66E-06
gypsy3	8921	3.79E-05
gypsy4	118804	5.04E-04
gypsy5	3430	1.46E-05
gypsy6	30156	1.28E-04
gypsy7	113	4.80E-07
gypsy8	45774	1.94E-04
gypsy9	2176	9.24E-06
H	0	0
HB	433	1.84E-06
HeT-A	0	0
HMS-Beagle	9670	4.11E-05
HMS-Beagle2	83	3.52E-07
hopper	0	0
hopper2	735	3.12E-06
I	49824	2.12E-04
Idefix	13748	5.84E-05
INE-1	1438828	6.11E-03
invader1	7766	3.30E-05
invader2	25244	1.07E-04
invader3	33186	1.41E-04
invader4	3718	1.58E-05
invader5	3909	1.66E-05
invader6	10630	4.51E-05
Ivk	15462	6.56E-05
jockey	6908	2.93E-05
jockey2	0	0
KP	0	0
looper1	250	1.06E-06
mariner2	0	0
Max	29677	1.26E-04
McClintock	85	3.61E-07
mdg1	5507	2.34E-05
mdg3	6040	2.56E-05
micropia	5766	2.45E-05
ninja-Dsim-like	44565	1.89E-04
NOF	0	0
opus	771	3.27E-06
qbert	0	0
Quasimodo	12155	5.16E-05

R1	5225	2.22E-05
R1-2	3144	1.33E-05
R1-element	9080	3.85E-05
roo	102780	4.36E-04
rooA	119349	5.07E-04
rover	38812	1.65E-04
Rt1a	273	1.16E-06
Rt1b	9200	3.91E-05
Rt1c	14821	6.29E-05
S	5668	2.41E-05
S2	400	1.70E-06
springer	25730	1.09E-04
Stalker	10628	4.51E-05
Stalker2	137186	5.82E-04
Stalker4	108913	4.62E-04
Tabor	256912	1.09E-03
TART-A	1406	5.97E-06
TART-B	3682	1.56E-05
Tc1	12605	5.35E-05
Tc1-2	3073	1.30E-05
Tc3	2926	1.24E-05
Tirant	17853	7.58E-05
transib1	18138	7.70E-05
transib2	20048	8.51E-05
transib3	41264	1.75E-04
transib4	1384	5.88E-06
Transpac	2560	1.09E-05
X	3605	1.53E-05
total	11542576	4.90E-02

R1	588	2.50E-06
R1-2	166	7.05E-07
R1-element	2392	1.02E-05
roo	25656	1.09E-04
rooA	18105	7.69E-05
rover	18695	7.94E-05
Rt1a	0	0
Rt1b	897	3.81E-06
Rt1c	0	0
S	462	1.96E-06
S2	0	0
springer	5239	2.22E-05
Stalker	2237	9.50E-06
Stalker2	49025	2.08E-04
Stalker4	11657	4.95E-05
Tabor	92855	3.94E-04
TART-A	437	1.86E-06
TART-B	468	1.99E-06
Tc1	472	2.00E-06
Tc1-2	0	0
Tc3	326	1.38E-06
Tirant	680	2.89E-06
transib1	3684	1.56E-05
transib2	1832	7.78E-06
transib3	6329	2.69E-05
transib4	0	0
Transpac	0	0
X	285	1.21E-06
total	2707323	1.15E-02
